# The BRD9/7 Inhibitor TP-472 Blocks Melanoma Tumor Growth by Suppressing ECM-Mediated Oncogenic Signaling and Inducing Apoptosis

**DOI:** 10.3390/cancers13215516

**Published:** 2021-11-03

**Authors:** Lawrence David Mason, Suresh Chava, Kiran Kumar Reddi, Romi Gupta

**Affiliations:** 1 Department of Biochemistry and Molecular Genetics, University of Alabama at Birmingham, Birmingham, AL 35322, USA; mason8@uab.edu (L.D.M.); schava@uab.edu (S.C.); kreddi@uab.edu (K.K.R.); 2 O’Neal Comprehensive Cancer Center, University of Alabama at Birmingham, Birmingham, AL 35322, USA

**Keywords:** melanoma, epigenetic regulators, extracellular matrix, bromodomain, chromatin

## Abstract

**Simple Summary:**

Melanoma is an aggressive form of skin cancer and the leading cause of skin cancer-related deaths. Current therapies, including those targeting oncogenic pathways and immunotherapies, provide therapeutic benefits to only a subset of melanoma patients. Therefore, more options for therapeutic interventions are needed. Epigenetic alterations play an important role in tumor development and progression. In this study, we identified that TP-472 a small molecule inhibitor of BRD7/9 blocks melanoma tumor growth in cell cultures and in mouse models of melanoma growth. Further studies revealed that TP-472 downregulates cancer-promoting signaling pathways and induces cell death. Thus, this study identifies TP-472 as a potentially useful therapeutic agent for melanoma therapy.

**Abstract:**

Melanoma accounts for the majority of all skin cancer-related deaths and only 1/3rd of melanoma patients with distal metastasis survive beyond five years. However, current therapies including BRAF/MEK targeted therapies or immunotherapies only benefit a subset of melanoma patients due to the emergence of intrinsic or extrinsic resistance mechanisms. Effective treatment of melanoma will thus require new and more effective therapeutic agents. Towards the goal of identifying new therapeutic agents, we conducted an unbiased, druggable epigenetic drug screen using a library of 32 epigenetic inhibitors obtained from the Structural Genome Consortium that targets proteins encoding for epigenetic regulators. This chemical genetic screening identified TP-472, which targets bromodomain-7/9, as the strongest inhibitor of melanoma growth in both short- and long-term survival assays and in mouse models of melanoma tumor growth. Mechanistically, using a transcriptome-wide mRNA sequencing profile we identified TP-472 treatment downregulates genes encoding various extracellular matrix (ECM) proteins, including integrins, collagens, and fibronectins. Reactome-based functional pathway analyses revealed that many of the ECM proteins are involved in extracellular matrix interactions required for cancer cell growth and proliferation. TP-472 treatment also upregulated several pro-apoptotic genes that can inhibit melanoma growth. Collectively, our results identify BRD7/9 inhibitor TP-472 as a potentially useful therapeutic agent for melanoma therapy.

## 1. Introduction

Melanoma is the deadliest form of skin cancer, accounting for 85% of all skin cancer–related deaths [1]. The five-year survival rate for melanoma with distal metastasis is below 30% [2]. Previous genome-scale sequencing studies have identified mutations in a number of genes involved in the initiation and progression of melanoma, including *BRAF* (50%), *NRAS* (20–30%), neurofibromin 1 (*NF1*) (10–15%), and cyclin-dependent kinase inhibitor 2A (*CDKN2A*) (20–40%) [3]. Current therapies for advanced melanoma include several clinically approved BRAF inhibitors (BRAFi; e.g., vemurafenib, dabrafenib) in combination with MEK inhibitors (MEKi; e.g., trametinib) [4,5]. Although BRAFi/MEKi combination treatments produce impressive initial clinical responses in a subset of *BRAF*-mutant advanced melanoma patients, resistance to treatment rapidly emerges within a few months, rendering the therapy ineffective [6,7]. Similarly, advanced melanoma patients can develop either intrinsic or acquired resistance to various immunotherapies following the initial response [8]. Therefore, additional new therapeutic approaches are necessary for treating advanced melanoma.

Epigenetic regulators have been shown to play an important role in both normal and cancer cells [9]. In particular, epigenetic regulators influence multiple aspects of tumorigenesis which includes regulating the expression of tumor suppressor genes and oncogenes, modulating signaling pathways resulting in enhanced cancer growth, invasion, and metastases [10]. They have also been associated with drug resistance and predicting response to the treatment [11]. Therefore, targeting epigenetic regulators can be used as an alternative cancer therapy [10]. Based on this rationale, several drugs targeting epigenetic regulators have received approval for clinical use [12].

Guided by these previous observations and successful clinical translation of drugs targeting epigenetic regulators we performed a chemical genetic screen in BRAF mutant melanoma cells. Our goal was to identify a candidate epigenetic regulator targeting drug that could effectively inhibit the growth of BRAF mutant melanoma cells. To do so, we used a library of 32 small molecule inhibitors obtained from the Structural Genome Consortium (SGC) targeting protein products of genes encoding epigenetic regulators. This chemical genetic screening identified TP-472, a small molecule inhibitor of bromodomain-7/9 (BRD7/9) as the strongest inhibitor of BRAF mutant melanoma cell growth in both short- and long-term survival assays, as well as in a human melanoma cell line xenograft-based mouse model of melanoma tumor growth. We observed that BRD7/9 are overexpressed in melanoma patient samples at both the mRNA and protein levels, and their overexpression is associated with a poor prognosis. Additionally, we performed mRNA sequencing analyses to elucidate the mechanism by which TP-472 inhibits the growth of melanoma cells. These analyses revealed that TP-472 treatment leads to reduced extracellular matrix (ECM)-mediated oncogenic signaling and increased apoptosis that promotes inhibition of melanoma growth. Collectively, our results identify TP-472 as a potentially useful candidate drug for melanoma therapy.

## 2. Materials and Methods

### 2.1. Cell Culture

The melanoma cell lines SKMEL-28, A375, A2058, and M14 were purchased from the American Type Culture Collection and grown as recommended. Cells were maintained in a humidified atmosphere of 5% CO_2_ at 37 °C in Dulbecco’s modified Eagle medium (Life Technologies, Carlsbad, CA, USA) or Roswell Park Memorial Institute 1640 medium (Life Technologies), each supplemented with 10% fetal bovine serum (Life Technologies) and 1% penicillin/streptomycin (Life Technologies).

### 2.2. Chemical Genetics Screen Using Small Molecule Inhibitors Targeting Specific Epigenetic Regulators

The screen was performed using the Structural Genome Consortium’s epigenetic chemical probe inhibitor library (Cat. No. 17525), targeting 32 genes encoding epigenetic regulators, obtained from Cayman Chemical. All of the inhibitors were dissolved in DMSO to prepare 10 mM stocks. The inhibitors and their targets are listed in Table S1. M14 and SKMEL-28 cells were seeded in 96-well plates (1 × 10^3^) and treated with different doses of small molecule inhibitors (listed in Table S1) or DMSO as a control. After five days of treatment with the inhibitors, cell viability was evaluated using the MTT assay.

### 2.3. Chemical Inhibitors

All inhibitors were purchased from Cayman chemicals and dissolved for cell culture work as well as in vivo experiments as suggested in the datasheet. Information regarding the inhibitors is shown in Table S1. Treatment conditions are described in the corresponding figure legends.

### 2.4. Analysis of mRNA Expression Using Patient-Derived Melanoma Samples

Datasets for gene expression in melanoma and normal skin samples were identified via a search of the Oncomine cancer profiling database. The Talantov dataset was used in the present study [13] and includes 45 cutaneous melanoma samples and seven normal skin samples analyzed using a Human Genome U133A array. Relative expression and their significance are shown. The Riker dataset includes 40 metastasis samples and 16 primary-site samples from 87 patients analyzed on a Human Genome U133 Plus 2.0 Array [14]). The Xu dataset includes 12 stage 1, 11 stage II, and 3 stage II samples analyzed on a Human Genome U133A Array [15]. To analyze the correlation of the mRNA expression levels of BRD7 and ECM genes, we downloaded their expression data from the Talantov melanoma dataset [13]. We then calculated the Pearson correlation coefficients for each dataset using GraphPad Prism, version 9.0 for Macintosh (GraphPad Software, San Diego, CA, USA; www.graphpad.com accessed on 6 October 2021).

### 2.5. Protein Expression Analysis of Patient-Derived Melanoma Samples from the Human Tissue Atlas Dataset Using Immunohistochemistry (IHC)

The Human Protein Atlas is a publicly available database containing millions of high-resolution images showing the spatial distribution of proteins detected with 15,598 different antibodies (release 9.0, November 2011) in 46 different normal human tissue types and 20 different cancer types, as well as 47 different human cell lines. Samples containing normal and cancerous tissue were collected and paraffin-embedded following approval by the local ethics committee. Each antibody listed in the database was used for IHC staining of both normal and cancerous tissue. The Human Tissue Atlas Dataset was used to calculate the three-year survival high and three-year survival low rates for patients based on epigenetic regulator expression.

### 2.6. RNA Sequencing and Data Analysis

A375 cells were treated with vehicle control (DMSO) or 5 μM or 10 μM TP-472 for 24 h, after which total RNA was extracted for analysis of gene expression on an Illumina HiSeq 2500 system. Total RNA was extracted using TRIzol^®^ reagent (Invitrogen, Waltham, MA, USA) according to the manufacturer’s instructions and purified using RNAeasy mini columns (Qiagen, Hilden, Germany) according to the manufacturer’s instructions. Finally, mRNA was purified from approximately 500 ng of total RNA using oligo-dT beads and sheared by incubation at 94 °C. Following first-strand synthesis with random primers, second-strand synthesis was performed with dUTP to generate strand-specific libraries. The resulting cDNA libraries were then end-repaired and A-tailed. Adapters were ligated, and second-strand digestion was performed using uracil-DNA-glycosylase. Indexed libraries that met appropriate cutoffs for both were quantified by qRT-PCR using a commercially available kit (KAPA Biosystems, Wilmington, MA, USA). The insert-size distribution was determined using LabChip GX or an Agilent Bioanalyzer. Samples with a yield ≥0.5 ng/μL were sequenced on an Illumina HiSeq 2500 system. Images were converted into nucleotide sequences using the base-calling pipeline RTA 1.18.64.0 and stored in FASTQ format.

### 2.7. MTT Assay

For MTT assays, 1 × 10^3^ cells in a 100 µL volume were aliquoted to triplicate wells in 96-well plates. After 24 h, inhibitor samples were prepared at a range of concentrations in 100 µL of medium and added to the cells. Cells were treated with inhibitors for five days, after which cell viability was evaluated by adding 20 µL of 5 mg/mL MTT dissolved in 1× phosphate-buffered saline to each well and incubating for 1 h at 37 °C. The MTT solution was then gently removed, and 100 µL of dimethyl sulfoxide (DMSO) was added to each well. After mixing each well by repeated pipetting, the absorbance was measured at 590 and 630 nm. The average absorbance was calculated for both wavelengths, and then the measurement value at 630 nm was subtracted from that at 590 nm. Relative cell survival was plotted relative to vehicle-treated control cells.

### 2.8. Clonogenic Assay

The clonogenic capacity of cells was assayed following treatment with JQ1, GSK343, and TP-472 at concentrations of 5 μM and 10 μM. For clonogenic assays, a total of 1000 cells (A375, SKMEL-28, M14, and A2058) were seeded in triplicate wells of a 6-well plate and incubated for 24 h, at which time the cells were treated with vehicle or inhibitor. After 3–4 weeks, colonies were fixed using a fixing solution containing 50% methanol and 10% acetic acid and then stained with 0.05% Coomassie blue (Sigma-Aldrich, St. Louis, MO, USA). The relative number of colonies was calculated by first counting the number of colonies for each sample and then plotting the average number of colonies counted for triplicate wells under the indicated conditions.

### 2.9. Mouse Tumorigenesis Experiment with TP-472 Treatment

A375-MA2 (5 × 10^6^) cells were then injected subcutaneously into male five- to six-week-old NSG mice (Jackson Laboratory, Bar Harbor, ME, USA, stock No. 005557). We used six animals in each group. Tumor volume was measured weekly. Tumor size was calculated using the following formula: length × width^2^ × 0.5. When the tumor volume reached approximately 80–100 mm^3^, vehicle (0.5% methyl cellulose in water) or TP-472 (20 mg/kg body weight) was administered intraperitoneally every other day until the end of the experimental period. At the end of the experiment, tumor volume was measured, mice were sacrificed and the images of the tumors were captured. All protocols were approved by the University of Alabama, Birmingham, Institutional Animal Care and Use Committee. The ASP# for this study is IACUC-21684. To calculate *p*-values we used a non-parametric Student’s test. The Student’s *t*-test is a standard approach for calculating *p*-values.

### 2.10. Apoptosis Assays

Melanoma cells (A375, SKMEL-28, M14, and A2058) were seeded at a density of 3 × 10^3^ cells/well in 75 µL of medium in white TC-treated clear-bottom 96-well plates (Costar, Corning, NY, USA, Cat. No. #3610) and incubated for 24  h at 37 °C, 95% relative humidity, and 5% CO_2_. The cells were then treated with vehicle (DMSO) or 10 µM TP-472 for 48 h, followed by immediate addition of Real Time-Glo Annexin V apoptosis reagent (Promega Corp., Madison, WI, USA, Cat. No. # JA1011). Luminescence was monitored using a Biotek Synergy MX Multi-Format Microplate Reader.

### 2.11. Soft Agar Assay

For the soft agar assay, 2 × 10^3^ A375 cells were seeded into a 0.4% soft agar layer. After 24 h, the cells were treated with various concentrations of TP-472 as indicated in the figure. After two–three weeks, images of colonies formed in the soft agar were taken using an inverted light microscope. The colonies were stained with 0.005% crystal-violet solution and counted. The average colony area of each sample was calculated using Image J software (NIH, Bethesda, MD, USA) and plotted. Each experiment was repeated at least twice.

### 2.12. Immunoblot Analysis

Whole-cell protein extracts were prepared using RIPA lysis buffer (Pierce) containing Protease Inhibitor Cocktail (Roche, Basel, Switzerland) and Phosphatase Inhibitor Cocktail (Sigma-Aldrich). The protein concentration was estimated using a Bradford Assay kit (Bio-Rad, Hercules, CA, USA). Proteins were resolved on 6%, 8%, 10%, or 15% polyacrylamide gels and transferred to PVDF membranes using a wet transfer apparatus from Bio-Rad. The membranes were blocked with 5% skim milk and probed with primary antibodies followed by the appropriate ECL-grade secondary HRP-conjugated antibody (GE Healthcare, Chicago, IL, USA). The blots were developed using the Supersignal Pico or Femto Reagent (Pierce Biotechnology, Waltham, MA, USA), as necessary. The details of the antibodies are provided in Table S2.

### 2.13. Matrigel Invasion Assay

Invasion assays were performed in BioCoat Growth Factor Reduced Matrigel Invasion Chambers (Cat #354483, BD Biosciences, Franklin Lakes, NJ, USA), using A375 cells under different treatment conditions. Cells were serum-starved for 6 h, and then 5 × 10^4^ cells/insert were seeded in triplicate in the top chamber containing a low-serum medium. Cells were incubated for 20 h to allow invasion toward the serum-rich medium in the bottom well. The number of cells invading the Matrigel was quantified by DAPI staining and imaging; 8–12 fields per membrane were counted, and quantification of nuclei was performed using ImageJ software. Each experiment was repeated at least three times.

### 2.14. Wound Healing Assay

A375 cells were seeded at a density of 2 × 10^5^ cells per well and grown in 6-well plates until confluent. A scratch was then created using a sterile 10-µL pipette tip. Cells were then either treated with DMSO or TP-472 and cell migration into the wound was monitored at 0 and 24 h using light microscopy. Quantification of wound healing was performed using ImageJ software. Each experiment was repeated at least three times.

### 2.15. Statistical Analyses

All experiments were conducted with at least three biological replicates. Results for individual experiments are expressed as the mean ± standard error of the mean (SEM). Statistical analyses of tumor progression in mice were performed using the area under the curve method in GraphPad Prism, version 9.0 for Macintosh (GraphPad Software, San Diego, CA, USA; www.graphpad.com accessed on 6 October 2021). *p*-values for the remaining experiments were calculated using the two-tailed unpaired Student’s *t*-test in GraphPad Prism, version 9.0 for Macintosh (GraphPad Software). In figures, ns, *, **, ***, and **** indicate non-significant *p*-value, *p* < 0.05, <0.01, <0.001, and <0.0001, respectively. BioRender software tool was used for drawing images presented in the manuscript.

## 3. Results

### 3.1. New Drug Candidates for BRAF-Mutant Melanoma Therapy Identified by Chemical Genetic Screening Using Small-Molecule Inhibitors of Epigenetic Regulators

**Figure 1:**
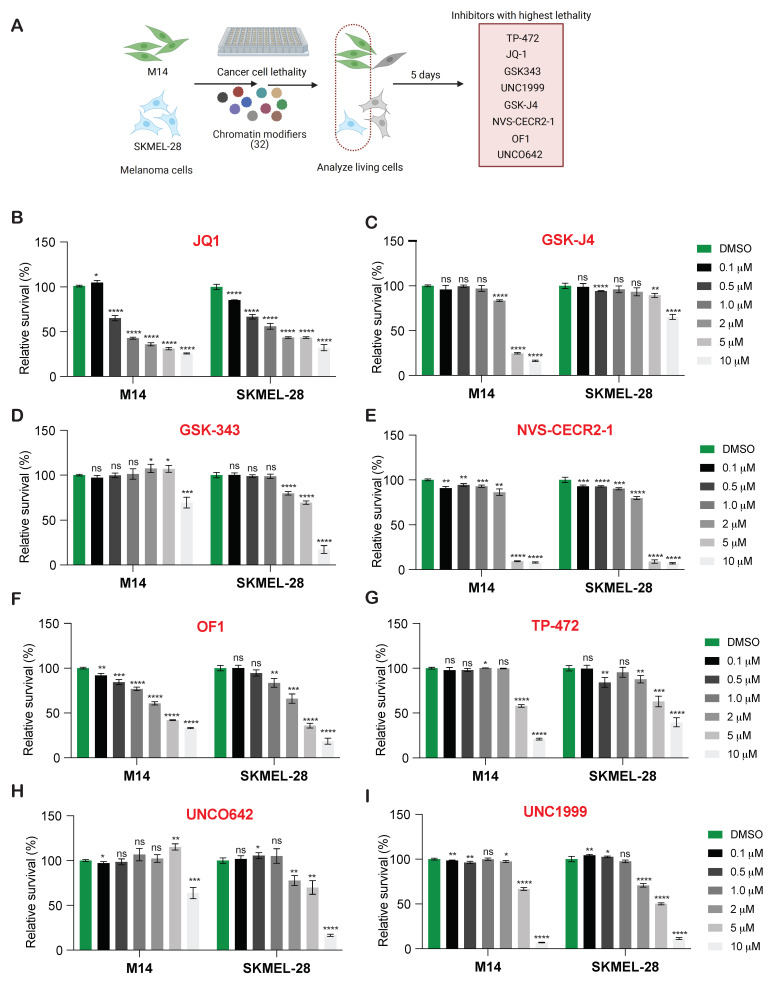
Pharmacological inhibition of epigenetic chromatin modifiers inhibits melanoma growth in short-term survival assays. (**A**). Schematic illustration of the druggable screening approach using 32 different epigenetic regulators with 2 different melanoma cell lines (M14 and SKMEL-28). (**B**–**I**). Indicated melanoma cell lines were treated with different concentrations of various epigenetic regulators for 5 days and analyzed for survival using the MTT assay. Relative cell survival is plotted relative to vehicle-treated cells. Data represent the mean ± SEM. * *p* < 0.05, ** *p* < 0.01, *** *p* < 0.001, **** *p* < 0.0001, ns = not significant.

Epigenetic regulators play an important role in tumor growth and metastasis [9,16]. Therefore, to determine if targeting these epigenetic regulators will have an effect on BRAF-mutant melanoma growth, we conducted a chemical genetics screen using the Structural Genome Consortium’s epigenetic chemical probe library, containing 32 small molecule inhibitors targeting proteins encoding epigenetic regulators (Table S1). We first tested the effect of these inhibitors on the short-term survival of BRAF mutant melanoma cells using the MTT assay. To do so, two different BRAF mutant melanoma lines (M14 and SKMEL-28) were treated with increasing concentrations of the 32 inhibitors for five days, after which cell survival was assessed (Figure 1A). A total of eight inhibitors targeting epigenetic regulators significantly inhibited melanoma cell survival (Table S3). JQ1, which targets the BET family of BRD proteins (which includes BRD2, BRD3, and BRD4), effectively inhibited the growth of both the melanoma cell lines even at the lower concentrations (Figure 1B). TP-472 (targeting BRD7/9), GSK-J4 (targeting KDM6A/B), GSK343 (targeting EZH2), UNC1999 (targeting EZH2/1), NVS-CERCR-1 (targeting CECR2), OF1 (targeting BRPF1/2/3; BRPF1B), and UNCO642 (targeting EHMT2) effectively inhibited the growth of both the BRAF mutant melanoma cell lines at 5 and 10 µM concentrations (Figure 1C–I). Thus, this screen identified eight out of the 32 small molecule inhibitors that effectively inhibited the survival of both BRAF mutant (M14 and SKMEL-28) melanoma cell lines.

### 3.2. Several Epigenetic Regulators Are Overexpressed in Samples from Melanoma Patients

**Figure 2:**
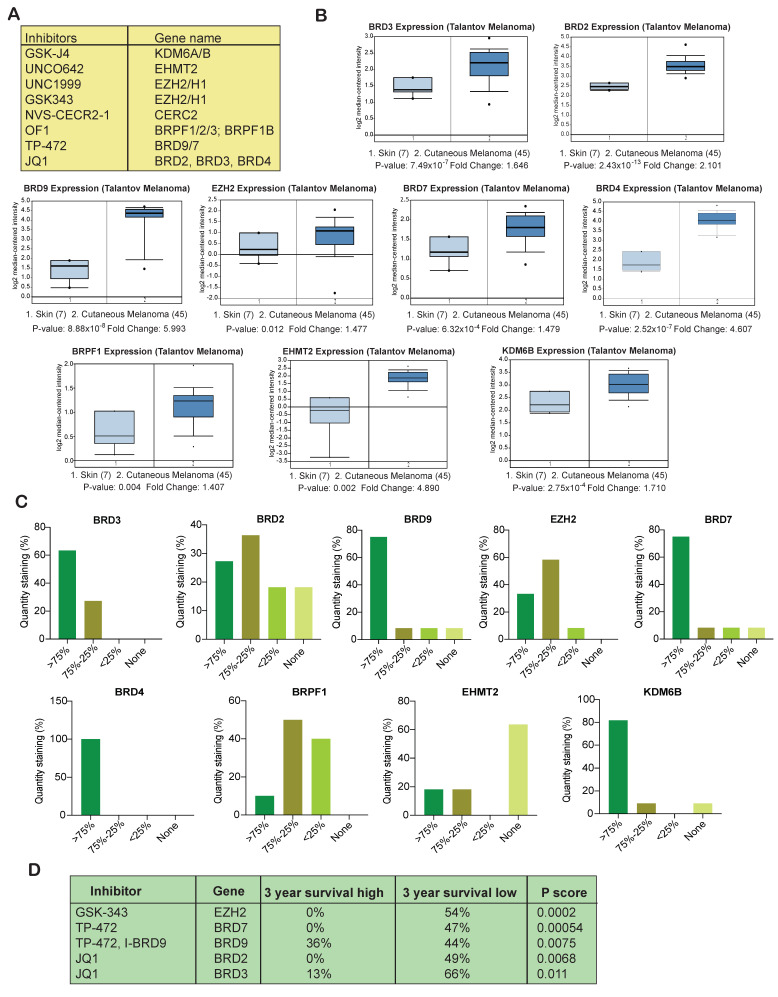
Overexpression of BRD7/9, BRD2/3, and EZH2 in melanoma samples is correlated with poor prognosis. (**A**). List of compounds that inhibited melanoma cell growth and their epigenetic targets. (**B**). Indicated patient melanoma datasets were analyzed for the shown epigenetic regulators using Oncomine. Gene upregulation in patient’s melanoma samples relative to expression in normal skin using Oncomine-Talantov melanoma patient dataset is shown. (**C**). Immunohistochemical analysis of the expression of indicated epigenetic regulators using the Human Protein Atlas dataset. Relative quantity staining for each of the epigenetic regulators are shown. (**D**). Survival analysis (three year) of melanoma patients based on the high and low expression of the epigenetic regulators using Human Protein Atlas datasets is shown.

After identifying the inhibitors that effectively inhibited the survival of melanoma cell lines, we next asked whether the target epigenetic regulators of these inhibitors are overexpressed in melanoma patient samples. To know that we evaluated the expression of the target epigenetic regulators in melanoma patient samples as compared to normal skin samples. Our analysis of publicly available Talantov melanoma datasets revealed that only EZH2, BRD7/9, BRD2/3/4, BRPF1, EHMT2, and KDM6B were significantly overexpressed at the mRNA level in patient melanoma tissue samples as compared to the normal skin samples (Figure 2A,B). We further investigated whether the proteins levels were also elevated and consistent with the mRNA levels for these epigenetic regulators in the Human Protein Atlas dataset. The Human Protein Atlas (HPA) contains gene expression data that includes quantitative transcriptomics data (RNA-Seq) and spatial proteomics data (immunohistochemistry on tissue microarrays). The results of immunohistochemistry analyses of melanoma tissues from the Human Protein Atlas indicated that BRD7, BRD9, BRD4, BRD3, and KDM6B were mostly expressed at >75% level. BRD2 and EZH2 were expressed between >75% and 75%–25% level, whereas BRPF1 was expressed at 75%–25% and <25% level in patient samples. EHMT2 protein expression level was found to be low in-patient samples (Figure 2C). When analyzed for the intensity, expression of EZH2 and BRD4 was found to be strong, and expression of both BRD7 and BRD9 was found to be moderate to strong. KDM6B, BRD2, and BRD3 were moderately to weakly expressed in melanoma samples, whereas weak staining intensity was observed for BRPF1 and EHMT2 in melanoma samples as compared with normal skin tissue indicating that they are significantly expressed at low levels (Figure S1) in melanoma samples. These results further confirmed that many of the targeted epigenetic regulators that significantly inhibited melanoma cell survival are overexpressed in melanoma patient samples and thus can serve as an excellent target for effective melanoma therapy.

### 3.3. Overexpression of Several Epigenetic Regulators Is Associated with Poor Melanoma Prognosis

After confirming that many of the target epigenetic regulators are overexpressed in melanoma patient samples, we next investigated the relationship between the expression of these regulators and melanoma patient survival. Our goal was to identify candidate epigenetic regulators whose overexpression is a predictor of poor prognosis. Examinations of Human Protein Atlas datasets revealed that overexpression of BET family BRD proteins (i.e., BRD2 and BRD3), BRD9, BRD7, and EZH2 were associated with relatively poor three-year survival in melanoma patients. Moreover, the overexpression of EZH2, BRD7, and BRD2 was associated with significantly lowest three-year survival (0%) (Figure 2D). These analyses thus indicated that monitoring the expression of BET family BRD proteins (BRD2 and BRD3), BRD9, BRD7, and EZH2 can be used to distinguish high- and low-risk patients and serve as an independent prognostic factor. These results further suggested that targeting BET family BRD proteins (BRD2 and BRD3), BRD7/9 and EZH2 could have a significant positive impact on melanoma patient survival.

### 3.4. Targeting EZH2, BRD9, and BRD7 Results in Long-Term Inhibition of Melanoma Cell Growth

**Figure 3:**
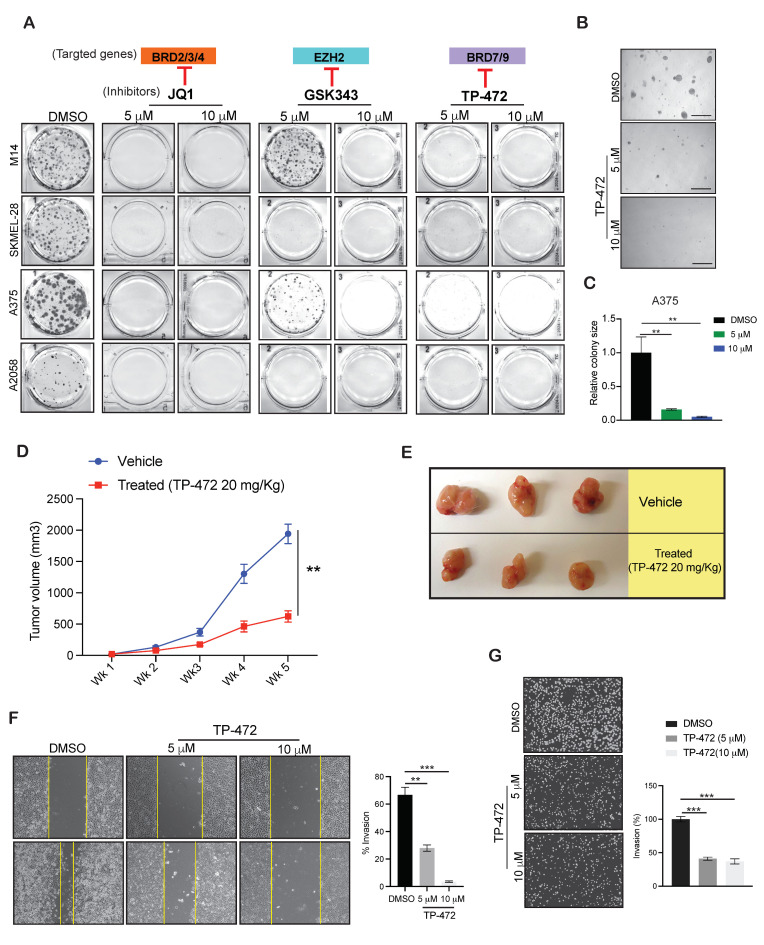
TP-472 inhibit melanoma growth in long-term survival assays, tumor growth in in vivo xenograft melanoma mouse model and invasion and migration in in vitro cell culture model. (**A**). Indicated melanoma cell lines were treated with the different concentrations of GSK343, JQ1, and TP-472 for 2 weeks. Cell survival was then measured using clonogenic assays. Representative images are shown. (**B**,**C**). A375 cells were treated with various concentrations of TP-472 and analyzed for ability to grow in an anchorage-independent manner in soft agar assays. Representative images of soft agar assays are shown. Scale bar, 500 μm (**B**). Bar diagram showing the relative colony size for each condition in panel B (**C**). (**D**). A375 cells were injected subcutaneously into the flanks of NSG mice (*n* = 6). The mice were treated with either vehicle or TP-472 (20 mg/Kg body weight) via intraperitoneal injection three times a week. The average tumor volume for each week is plotted. (**E**). Representative tumor images of NSG mice in vehicle and TP-472 treated mice at the end of the experiment. (**F**). A375 under indicated conditions were analyzed for the migration using wound-healing assay. Representative images showing the extent of migration in TP-472 treated cells relative DMSO treated cells are presented on the left and the quantification is presented as a bar diagram on the right. (**G**). A375 under indicated conditions were analyzed for invasive capacity using Matrigel invasion assay. Representative images of TP-472 treated invaded cells relative DMSO treated invaded cells are shown on the left and the quantification is presented as a bar diagram on the right. Data represent the mean ± standard error of three biological replicates. ** *p* < 0.01 and *** *p* < 0.001.

Our goal was to identify the inhibitor targeting epigenetic regulator suitable for use in treating melanoma patients in clinical settings that could positively impact melanoma patient survival. Therefore, to mimic the clinical scenario, we conducted a long-term clonogenic survival assay using GSK343 (targeting EZH2), JQ1 (targeting BET family BRD proteins-BRD2 and BRD3), TP-472 (targeting BRD7/9) using multiple BRAF mutant melanoma cell lines (M14, SKMEL-28, A375, and A2058). As shown in Figure 3A, JQ1 effectively inhibited the growth of melanoma cells at much lower concentrations as compared with the other candidates, consistent with the results obtained from the short-term survival assay using MTT. At a concentration of 10 µM, GSK343 effectively suppressed the growth of multiple melanoma cell lines. TP-472 also strongly inhibited the long-term survival of multiple melanoma cell lines at concentrations of 5 and 10 µM. A previous mouse model study demonstrated that inhibition of EZH2 using small-molecule inhibitors blocks melanoma growth and metastasis [17]. Inhibitors targeting EZH2 also induce the re-expression of tumor suppressors associated with enhanced patient survival [17]. Past studies have also revealed that JQ1 can be used to treat vemurafenib-resistant melanoma cells [18] and that BET inhibitors effectively suppress melanoma progression via the noncanonical NF-κB/SPP1 pathway [19]. However, information regarding the role of TP-472 in melanoma therapy remains limited. Therefore, we chose to investigate the role of TP-472 in melanoma and determine if TP-472 represents a new candidate drug for melanoma therapy.

### 3.5. TP-472 Inhibits Melanoma Tumor Growth In Vivo in Melanoma Xenograft Mouse Model

Based on the results described above, we next examined whether TP-472 inhibits the growth of melanoma cells in vitro using a soft agar assay, which is commonly used as a surrogate assay to measure the tumor-forming potential of cancer cells. To this end, we treated the A375 cell line with different concentrations of TP-472 and measured their ability to form colonies in soft agar. The results showed that TP-472 significantly inhibited the growth of A375 melanoma cells in a concentration-dependent manner (Figure 3B,C). Following this result, we further examined whether TP-472 inhibits the growth of melanoma cells in vivo using a mouse xenograft model of melanoma tumor growth. A375-MA2 melanoma cells were injected subcutaneously into the flanks of NSG mice, and tumor growth was monitored in mice treated with either vehicle or TP-472. We observed that the treatment with TP-472 significantly inhibited the subcutaneous tumor growth in mice (Figure 3D,E). These results confirmed that TP-472 effectively blocks melanoma tumor growth both in cell culture as well in in vivo in mice.

Additionally, we also checked whether TP-472 can inhibit metastases in melanoma cells. To do so, we first assessed whether TP-472 target BRD7/9 are overexpressed in metastatic melanoma patient samples in the Riker melanoma dataset [14] and associated with advanced disease stage (stage III) in the Xu melanoma dataset [15]. Our analysis of publicly available melanoma datasets (Riker and Xu melanoma datasets) revealed that BRD7 is significantly overexpressed in metastatic melanoma patient samples as compared to the primary site samples and is also present at higher levels in advanced-stage melanoma patient samples (Figure S2A,B). Based on this result we next tested the effect of TP-472 on cell migration using wound healing assay and invasion using matrigel invasion assay. Both these assays are simple in vitro assays to measure the ability of the cells to migrate and invade. We observed TP-472 significantly inhibited the ability of melanoma cells to invade in a matrigel invasion assay and suppressed their migratory capacity in a wound-healing assay compared to the control DMSO treated cells (Figure 3F,G and Figure S2C,D). These results confirmed that TP-472 effectively blocks melanoma metastasis.

### 3.6. Transcriptome-Wide mRNA Expression Profiling Revealed That TP-472 Treatment Leads to Downregulation of Several ECM Proteins and Upregulation of Pro-Apoptotic Genes

**Figure 4:**
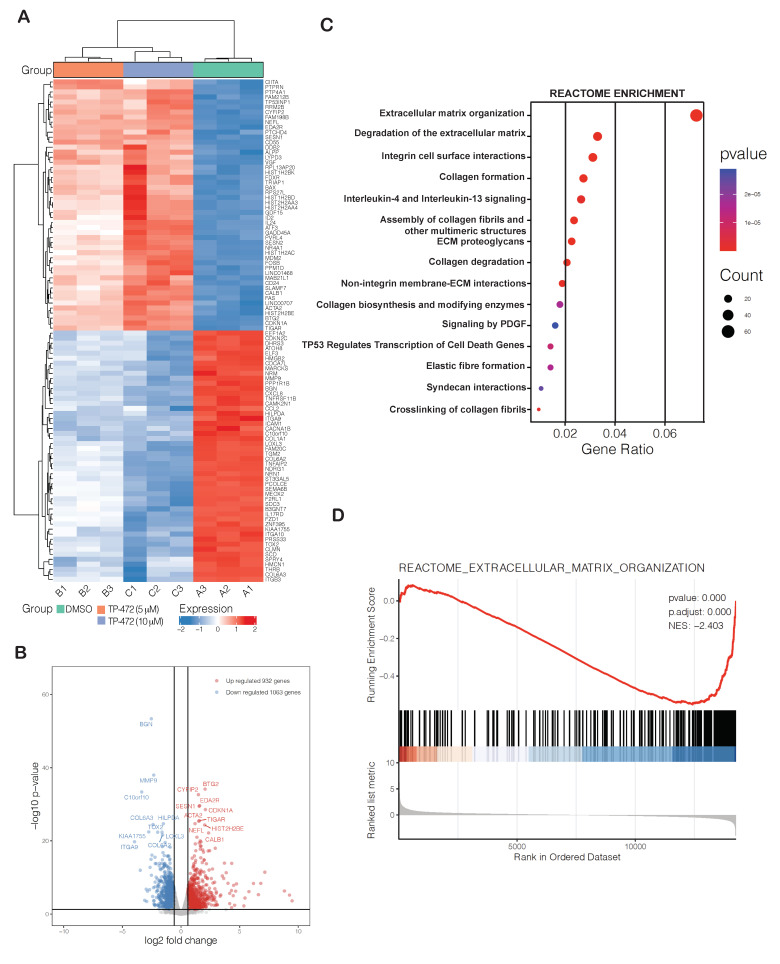
Transcriptome-wide mRNA expression profiling revealed that TP-472 treatment leads to downregulation of several extracellular matrix (ECM) proteins. A375 cells were treated with either DMSO, 5 μM TP-472, or 10 μM TP-472 for 24 h, after which RNA sequencing was performed. (**A**). Heatmap showing the top 100 genes exhibiting altered expression after 24 h of TP-472 treatment. (**B**). Volcano plot showing the top 100 genes exhibiting altered expression after treatment. (**C**). Reactome-based functional pathways analysis identified pathways that were significantly activated or suppressed in A375 cells treated with TP-472. (**D**). Reactome-based functional pathways revealed that several genes associated with ECM organization were downregulated.

**Figure 5:**
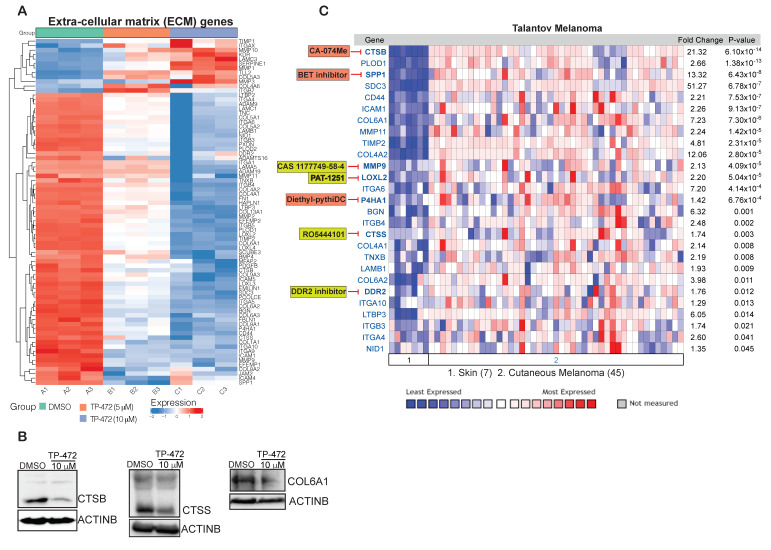
TP-472 treatment leads to downregulation of several ECM proteins in melanoma cells. A375 cells were treated with DMSO, 5 μM TP-472, or 10 μM TP-472 for 24 h, after which RNA sequencing was performed. (**A**). Heatmap showing the several ECM genes that were downregulated after 5 μM, or 10 μM of TP-472 treatment. (**B**). A375 cell line was treated with 10 μM of TP-472 for 24 h. Immunoblotting was performed to measure protein expression of the shown candidates. ACTINB were measured as loading controls. (**C**). Indicated ECM proteins were analyzed using Oncomine-Talantov melanoma patient dataset. All significantly altered ECM genes in melanoma samples versus normal skin samples are shown. Many of the ECM proteins have direct pharmacological targets and can be either targeted alone (box highlighted in yellow color) or in combination (based on the significant co-expression with BRD7) with TP-472 (box highlighted in orange color).

Finally, we examined how TP-472 inhibits melanoma growth and metastases in vitro using cell cultures as well as in vivo in mice. An unbiased, large-scale mRNA expression profiling analysis was conducted on A375 melanoma cells treated with either DMSO or with 5 µM or 10 µM TP-472 for 24 h. Treatment with TP-472 at either 5 µM or 10 µM resulted in the upregulation of 932 genes and downregulation of 1063 genes (Table S4). The degree of change in expression (both up- and downregulation) was greater in cells treated with 10 µM TP-472 than in cells treated with 5 µM TP-472, indicating that TP-472 affects mRNA expression of its target genes in a concentration-dependent manner. Our analysis revealed that many of the top 100 significantly downregulated genes encode ECM proteins (Figure 4A,B). Reactome-based functional pathway enrichment analysis revealed that downregulation of several of these ECM protein genes led to significant inhibition of various pro-oncogenic pathways, such as ECM organization, integrin cell surface interaction, collagen formation and degradation, and non-integrin membrane-ECM interactions (Figure 4C,D). Significantly downregulated ECM proteins that affected ECM-regulated pro-oncogenic pathways included collagens, fibronectins, integrins, and matrix metallopeptidase (Figure 5A). We confirmed the expression of a few downregulated ECM proteins (CCSS, CTSB, COL6A1) identified in the RNA sequencing analysis using immunoblotting (Figure 5B). Many ECM proteins, such as integrins and collagens, are known to play critical roles in supporting tumor structure and matrix rigidity and modulating the tumor microenvironment, which affects many intra- and extracellular signaling pathways [20,21]. Thus, targeting these proteins can impact immunogenicity, tumor growth, and proliferation, as well as therapy response [22,23].

To assess the clinical relevance of ECM genes that were downregulated upon TP-472 treatment, we examined whether these ECM genes are overexpressed in melanoma patient samples in a manner similar to the epigenetic regulator BRD7/9 using the Talantov melanoma dataset. We observed that the expression of several ECM genes was significantly higher in patient-derived melanoma samples compared to the normal skin samples (Figure 5C) and a few of them are also significantly co-expressed with TP-472 target epigenetic regulator BRD7 (Figure S3). Thus, many of these ECM genes can be directly targeted using specific small-molecule inhibitors either alone or in combination with TP-472, to enhance the effectiveness and durability of melanoma therapy (Figure 5C). In sum, these analyses revealed that TP-472 inhibits the expression of several ECM proteins that have been shown to regulate numerous key processes at various stages of tumorigenesis. As such, the expression profiles of ECM-related genes could serve as valuable prognostic factors for many cancers. Even more, the proteins encoded by these genes are already being targeted for effective cancer therapy [24,25].

**Figure 6:**
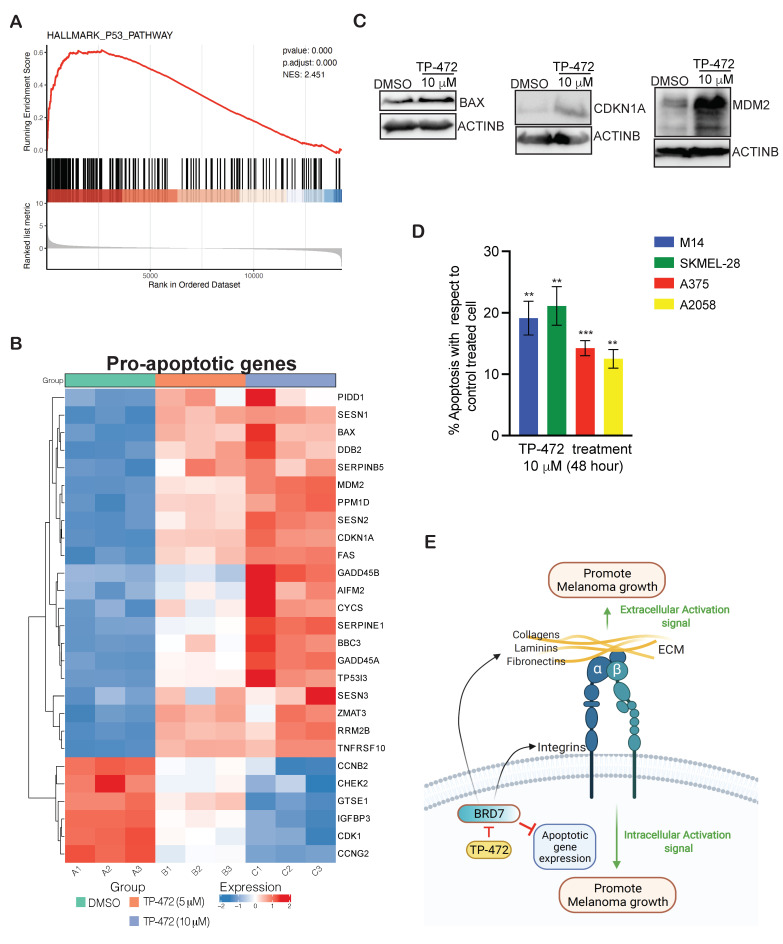
Transcriptome-wide mRNA expression profiling revealed that TP-472 treatment leads to upregulation of pro-apoptosis genes in melanoma cells. (**A**). Reactome-based functional pathways analysis revealed upregulation of several genes associated with the p53 pathway and apoptosis. (**B**). Heatmap showing the several pro-apoptosis genes that were upregulated after 5 μM, or 10 μM of TP-472 treatment. (**C**). A375 cell line was treated with 10 μM of TP-472 for 24 h. Immunoblotting was performed to measure the protein expression of the shown candidates. ACTINB were measured as loading controls. (**D**). Bar diagram showing apoptosis of melanoma cells treated with 10 μM TP-472 for 48 h (percentage apoptosis in inhibitor-treated cells relative to vehicle-treated control cells). (**E**). Model showing that TP-472 treatment of melanoma cells leads to suppression of ECM-mediated pro-oncogenic signaling pathways and activation of apoptosis genes, ultimately leading to inhibition of melanoma cell growth and proliferation. Data represent the mean ± SEM. ns = not significant, ** *p* < 0.01, *** *p* < 0.001.

Large-scale mRNA expression profiling analysis also revealed many pro-apoptotic genes associated with the p53 pathway to be upregulated in melanoma cells treated with TP-472 (Figure 6A,B). This includes BAX, GADD45B, CDKN1A, etc. The roles of many of these pro-apoptotic genes are very well established in cancer cells [26,27,28]. We further confirmed the upregulation of a few pro-apoptotic genes (BAX, MDM2, CDKN1A) in TP-472 treated cells using immunoblotting (Figure 6C). Since many of the pro-apoptotic genes were found to be upregulated, we measured apoptosis using an annexin V–based detection method in melanoma cells with and without TP-472 treatment. We observed that consistent with the upregulation of many pro-apoptotic genes, TP-472 treatment promoted apoptosis of melanoma cells (Figure 6D), thus inhibiting the growth and proliferation of melanoma tumors. However, we did not observe substantial activation of caspase 8/9 suggesting that TP-472 treatment might be activating caspase 8/9-independent apoptotic pathways in melanoma cells. Overall, our results demonstrated that the TP-472 treatment of melanoma cells results in the upregulation of pro-apoptotic genes and downregulation of ECM protein genes, ultimately leading to the inhibition of melanoma tumor growth.

## 4. Discussion

Oncogenic mutations in *BRAF* are reportedly found in approximately 50% of melanoma cases. BRAFi, either alone or in combination with MEKi, represents a therapeutic option for treating advanced melanoma with *BRAF* mutations [4,5]. However, due to the rapid emergence of acquired BRAFi resistance, the clinical benefits of these therapies are often limited [6,7]. Several mechanisms whereby targeted therapy resistance arises have been identified to date, and studies are ongoing to identify additional resistance mechanisms and develop therapies to treat drug-resistant cancers [29,30,31]. Apart from that, efforts are also taken to identify new melanoma targets for effective and durable treatment.

Several recent studies demonstrated that cancer cells can acquire numerous epigenetic alterations in addition to genetic mutations [9,32]. These epigenetic alterations of DNA and histone proteins are important in cancer initiation and progression [33,34]. Some of the most common epigenetic alterations include methylation, acetylation, ubiquitination, and phosphorylation [35]. These modifications are introduced by three specific groups of epigenetic regulators, which are known as “readers”, “writers”, and “erasers”. Writers introduce various chemical modifications of DNA and histones, whereas reader proteins contain specialized domains that identify and interpret those modifications, and erasers are a group of enzymes that effectively remove these chemical modifications. Epigenetic modifications regulate gene expression in cancer cells via effects on transcription [36]. Lately, targeting epigenetic regulators has led to the development of effective therapy for many types of cancers [9,10].

In this study, we conducted an unbiased chemical genetic screen using a library from the Structural Genome Consortium (SCG) that has 32 small molecule inhibitors, which targets proteins encoding for epigenetic regulators. This screen identified eight out of the 32 small molecule inhibitors that effectively inhibited the survival of multiple BRAF mutant melanoma cell lines. We also observed that many of the targeted epigenetic regulators that significantly suppressed melanoma cell survival are overexpressed in melanoma patient samples and thus can serve as an excellent target for effective melanoma therapy. However, to fully determine the expression level of various epigenetic regulators in different subtypes of melanoma further studies have to be performed. These study findings are summarized in Figure 6E. Using a series of in vitro and in vivo assays, we discovered that TP-472, a small molecule inhibitor of BRD7/9, strongly inhibited the growth of melanoma cells in both in vitro and in vivo assays as well as metastasis in in vitro assays. BRD7/9 belong to the BRD-containing family of epigenetic regulators, which function as readers and regulate gene transcription, DNA replication, and cell-cycle progression, thus playing an important role in tumor growth and development [37,38]. We found that the TP-472 target BRD7 is overexpressed in melanoma patient samples and associated with a poor prognosis. Other studies have shown that BRD7 promotes the growth of colorectal cancer cells via c-Myc stabilization [39]. Based on our results, we suggest that BRD7 is an important new epigenetic regulator of melanoma growth and TP-472 can be employed as a new candidate drug for the effective treatment of melanoma. Further studies using in vivo melanoma models of metastasis will reveal the role of TP-472 in melanoma metastasis.

Notably, in transcriptome-wide mRNA expression profiling experiments performed with TP-472–treated melanoma cells, we found that genes encoding several ECM proteins, such as integrins, collagens, fibronectins, and metalloproteins, were downregulated. Reactome-based functional pathway analyses revealed that many of the ECM proteins are involved in extracellular matrix interactions required for cancer cell growth and proliferation. Several ECM proteins are commonly expressed at high levels in solid cancers and contribute to matrix stiffness, which is associated with malignancy and the metastatic phenotype [40]. Further deregulation of ECM protein expression is shown to alter the tumor microenvironment, which plays a key role in regulating both intracellular and extracellular signaling [20]. Based on the important role they play in tumor growth and progression; these proteins are emerging as promising targets for cancer therapies [23]. Some of these therapies involve targeting integrins; for example, ibrutinib (pharmacologic inhibitor of integrin signaling) has been used clinically to treat lymphoid leukemia and lymphoma [41,42], whereas vitaxin, a humanized monoclonal antibody, which has specificity for the integrin alpha v beta 3 (vitronectin receptor) has been used to treat ovarian cancer [43] and other cancers, including metastatic types [44,45].

In TP-472–treated melanoma cells, several of the identified downregulated ECM proteins for example DDR1, LOXL2, CTSS, MMP9, P4HA1, CTSB, PLOD1, SPP1 have specific small-molecule inhibitors. For example, DDR1 inhibitors targeting DDR have low IC50 values and have been shown to potently attenuate tumor growth in vitro [46]. Similarly, other studies have shown that MMP9 and CTSS inhibitors effectively inhibit tumor growth as well [47]. Additionally, PAT-1251 (newly designated GB2064), which targets LOXL2, has been used in phase 1 clinical trials for the treatment of lung disease as well as metastatic breast cancer [48,49]. Studies have also shown that SPP1 is an important target of BET inhibitors, which can effectively inhibit the growth of melanoma cells [19]. A recent study identified CA-074Me as a specific cell-permeable CTSB inhibitor [50] and small-molecule inhibitor diethyl-pythiDC that targets P4HA1 has been used for treating colorectal cancer [51]. Since these ECM genes which have specific pharmacological inhibitors are significantly overexpressed in patient-derived melanoma samples compared to normal skin samples and few of them are also significantly co-expressed with TP-472 target epigenetic regulator BRD7, they can be targeted either alone or in combination with TP-472, to enhance the effectiveness and durability of melanoma therapy

In addition to several ECM proteins that were found to be downregulated we also observed upregulation of several pro-apoptotic genes such as BAX, CDKN1A, GADD45A, GADD45B, etc. in TP-472–treated melanoma cells. The role of these genes is very well established in the apoptosis and cell death processes in cancer cells and thus they serve as an essential player in tumor growth suppression [26,27,28]. However, since TP-472 treatment did not substantially activate caspase 8/9, we anticipate that induction of apoptosis in the treated melanoma cells might involve caspase 8/9-independent apoptotic pathways. Further studies will reveal the role of TP-472 in activating caspase 8/9-independent apoptotic pathways in melanoma cells.

Taken together, our results indicate that TP-472 inhibits the expression of several ECM proteins and activates the expression of several pro-apoptotic genes. Specific small-molecule inhibitors of many of these downstream ECM proteins are available and are employed in multiple studies for providing effective cancer therapy. Since they are significantly overexpressed in patient-derived melanoma samples compared to normal skin samples and are also significantly co-expressed with TP-472 target epigenetic regulator BRD7, they can be targeted either alone or in combination with TP-472 and thus serve as a new option for melanoma therapy. Our results support the need for clinical testing of TP-472 along with other inhibitors targeting the ECM proteins for treating melanoma patients in which other treatment options have not worked successfully.

## 5. Conclusions

The results of this study demonstrate that BRD7/9 small molecule inhibitor TP-472 blocks the growth of melanoma in in vitro cell culture-based assays and in vivo in mice via suppressing the expression of several ECM proteins important for melanoma growth and progression and by inducing apoptosis. Our results identify TP-472 as a candidate drug useful for melanoma therapy.

## Data Availability

The data underlying this article will be shared on reasonable request to the corresponding author.

## Supplementary Materials

The following are available online at https://www.mdpi.com/article/10.3390/cancers13215516/s1, Figure S1: Several Epigenetic regulators are overexpressed in melanoma samples, Figure S2: TP-472 inhibits invasion and migration of melanoma cells in in vitro cell culture model, Figure S3: Correlation of the mRNA expression levels of BRD7 and ECM genes performed using the Talantov melanoma dataset, Table S1: List of inhibitors targeting indicated chromatin modifiers and the concentrations at which they were used in the chemical genetic screen, Table S2: List of Reagents, data and software used in this study with source and identifier, Table S3: List of inhibitors targeting indicated chromatin modifiers that showed an effect on inhibiting M14 and SKMEL-28 melanoma cell growth, Table S4: RNA sequencing data showing significant differentially expressed gene in A375 cell line upon TP-472 treatment (5 µM and 10 µM) in comparison to control treated cells.
