# Supplementary material for: The BRD9/7 Inhibitor TP-472 Blocks Melanoma Tumor Growth by Suppressing ECM-Mediated Oncogenic Signaling and Inducing Apoptosis
Source: Cancers (Basel). 2021 Nov 3;13(21):5516. doi: 10.3390/cancers13215516 (PMC8582741; doi:10.3390/cancers13215516)
Supplement: Supplementary file 1 [file cancers-13-05516-s001.zip › cancers-1381011-supplementary.pdf]

Supplementary data

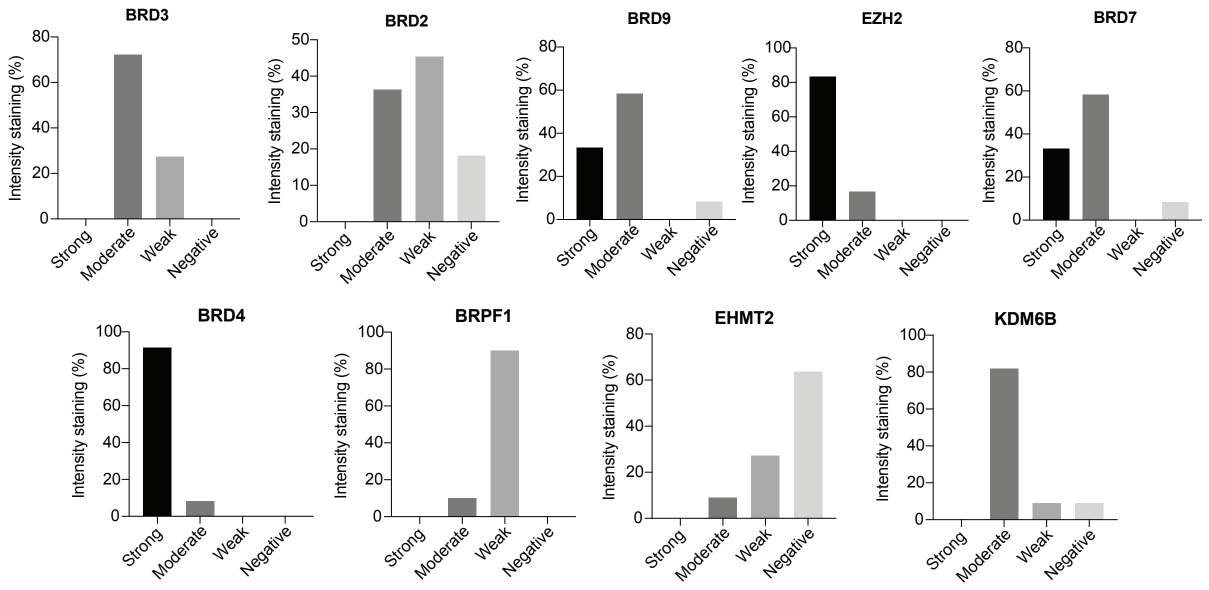

Figure S1. Several Epigenetic regulators are overexpressed in melanoma samples. Immunohistochemical analysis of the expression of indicated epigenetic regulators using the Human Protein Atlas dataset. Relative intensity staining for each of the shown chromatin modifier is plotted.

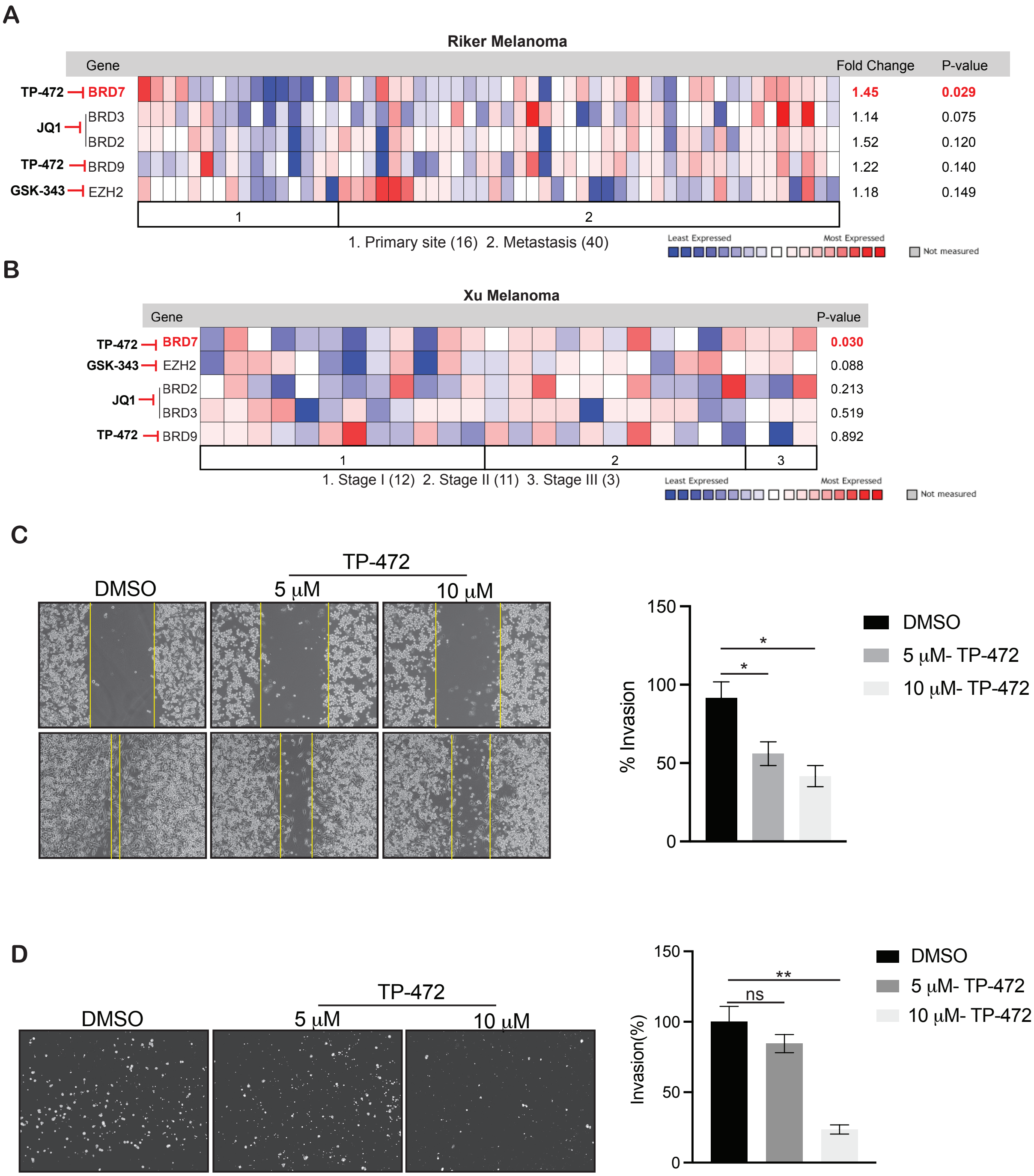

Figure S2. TP-472 inhibits invasion and migration of melanoma cells in *in vitro* cell culture model.

A-B. Indicated patient melanoma datasets were analyzed for the shown epigenetic regulators using Oncomine. Gene upregulation in patient's melanoma metastatic samples relative to the expression in primary site sample using Riker melanoma-patient dataset is shown (**A**) and gene upregulation in patient's melanoma samples at various stages using Xu melanoma patient dataset is shown (**B**). **C.** M14 under indicated condition were analyzed for the migration using wound healing assay. Representative images showing the extent of migration in TP-472 treated cells relative DMSO treated cells is presented in left and the quantification is presented as bar diagram in right. **D**. M14under indicated condition were analyzed for invasive capacity using Matrigel invasion assay. Representative images of TP-472 treated invaded cells relative DMSO treated invaded cells is shown in left and the quantification is presented as bar diagram in right. Data represent the mean ± standard error of three biological replicates. ns=non-significant, **P*<0.05, **p<0.01 and ns = not significant.

Pearson correlation coefficients to check the significance of co-expression of ECM genes along with BRD7

Gene name		Pearson r	p-value
CTSB	BRD7	<0.0001	****
PLOD1	BRD7	<0.0001	****
SPP1	BRD7	0.011	*
SDC3	BRD7	<0.0001	****
CD44	BRD7	0.1324	ns
ICAM1	BRD7	0.3224	ns
COL6A1	BRD7	0.917	ns
MMP11	BRD7	0.0344	*
TIMP2	BRD7	<0.0001	****
COL4A2	BRD7	0.0008	***
MMP9	BRD7	0.1695	ns
LOXL2	BRD7	0.3182	ns
ITGA6	BRD7	0.2462	ns
P4HA1	BRD7	0.006	**
BGN	BRD7	0.8885	ns
ITGB4	BRD7	0.9356	ns
CTSS	BRD7	0.0687	ns
COL4A1	BRD7	0.0137	*
TNXB	BRD7	0.6198	ns
LAMB1	BRD7	0.0006	***
COL6A2	BRD7	0.0475	*
DDR2	BRD7	0.7577	ns
ITGA10	BRD7	0.1539	ns
LTBP3	BRD7	0.0307	*
ITGB3	BRD7	0.3924	ns
ITGA4	BRD7	0.2561	ns
NID1	BRD7	0.5377	ns

Figure S3. Correlation of the mRNA expression levels of BRD7 and ECM genes performed using the Talantov melanoma dataset. ns, *, **, ***, and **** indicate non-significant P-value, $P<0.05$, <0.01, <0.001, and <0.0001, respectively.

SUPPLEMENTARY TABLES

Table S1: List of inhibitors targeting indicated chromatin modifiers and the concentrations at which they were used in the chemical genetic screen.

Table S2: List of inhibitors targeting indicated chromatin modifiers that showed an effect on inhibiting M14 and SKMEL-28 melanoma cell growth.

Table S3: RNA sequencing data showing significant differentially expressed gene in A375 cell line upon TP-472 treatment (5 μM and 10 μM) in comparison to control treated cells.

Table S4: List of Reagents, data and software used in this study with source and identifier.

Table S1: List of inhibitors targeting indicated chromatin modifiers and the concentrations at which they were used in the chemical genetic screen.

S.No.	Target protein	Inhibitors	Inhibitor concentrations (µm)
1	BAZ2A/2B	BAZ2-ICR	0.1, 0.5, 1, 2, 5, 10
2	BAZ2A/2B	GSK2801	0.1, 0.5, 1, 2, 5, 10
3	BET family	JQ1	0.1, 0.5, 1, 2, 5, 10
4	BRD9/7	BI-9564	0.1, 0.5, 1, 2, 5, 10
5	BRD9/7	TP-472	0.1, 0.5, 1, 2, 5, 10
6	BRD9	I-BRD9	0.1, 0.5, 1, 2, 5, 10
7	BRPF1/2/3; BRPF1B	NI-57	0.1, 0.5, 1, 2, 5, 10
8	BRPF1/2/3; BRPF1B	OF1	0.1, 0.5, 1, 2, 5, 10
9	BRPF1/2/3; BRPF1B	PFI-4	0.1, 0.5, 1, 2, 5, 10
10	CECR2	NVS-CECR2-1	0.1, 0.5, 1, 2, 5, 10
11	CREBBP, EP300	I-CBP112	0.1, 0.5, 1, 2, 5, 10
12	CREBBP, EP300	SGC-CBP30	0.1, 0.5, 1, 2, 5, 10
13	DOT1L	SGC0946	0.1, 0.5, 1, 2, 5, 10
14	EED	A-395	0.1, 0.5, 1, 2, 5, 10
15	EZH2/H1	GSK343/UNC1999	0.1, 0.5, 1, 2, 5, 10
16	G9a (EHMT2)/GLP	A-366	0.1, 0.5, 1, 2, 5, 10
17	G9a (EHMT2)/GLP	UNC0638	0.1, 0.5, 1, 2, 5, 10
18	G9a (EHMT2)/GLP	UNCO642	0.1, 0.5, 1, 2, 5, 10
19	IDH1 mutant	GSK864	0.1, 0.5, 1, 2, 5, 10
20	JMJD3/UTX (KDM6A/B)	GSK-J4	0.1, 0.5, 1, 2, 5, 10
21	LSD1 (KDM1A)	GSK-LSD1	0.1, 0.5, 1, 2, 5, 10
22	PAD4 (PADI4)	GSK484	0.1, 0.5, 1, 2, 5, 10
23	PRMT Type I	MS023	0.1, 0.5, 1, 2, 5, 10
24	PRMT3	SGC707	0.1, 0.5, 1, 2, 5, 10
25	PRMT4	TP-064	0.1, 0.5, 1, 2, 5, 10
26	PRMT4/6	MS049	0.1, 0.5, 1, 2, 5, 10
27	PRMT5	GSK591	0.1, 0.5, 1, 2, 5, 10
28	SETD7	(R)-PFI-2	0.1, 0.5, 1, 2, 5, 10
29	SMARCA2/4, PB1	PFI-3	0.1, 0.5, 1, 2, 5, 10
30	SMYD2	BAY-598	0.1, 0.5, 1, 2, 5, 10
31	SUV420H1/H2 (KMT5B/C)	A-196	0.1, 0.5, 1, 2, 5, 10
32	WDR5	OICR-9429	0.1, 0.5, 1, 2, 5, 10

Table S2: List of inhibitors targeting indicated chromatin modifiers that showed an effect on inhibiting melanoma growth.

S.No.	Target protein	Inhibitors
1	BET family	JQ1
2	BRD9/7	TP-472
3	EZH2/H1	UNC1999
4	BRPF1/2/3; BRPF1B	OF1
5	CECR2	NVS-CECR2-1
6	EZH2/H1	GSK-343
7	G9a (EHMT2) GLP	UNCO642
8	JMJD3/UTX (KDM6A/B)	GSK-J4

Table S3: Significant differentially expressed genes in TP-472 treated A375 melanoma cells

Gene_symbc	NAME	Foldchange	PValue	FDR	A1	A2
ABAT	4-aminobuty	1.6237486	0.00089983	0.00735293	5.10951601	4.17641524
ABCA12	ATP-binding	4.5530574	6.28E-05	0.00088155	0.28052245	0.38897985
ABCA13	ATP-binding	1.86693014	0.00090558	0.00739143	2.54473935	2.94805782
ABCA2	ATP-binding	-1.5151398	0.00057295	0.0051781	71.5332242	73.455774
ABCA3	ATP-binding	1.72398809	2.83E-06	6.74E-05	7.05313583	8.92606395
ABCA7	ATP-binding	1.62517823	5.34E-06	0.00011489	10.659853	10.0315856
ABCB1	ATP-binding	2.13678721	3.70E-06	8.44E-05	5.71063554	6.22367762
ABCB9	ATP-binding	1.85659391	7.75E-09	4.47E-07	29.7353795	31.9577657
ABCC3	ATP-binding	-1.8436964	0.0002903	0.00299829	76.5225163	76.5471402
ABCC9	ATP-binding	2.32499915	0.02199457	0.08097282	0.38070904	0.36850723
ABCG1	ATP-binding	-1.9776014	1.35E-05	0.00025111	7.15332242	8.12763163
ABCG4	ATP-binding	2.1880745	0.01529916	0.06224044	0.62115685	0.49134297
ABI2	abl-interacto	-1.5840938	6.15E-06	0.0001297	63.9791554	65.4919234
ABI3BP	ABI family; n	-2.0764177	0.00651471	0.03322873	3.14585888	2.66144109
ABL2	ABL proto-or	1.59072019	0.00080015	0.00665822	103.292373	110.818312
ACACA	acetyl-CoA ca	-1.5502555	0.00020631	0.0023024	129.340886	130.656285
ACE	angiotensin I	-3.7734426	6.35E-06	0.00013259	3.10578424	3.68507227
ACER2	alkaline cera	4.28558252	8.35E-11	8.88E-09	1.22227638	2.39529698
ACHE	acetylcholine	2.96860112	0.0001498	0.00178941	0.98182857	1.02363119
ACRC	acidic repeat	2.55085835	0.0001216	0.00150295	2.16403031	1.57639203
ACSL5	acyl-CoA syn	-2.0136524	0.00242438	0.01569366	3.70690377	4.11499737
ACSS1	acyl-CoA syn	-1.527372	0.00214101	0.01425106	12.4832489	11.8945944
ACSS2	acyl-CoA syn	-1.5478084	5.27E-05	0.00076217	34.123552	32.5719444
ACTA2	actin; alpha 2	2.85637266	4.17E-26	5.94E-23	19.4161609	21.168693
ACTA2-AS1	ACTA2 antise	2.37431718	0.04218891	0.12815525	0.54100758	0.12283574
ACTN2	actinin; alpha	2.64021809	0.01425617	0.05899092	0.64119417	0.45039772
ACTR3B	ARP3 actin-r	1.51506382	0.00109127	0.00849357	11.8821294	10.5638739
ADAM19	ADAM metal	-1.6477481	0.02219108	0.08133875	329.413503	338.678615
ADAM9	ADAM metal	-1.5395524	0.00014626	0.00175301	193.119668	197.970272
ADAMTS10	ADAM metal	-4.2767586	8.31E-07	2.44E-05	2.32432885	2.57955059
ADAMTS16	ADAM metal	-2.6246718	0.00623446	0.03215203	2.42451544	2.57955059
ADAMTSL3	ADAMTS-like	-1.7589491	0.00449312	0.02506958	4.40820989	4.13547
ADAMTSL4	ADAMTS-like	-2.1619234	2.88E-06	6.83E-05	49.512212	50.6492711
ADAP1	ArfGAP with	-1.7750159	0.0090968	0.04248471	2.92544838	3.17325668
ADCY1	adenylate cy	1.68483717	0.01840696	0.0709926	1.56291078	1.86300876
ADGRA2	adhesion G p	-2.110726	8.66E-07	2.50E-05	17.8933247	20.841131
ADGRB2	adhesion G p	-1.5857411	4.99E-06	0.00010816	28.5331404	29.5624687
ADGRE2	adhesion G p	1.50659554	0.01825118	0.07050482	3.78705305	4.77012133
ADGRF1	adhesion G p	-1.6934818	0.00038198	0.00374647	55.7037432	60.1485686
ADGRG1	adhesion G p	-1.6549166	4.29E-06	9.59E-05	249.705053	259.019636

ADGRL3	adhesion G p	-2.3258266	0.00049076	0.00458311	10.279144	9.47882479
ADI1	acireductone	1.70147032	9.75E-08	3.95E-06	81.1310994	81.7881319
ADM	adrenomedu	-1.6314695	9.41E-06	0.00018514	22.7824302	22.8269755
ADORA1	adenosine A:	-1.8813291	8.21E-06	0.00016401	21.2996687	20.7797131
ADPRHL1	ADP-ribosylh	1.75670909	0.00340037	0.02029258	3.54660523	3.05042094
ADRB2	adrenocepto	1.72675287	0.00365597	0.021432	6.87279997	6.22367762
AEBP1	AE binding p	-1.7112851	3.00E-08	1.44E-06	192.618735	199.853753
AEN	apoptosis en	1.8801372	1.31E-10	1.29E-08	84.2368836	85.20706
AFAP1	actin filamer	-2.5021962	1.10E-09	8.46E-08	311.079357	325.432827
AFAP1L2	actin filamer	-2.2616435	2.36E-05	0.00039773	9.59787518	9.92922252
AGAP2	ArfGAP with	-1.8864438	3.98E-05	0.00061327	9.69806177	10.3591476
AGMO	alkylglycerol	-2.1345504	3.84E-07	1.28E-05	25.7078786	28.293166
AIFM2	apoptosis-in	1.60585729	0.00032191	0.00324641	26.6496325	23.8096614
AIM1	absent in me	-2.3106723	2.15E-06	5.37E-05	89.8272952	88.9126049
AIMP2	aminoacyl tR	1.68573244	6.57E-05	0.00090938	26.4091847	24.2805318
AJAP1	adherens jun	1.94633342	1.52E-09	1.14E-07	20.3378775	21.8033443
AK1	adenylate kir	1.50989216	3.26E-05	0.00051667	39.5536651	39.6554722
AK4	adenylate kir	-1.7583517	6.22E-06	0.00013094	69.2088953	72.9439584
AK5	adenylate kir	-1.6785495	0.0108358	0.04822642	5.75071018	4.89295707
AKNA	AT-hook tran	-1.7236633	0.00030623	0.00312558	8.25537489	6.9811647
AKR1C1	aldo-keto rec	-1.51309	0.02765961	0.095097	5.8909714	7.39061717
AKR1C2	aldo-keto rec	-1.8012715	0.00030231	0.00309498	8.31548685	8.16857687
AKR1C3	aldo-keto rec	-1.5552393	3.68E-06	8.42E-05	26.6496325	26.7781919
ALDOC	aldolase C; fr	-1.8800363	1.93E-08	9.91E-07	103.492746	102.813516
ALG10B	ALG10B; alpl	-1.5804037	0.00092689	0.00751798	18.4543696	17.7702374
ALOXE3	arachidonate	2.31674824	0.04116863	0.1258701	1.0219032	0.67559658
ALPK2	alpha-kinase	-1.8564143	1.50E-06	3.96E-05	38.8924337	38.5908958
ALPL	alkaline phos	2.72543482	0.00551577	0.02934124	0.80149271	0.75748708
ALPP	alkaline phos	3.12333515	9.62E-21	6.53E-18	8.07503903	9.90874989
ALPPL2	alkaline phos	3.76346132	0.00777852	0.03813269	0.32059708	0.20472624
AMACR	alpha-methy	-1.5551456	0.00052463	0.00483157	65.0411333	65.7375948
AMDHD1	amidohydrol	2.23176883	0.02868261	0.09748531	0.46085831	0.36850723
AMOT	angiomotin	-1.7269752	2.95E-05	0.00047775	29.2544838	33.5751029
AMOTL1	angiomotin l	-1.5858565	0.00019625	0.0022126	260.585317	278.468628
AMZ2P1	archaelysin f	2.22959591	4.39E-05	0.00065917	4.96925479	4.09452475
ANG	angiogenin; r	-2.4839003	0.00043625	0.00415849	2.92544838	3.05042094
ANGPTL4	angiopoietin-	-1.8831345	5.13E-09	3.18E-07	53.4595636	53.1264586
ANK1	ankyrin 1; ery	2.02818666	0.00851898	0.04050849	1.00186589	1.5559194
ANK2	ankyrin 2; ne	-1.7640133	0.0020538	0.01380598	8.0349644	7.71817915
ANKDD1A	ankyrin repea	-1.7389557	0.03439581	0.11079897	2.38444081	2.43624223
ANKH	ANKH inorga	-2.184918	3.40E-08	1.61E-06	186.226831	187.304035
ANKRA2	ankyrin repea	1.5799306	2.43E-05	0.00040555	15.8094437	17.1970039
ANKRD1	ankyrin repea	2.32185803	1.72E-07	6.45E-06	11.160786	11.3418336

ANKRD20A1:	ankyrin repea	2.49460203	4.19E-09	2.65E-07	4.1877994	3.2756198
ANKRD24	ankyrin repea	2.27616688	0.00071126	0.00608047	1.28238833	1.00315856
ANKRD30A	ankyrin repea	-1.6569614	0.01117355	0.04940442	6.63235216	7.28825405
ANKRD36C	ankyrin repea	-1.6460277	0.00140532	0.01041998	6.67242679	6.91974683
ANLN	anillin actin l	-1.8464832	5.18E-05	0.00075249	239.145387	250.503024
ANO1	anoctamin 1,	-2.1127478	1.15E-05	0.00021815	51.6962797	54.5390697
ANO6	anoctamin 6	-1.5687276	0.00013821	0.00167482	116.777488	120.849898
ANXA10	annexin A10	2.32163272	0.0003701	0.00364247	1.60298542	1.49450153
AOAH	acyloxyacyl h	-3.6870006	0.00432055	0.02430802	1.08201516	1.65828252
AOC2	amine oxidas	3.14622825	0.000106	0.00134872	1.10205247	0.73701445
AOC3	amine oxidas	5.07424963	2.26E-05	0.00038584	0.26048513	0.30708936
AOX1	aldehyde oxi	1.70357386	0.00405926	0.02314867	2.84529911	2.72285896
AP1S3	adaptor-rela	1.67593729	7.30E-05	0.00099037	5.97112067	6.42840386
AP5Z1	adaptor-rela	1.6815938	4.92E-05	0.00072398	22.5019078	19.694664
APLP1	amyloid beta	1.88721269	1.58E-10	1.55E-08	11.0405621	12.3654647
APOBEC3B	apolipoprote	-2.0746863	7.28E-08	3.07E-06	22.3215719	22.1923241
APOBEC3D	apolipoprote	-2.0148449	7.83E-09	4.50E-07	27.3309013	26.2868489
APOBEC3F	apolipoprote	-1.8429327	2.35E-07	8.25E-06	46.7470622	44.0980315
APOBEC3G	apolipoprote	-2.5239471	4.37E-08	1.97E-06	204.300491	201.593926
APOBEC3H	apolipoprote	2.23572689	0.00159658	0.01140012	1.16216443	0.75748708
APOL1	apolipoprote	-1.6121436	0.01371334	0.05752981	6.09134458	6.14178712
AQP3	aquaporin 3 (	2.20632001	5.72E-11	6.37E-09	9.0568676	9.02842707
ARC	activity-regul	14.4785164	4.99E-12	6.78E-10	1.64306005	1.00315856
AREG	amphiregulin	2.88276781	1.52E-07	5.78E-06	18.9953772	20.4316785
ARHGAP11A	Rho GTPase	-1.6066772	0.00063837	0.0055997	86.9218842	96.3032221
ARHGAP18	Rho GTPase	-1.5946003	5.39E-07	1.69E-05	65.2214691	66.1675199
ARHGAP24	Rho GTPase	-1.5046564	0.0043337	0.02436274	11.1808233	9.94969514
ARHGAP26	Rho GTPase	-1.5478236	0.00018242	0.00207976	38.6720232	39.3483828
ARHGAP28	Rho GTPase	-2.0070147	0.00604956	0.03142993	2.9454857	3.33703767
ARHGAP33	Rho GTPase	-1.8145289	0.00901055	0.04218081	2.18406763	2.70238633
ARHGEF16	Rho guanine	-2.4813923	7.30E-05	0.00099037	3.06570961	3.48034604
ARHGEF17	Rho guanine	-1.5983172	0.0005751	0.00518547	15.7292944	16.3576264
ARHGEF39	Rho guanine	-1.5770842	0.00218199	0.01446975	14.066197	15.1702142
ARHGEF40	Rho guanine	-1.9619523	1.90E-07	6.93E-06	53.4395263	54.2933982
ARID3B	AT rich intera	1.81968563	1.89E-06	4.84E-05	10.1188454	8.76228296
ARL14	ADP-ribosyla	2.55748902	0.00063342	0.00557904	0.5610449	0.96221332
ARL14EPL	ADP-ribosyla	1.76632864	0.00780143	0.03819242	1.5829481	1.80159089
ARL6IP1	ADP-ribosyla	-1.5611239	2.37E-08	1.19E-06	213.858292	204.501039
ARL6IP6	ADP-ribosyla	-1.7813211	1.11E-08	6.11E-07	22.5419824	22.4584682
ARNTL	aryl hydrocar	1.78459802	2.46E-06	5.95E-05	9.53776323	9.72449628
ARPIN	actin-related	-1.6272132	0.0013751	0.01024386	34.8248582	39.0208209
ARRDC3	arrestin dom	-1.5504446	8.53E-07	2.48E-05	52.4176231	55.0713579
ARRDC4	arrestin dom	2.18589823	0.00014302	0.00172	3.00559766	3.05042094

ARSG	arylsulfatase	1.75282789	0.03959841	0.1225444	1.16216443	1.00315856
ARSJ	arylsulfatase	-1.55515	0.00080958	0.0067195	27.0103043	25.0175462
ART3	ADP-ribosyltı	-4.646219	3.48E-08	1.64E-06	4.92918015	5.32288217
ARVCF	armadillo re|	1.93179617	0.00031001	0.00315573	3.18593351	2.9275852
ASAP3	ArfGAP with	-2.4248767	1.28E-06	3.46E-05	7.63421804	6.59218485
ASCC3	activating sig	1.68808409	6.42E-06	0.00013387	68.9283729	76.3628866
ASF1B	anti-silencing	-1.5642451	3.25E-05	0.00051667	63.7988196	60.1076233
ASIC1	acid sensing	-1.5565686	0.00020993	0.00233364	22.5219451	21.4348371
ASMTL-AS1	ASMTL antis	1.99985157	0.03058674	0.10182005	0.30055977	0.65512396
ASNS	asparagine s	1.68444766	0.000573	0.0051781	34.0434028	34.0664459
ASPH	aspartate be	-1.572622	7.03E-05	0.00096173	270.163155	268.826022
ASPM	abnormal spi	-2.2183297	1.95E-05	0.00034109	167.471901	184.355977
ASS1	argininosucci	-2.9732022	0.00920154	0.04275551	1.5829481	1.10552168
ASTN2	astrotactin 2	2.60111967	1.71E-10	1.63E-08	4.34809794	4.95437495
ATAD2	ATPase fami	-1.5634702	0.00072345	0.00615149	93.5141617	102.465482
ATAD3B	ATPase fami	1.91042517	6.58E-09	3.92E-07	49.3118389	44.343703
ATF3	activating tra	20.015805	1.39E-14	3.25E-12	3.6267545	3.64412703
ATG101	autophagy re	1.64242578	0.00011567	0.0014435	73.476844	68.828961
ATG4A	autophagy re	2.25296913	2.64E-09	1.78E-07	9.09694224	9.06937232
ATOH8	atonal bHLH	-5.5611986	7.68E-17	2.81E-14	11.6216443	11.1575799
ATP10A	ATPase; clas	-1.637662	0.0058281	0.03050175	11.1808233	12.0379028
ATP5E	ATP synthase	1.58868269	0.00058354	0.00524012	101.609238	102.404064
ATP5I	ATP synthase	1.63107365	0.00189226	0.01301707	39.1529188	36.9735585
ATP6V0B	ATPase; H+ t	1.57441859	0.00010219	0.00131076	92.7327063	91.0212852
ATP6V0E2	ATPase; H+ t	1.56974256	1.89E-05	0.00033127	33.0816115	33.6365208
ATP6V1B1-A	ATP6V1B1 ar	1.99800893	0.00258944	0.01654035	1.74324664	1.78111827
ATP8B3	ATPase; ami	-2.4041725	0.00050989	0.00472459	2.78518716	2.57955059
ATP9A	ATPase; clas	-2.2306903	9.59E-08	3.90E-06	79.5080766	81.7676592
ATRNL1	attractin-like	-1.6513067	0.00890535	0.04180175	4.06757549	3.82838064
ATXN1	ataxin 1	-1.6475521	0.0007389	0.00626788	22.7223183	26.0207048
AURKAPS1	aurora kinase	2.04599589	0.00440995	0.02473285	1.5829481	1.61733728
AURKB	aurora kinase	-1.6677805	2.44E-06	5.93E-05	58.969826	58.1217788
AVPI1	arginine vasc	2.04430808	1.10E-05	0.00021078	32.0597083	35.4381117
B3GNT4	UDP-GlcNAc	-2.027521	0.03614639	0.11488005	1.04194052	1.53544678
B3GNT5	UDP-GlcNAc	-1.6088486	0.00281077	0.01756854	19.4762728	22.1718515
B3GNT7	UDP-GlcNAc	-3.4296944	4.46E-13	7.47E-11	79.027181	73.3329383
B4GALNT3	beta-1;4-N-a	-2.7603003	1.10E-05	0.00021078	3.22600815	2.98900307
B4GALNT4	beta-1;4-N-a	-2.2936465	3.56E-09	2.34E-07	51.2955333	49.9327293
BAGE4	B melanoma	23.8290413	0.00383049	0.02217237	0.08014927	0.14330837
BAHCC1	BAH domain	-1.6582148	0.00104833	0.00825859	41.2768745	42.275968
BAK1	BCL2-antago	1.56117294	6.89E-05	0.00094633	22.3616466	22.0080705
BARD1	BRCA1 assoc	-1.6327645	0.00071443	0.00609298	8.13515099	8.27093999
BARX1	BARX homec	-1.9556693	9.53E-05	0.00123487	13.9860478	14.3513092

BAX	BCL2-associa	2.38532272	2.03E-15	5.37E-13	110.18521	101.503269
BBC3	BCL2 binding	2.70479215	1.02E-10	1.06E-08	12.7637714	13.1843697
BCAM	basal cell adl	-1.9538236	1.73E-09	1.29E-07	61.7750505	58.6131218
BCL3	B-cell CLL/lyr	-1.5617209	2.50E-05	0.00041549	32.7009025	35.908982
BCRP2	breakpoint cl	-2.5917594	0.04036942	0.12426897	1.84343323	1.74017302
BCYRN1	brain cytopla	4.30449553	3.27E-07	1.11E-05	1.32246297	1.10552168
BEND5	BEN domain	-2.685257	0.00179231	0.01251458	2.20410495	1.96537188
BEST3	bestrophin 3	2.46186448	0.00314938	0.01922136	0.64119417	0.63465134
BGN	biglycan	-5.767678	4.48E-54	6.39E-50	90.6688626	83.7535037
BHLHE41	basic helix-lc	-1.5983898	0.00012574	0.0015408	11.0205247	11.7308134
BIN1	bridging inte	-1.5039443	0.00149305	0.01086693	12.363025	10.7481275
BISPR	BST2 interfe	-2.113086	0.00296953	0.01833569	2.62488862	2.10868025
BLOC1S2	biogenesis o	2.184692	3.78E-10	3.31E-08	30.5168349	33.5955756
BMF	Bcl2 modifyi	-1.9262084	0.00012436	0.00152776	8.17522562	7.82054227
BMP4	bone morphc	-2.0258571	0.00103715	0.00818862	11.922204	11.7922313
BMP8B	bone morphc	-1.8505133	0.0002554	0.0027223	16.8313469	16.9718051
BMS1P20	BMS1 riboso	1.62437457	0.0012468	0.00949157	4.30802331	4.0535795
BMX	BMX non-rec	-7.2208068	5.67E-07	1.75E-05	4.62862039	4.4016141
BNIP3	BCL2/adenov	-1.715566	2.74E-08	1.35E-06	218.12624	232.732787
BNIP3L	BCL2/adenov	-1.665528	2.67E-09	1.78E-07	116.23648	111.473436
BOC	BOC cell adh	-2.081402	0.00097885	0.00782927	3.86720232	4.60634034
BOLA2	bolA family r	1.70731096	0.00095046	0.00764455	22.9627661	23.0521743
BOLA2B	bolA family r	1.70731096	0.00095049	0.00764455	22.9627661	23.0521743
BRCAT54	NA	-2.0471513	0.00569324	0.02993391	2.36440349	2.45671485
BRF2	BRF2; RNA p	1.56319218	0.00522249	0.02816025	7.27354633	7.37014455
BRINP2	bone morphc	1.778175	0.00140833	0.01042993	2.2642169	2.94805782
BRIP1	BRCA1 intera	-1.5565415	0.0017975	0.01254462	22.0210122	23.7482435
BRMS1	breast cance	1.65208608	0.00016603	0.00194263	39.6939264	37.2397026
BSCL2	Berardinelli-!	2.30799127	0.00150552	0.01093538	15.9897795	15.0269058
BSN	bassoon pres	1.59549525	0.02648802	0.09233755	1.88350786	1.9858445
BST2	bone marrow	-3.1103587	1.01E-06	2.85E-05	5.99115799	5.3433548
BTBD3	BTB (POZ) d	-1.8095883	1.91E-08	9.84E-07	114.633495	116.857736
BTG2	BTG family;	4.15699849	6.96E-35	3.31E-31	21.0592209	20.3702606
BTNL9	butyrophilin-	2.97407449	0.00236314	0.0153777	0.50093294	0.26614411
BUB1	BUB1 mitoti	-1.5061788	0.00012299	0.00151624	60.9535204	61.4178712
BUB1B	BUB1 mitoti	-1.6843438	0.00014085	0.00170045	89.6469594	90.7346684
C10orf10	chromosome	-10.17062	4.16E-34	1.48E-30	7.37373291	8.107159
C10orf35	chromosome	2.3489796	0.0001155	0.00144264	1.52283615	1.92442663
C11orf91	chromosome	3.08187792	6.41E-05	0.00089038	0.96179125	1.06457643
C12orf45	chromosome	1.64137915	0.00941658	0.04342981	8.17522562	7.86148752
C12orf60	chromosome	1.91023502	0.00719272	0.03567819	1.72320932	1.35119317
C14orf132	chromosome	-1.9909296	0.00019079	0.00215792	4.16776208	5.07721069
C15orf48	chromosome	-2.0601057	4.11E-05	0.00062845	10.0988081	9.13079019

C16orf54	chromosome	-2.237783	0.0118564	0.0515643	1.48276151	2.4157696
C17orf51	chromosome	1.63150287	1.44E-05	0.00026367	15.8094437	16.4190442
C17orf89	chromosome	1.7470372	6.57E-06	0.00013665	32.7209398	27.5356789
C17orf96	chromosome	-1.5146429	0.0003562	0.00353852	29.4147824	30.0742843
C17orf97	chromosome	1.57308536	0.0319065	0.10503498	2.18406763	2.4976601
C18orf32	chromosome	1.68537071	0.00057732	0.00519407	18.8951906	14.6583986
C19orf33	chromosome	1.72830076	0.00079016	0.00660061	15.2884734	12.6725541
C19orf71	chromosome	-1.6622482	0.03219537	0.10557206	3.38630669	2.66144109
C1QL1	complement	-2.0920715	0.00105816	0.00830602	3.92731427	4.54492247
C1QL4	complement	-2.368333	0.00036622	0.00361178	2.86533643	2.76380421
C1QTNF1	C1q and tum	-2.0004034	6.63E-06	0.00013769	37.8905678	37.2806478
C1orf106	chromosome	-1.7303702	8.16E-07	2.41E-05	54.7018773	53.5359111
C1orf116	chromosome	2.60328119	0.00011916	0.00148054	0.88164198	1.53544678
C1orf21	chromosome	-1.6914097	1.05E-05	0.00020263	20.3779521	20.5545142
C20orf24	chromosome	1.61518889	1.82E-05	0.00032201	91.7108031	92.986657
C21orf58	chromosome	-1.848178	3.45E-06	7.96E-05	10.9203381	11.1371073
C22orf34	chromosome	1.59803673	0.02157287	0.07985323	2.30429154	1.94489926
C2orf72	chromosome	-3.0168053	0.0003828	0.00375195	3.40634401	2.19057074
C3	complement	-2.5744197	1.15E-10	1.16E-08	190.675115	186.710329
C3orf52	chromosome	1.98793672	0.00032562	0.00327254	3.52656792	3.1937293
C6orf141	chromosome	-2.3004903	4.50E-10	3.84E-08	13.6053387	13.6757127
C6orf52	chromosome	3.00374104	0.02293787	0.08313061	0.36067172	0.49134297
C7orf43	chromosome	1.64461574	0.00011081	0.00139872	25.1267964	23.8710793
C8orf37	chromosome	-1.7001752	0.03943519	0.12211883	4.66869502	4.72917608
C8orf48	chromosome	1.80679355	0.01330907	0.05623242	1.26235102	1.2897753
C9orf156	chromosome	1.57055927	0.0045405	0.02522674	5.28985187	7.28825405
C9orf43	chromosome	1.73045745	0.04617023	0.13650872	0.76141807	0.8598502
CA5B	carbonic anh	-1.5748632	0.00016464	0.00192788	15.0480256	14.310364
CA9	carbonic anh	-3.8055935	2.53E-08	1.25E-06	79.1674422	81.1330079
CAB39L	calcium bind	1.64387806	0.00113268	0.00874895	4.82899357	4.38114148
CACNA1A	calcium char	-1.8118723	0.01362421	0.05722328	3.20597083	2.8456947
CACNA1B	calcium char	-11.096236	1.05E-12	1.61E-10	2.14399299	2.8456947
CACNA2D2	calcium char	2.36505186	0.00340021	0.02029258	0.64119417	0.59370609
CACNG6	calcium char	-1.7619344	0.00132876	0.00996643	5.69059823	6.75596584
CALB1	calbindin 1; 2	5.11679527	6.68E-23	5.29E-20	5.91100872	7.24730881
CALB2	calbindin 2	1.79738656	4.30E-05	0.00064895	13.184555	14.5150902
CALHM2	calcium hom	-1.7504363	4.22E-07	1.38E-05	47.0676593	45.3878068
CAMK1D	calcium/caln	-5.0796345	6.13E-08	2.64E-06	2.34436617	1.92442663
CAMK2D	calcium/caln	-1.5951326	2.33E-06	5.71E-05	52.4176231	53.3926027
CAMK2N1	calcium/caln	-2.1422258	1.42E-16	4.70E-14	65.7224021	67.1297333
CAMKK1	calcium/caln	-1.605701	0.00941675	0.04342981	7.27354633	5.17957381
CARD9	caspase recr	1.81897698	0.0330662	0.10770889	1.30242565	0.88032282
CASC5	cancer susce	-1.6116004	0.00609901	0.03162272	50.0331823	52.3484989

CASZ1	castor zinc fi	1.66111021	0.02312154	0.0835671	2.62488862	2.33387911
CAT	catalase	-1.5363851	2.38E-05	0.00039869	106.939165	102.629263
CAV1	caveolin 1; ca	-1.7333394	3.13E-07	1.07E-05	441.86293	437.868477
CBR3	carbonyl redu	-1.6485113	0.01108773	0.04914811	4.28798599	3.60318178
CBS	cystathionine	-1.5249959	0.00058074	0.00522151	17.9935113	16.4395169
CBWD3	COBW doma	1.51557713	0.00687403	0.03457396	9.0568676	11.587505
CBX2	chromobox h	-1.6221745	3.84E-05	0.00059553	27.8318343	26.1640131
CBX5	chromobox h	-1.5724815	6.77E-07	2.05E-05	170.136865	168.448748
CBX7	chromobox h	-1.5215608	0.00046933	0.00442066	17.1920186	16.214318
CCBE1	collagen and	-1.6187144	1.85E-05	0.00032703	14.9678763	15.5182488
CCDC102B	coiled-coil do	-1.9720689	1.02E-07	4.11E-06	32.6608279	34.3530626
CCDC144CP	coiled-coil do	6.22889894	2.38E-05	0.00039869	0.06011195	0.12283574
CCDC159	coiled-coil do	1.65924471	0.02743947	0.0947057	1.46272419	1.31024792
CCDC181	coiled-coil do	1.59906663	0.00407617	0.02322221	6.39190435	5.81422514
CCDC64	coiled-coil do	6.14428281	1.52E-06	4.00E-05	0.26048513	0.24567148
CCDC84	coiled-coil do	1.56956162	0.00069533	0.00597654	7.37373291	7.3291993
CCDC85A	coiled-coil do	1.83484965	0.04182726	0.12728598	1.0219032	1.31024792
CCL2	chemokine ((	-3.0794202	1.45E-14	3.34E-12	26.9101177	29.2553793
CCL28	chemokine ((	-1.7443674	4.44E-05	0.00066442	12.0624653	11.1985252
CCL3	chemokine ((	141.87235	3.53E-12	4.98E-10	0.02003732	0
CCNA1	cyclin A1	1.92904839	6.88E-09	4.05E-07	8.09507635	8.70086509
CCNA2	cyclin A2	-1.7819347	2.05E-06	5.20E-05	72.0141198	70.4667709
CCNB2	cyclin B2	-1.7668823	1.02E-06	2.88E-05	64.8407601	68.3376181
CCNF	cyclin F	-1.8375923	2.11E-07	7.57E-06	52.9385934	53.7406373
CCNG2	cyclin G2	-1.9953129	5.64E-10	4.67E-08	43.9819124	41.3956452
CCR10	chemokine ((	-2.2942315	0.00323072	0.01961468	2.42451544	1.88348138
CD163L1	CD163 molec	2.49339551	1.25E-05	0.00023573	1.50279883	2.64096846
CD1D	CD1d molecu	1.98588972	0.00043243	0.00413044	1.74324664	2.00631713
CD24	CD24 molecu	4.49271054	1.75E-14	3.83E-12	1.82339591	1.6378099
CD274	CD274 molec	1.88168017	0.00030771	0.00313446	4.44828453	4.36066886
CD44	CD44 molecu	-1.5249241	6.99E-06	0.00014421	1137.33819	1161.24816
CD55	CD55 molecu	1.93965135	5.26E-16	1.60E-13	152.103279	151.292689
CD68	CD68 molecu	1.703239	5.36E-07	1.69E-05	55.3230342	53.0445681
CD79B	CD79b molec	6.2128872	0.00017789	0.00203951	0.16029854	0.18425361
CDC25B	cell division (	-1.5993055	1.21E-06	3.32E-05	237.201767	245.978574
CDC25C	cell division (	-1.9460412	2.97E-06	7.03E-05	10.5195918	12.4883005
CDC42BPG	CDC42 bindir	1.8800932	0.00157596	0.01129073	1.72320932	3.11183881
CDC42EP4	CDC42 effect	-1.6544125	9.59E-08	3.90E-06	65.6422528	68.8699063
CDC42EP5	CDC42 effect	-7.8240783	1.57E-07	5.96E-06	1.96365713	2.47718747
CDC45	cell division (	-1.6529779	1.25E-05	0.00023484	21.8807509	19.9812808
CDCA2	cell division (	-1.8302666	6.47E-07	1.97E-05	25.7479532	28.2317481
CDCA3	cell division (	-1.6815012	0.00042768	0.00409606	25.1468337	23.7687162
CDCA7L	cell division (	-1.9762606	1.49E-15	4.15E-13	72.5751647	71.6541831

CDH10	cadherin 10;	3.20547362	5.52E-05	0.00079279	0.48089562	1.06457643
CDH11	cadherin 11;	-2.2759004	2.46E-06	5.95E-05	14.3667568	15.3954131
CDH12	cadherin 12;	-1.76058	0.00015321	0.00182192	19.4562355	18.4253614
CDK1	cyclin-depen	-2.0131398	7.91E-09	4.53E-07	83.2750924	85.0637517
CDK18	cyclin-depen	-1.5518349	9.61E-05	0.00124295	26.0284757	23.8506067
CDK19	cyclin-depen	-1.6034461	2.37E-05	0.00039867	29.7353795	30.3813736
CDK5R2	cyclin-depen	1.99751446	0.01881474	0.07213582	0.62115685	0.96221332
CDKN1A	cyclin-depen	4.2355583	2.70E-29	4.82E-26	161.220258	156.451791
CDKN1B	cyclin-depen	-1.548971	6.79E-08	2.88E-06	59.1100872	55.1327757
CDKN1C	cyclin-depen	-3.3189732	6.11E-10	5.00E-08	25.4473935	24.4443128
CDKN2C	cyclin-depen	-2.0424131	1.41E-13	2.61E-11	47.4483683	45.2035532
CDKN3	cyclin-depen	-1.6529693	5.84E-05	0.00083212	14.6673166	13.5528769
CDON	cell adhesior	-1.8037133	2.84E-09	1.89E-07	48.4301969	53.1264586
CDRT1	CMT1A dupli	2.90209149	0.00041802	0.00402731	0.60111953	0.79843233
CDT1	chromatin lic	-1.6274074	5.39E-07	1.69E-05	63.7988196	63.3218252
CEBPD	CCAAT/enhai	-2.1202258	6.37E-05	0.00088725	10.8201516	12.4883005
CELSR1	cadherin; EG	-1.5957884	0.00271538	0.0171225	26.6496325	25.4679439
CELSR2	cadherin; EG	-1.6004756	0.00247959	0.01598218	70.6916568	75.6053995
CEND1	cell cycle exi	1.95403457	1.23E-07	4.86E-06	9.43757664	8.80322821
CENPA	centromere p	-1.5323279	0.00101395	0.00803659	18.7148547	17.5040933
CENPE	centromere p	-1.52054	0.00074041	0.00627693	64.6604242	68.3785633
CENPF	centromere p	-2.2972098	1.97E-07	7.13E-06	391.729561	409.432002
CENPH	centromere p	-1.6610265	2.24E-09	1.53E-07	42.7395987	42.7263658
CENPI	centromere p	-1.9970888	2.74E-05	0.00044762	5.63048627	6.14178712
CENPK	centromere p	-1.6292599	4.42E-05	0.00066256	20.4781387	20.1450618
CENPU	centromere p	-1.5482216	1.01E-07	4.08E-06	55.5835193	61.5816522
CERKL	ceramide kin	-1.9160807	0.01780583	0.06938276	2.70503789	2.98900307
CERS4	ceramide syr	-3.1382304	8.28E-06	0.00016478	3.64679182	3.6236544
CES2	carboxylester	1.64376256	2.08E-09	1.47E-07	52.7582575	48.8272076
CFAP53	cilia and flag	2.09659581	0.0103358	0.04656828	0.60111953	0.79843233
CFHR3	complement	1.89289587	0.02030323	0.07638972	1.10205247	0.79843233
CFI	complement	-1.9635461	1.90E-05	0.00033364	11.0605994	11.0347442
CHAC1	ChaC glutath	5.30806315	8.57E-05	0.00113154	3.44641864	2.43624223
CHAF1B	chromatin as	-1.6359206	0.00057422	0.00518251	15.328548	13.7985484
CHCHD7	coiled-coil-he	1.59008394	0.00016696	0.00194867	23.2232512	23.2159553
CHEK2	checkpoint ki	-1.5007505	0.00145366	0.01064547	12.0223906	13.65524
CHMP4C	charged mult	1.85904874	6.00E-07	1.84E-05	6.69246411	6.65360272
CHRDL1	chordin-like 1	-2.5574725	1.65E-10	1.59E-08	10.4594798	9.35598905
CHRM4	cholinergic re	-3.0408973	0.01346683	0.05669595	0.86160466	1.53544678
CHRNB2	cholinergic re	4.16106617	4.27E-06	9.56E-05	0.48089562	0.45039772
CHST1	carbohydrate	-1.9091481	8.87E-07	2.55E-05	34.123552	34.168809
CHST11	carbohydrate	-1.7920857	1.75E-06	4.52E-05	117.739279	114.667166
CHST15	carbohydrate	-1.9918925	0.00127208	0.00962415	2.6048513	2.72285896

CHST2	carbohydrate	1.97600568	2.53E-05	0.00041919	6.7926507	6.1213145
CHSY3	chondroitin s	-1.5563539	0.0082231	0.03960374	5.57037432	5.05673806
CIITA	class II; majc	2.32537385	8.20E-15	2.13E-12	14.0862343	15.2521047
CILP2	cartilage inte	2.22155544	0.00012342	0.00152017	1.5829481	1.82206351
CIT	citron rho-int	-1.8251254	1.85E-06	4.76E-05	77.5444195	83.0369619
CITED2	Cbp/p300-int	1.77472747	1.84E-05	0.000325	27.4310879	28.6412006
CITED4	Cbp/p300-int	-2.2662939	4.48E-08	2.00E-06	103.853418	99.0874989
CKAP2L	cytoskeleton	-1.8347832	3.57E-07	1.19E-05	55.8440044	57.8761073
CKMT1B	creatine kina	8.09131189	8.14E-07	2.41E-05	0.48089562	0.18425361
CLDN4	claudin 4	-1.6809745	1.93E-05	0.00033804	38.1310156	35.6837832
CLEC11A	C-type lectin	-1.9497415	1.88E-07	6.92E-06	12.5032862	11.751286
CLGN	calmegin	1.56416092	0.00866238	0.04095807	3.52656792	3.50081866
CLIC4	chloride intra	-1.5909512	3.22E-06	7.49E-05	491.976261	506.922637
CLIP3	CAP-GLY don	-1.6945658	3.61E-09	2.36E-07	86.28069	86.1078555
CLMN	calmin (calpo	-2.8398832	2.15E-13	3.82E-11	17.6128023	17.2379492
CLP1	cleavage anc	1.59157795	5.43E-06	0.00011633	26.0284757	24.0348603
CMTM7	CKLF-like MA	-2.0327266	1.14E-11	1.46E-09	119.903309	125.968054
CMTM8	CKLF-like MA	-1.5344506	0.00291672	0.0180811	10.5997411	9.97016776
CMYA5	cardiomyopa	3.35106502	0.00921839	0.04277799	0.5610449	0.18425361
CNTNAP1	contactin ass	-1.6755453	0.00040404	0.00392233	77.1837478	75.8920162
CNTNAP3	contactin ass	-2.1332705	1.45E-07	5.53E-06	40.6557176	39.1641292
CNTNAP3B	contactin ass	-1.6573119	0.00012883	0.00157457	33.4422832	34.1483364
CNTNAP3P2	contactin ass	-1.6831666	0.01416035	0.05878217	7.19339705	6.87880158
CNTRL	centriolin	-2.3582749	7.54E-06	0.00015327	39.5536651	39.2460197
COBL	cordon-bleu \	1.69563429	3.24E-07	1.10E-05	9.55780054	11.0347442
COCH	cochlin	-1.5068817	0.00615987	0.03184052	10.6197784	11.7308134
COL13A1	collagen; typ	-2.0841136	1.34E-05	0.00024928	87.7033396	88.7897692
COL1A1	collagen; typ	-2.5492985	2.75E-12	4.04E-10	10.2991813	10.0315856
COL4A1	collagen; typ	-2.6213283	9.04E-07	2.59E-05	480.074095	500.064308
COL4A2	collagen; typ	-2.5036278	3.53E-08	1.66E-06	935.12158	941.658802
COL4A6	collagen; typ	2.44504568	0.03889263	0.12099081	0.60111953	0.53228822
COL5A1	collagen; typ	-1.6224656	3.03E-05	0.00048683	67.2652755	71.2447306
COL5A2	collagen; typ	-1.9693929	1.19E-07	4.68E-06	348.989962	358.680368
COL5A3	collagen; typ	2.09475365	2.72E-06	6.52E-05	3.52656792	3.60318178
COL6A1	collagen; typ	-1.9903008	3.87E-10	3.34E-08	256.137032	251.690436
COL6A2	collagen; typ	-2.9705639	2.29E-22	1.72E-19	107.700583	102.772571
COL6A3	collagen; typ	-5.2066131	3.39E-25	3.72E-22	60.2522143	61.6021248
COL8A1	collagen; typ	-3.6336449	5.48E-10	4.59E-08	10.6999277	10.0930035
COL8A2	collagen; typ	-2.3664463	0.00837155	0.04012882	1.10205247	1.22835742
COL9A3	collagen; typ	-2.0503033	1.31E-06	3.50E-05	598.354381	578.085477
COLEC12	collectin sub	-3.6706533	2.11E-09	1.48E-07	21.2595941	17.4836207
COLQ	collagen-like	1.52920377	0.04709783	0.13858996	1.88350786	1.65828252
COMMD3-BM	COMMD3-BM	19.9174931	0.01739873	0.06817414	0	0

CORIN	corin; serine	2.43545965	0.01296464	0.05515195	0.38070904	0.63465134
COX6A1	cytochrome c	1.53158662	0.00069068	0.00595099	147.775218	139.889438
COX7B	cytochrome c	1.50572521	0.00085559	0.00703984	55.4232208	54.4367065
CPEB2	cytoplasmic	1.99708714	8.59E-05	0.00113244	7.43384487	6.85832895
CPEB4	cytoplasmic	1.50973785	0.00023479	0.00254639	38.5317619	39.6554722
CPED1	cadherin-like	-3.4799158	2.01E-05	0.00034788	3.04567229	2.98900307
CPM	carboxypepti	3.69636002	1.45E-07	5.53E-06	1.34250029	2.43624223
CPNE2	copine II	-1.9834541	4.08E-09	2.61E-07	23.5438483	25.6521976
CPQ	carboxypepti	-1.6533703	0.01967357	0.07444717	3.10578424	3.35751029
CPS1	carbamoyl-pl	-1.591105	0.00180755	0.01259011	19.5964967	21.92618
CRIP2	cysteine-rich	-1.8502487	7.62E-08	3.19E-06	102.671216	101.114289
CRISPLD2	cysteine-rich	2.01008841	0.03063917	0.10189874	1.04194052	1.14646693
CRLF1	cytokine rece	-1.9412939	0.00152158	0.01101601	3.40634401	3.07089356
CROT	carnitine O-c	1.72960275	2.51E-06	6.06E-05	9.91847226	8.49613885
CSAG3	CSAG family,	1.54737937	0.00016012	0.00188279	32.6808652	29.0711257
CSF2	colony stimu	-1.6383674	2.13E-05	0.00036562	22.3816839	26.4711025
CSF3	colony stimu	-2.8725163	3.03E-05	0.00048683	2.48462739	3.02994831
CSGALNACT1	chondroitin s	-1.6382417	0.01320477	0.05592308	12.9040326	13.8394937
CSMD3	CUB and Sus	3.5225083	4.84E-10	4.10E-08	2.74511252	2.35435173
CSPG4	chondroitin s	-2.0754819	6.25E-06	0.00013143	696.557275	703.336989
CSRNP2	cysteine-seri	1.5016486	9.65E-06	0.00018831	29.8355661	35.8066189
CT55	cancer/testis	1.92830547	0.0060067	0.03124143	2.24417958	1.2897753
CTDSP2	CTD (carboxy	-1.6422024	5.36E-07	1.69E-05	177.310224	182.247297
CTDSPL	CTD (carboxy	-1.6751699	3.83E-06	8.71E-05	27.811797	28.3136386
CTGF	connective ti	2.59535006	1.42E-05	0.00026188	35.506127	34.7625151
CTH	cystathionine	1.57123731	0.00141263	0.01045243	6.91287461	6.42840386
CTHRC1	collagen tripl	-1.6779413	5.26E-07	1.67E-05	52.3975858	48.970516
CTNNAL1	catenin (cadh	-1.6532862	2.37E-11	2.94E-09	96.0589011	102.21981
CTNS	cystinosin; ly	1.57897722	3.61E-05	0.00056626	14.507018	13.3890959
CTSB	cathepsin B	-1.5292325	9.74E-07	2.78E-05	324.865032	309.975996
CTSS	cathepsin S	-1.8345962	2.77E-05	0.00045089	14.8476524	13.7576032
CTSZ	cathepsin Z	-1.5260849	6.13E-06	0.00012936	263.190168	253.492027
CUBN	cubilin (intrir	-2.1257183	0.00537963	0.02882147	1.72320932	2.43624223
CX3CL1	chemokine (C	-4.7898818	0.00308296	0.01891318	1.64306005	1.04410381
CXADR	coxsackie viru	1.6571313	0.00230559	0.01508585	3.54660523	3.93074376
CXCL8	chemokine (C	-1.9236446	9.36E-16	2.78E-13	373.896348	378.415977
CXXC5	CXXC finger p	-1.7778403	0.00011077	0.00139872	22.3616466	21.8852348
CXorf40B	chromosome	1.56771002	0.002801	0.01753057	14.4669434	11.1575799
CXorf57	chromosome	1.95333358	0.01624751	0.06491285	1.52283615	0.92126807
CYCS	cytochrome c	1.50875824	2.06E-05	0.00035444	122.367899	131.454717
CYFIP2	cytoplasmic	2.80473622	2.27E-33	6.46E-30	43.1203077	38.7546767
CYGB	cytoglobin	-3.0209151	0.0013031	0.00981524	0.92171661	1.33072054
CYP1A1	cytochrome P	4.03913125	1.49E-06	3.94E-05	3.2861201	2.37482435

CYP1B1	cytochrome I	6.01417045	1.93E-10	1.82E-08	0.78145539	1.43308366
CYP2E1	cytochrome I	1.60806299	0.03502025	0.11235336	1.26235102	1.18741218
CYP4F11	cytochrome I	3.06021202	0.00453847	0.02522534	0.24044781	0.40945247
CYP4F3	cytochrome I	55.4342527	7.11E-07	2.14E-05	0.04007464	0
CYP4X1	cytochrome I	-2.0294543	0.00236039	0.01536679	3.68686646	3.33703767
CYR61	cysteine-rich	2.03477489	0.00040618	0.00393243	82.2732265	77.099901
CYSRT1	cysteine-rich	1.85696	0.00962356	0.04416919	1.90354518	1.43308366
DACH1	dachshund fa	-2.0896601	0.0052405	0.02822263	2.6048513	2.04726237
DAPK1	death-associ	2.03693108	4.69E-11	5.35E-09	12.0624653	11.9969575
DAPK3	death-associ	1.71950238	3.33E-08	1.58E-06	56.885945	52.5532252
DCAF4L1	DDB1 and CU	2.51225394	0.00698605	0.03495722	0.5610449	0.57323346
DCBLD1	discoidin; CU	-1.5155379	0.03446076	0.11093299	18.1538098	16.3576264
DCHS1	dachsous cad	-3.2570918	8.83E-07	2.55E-05	2.86533643	3.60318178
DCLK1	doublecortin-	1.64566642	0.0048066	0.02635585	5.24977724	5.3433548
DCLRE1B	DNA cross-lir	-1.5358934	0.00012434	0.00152776	19.616534	18.8143412
DDB2	damage-spe	2.12160142	1.93E-14	4.16E-12	44.8835916	45.3059163
DDIT3	DNA-damage	4.83193429	1.74E-05	0.00030846	10.3592933	8.70086509
DDN	dendrin	2.32424177	1.21E-06	3.32E-05	1.96365713	2.04726237
DDR2	discoidin dor	-1.5513958	0.00389369	0.02246518	38.8323217	40.5153224
DDX60	DEAD (Asp-G	-1.9296155	5.07E-05	0.00074023	8.97671833	11.8945944
DENND2A	DENN/MADD	-2.159461	7.43E-09	4.32E-07	25.8281025	27.044336
DENND2C	DENN/MADD	3.51027186	0.00076423	0.00641786	0.58108221	0.38897985
DEPDC1	DEP domain	-1.5430241	0.00565569	0.02981842	76.0416207	77.8369155
DEPDC7	DEP domain	3.06215975	7.29E-12	9.70E-10	11.160786	11.8945944
DGKG	diacylglycero	2.00700227	6.87E-10	5.59E-08	7.69433	8.37330311
DHFR	dihydrofolate	-1.6764752	6.78E-09	4.03E-07	112.890248	112.599431
DHRS12	dehydrogena	1.88917305	0.00385199	0.02227869	1.32246297	1.16693955
DHRS13	dehydrogena	-1.9035738	5.52E-10	4.60E-08	21.5401165	22.908866
DHRS3	dehydrogena	-2.2455701	1.46E-16	4.74E-14	113.691741	106.130081
DHTKD1	dehydrogena	-1.5208168	0.00023452	0.0025454	43.5410914	40.3924866
DIAPH3	diaphanous-r	-1.6591135	0.00147807	0.01076339	17.6528769	19.3466294
DIP2C	disco-interac	-1.5061681	0.01001376	0.04553482	14.56713	15.7434477
DIRAS1	DIRAS family	-2.1069568	6.44E-05	0.00089243	4.20783672	4.23783311
DISP2	dispatched h	1.54720934	0.00276592	0.01736434	6.07130726	6.30556811
DKK1	dickkopf WN	2.67356361	4.22E-12	5.83E-10	118.781219	120.747535
DKK3	dickkopf WN	-2.5798042	1.15E-10	1.16E-08	19.2157877	18.0773268
DLG4	discs; large h	-1.6707905	3.02E-08	1.45E-06	67.485686	67.9486382
DLGAP5	discs; large (	-1.623128	7.03E-05	0.00096173	59.9716919	54.9075769
DLL1	delta-like 1 (	4.46651077	0.01249871	0.05374746	0.32059708	0.22519886
DLL3	delta-like 3 (	1.93749532	0.00145281	0.01064476	3.38630669	2.90711257
DLL4	delta-like 4 (	2.34033552	0.0161322	0.06458068	0.38070904	0.45039772
DMBT1	deleted in m	1.8324924	0.0002778	0.0028982	3.92731427	4.15594262
DMKN	dermokine	1.93644501	5.46E-06	0.00011675	8.47578539	8.39377573

DNAH5	dynein; axon	1.89529001	1.29E-06	3.48E-05	4.60858307	4.89295707
DNAJB9	DnaJ (Hsp40)	1.71785148	1.97E-05	0.00034352	16.0298542	16.705661
DNMT3B	DNA (cytosin	-1.7770051	0.03685801	0.11631168	4.10765013	3.45987341
DOCK1	dedicator of	-1.5095836	0.00149497	0.01087532	102.050059	113.356918
DOCK11	dedicator of	-1.8804306	1.10E-05	0.00021116	19.0354518	18.8552865
DOCK6	dedicator of	-1.5281909	0.00015922	0.00187528	38.9525456	34.8648782
DOHH	deoxyhypusir	1.6194764	0.00052707	0.00484911	20.938997	19.6741914
DPF3	D4; zinc and	1.87606636	0.0458972	0.1358706	1.16216443	0.75748708
DPYSL2	dihydropyrim	-1.6614721	2.62E-09	1.77E-07	100.226663	96.2418042
DPYSL3	dihydropyrim	-1.985567	8.62E-09	4.88E-07	228.225049	225.751622
DQX1	DEAQ box RN	2.51014882	0.01790467	0.0696396	0.60111953	0.47087035
DRAM1	DNA-damage	1.65918306	1.28E-08	6.86E-07	31.658962	33.6365208
DRAP1	DR1-associat	1.63896767	0.00035736	0.00354003	236.079677	218.627149
DSC3	desmocollin	1.78173205	1.10E-05	0.00021149	10.2991813	12.5497184
DUSP1	dual specifici	1.88831472	4.08E-05	0.00062575	13.0442938	12.3449921
DUSP10	dual specifici	2.74023972	6.45E-09	3.88E-07	9.67802445	10.0315856
DUSP14	dual specifici	2.00825475	2.18E-07	7.74E-06	30.7973573	32.7152527
DUSP5P1	dual specifici	-1.987804	0.00014822	0.001775	4.22787403	4.25830574
DUSP8	dual specifici	8.04197098	2.08E-06	5.24E-05	0.10018659	0.3480346
DUSP9	dual specifici	-1.8686997	0.00089871	0.007348	5.12955333	5.85517039
DUT	deoxyuridine	-1.5523023	9.25E-07	2.64E-05	121.486257	121.750693
DYNC1I1	dynein; cytop	2.09359753	0.00026111	0.00276453	1.703172	1.88348138
DYRK3	dual-specific	1.99521662	5.58E-08	2.44E-06	18.5345189	19.2647389
E2F1	E2F transcrip	-1.5123479	8.52E-05	0.00112707	37.6701573	38.7751494
E2F2	E2F transcrip	-1.8285323	0.00556581	0.02947542	7.49395682	8.6189746
E2F8	E2F transcrip	-1.5517363	0.00339286	0.02028172	5.85089677	6.039424
EAF2	ELL associate	1.68223723	0.02172915	0.08026494	2.30429154	1.71970039
EBI3	Epstein-Barr	-1.765388	0.00015372	0.00182559	16.21019	15.0678511
ECE1	endothelin co	-1.6353699	2.46E-08	1.22E-06	140.621896	138.763444
ECM1	extracellular	-1.665051	4.65E-05	0.00068838	31.4585888	31.6711489
EDA2R	ectodysplasir	3.02388587	2.31E-30	5.49E-27	10.9403755	11.8331765
EEF1A2	eukaryotic tr	-1.9089876	1.32E-14	3.19E-12	125.914504	125.497184
EFCAB13	EF-hand calc	-1.5215325	0.03871857	0.12057936	3.847165	3.88979851
EFEMP1	EGF containi	-1.6674487	8.90E-06	0.00017636	13.2847416	13.4709864
EFEMP2	EGF containi	-2.1001415	8.61E-08	3.55E-06	28.1724687	27.5561516
EFHD2	EF-hand dom	-1.5158549	3.47E-07	1.17E-05	177.350299	175.900783
EFNA3	ephrin-A3	-2.1395274	2.14E-08	1.08E-06	14.8075778	15.5387214
EFNA5	ephrin-A5	-1.6989144	0.00020272	0.00227482	10.8401889	10.0315856
EFNB3	ephrin-B3	-2.2376374	1.25E-08	6.70E-07	17.151944	14.2080009
EFR3B	EFR3 homolo	3.12043067	1.55E-11	1.96E-09	3.04567229	4.23783311
EGR1	early growth	11.5162105	3.85E-11	4.50E-09	6.1113819	7.71817915
EGR2	early growth	4.45478748	0.00010294	0.00131685	0.66123148	0.38897985
EID3	EP300 intera	4.49451322	7.78E-06	0.00015765	0.5610449	0.36850723

EIF3C	eukaryotic tra	2.65240696	0.02777689	0.09532349	14.9278017	3.64412703
EIF4E3	eukaryotic tra	-1.6249926	0.02407542	0.08607168	3.58667987	3.80790802
ELF3	E74-like facto	-6.2113121	1.72E-13	3.15E-11	7.8145539	7.94337801
ELFN1-AS1	ELFN1 antise	1.59190792	0.01713891	0.06741883	3.36626937	1.94489926
ELMOD1	ELMO/CED-1	2.00848267	1.19E-05	0.00022556	3.74697841	3.68507227
ELMSAN1	ELM2 and My	-1.7332173	2.67E-06	6.42E-05	56.9059823	58.1013062
EMC3-AS1	EMC3 antiser	-1.8418644	6.18E-05	0.00086952	7.05313583	6.83785633
EMILIN1	elastin micro	-1.9706764	8.74E-11	9.22E-09	126.3954	121.484549
EMP1	epithelial me	-1.5603315	2.06E-08	1.05E-06	307.051856	300.108191
EMP2	epithelial me	-1.5115158	0.00013229	0.00161	73.4968813	70.4872435
EN2	engrailed hor	1.80459583	1.06E-05	0.00020481	12.2227638	10.3182024
ENO2	enolase 2 (ga	-1.5884248	8.97E-07	2.58E-05	155.128914	154.609255
ENOX2	ecto-NOX dis	1.53293503	0.00020899	0.00232497	13.685488	12.9591708
ENPP2	ectonucleotic	1.93433951	0.02650101	0.09233884	0.82153003	0.98268594
EPAS1	endothelial P	-1.9408558	4.49E-08	2.00E-06	14.7875405	14.4127271
EPHA7	EPH receptor	-2.0520471	0.00193187	0.01319168	45.9255322	49.1547696
EPHB3	EPH receptor	-1.8435116	5.45E-07	1.70E-05	12.9240699	12.7544446
EPHB6	EPH receptor	1.98726146	0.04039762	0.12428897	0.44082099	0.51181559
EPHX2	epoxide hydr	1.58566569	0.02506821	0.0886689	1.72320932	1.37166579
EPPK1	epiplakin 1	7.26952818	7.75E-05	0.00104446	0.16029854	0.02047262
ERAP1	endoplasmic	-1.529119	0.00084254	0.00694454	103.733194	102.854462
ERBB3	erb-b2 recep	-1.5942486	1.97E-05	0.00034288	212.175157	217.726354
ERRFI1	ERBB recepto	-1.6844285	6.08E-11	6.72E-09	397.420159	396.206687
ESM1	endothelial c	1.90307652	1.38E-07	5.31E-06	101.468977	105.045032
ESPL1	extra spindle	-1.83233	7.62E-06	0.00015466	61.995461	65.839958
ESPN	espin	-2.2512123	7.64E-07	2.28E-05	23.8644454	20.349788
ESPNP	espin pseudo	-1.9227436	0.01823725	0.07050482	2.76514984	1.86300876
EVI2A	ecotropic vira	-2.2844216	0.02024962	0.07624696	1.04194052	1.57639203
EVL	Enah/Vasp-li	-1.5384327	0.00236649	0.01539244	8.27541221	7.77959702
EXO1	exonuclease	-1.612229	6.11E-06	0.00012923	44.6231065	43.422435
F2R	coagulation f	-2.1810323	7.91E-11	8.54E-09	139.439694	145.355629
F2RL1	coagulation f	-3.3067256	7.30E-13	1.18E-10	28.0522448	30.360901
F8	coagulation f	1.81692183	0.00077647	0.00650692	3.64679182	3.54176391
FA2H	fatty acid 2-h	7.11461252	0.00382598	0.02215525	0.26048513	0.10236312
FABP5	fatty acid bin	1.60695308	5.86E-05	0.00083388	28.4329538	26.6144109
FABP6	fatty acid bin	1.76168769	0.01513621	0.06178919	2.08388104	2.10868025
FABP7	fatty acid bin	-2.2511771	0.00630172	0.03244429	1.84343323	2.39529698
FAIM2	Fas apoptotic	-2.1573423	2.46E-05	0.00040963	11.7218309	10.5229286
FAM101B	family with s	-1.8647698	8.22E-06	0.00016401	7.83459122	7.88196014
FAM110A	family with s	-1.5202916	0.02538838	0.08949043	6.01119531	6.22367762
FAM118B	family with s	1.60622826	3.56E-05	0.00056006	15.2083241	15.5387214
FAM126B	family with s	1.5993764	0.0002875	0.0029776	7.91474049	8.02526851
FAM131B	family with s	-1.7597491	0.00106736	0.00836228	14.947839	16.9922777

FAM131C	family with s	-2.1762099	0.00030089	0.00308484	3.10578424	2.74333158
FAM132B	family with s	-1.7823234	0.01643514	0.06540553	2.30429154	2.39529698
FAM13B	family with s	1.5473153	3.86E-05	0.0005979	30.7572827	33.4932124
FAM155A	family with s	-1.7199494	0.00084424	0.00695448	7.19339705	7.90243277
FAM155B	family with s	1.68556644	0.0451059	0.13402922	1.48276151	0.81890495
FAM161A	family with s	-1.5205053	0.00541693	0.02895728	8.21530026	10.3796202
FAM167A	family with s	-1.5330227	0.00530127	0.02845514	7.09321047	7.06305519
FAM171A1	family with s	-1.9529251	2.09E-09	1.47E-07	26.5694833	25.7955059
FAM171A2	family with s	-1.8468708	0.00015585	0.00183965	8.0349644	7.61581603
FAM172A	family with s	-1.5088494	0.00072133	0.0061371	24.8663113	26.8191371
FAM180A	family with s	2.82189697	0.0007009	0.00600995	0.46085831	0.83937757
FAM189A2	family with s	2.0602531	0.02462924	0.08739823	0.58108221	0.73701445
FAM198B	family with s	3.26283537	1.91E-20	1.19E-17	4.5284338	4.31972361
FAM19A2	family with s	4.112418	7.25E-05	0.0009874	0.20037318	0.36850723
FAM200A	family with s	1.58738871	7.93E-06	0.00015995	11.922204	10.4819834
FAM20C	family with s	-2.899076	1.59E-17	5.97E-15	176.14806	173.505486
FAM212B	family with s	2.3974622	4.83E-18	1.97E-15	15.8895929	17.4631481
FAM212B-AS	FAM212B an	2.09371372	0.03064613	0.10189874	0.86160466	0.49134297
FAM222A	family with s	1.58433513	0.00205987	0.01384024	6.93291192	6.32604074
FAM225B	family with s	1.89428341	0.01563946	0.06324574	4.70876966	1.67875515
FAM227A	family with s	1.61046132	0.01387664	0.05803841	2.16403031	2.21104336
FAM27B	family with s	2.79434916	8.15E-07	2.41E-05	5.30988919	3.29609242
FAM64A	family with s	-1.5981278	0.00074195	0.00628251	22.0610868	21.4348371
FAM65B	family with s	-1.7709473	0.01636973	0.06529106	2.78518716	3.7055449
FAM69B	family with s	-1.5641305	0.00013662	0.0016584	15.2083241	16.0300644
FAM72A	family with s	-1.5471874	0.00017005	0.00197822	23.0028407	25.897869
FAM72B	family with s	-1.5867978	2.71E-05	0.00044392	20.1976162	20.841131
FAM72D	family with s	-1.734728	0.00296607	0.01832226	19.9170938	14.7402891
FAM83D	family with s	-1.5274623	1.35E-07	5.20E-06	59.851468	59.5343898
FAM83H	family with s	2.29713438	2.82E-10	2.56E-08	5.73067286	4.8315392
FAM84A	family with s	2.72289216	0.00538781	0.02884361	0.54100758	0.26614411
FAM90A1	family with s	1.80562326	0.02304403	0.08337153	1.2423137	1.22835742
FANCD2	Fanconi aner	-1.7694995	1.61E-05	0.00029151	39.8742622	43.2791266
FANCI	Fanconi aner	-1.651398	1.96E-09	1.41E-07	107.159575	111.555327
FAR2	fatty acyl CoA	-1.838906	1.15E-05	0.00021963	22.0410495	21.4348371
FARP1	FERM; RhoG	-2.2500112	1.53E-08	8.04E-07	73.2363962	71.9817451
FAS	Fas cell surfa	2.93336019	3.78E-20	2.15E-17	16.3504512	19.0600127
FAXDC2	fatty acid hyd	-1.5009739	0.00080353	0.00668093	17.4324664	16.0095918
FBLIM1	filamin bindi	1.52925445	0.01580169	0.06363094	3.30615742	3.72601752
FBLN1	fibulin 1	-3.2628986	8.58E-08	3.55E-06	4.60858307	5.17957381
FBXO22	F-box proteir	1.74273686	8.06E-12	1.06E-09	47.9693386	45.6334783
FBXO22-AS1	FBXO22 antis	1.59722035	0.00201377	0.01358494	11.100674	12.8363351
FBXO43	F-box proteir	-1.633659	0.01072157	0.04779267	4.78891893	4.34019623

FBXO5	F-box protei	-1.8405104	2.58E-09	1.75E-07	25.2470203	25.6521976
FBXW7	F-box and W	1.7395139	4.30E-06	9.59E-05	13.7055253	12.1402659
FCGR2A	Fc fragment	-1.7814744	0.00039911	0.00388239	5.91100872	6.79691108
FCMR	Fc fragment	2.0463212	0.00242601	0.01569366	1.08201516	1.39213841
FDX1L	ferredoxin 1-	1.80731904	4.47E-05	0.0006664	16.4506378	16.6647157
FDXR	ferredoxin re	3.26405682	2.73E-19	1.39E-16	25.9082518	24.5057306
FGFR1	fibroblast gr	-1.89202	1.94E-09	1.40E-07	83.4153536	82.0747486
FGFR3	fibroblast gr	-1.614623	0.00926096	0.04284997	8.73627052	9.3150438
FGFRL1	fibroblast gr	-1.7826826	7.12E-05	0.00097138	150.29992	149.511571
FHL1	four and a ha	-1.696579	1.99E-08	1.02E-06	30.4366856	31.8349299
FHL2	four and a ha	2.25259534	1.17E-09	8.98E-08	36.6883287	36.4412703
FIBCD1	fibrinogen C	-2.6046148	0.00130301	0.00981524	3.12582156	3.35751029
FKBP9P1	FK506 bindin	-1.880401	0.00328367	0.01979511	3.80709036	3.13231143
FLJ26245	NA	-2.6868737	9.47E-06	0.00018572	3.44641864	3.91027114
FLJ36000	NA	1.88284007	0.02275892	0.0827586	0.70130612	1.10552168
FLJ37201	NA	-1.9200704	0.01358389	0.05708758	1.9235825	1.76064564
FMNL1	formin-like 1	-1.6061154	1.96E-06	4.98E-05	27.2707894	26.5939382
FMNL3	formin-like 3	-1.6966092	0.0001766	0.00203022	56.8458703	58.2650872
FN1	fibronectin 1	-2.9820218	4.32E-07	1.41E-05	766.467477	784.429051
FNDC4	fibronectin ty	-1.5414338	0.01032903	0.04655248	4.46832185	4.38114148
FOLR1	folate recept	2.14301047	0.02489858	0.08815643	0.36067172	0.8598502
FOS	FBJ murine c	2.74336587	0.00032016	0.00323364	3.88723963	3.33703767
FOSB	FBJ murine c	3.89899919	3.26E-12	4.70E-10	3.42638133	3.31656505
FOXD2	forkhead box	-1.832217	0.00099218	0.00790801	7.53403146	8.1890495
FOXD2-AS1	FOXD2 antis	-1.6131416	0.00399864	0.02291299	7.1332851	8.00479588
FOXL1	forkhead box	-1.5045466	0.04244185	0.12863438	3.1658962	4.77012133
FOXM1	forkhead box	-1.6520469	4.38E-08	1.97E-06	164.406192	170.536956
FOXP1	forkhead box	-1.5532802	5.60E-06	0.0001197	28.4930658	28.8254542
FREM1	FRAS1 relate	-2.9512122	0.0007802	0.00653272	1.56291078	2.27246124
FREM2	FRAS1 relate	-2.3892681	1.88E-07	6.92E-06	35.506127	40.7200486
FRG2	FSHD region	8.33650932	0.00298661	0.01843319	0.12022391	0.10236312
FRMD4A	FERM domai	-1.7840857	2.09E-10	1.94E-08	128.659617	132.130314
FSCN1	fascin actin-l	-1.5106336	4.11E-06	9.27E-05	486.866745	475.886139
FSTL1	follistatin-lik	-1.8623561	6.21E-09	3.77E-07	51.6361677	56.5044415
FTH1	ferritin; heav	1.89880123	2.43E-05	0.00040565	2600.10246	2490.94508
FTH1P3	ferritin; heav	2.0344804	0.04230536	0.12838452	1.12208979	0.98268594
FUNDC2	FUN14 doma	1.59475249	0.00018324	0.00208739	36.0872092	39.3074376
FUT11	fucosyltransf	-1.6216232	9.18E-05	0.00119849	26.2488862	25.734088
FXYD3	FXYD domair	-2.3258171	3.72E-06	8.47E-05	10.8602262	9.99064039
FXYD6	FXYD domair	-3.4938389	3.54E-05	0.00055704	1.66309737	1.53544678
FXYD7	FXYD domair	-3.6725015	4.47E-06	9.90E-05	2.9454857	3.09136619
FZD1	frizzled class	-2.6758079	3.44E-12	4.91E-10	76.6026656	73.4762466
FZD2	frizzled class	-1.7779248	1.28E-08	6.86E-07	45.34445	42.4806943

GABRE	gamma-ami	-1.6750985	0.00318613	0.01939184	5.57037432	5.15910118
GADD45A	growth arres	5.25715232	9.37E-13	1.47E-10	36.1473211	32.0601288
GADD45B	growth arres	2.33181164	0.00029029	0.00299829	9.57783786	9.45835217
GAL	galanin/GMA	1.61656231	0.02561197	0.0900558	1.74324664	1.82206351
GAL3ST4	galactose-3-(	1.65760198	0.01126786	0.04976088	2.42451544	3.11183881
GALNT12	polypeptide I	2.03837327	6.27E-06	0.00013143	2.78518716	2.96853044
GAN	gigaxonin	1.77179336	0.00083377	0.0068842	3.60671719	3.29609242
GAP43	growth asso	2.25782244	1.21E-05	0.00022884	2.92544838	2.96853044
GAREM	GRB2 associ	1.79576476	4.19E-05	0.00063507	3.86720232	4.52444985
GAREML	GRB2 associ	-1.7739377	0.00018603	0.00211579	8.55593466	7.45203504
GAS6-AS1	GAS6 antiser	2.81795992	1.94E-10	1.82E-08	2.48462739	1.78111827
GAS6-AS2	GAS6 antiser	3.02332645	0.00047803	0.00448483	0.58108221	0.61417871
GAST	gastrin	10.1125699	1.26E-05	0.00023703	0.04007464	0.12283574
GATA3	GATA binding	-1.6693577	0.00024939	0.00267831	10.9804501	9.47882479
GATSL3	GATS proteir	-2.1409582	0.0017538	0.01228778	3.68686646	3.31656505
GBA	glucosidase;	1.50904966	2.27E-07	8.00E-06	67.0248277	70.2620447
GBAP1	glucosidase;	1.60038034	0.03226971	0.10566995	1.62302273	1.82206351
GBP1	guanylate bir	-2.0235384	0.00013717	0.00166371	11.4613457	10.5638739
GBP2	guanylate bir	-1.7930467	0.00024618	0.00264776	19.235825	18.2615804
GCLM	glutamate-cy	1.62314764	2.76E-06	6.60E-05	42.258703	40.6586308
GDA	guanine dear	1.5526025	4.79E-06	0.00010499	92.3519973	94.2150145
GDF15	growth differ	5.46140143	4.67E-18	1.96E-15	10.0386962	10.2567845
GDPGP1	GDP-D-gluco	1.79525167	0.00285354	0.01778903	2.16403031	2.53860534
GEM	GTP binding	1.90206654	7.19E-06	0.00014745	12.0624653	11.587505
GFPT2	glutamine-fr	-1.5134481	6.82E-05	0.00093935	129.861856	124.391662
GFRA1	GDNF family	2.27081988	0.03676038	0.11613218	0.28052245	0.96221332
GGT1	gamma-gluta	2.5860991	5.45E-05	0.00078389	1.2423137	1.04410381
GINS1	GINS comple	-1.5094834	1.43E-05	0.00026237	35.5261643	35.9703999
GINS2	GINS comple	-1.7239715	1.67E-06	4.35E-05	27.1305282	27.0648086
GJA3	gap junction	1.54763786	7.91E-05	0.00106267	9.07690492	8.51661148
GJA5	gap junction	-3.9420657	1.20E-08	6.55E-07	35.8467614	39.286965
GJB3	gap junction	1.69799823	2.64E-07	9.19E-06	29.1943719	31.6506763
GJC2	gap junction	-1.7455071	0.00264197	0.01677836	23.8844827	21.7009812
GLDN	gliomedin	-2.9102979	0.00399599	0.02291299	1.38257492	1.35119317
GLI3	GLI family zi	-1.5765072	0.00731705	0.03624443	47.3081071	44.3027578
GLIS2	GLIS family z	-1.6110701	4.34E-06	9.66E-05	40.9162027	41.1704463
GLIS3	GLIS family z	-2.0711973	1.78E-07	6.62E-06	86.6413617	87.3771581
GLRX2	glutaredoxin	1.61414051	0.00053865	0.00492698	18.4343323	19.1828484
GLS2	glutaminase	4.23685774	0.0026688	0.01691108	0.24044781	0.40945247
GM2A	GM2 ganglio	1.51897703	4.88E-06	0.00010659	36.8887019	35.9703999
GMPR	guanosine m	2.59998296	0.04107022	0.12567997	0.20037318	0.30708936
GNAL	guanine nucl	-1.5171287	0.04124301	0.12599232	4.60858307	4.27877836
GNAO1	guanine nucl	-1.5218955	0.00011425	0.00142953	14.2264956	14.2284735

GNAS	GNAS compl	-1.5378998	9.69E-06	0.00018831	1280.64509	1269.93732
GNG4	guanine nucl	-2.2077755	7.57E-09	4.38E-07	69.2890446	74.7660219
GNG7	guanine nucl	-1.5175575	0.03032923	0.10115753	2.96552302	2.68191371
GOLGA6L5P	golgin A6 far	-3.3000931	0.00905177	0.0423331	1.0219032	1.31024792
GOLT1A	golgi transpo	1.64733016	0.02533732	0.08935464	3.04567229	2.37482435
GPATCH4	G patch dom	1.57799686	7.20E-06	0.00014745	113.030509	102.076502
GPC1	glypican 1	1.59330005	3.37E-06	7.81E-05	41.817882	39.4712186
GPD1	glycerol-3-ph	2.38927548	0.01323154	0.0560198	0.38070904	0.63465134
GPR135	G protein-co	-1.6088988	0.0228402	0.08292912	3.04567229	2.68191371
GPR146	G protein-co	-2.6870671	0.00053189	0.00488715	4.44828453	3.64412703
GPR153	G protein-co	-2.0336018	1.42E-06	3.78E-05	117.859503	122.528653
GPR160	G protein-co	-1.6655952	0.01051719	0.04719158	4.00746354	4.09452475
GPR161	G protein-co	-1.5442684	0.00011169	0.00140862	49.4521001	46.9232536
GPR179	G protein-co	2.220756	0.00864882	0.04092107	0.62115685	0.45039772
GPR25	G protein-co	-4.0071434	0.00347236	0.02057572	1.2423137	1.2078848
GPR39	G protein-co	-1.6583268	0.00028643	0.00296871	8.91660638	8.63944722
GPR85	G protein-co	2.61952432	4.89E-07	1.58E-05	1.54287346	1.65828252
GPR87	G protein-co	22.411408	1.40E-08	7.42E-07	0.10018659	0.06141787
GPRC5A	G protein-co	1.65964695	1.90E-09	1.38E-07	357.966681	379.030156
GPRC5B	G protein-co	-1.8825745	2.01E-05	0.00034788	7.93477781	8.43472098
GPRC5D	G protein-co	2.14023903	0.02910391	0.09851062	0.48089562	0.38897985
GPS2	G protein pat	1.50353576	0.00018718	0.00212448	76.5826283	72.9030132
GPSM2	G-protein sig	-1.9365013	4.27E-08	1.95E-06	31.7391112	33.247541
GPX1	glutathione p	1.64814563	8.58E-05	0.00113154	222.915159	215.43342
GPX3	glutathione p	1.72367313	2.01E-05	0.00034811	10.1388828	10.9528537
GRAMD1C	GRAM doma	-1.5122836	0.03518234	0.11274645	3.98742622	3.1937293
GREB1	growth regul	2.43157864	2.68E-05	0.00043934	2.78518716	3.00947569
GRHL3	grainyhead-li	3.78630771	3.06E-06	7.18E-05	0.9016793	0.51181559
GRID2	glutamate re	-2.4384734	1.53E-05	0.00027868	4.42824721	4.03310688
GRIN2C	glutamate re	3.43375849	0.00037074	0.00364369	0.36067172	0.36850723
GRK6	G protein-co	-1.6341472	2.16E-09	1.49E-07	45.7652336	43.9342506
GSG2	germ cell ass	-1.5603338	0.00557139	0.029494	11.5615323	12.6725541
GSTT2B	glutathione S	2.07769879	0.03208774	0.10538889	1.38257492	1.37166579
GTSE1	G-2 and S-ph	-1.5966652	0.00076896	0.00645375	35.6664255	35.6633106
GUSBP1	glucuronidas	1.50257548	0.01311811	0.0556719	5.45015041	5.6913894
GXYLT2	glucoside xyl	-1.5469821	0.01848175	0.07120394	11.3010472	11.423724
GYLTL1B	glycosyltrans	-2.1493966	0.03546877	0.11335179	1.34250029	1.24883005
H19	H19; imprint	-4.6089713	1.86E-09	1.36E-07	5.28985187	3.66459965
H1FX-AS1	H1FX antiser	-1.9707407	0.00913483	0.04258437	2.38444081	2.23151599
HAP1	huntingtin-as	2.50004867	0.00549626	0.02928121	0.86160466	0.53228822
HAPLN1	hyaluronan a	-2.7902019	2.69E-07	9.33E-06	30.3164617	32.592417
HAPLN3	hyaluronan a	-1.8941445	3.06E-06	7.18E-05	12.2027265	13.3072054
HAS2	hyaluronan s	-2.1242908	0.00122959	0.00939059	15.1482122	16.0095918

HBEGF	heparin-bind	1.90460746	1.35E-05	0.00025077	11.922204	11.5260872
HCN1	hyperpolariza	-4.8951422	0.0002263	0.0024675	4.76888161	5.28193693
HCP5	HLA complex	1.55957536	0.00519922	0.02806592	2.86533643	4.23783311
HDAC9	histone deac	1.89134092	0.00090114	0.0073594	10.0186589	11.1371073
HECW1	HECT; C2 anc	2.88003593	0.00018192	0.00207571	0.76141807	1.24883005
HEG1	heart develo	-1.9266772	1.73E-05	0.00030757	126.876296	140.196527
HERC3	HECT and RL	-1.6565064	0.00061086	0.00544426	25.1267964	24.0962781
HES6	hes family bl	1.67109999	0.00447545	0.02500182	3.48649328	2.9275852
HEY1	hes-related f	-1.8085111	0.00115671	0.00891521	5.39003846	5.28193693
HEY2	hes-related f	-2.6693906	0.00648905	0.03314345	0.76141807	1.5559194
HHAT	hedgehog ac	1.82378493	2.83E-07	9.80E-06	11.6016069	12.3245195
HHEX	hematopoiet	-1.639839	3.28E-07	1.11E-05	30.4767602	30.0947569
HIBADH	3-hydroxyisol	-1.5513577	1.26E-06	3.43E-05	60.7731846	59.309191
HIC1	hypermethyl	3.77126101	0.00029984	0.00308206	0.28052245	0.71654183
HID1	HID1 domain	-1.5671345	0.00644385	0.03296935	6.15145653	6.81738371
HILPDA	hypoxia indu	-2.829223	1.99E-25	2.36E-22	29.6752675	31.5892584
HIP1	huntingtin in	-1.5623186	0.0005628	0.00509885	82.0327787	87.9913369
HIST1H1C	histone clust	4.25865624	3.08E-11	3.68E-09	16.0498915	15.3749404
HIST1H1E	histone clust	3.89313718	0.00819908	0.03958169	0.24044781	0.14330837
HIST1H2AC	histone clust	3.03768523	2.86E-12	4.16E-10	3.94735159	3.91027114
HIST1H2AE	histone clust	3.13999979	0.00139639	0.01036995	0.58108221	0.26614411
HIST1H2BD	histone clust	3.51838529	8.51E-15	2.17E-12	10.4594798	8.6189746
HIST1H2BG	histone clust	7.41600658	1.18E-07	4.66E-06	0.50093294	0.32756198
HIST1H2BK	histone clust	3.7930876	1.17E-17	4.50E-15	17.8532501	15.3954131
HIST1H3D	histone clust	5.39711228	8.79E-08	3.62E-06	0.6812688	0.40945247
HIST1H3H	histone clust	3.89264959	4.59E-05	0.00067967	0.42078367	0.3480346
HIST1H4H	histone clust	6.58483286	9.83E-06	0.00019087	0.42078367	0.16378099
HIST1H4K	histone clust	2.29022983	0.02968054	0.09975883	1.22227638	0.69606921
HIST2H2AA3	histone clust	3.92252138	1.69E-15	4.54E-13	10.0386962	9.68355103
HIST2H2AA4	histone clust	3.92252131	1.68E-15	4.54E-13	10.0386962	9.68355103
HIST2H2BC	histone clust	2.10756181	2.02E-06	5.12E-05	4.26794867	3.39845554
HIST2H2BE	histone clust	4.05826689	4.42E-25	4.50E-22	4.78891893	5.28193693
HIST2H2BF	histone clust	2.65121368	0.0001183	0.00147328	0.92171661	1.24883005
HIST2H3A	histone clust	3.20576492	8.18E-06	0.00016401	0.82153003	0.96221332
HIST2H3C	histone clust	3.20576446	8.19E-06	0.00016401	0.82153003	0.96221332
HIST2H4A	histone clust	2.51188769	1.88E-07	6.92E-06	3.70690377	3.7055449
HIST2H4B	histone clust	2.51188769	1.88E-07	6.92E-06	3.70690377	3.7055449
HIST3H2A	histone clust	1.81127214	0.00141361	0.01045426	5.02936674	4.62681297
HIVEP3	human immu	-2.3738806	3.99E-08	1.83E-06	72.3547542	76.3833592
HJURP	Holliday junc	-1.6564393	3.50E-07	1.17E-05	47.1878832	46.5342738
HKDC1	hexokinase d	-3.8111003	3.27E-05	0.00051817	4.64865771	5.01579282
HLA-DMB	major histoc	2.40303927	1.12E-11	1.45E-09	4.5284338	4.97484757
HLA-DOA	major histoc	2.62407503	3.21E-06	7.48E-05	1.80335859	1.92442663

HLA-DQA1	major histoc	2.23277859	0.00700583	0.03501931	0.62115685	0.96221332
HLA-DQB1	major histoc	1.64598036	0.0010999	0.00853737	3.76701573	3.80790802
HLA-DQB2	major histoc	1.80643192	0.00779406	0.0381826	2.78518716	2.04726237
HLA-DRA	major histoc	1.53860207	2.15E-06	5.37E-05	71.2126271	73.4148287
HLA-DRB1	major histoc	1.56562229	0.00020583	0.00229883	14.2064582	12.3654647
HLA-DRB5	major histoc	1.55753183	0.00229757	0.01506702	5.75071018	7.28825405
HLA-DRB6	major histoc	1.5875607	0.00396393	0.02279244	4.36813526	4.4835046
HMCN1	hemicentin 1	-2.9995249	1.43E-15	4.08E-13	28.1323941	30.4837368
HMGB2	high mobility	-2.0766447	9.41E-13	1.47E-10	144.168501	141.281576
HMHA1	histocompati	-1.6079796	0.00094982	0.00764455	13.184555	13.2457876
HMMR	hyaluronan-r	-1.8066081	0.00014478	0.00173965	70.511321	68.3376181
HMOX1	heme oxyger	6.61764692	3.26E-09	2.15E-07	7.91474049	7.77959702
HMOX2	heme oxyger	1.59086713	0.00031968	0.00323103	57.0662808	51.8366833
HOXA13	homeobox A	-3.6430493	0.00146624	0.01070075	2.58481398	2.08820762
HOXB5	homeobox B!	-1.8510915	0.00061299	0.00544974	11.7418682	11.4851419
HOXB8	homeobox B	-1.7557507	2.42E-06	5.87E-05	22.5620197	24.7309295
HOXB9	homeobox B!	-1.5422692	0.00026619	0.00280584	11.7619055	12.3449921
HPGD	hydroxyprost	2.3864428	0.02977982	0.09992752	0.48089562	0.22519886
HPN-AS1	HPN antisens	-2.3055227	8.54E-05	0.00112959	2.90541107	3.54176391
HRAS	Harvey rat sa	1.67705045	0.00013895	0.00168235	37.4497468	35.2333855
HS3ST1	heparan sulf	-1.5378274	0.00827135	0.03976804	7.79451659	8.00479588
HS3ST3A1	heparan sulf	-1.6539357	7.11E-05	0.00097138	31.6789993	31.937293
HSD17B11	hydroxystero	-1.5960517	1.52E-05	0.00027751	39.9544115	40.1672878
HSD17B7P2	hydroxystero	4.11646202	0.00060859	0.00542746	0.14026122	0.24567148
HSPA4L	heat shock 7	1.73553538	2.35E-06	5.76E-05	10.9804501	12.5292457
HSPB8	heat shock 2	2.17257463	3.81E-08	1.76E-06	11.2810099	10.7481275
HSPBAP1	HSPB (heat s	1.50078316	0.002302	0.01507589	6.83272534	6.18273237
HTR1D	5-hydroxytryı	-2.0529477	0.03337314	0.10836197	1.64306005	1.12599431
HYPK	huntingtin in	1.9082786	1.19E-06	3.29E-05	109.243456	94.6244669
ICAM1	intercellular	-1.901917	5.13E-19	2.44E-16	154.607943	158.437635
ICAM4	intercellular	-1.6422387	0.02871026	0.09753571	2.46459008	2.74333158
ICAM5	intercellular	-2.0110727	3.96E-07	1.31E-05	31.7791859	29.869558
ICOSLG	inducible T-c	-1.7342286	0.0068167	0.03437538	3.10578424	2.80474945
ID2	inhibitor of D	6.42377062	4.87E-13	8.07E-11	1.5829481	1.24883005
ID3	inhibitor of D	1.71929542	7.46E-07	2.23E-05	46.7470622	45.5925331
IDH2	isocitrate de	-1.5505907	0.00192821	0.01317948	18.1137352	16.3371537
IER5	immediate e	1.69948611	2.90E-08	1.41E-06	78.1255017	81.1330079
IFI27L2	interferon; al	1.59636027	0.00287216	0.01788168	12.2428011	11.751286
IFI44L	interferon-in	-3.4051916	0.00011191	0.00140881	2.38444081	1.67875515
IFITM1	interferon in	-3.9846498	5.20E-08	2.29E-06	3.18593351	2.12915287
IFNGR2	interferon ga	-1.5989989	3.04E-08	1.45E-06	66.7242679	69.2384135
IFT140	intraflagellar	-1.5708535	4.35E-05	0.00065633	47.8491147	48.2539742
IFT80	intraflagellar	-1.5341662	0.00053334	0.00489734	41.1766879	41.7641524

IGF2BP3	insulin-like g	-1.6632799	0.00035829	0.0035434	268.279647	264.301573
IGFBP3	insulin-like g	-2.7908989	2.73E-08	1.35E-06	376.982095	376.675804
IGFBP5	insulin-like g	-3.2821495	0.00082917	0.00685416	358.808248	368.159193
IGFBP7	insulin-like g	-1.8722064	1.74E-07	6.52E-06	65.3016184	62.4824477
IGFN1	immunoglob	1.83014498	0.0001217	0.00150295	5.91100872	5.13862856
IL11	interleukin 1	2.10832977	9.35E-09	5.24E-07	28.4329538	31.8554025
IL12A	interleukin 1	1.59150163	0.03921043	0.1216078	1.86347055	1.94489926
IL12RB1	interleukin 1	-1.5697605	0.04415463	0.13194542	2.28425422	2.067735
IL16	interleukin 1	2.23811614	0.03555552	0.11348301	0.40074635	0.30708936
IL17RD	interleukin 1	-4.5726746	1.55E-14	3.50E-12	58.3286318	60.2099864
IL18	interleukin 1	-1.5533665	0.00318684	0.01939184	8.09507635	8.90559133
IL18R1	interleukin 1	-1.8624004	2.43E-08	1.21E-06	22.4618331	20.8616036
IL1RL1	interleukin 1	1.7266223	1.77E-06	4.57E-05	33.9832908	34.33259
IL22RA1	interleukin 2	-1.8250918	0.00109844	0.00853071	3.86720232	3.31656505
IL23A	interleukin 2	3.6515234	0.00145955	0.01067962	0.22041049	0.24567148
IL24	interleukin 2	13.0573613	1.18E-14	2.96E-12	15.9296676	16.2347906
ILDR2	immunoglob	2.52954269	0.00083173	0.00687129	0.72134344	0.45039772
ILVBL	ilvB (bacteria	-1.5833721	1.33E-07	5.15E-06	81.4116218	80.6007197
IMPA2	inositol(myo	-1.5980874	2.32E-05	0.00039222	29.1943719	30.5656272
INPP5D	inositol polyp	2.2620325	4.38E-09	2.75E-07	4.60858307	4.81106658
INPP5J	inositol polyp	1.89217844	0.00131415	0.00987534	1.84343323	2.74333158
IPO5	importin 5	-1.5086967	1.96E-05	0.00034264	376.401013	385.356197
IQCG	IQ motif con	1.51105137	0.01252797	0.0538408	4.04753818	3.58270916
IQGAP2	IQ motif con	-3.0709705	1.65E-09	1.24E-07	7.31362096	5.99847876
IQGAP3	IQ motif con	-1.8227902	0.00014093	0.00170045	100.54726	100.602473
IQSEC1	IQ motif and	-1.5872698	0.00021119	0.00234402	51.0751228	55.7060092
IRAK2	interleukin-1	-1.5412899	4.55E-05	0.00067634	16.7511976	17.3198397
IRF2BPL	interferon re	-1.7294986	3.89E-07	1.29E-05	113.070584	112.98841
IRF5	interferon re	1.55116814	0.03395839	0.10978698	2.2642169	1.43308366
ISCU	iron-sulfur cl	1.73458584	2.13E-08	1.08E-06	53.4395263	48.151611
ISLR	immunoglob	-2.5298413	0.000979	0.00782927	12.0424279	12.9386982
ISPD	isoprenoid sy	1.6207066	0.03245854	0.10611761	1.46272419	1.71970039
ITGA1	integrin; alph	-1.7152643	0.00936572	0.04325043	363.416831	380.504185
ITGA10	integrin; alph	-3.5345483	1.38E-14	3.25E-12	11.5815696	10.2772571
ITGA4	integrin; alph	-1.6899424	0.00019498	0.00220007	55.4232208	58.2036693
ITGA5	integrin; alph	-1.6282075	4.98E-09	3.10E-07	358.828285	368.118248
ITGA6	integrin; alph	-2.0269148	2.32E-05	0.00039227	558.92094	585.68082
ITGA9	integrin; alph	-15.545851	1.70E-20	1.10E-17	3.58667987	3.21420193
ITGAX	integrin; alph	1.82194804	0.01036068	0.04662144	1.18220174	1.51497416
ITGB3	integrin; beta	-3.0498763	2.91E-13	5.05E-11	198.149035	208.001857
ITGB4	integrin; beta	-2.6812146	3.83E-05	0.00059519	1062.9797	1072.19225
ITGB5	integrin; beta	-1.7834665	2.84E-07	9.84E-06	356.363695	349.303906
ITGB7	integrin; beta	1.90460177	4.37E-05	0.00065802	7.37373291	6.44887648

ITPKA	inositol-trisp	-2.4918168	0.03882538	0.12083436	2.18406763	2.76380421
ITPKC	inositol-trisp	1.51327227	0.00051797	0.00478396	16.5107498	17.9954363
ITPRIPL1	inositol 1;4;5	-1.5553686	0.00785061	0.03835414	8.65612125	9.19220806
JAM2	junctional ad	-1.8805421	0.00666186	0.03384603	2.74511252	3.05042094
JOSD2	Josephin don	1.52079608	0.0033338	0.02002949	26.0685503	26.2459036
JUN	jun proto-onc	1.86015623	0.00057337	0.00517816	38.7922471	35.3971665
KANK1	KN motif anc	-1.503402	0.00040565	0.00393091	51.9768021	52.1233001
KANK2	KN motif anc	-1.7083013	5.85E-05	0.00083336	159.917833	156.738407
KANK3	KN motif anc	2.47823912	0.00277662	0.01740857	0.70130612	1.67875515
KBTBD8	kelch repeat	1.97045377	0.00019394	0.00219178	3.82712768	4.74964871
KCCAT211	NA	-5.033805	0.00021075	0.00234087	2.38444081	2.45671485
KCND1	potassium ch	-2.1396292	2.25E-06	5.53E-05	15.2083241	15.8458108
KCNF1	potassium ch	3.17704144	0.00269795	0.01705031	0.22041049	0.40945247
KCNH3	potassium ch	-2.3439977	5.19E-05	0.00075249	5.28985187	4.77012133
KCNH8	potassium ch	2.21675914	0.00022198	0.00243337	1.42264956	1.45355629
KCNIP3	Kv channel ir	-1.6953697	0.00325389	0.01968215	4.44828453	3.76696277
KCNJ2	potassium ch	-2.2890628	8.04E-09	4.58E-07	9.91847226	10.9528537
KCNK5	potassium ch	-2.0881246	1.78E-09	1.32E-07	13.6053387	12.9796435
KCNMA1	potassium ch	-1.5355168	0.00122402	0.00936316	26.8900804	29.7262497
KCNN3	potassium ch	-1.6175292	0.00636087	0.03269158	5.39003846	5.56855366
KDELC2	KDEL (Lys-As	-1.6321103	6.76E-05	0.00093279	45.5848978	47.2508156
KDR	kinase insert	4.35517224	1.07E-08	5.88E-07	1.98369445	1.84253614
KHDRBS3	KH domain co	1.80286585	8.26E-07	2.43E-05	12.6235102	12.3449921
KHK	ketohexokina	1.64546073	2.47E-05	0.00041027	7.03309851	7.22683618
KIAA0101	KIAA0101	-1.8473021	3.83E-10	3.32E-08	77.3640836	82.0338033
KIAA1024	KIAA1024	1.51183948	0.03820291	0.11934012	2.92544838	3.00947569
KIAA1324	KIAA1324	4.57514106	1.08E-08	5.94E-07	0.92171661	0.96221332
KIAA1467	NA	-1.5936157	0.00010072	0.0012943	17.7330262	17.6064564
KIAA1522	KIAA1522	-1.7716291	7.83E-07	2.33E-05	66.1832604	69.9549553
KIAA1755	KIAA1755	-6.7168935	3.49E-23	3.31E-20	29.95579	29.0097078
KIF11	kinesin famil	-1.8423458	8.40E-08	3.49E-06	147.23421	152.009231
KIF14	kinesin famil	-1.6946316	0.00464845	0.02567639	73.6371425	77.9392786
KIF15	kinesin famil	-2.0481766	4.59E-06	0.00010149	39.9944861	41.1499737
KIF18B	kinesin famil	-1.5278595	0.00045775	0.00433166	34.6645596	34.0459733
KIF20A	kinesin famil	-1.7129839	0.00039768	0.00387117	92.3920719	100.02924
KIF21B	kinesin famil	-2.0762776	1.67E-08	8.72E-07	44.903629	49.9941472
KIF22	kinesin famil	-1.5435698	1.58E-05	0.00028599	78.0453524	75.195947
KIF2C	kinesin famil	-1.6941574	5.51E-07	1.71E-05	97.9424089	103.345805
KIF4A	kinesin famil	-1.7014708	1.01E-06	2.85E-05	75.7811355	84.6747718
KIFC1	kinesin famil	-1.560475	0.00015452	0.00183201	46.2260919	44.5689019
KIRREL	kin of IRRE li	-1.91231	1.74E-07	6.52E-06	254.093226	265.857492
KITLG	KIT ligand	3.66757477	2.79E-08	1.37E-06	2.62488862	2.53860534
KLF11	Kruppel-like	-1.8344318	0.00020839	0.00232199	7.73440463	8.31188524

KLF13	Kruppel-like	-1.5784067	7.23E-05	0.00098593	22.5219451	23.7072983
KLHL18	kelch-like far	1.5037199	8.92E-06	0.00017652	27.310864	30.6065725
KLHL21	kelch-like far	1.6073607	8.51E-07	2.48E-05	138.457865	135.733495
KLHL4	kelch-like far	-1.6497732	0.04787006	0.1404563	2.44455276	2.72285896
KLK6	kallikrein-rela	-2.0434263	0.00036428	0.00359763	5.550337	4.36066886
KLLN	killin; p53-reg	2.46803016	4.99E-07	1.60E-05	2.32432885	2.82522208
KLRC2	killer cell lec	-6.3703122	2.05E-07	7.39E-06	3.76701573	4.29925099
KLRG1	killer cell lec	1.53842403	0.04041561	0.12431748	2.30429154	2.37482435
KNTC1	kinetochore a	-1.8729428	2.16E-09	1.49E-07	56.004303	63.1580442
KREMEN1	kringle conta	-2.2972192	0.00016186	0.00189845	7.05313583	7.69770653
KRT17	keratin 17; ty	6.67909794	8.30E-05	0.00110395	0.16029854	0.26614411
KRT6A	keratin 6A; ty	12.4989112	1.83E-10	1.74E-08	0.12022391	0.12283574
KRTAP2-3	keratin assoc	7.31762582	2.02E-09	1.43E-07	0.44082099	0.32756198
KSR1	kinase suppre	1.50758602	0.03519672	0.11276718	3.1658962	2.70238633
LACC1	laccase (mul	1.69250872	0.00198245	0.01342132	5.79078482	4.52444985
LAGE3	L antigen far	1.5167036	0.0032451	0.01966239	20.7786985	20.8820762
LAMA5	laminin; alph	-1.7299023	0.00563263	0.02971884	800.751327	827.032581
LAMB1	laminin; beta	-1.9059726	3.51E-06	8.07E-05	1119.76546	1150.92996
LAMC1	laminin; gam	-1.5261479	0.0003455	0.00343839	990.384502	1007.92868
LAMC3	laminin; gam	2.5748785	0.00546434	0.02914384	0.44082099	0.40945247
LAMTOR4	late endoson	1.66017761	5.07E-05	0.00074023	38.5718366	37.2806478
LANCL3	LanC lantibio	2.30399326	0.00358319	0.02109216	0.84156734	0.73701445
LARGE	like-glycosylt	1.74616741	0.00837549	0.04013422	1.3625376	1.53544678
LAT2	linker for act	1.51427494	0.02307559	0.08346452	3.74697841	3.25514718
LCAT	lecithin-chole	1.80715536	0.00424818	0.02399558	1.9235825	1.71970039
LCN2	lipocalin 2	-4.067674	2.53E-10	2.32E-08	16.7511976	15.5387214
LCP1	lymphocyte c	6.12739322	4.99E-06	0.00010816	0.3406344	0.24567148
LDB2	LIM domain l	-2.6373019	0.00142878	0.01052746	4.88910552	4.13547
LDHC	lactate dehyc	1.58153783	0.03727174	0.11715033	2.24417958	1.76064564
LDLR	low density l	-1.881822	5.26E-12	7.07E-10	192.738959	198.482087
LEF1	lymphoid enh	-1.6498319	1.11E-05	0.00021181	18.6347055	19.4489926
LFNG	LFNG O-fuco	-2.1085568	3.87E-06	8.77E-05	19.8770192	18.0159089
LGI4	leucine-rich r	-3.225959	0.00022077	0.00242577	1.42264956	1.82206351
LGR6	leucine-rich r	-2.1315023	0.02117683	0.07864702	39.6939264	36.7483596
LHX8	LIM homeobo	2.01219605	0.04399926	0.13164676	0.44082099	0.88032282
LIG4	ligase IV; DN	1.59746485	9.50E-05	0.00123332	30.5168349	30.2994831
LIMA1	LIM domain a	1.59095604	4.20E-06	9.45E-05	84.7778912	90.2023802
LIMCH1	LIM and calp	-1.5988823	2.37E-07	8.34E-06	100.046327	103.120606
LIN37	lin-37 DREAM	1.59195224	0.0028893	0.01795698	8.25537489	6.20320499
LINC00239	long interger	1.81778644	0.03210746	0.10542937	0.72134344	0.75748708
LINC00326	long interger	-2.509332	4.46E-08	2.00E-06	10.6798903	8.59850197
LINC00341	long interger	-1.6689171	0.00352277	0.02081385	5.69059823	6.44887648
LINC00342	long interger	-1.8058608	0.00068164	0.00589086	4.46832185	5.03626544

LINC00461	long interger	-1.5175033	0.02448429	0.08700195	7.57410609	7.71817915
LINC00473	long interger	1.69009259	0.0001556	0.00183869	6.39190435	6.91974683
LINC00491	long interger	1.52809845	0.0389988	0.12124168	2.92544838	1.94489926
LINC00552	long interger	2.10129164	0.0007622	0.00641058	1.74324664	1.69922777
LINC00648	long interger	-1.6068695	0.02563606	0.09011827	5.39003846	5.56855366
LINC00662	long interger	1.69486143	0.00671469	0.03401744	1.98369445	1.90395401
LINC00669	long interger	-2.7610446	0.03525776	0.11293737	1.54287346	1.47402891
LINC00685	long interger	1.76357949	0.00827936	0.03978054	1.64306005	1.2897753
LINC00701	long interger	2.2720375	0.04071907	0.12490045	0.48089562	0.67559658
LINC00707	long interger	2.40333778	1.34E-12	2.01E-10	13.2847416	14.6993438
LINC00853	long interger	2.74194837	0.03158291	0.10421117	0.22041049	0.32756198
LINC00941	long interger	1.51486981	0.00232434	0.01518697	8.57597198	8.55755672
LINC00993	long interger	-2.2238848	0.00507248	0.02749636	1.52283615	2.04726237
LINC01001	long interger	2.43746277	0.04646813	0.13719025	0.9016793	0.73701445
LINC01021	long interger	3.1353165	1.49E-06	3.94E-05	1.00186589	1.41261104
LINC01024	long interger	1.60582013	0.03321145	0.10806926	1.32246297	1.57639203
LINC01089	long interger	-1.5575434	0.02389096	0.08571754	5.10951601	5.81422514
LINC01106	long interger	-2.2359102	0.00554429	0.02940276	1.78332128	2.14962549
LINC01119	long interger	2.16769438	0.04169095	0.12697965	0.60111953	0.55276084
LINC01123	long interger	-1.7974422	0.02637461	0.09213962	4.8690682	4.19688787
LINC01134	long interger	2.35151409	0.00874136	0.04122388	0.26048513	0.94174069
LINC01239	long interger	-3.4890713	0.00010732	0.00136317	20.7586611	21.762399
LINC01468	long interger	2.49478177	4.33E-13	7.35E-11	12.6635848	11.7717587
LINC01606	long interger	1.87702066	0.02835779	0.09684674	1.00186589	0.67559658
LIPT1	lipoyltransfe	1.77982135	0.00196365	0.01333933	3.76701573	2.98900307
LLPH	LLP homolog	1.52765854	4.43E-05	0.00066299	39.5737025	36.9735585
LMNB1	lamin B1	-1.8327075	6.39E-07	1.95E-05	140.842306	142.530407
LMNB2	lamin B2	-1.5128594	0.00010337	0.00132123	225.119264	215.453892
LMO2	LIM domain	1.5312723	0.00820615	0.0396024	3.86720232	4.21736049
LNX1	ligand of nur	-1.5812146	0.01065301	0.04760618	6.15145653	5.75280727
LOC100129	NA	-1.8000435	1.16E-06	3.22E-05	43.1804196	42.4192764
LOC100133	NA	2.07110047	0.04837658	0.14159266	0.66123148	0.96221332
LOC100272	NA	1.75762775	0.018107	0.0701394	1.0219032	1.2897753
LOC100288	NA	-1.936516	1.06E-05	0.00020502	8.77634515	10.0725309
LOC100506	NA	-2.2694557	0.00695107	0.03484606	2.64492594	2.14962549
LOC100507	NA	-2.7863214	7.52E-06	0.00015315	3.6267545	2.82522208
LOC100507	NA	1.63068253	2.41E-06	5.87E-05	32.3201935	30.5656272
LOC100652	NA	1.6960151	0.03934279	0.12188568	1.86347055	1.47402891
LOC101927	NA	1.58202294	0.00260938	0.01663815	7.3536956	7.92290539
LOC101927	NA	-3.6662279	0.03544576	0.11335179	1.28238833	1.12599431
LOC101928	NA	-2.5457284	0.00637418	0.03274639	1.08201516	1.2078848
LOC101928	NA	5.25995845	3.92E-05	0.00060531	0.30055977	0.71654183
LOC101929	NA	7.95967907	0.00044113	0.00419387	0.08014927	0.02047262

LOC1019304!	NA	1.92723049	2.25E-08	1.13E-06	11.160786	11.4032514
LOC102606(	NA	1.91221723	0.00334432	0.02007577	1.54287346	2.04726237
LOC1027237(	NA	3.66275972	1.69E-05	0.00030408	0.54100758	0.53228822
LOC1027238:	NA	-2.4088756	1.01E-06	2.87E-05	8.69619588	8.41424836
LOC115110	NA	3.19365223	0.0055896	0.02954653	0.30055977	0.36850723
LOC152225	NA	-1.8609165	0.00575907	0.03021813	3.2861201	3.52129128
LOC344887	NA	4.73725397	0.00222693	0.01470621	0.14026122	0.16378099
LOC374443	NA	1.59718804	0.02973539	0.09982317	1.2423137	1.82206351
LOC388242	NA	-2.3945587	0.00402363	0.02302839	1.72320932	1.45355629
LOC440300	NA	-1.5016605	0.04549978	0.13492127	6.15145653	5.91658826
LOC440434	NA	-1.6242578	4.60E-06	0.00010153	18.5745935	19.3466294
LOC613038	NA	-2.3945598	0.00402921	0.02304187	1.72320932	1.45355629
LOC645166	NA	1.85670535	4.08E-07	1.34E-05	16.2903393	17.1151134
LOC650226	NA	1.76650174	0.03899734	0.12124168	2.20410495	3.13231143
LOC654342	NA	2.21611056	0.00026096	0.00276453	5.61044896	5.81422514
LOC728485	NA	2.03762047	0.04667151	0.13759115	0.44082099	0.53228822
LOC728743	NA	-1.6707945	0.01913018	0.07305044	5.24977724	4.99532019
LOC730101	NA	-1.86788	0.0002738	0.00286065	14.4268687	15.2725773
LONRF1	LON peptida:	-1.5947565	0.0019844	0.01342812	9.15705419	9.00795445
LOXL2	lysyl oxidase-	-1.849486	2.80E-08	1.37E-06	309.696782	308.829529
LOXL3	lysyl oxidase-	-3.0695374	4.14E-23	3.50E-20	146.392643	140.565035
LOXL4	lysyl oxidase-	-2.2165225	1.14E-10	1.16E-08	540.947466	536.382742
LPAR4	lysophosphat	-1.8340003	0.01913713	0.07305741	1.40261224	1.49450153
LPIN2	lipin 2	-1.5493836	0.00066956	0.00581114	55.7638552	61.0288914
LRFN1	leucine rich r	-1.561979	0.0006756	0.00585291	11.8620921	13.5119317
LRIG1	leucine-rich r	-1.5124397	0.00510959	0.02766595	10.0186589	9.84733202
LRIG3	leucine-rich r	-1.6499945	6.41E-05	0.00089038	69.8901641	74.0904253
LRP1B	low density l	-1.7190052	0.01477549	0.06061184	4.62862039	6.039424
LRP5	low density l	-1.7152104	4.24E-06	9.51E-05	117.378607	118.659327
LRRC17	leucine rich r	-2.9091309	3.27E-07	1.11E-05	9.0568676	9.21268068
LRRC46	leucine rich r	1.97200991	0.0103094	0.04649879	1.30242565	1.61733728
LRRC4B	leucine rich r	-1.6914069	8.03E-05	0.00107455	52.6781082	46.9846715
LSAMP	limbic syster	-1.6229735	0.04158818	0.1267479	3.34623206	3.43940079
LSMEM1	leucine-rich :	1.84784965	0.02984307	0.10003827	0.52097026	0.94174069
LSMEM2	leucine-rich :	3.22401254	0.00701132	0.0350345	0.24044781	0.4299251
LTB	lymphotoxin	-2.508636	0.00152204	0.01101601	2.86533643	2.94805782
LTBP2	latent transf	-1.7599669	0.00109794	0.00853071	35.3458284	35.7861463
LTBP3	latent transf	-1.5209835	0.00028068	0.00292393	632.197411	626.871739
LUCAT1	lung cancer a	-2.477541	0.00259056	0.01654035	2.56477667	2.80474945
LVRN	laeverin	3.82316483	0.00101933	0.00807025	0.44082099	0.59370609
LYNX1	Ly6/neurotox	1.68673522	0.01182287	0.05149362	1.56291078	1.41261104
LYPD3	LY6/PLAUR c	2.04477661	2.42E-13	4.26E-11	14.0060851	14.3308366
LYPD6	LY6/PLAUR c	-1.6608045	0.0224212	0.08184543	2.18406763	2.45671485

LZTS1	leucine zippe	-2.5562568	4.02E-10	3.45E-08	15.0279883	14.2284735
MAB21L1	mab-21-like	2.61964582	5.37E-13	8.80E-11	4.94921747	5.2614643
MAF	v-maf avian	1.90175503	3.10E-10	2.76E-08	11.100674	12.5087731
MAFB	v-maf avian	2.37576295	0.00484037	0.0265104	1.54287346	1.26930267
MAGEA2	melanoma a	1.54688097	0.00017911	0.00205014	35.886836	41.7846251
MAGEB2	melanoma a	2.94263242	0.00074331	0.00628656	0.96179125	0.38897985
MAGEC1	melanoma a	2.00821442	0.0121402	0.05252278	0.96179125	0.83937757
MAGEH1	melanoma a	1.77164681	0.00016131	0.00189354	4.16776208	4.42208673
MAGIX	MAGI family	1.9662455	0.00051772	0.00478396	2.30429154	2.19057074
MALAT1	metastasis a	2.17222618	0.00445856	0.02491724	565.052359	491.363442
MALT1	MALT1 parac	-1.5756884	0.00031187	0.00316785	121.085511	130.512976
MAML2	mastermind-	-1.9608438	3.08E-05	0.00049343	51.4157572	56.422551
MAN1A1	mannosidase	-1.9516892	1.88E-05	0.00033097	24.2050798	25.6931428
MANSC1	MANSC dom	-1.5604531	9.56E-05	0.00123687	21.1193329	21.9671253
MAP1LC3B2	microtubule-	2.22948472	0.00776811	0.03810787	2.02376909	1.26930267
MAP2	microtubule-	-4.3392945	7.99E-11	8.56E-09	3.76701573	4.89295707
MAP2K3	mitogen-acti	1.68465259	7.29E-06	0.0001491	51.7964663	53.4130753
MAP3K1	mitogen-acti	-1.8761443	2.72E-05	0.00044481	29.454857	29.9514485
MAP3K6	mitogen-acti	-1.7825784	0.00021683	0.002388	15.5890332	16.7466062
MAP7	microtubule-	2.22738401	1.26E-06	3.43E-05	3.42638133	2.45671485
MAPK3	mitogen-acti	-1.5898034	8.13E-06	0.00016356	56.885945	57.9375252
MAPK4	mitogen-acti	3.56504426	0.01449344	0.05979908	0.3406344	0.30708936
3-Mar	membrane-a	2.91597443	0.00473239	0.02604914	0.42078367	0.49134297
4-Mar	membrane-a	1.57322837	8.34E-06	0.00016578	11.2810099	12.897753
MARCKS	myristoylate	-2.0580245	1.37E-16	4.64E-14	106.017448	105.085978
MAST1	microtubule	-2.0091459	0.00199745	0.01349724	3.30615742	2.98900307
MAST4	microtubule	1.76365403	1.11E-07	4.45E-06	13.3849282	14.0032746
MATN2	matrilin 2	1.95369943	0.04941203	0.14391394	0.5610449	0.38897985
MBOAT1	membrane b	-2.0289608	0.00087308	0.00716305	6.91287461	6.34651336
MCF2	MCF.2 cell lir	2.17505771	0.00185396	0.01286309	0.82153003	0.81890495
MCF2L	MCF.2 cell lir	1.57619539	4.30E-07	1.41E-05	25.8681772	27.4537884
MCM2	minichromos	-1.7476655	5.22E-09	3.22E-07	183.84239	175.450385
MCM5	minichromos	-1.8498032	1.05E-08	5.84E-07	74.1981874	71.4699295
MCM6	minichromos	-1.7221138	7.97E-07	2.37E-05	69.2890446	73.9675896
MCTP2	multiple C2 c	1.57738365	0.02862404	0.09740239	1.5829481	1.69922777
MDK	midkine (neu	-2.0982747	6.81E-09	4.03E-07	97.4214387	93.6827263
MDM2	MDM2 proto	4.97760801	5.63E-18	2.23E-15	99.6455809	100.11113
MED6	mediator cor	1.50234267	0.00040575	0.00393091	21.1794448	21.6600359
MEGF11	multiple EGF	2.30470421	0.00323098	0.01961468	0.74138075	0.61417871
MELK	maternal em	-1.7258701	4.03E-08	1.85E-06	54.2209817	52.3689715
MEOX2	mesenchyme	-2.7109114	8.38E-14	1.59E-11	66.4036709	65.2871971
METTL21B	methyltransf	-2.0081372	2.07E-05	0.00035622	9.31735273	11.4441967
METTL7A	methyltransf	-2.1669012	3.34E-06	7.74E-05	6.87279997	6.16225975

MEX3A	mex-3 RNA b	-2.0149152	3.49E-10	3.07E-08	38.8723963	39.3688555
MFAP2	microfibrillar	-1.740972	9.67E-06	0.00018831	81.7522562	80.2526851
MFSD7	major facilita	1.84652035	0.04466595	0.13305488	0.80149271	0.53228822
MGAT3	mannosyl (be	-2.1689238	0.0048535	0.02656027	2.2642169	1.9858445
MGAT4A	mannosyl (al	2.1812199	0.0085756	0.04068272	0.44082099	0.96221332
MGAT5B	mannosyl (al	-1.6067382	1.40E-05	0.00025813	19.676646	18.8962317
MGLL	monoglycerid	-1.5692963	7.36E-08	3.10E-06	53.2191158	50.8335248
MGP	matrix Gla pr	-5.3226483	1.52E-05	0.00027648	2.24417958	1.2078848
MICA	MHC class I p	1.91230694	0.0002631	0.00278145	9.03683028	8.70086509
MICAL3	microtubule	-1.5510322	3.84E-05	0.00059553	129.300811	131.86417
MINOS1-NBI	MINOS1-NBI	609.71218	6.50E-06	0.00013549	0	0
MIR210HG	MIR210 host	-3.0982621	8.96E-06	0.00017711	12.0223906	12.7134993
MIR22HG	MIR22 host g	1.97112992	1.23E-08	6.61E-07	13.5251894	13.5938222
MIR663A	microRNA 66	3.21258015	0.00225657	0.01486641	0.88164198	0.4299251
MIRLET7BHG	MIRLET7B ho	-1.7037813	0.0131097	0.05565276	5.39003846	5.20004643
MIS18BP1	MIS18 bindir	-1.5700998	0.00058712	0.00526229	38.5317619	39.1641292
MISP	mitotic spinc	1.52937973	9.19E-06	0.00018115	24.8462739	27.8837135
MKI67	marker of pro	-2.080345	1.30E-05	0.00024167	345.844104	350.102339
MLH3	mutL homolo	2.28414032	0.0029408	0.01819535	0.98182857	0.55276084
MLPH	melanophilin	-1.9166731	4.36E-06	9.69E-05	23.523811	22.5608314
MMD	monocyte to	-1.6386641	6.34E-05	0.00088526	21.9809375	23.1545375
MME	membrane m	-1.5236018	0.00663303	0.03372859	7.61418073	7.53392554
MMP1	matrix metal	3.65445537	1.42E-09	1.07E-07	6.91287461	7.39061717
MMP10	matrix metal	5.18799069	5.66E-07	1.75E-05	0.54100758	0.26614411
MMP11	matrix metal	-1.7959081	0.03170058	0.10453064	4.68873234	3.33703767
MMP2	matrix metal	-1.9190431	2.54E-06	6.12E-05	725.771684	737.976668
MMP3	matrix metal	2.38713691	0.03076641	0.10208432	0.64119417	0.88032282
MMP9	matrix metal	-5.0031458	1.02E-38	7.30E-35	42.9600091	43.4838528
MMRN2	multimerin 2	-2.2488393	0.01204254	0.0521974	0.98182857	1.49450153
MNS1	meiosis-spec	-1.6164252	0.00170781	0.01201879	7.63421804	6.77643846
MORN2	MORN repea	1.54960362	0.0003193	0.00322947	7.85462854	8.72133771
MOSPD1	motile sperm	1.97722463	1.33E-06	3.56E-05	10.6999277	9.58118791
MPV17L2	MPV17 mito	1.66848195	0.00030305	0.00310038	12.1025399	12.4883005
MRC2	mannose rec	-1.6160892	3.54E-05	0.00055704	134.39029	128.936584
MRGPRX4	MAS-related	3.57491093	8.04E-05	0.00107456	0.64119417	0.73701445
MRPL27	mitochondria	1.60069895	0.00035724	0.00354003	31.4986634	30.6679904
MRPL52	mitochondria	1.59388132	0.00662076	0.03370934	18.4744069	20.0426986
MSX2	msh homeob	1.71008343	0.00019056	0.00215702	6.47205362	5.20004643
MTRNR2L2	MT-RNR2-lik	1.92502498	0.00030018	0.00308206	3.10578424	3.60318178
MUC13	mucin 13; ce	2.53001548	0.00327743	0.01976585	0.78145539	0.53228822
MUC20	mucin 20; ce	2.15580373	0.00725265	0.03596294	0.58108221	0.71654183
MUC5AC	mucin 5AC; c	-2.4614511	0.005512	0.02934066	1.48276151	1.49450153
MUC5B	mucin 5B; oli	-1.7023404	0.00251279	0.01615963	13.5251894	12.2835742

MXD1	MAX dimeriz	2.07198399	1.46E-08	7.66E-07	8.87653174	9.76544153
MXD3	MAX dimeriz	-1.8370443	6.32E-06	0.00013229	11.1407486	11.2189978
MXI1	MAX interact	-1.6296819	1.13E-08	6.15E-07	41.877994	42.1326597
MYB	v-myb avian	-1.9307648	0.04433094	0.1322781	1.12208979	1.24883005
MYBL2	v-myb avian	-1.6654613	4.35E-08	1.97E-06	106.458269	97.941032
MYCN	v-myc avian	-4.9547703	0.00152788	0.01104147	0.86160466	1.41261104
MYEOV2	myeloma ove	1.56131464	0.00104287	0.00822006	19.7768326	16.4190442
MYH10	myosin; heav	-1.5770113	0.00067738	0.00586115	232.513035	236.131242
MYH14	myosin; heav	1.77332057	1.74E-08	9.00E-07	13.0442938	13.0410613
MYH16	myosin; heav	1.75735378	1.63E-05	0.00029326	7.4739195	5.56855366
MYH6	myosin; heav	5.89992982	1.65E-05	0.00029668	0.22041049	0.45039772
MYH7	myosin; heav	5.18032911	0.00115975	0.00893386	0.24044781	0.24567148
MYOCD	myocardin	7.33843742	2.28E-05	0.00038861	0.22041049	0.08189049
MYPN	myopalladin	1.8827174	0.00402802	0.02304187	1.703172	2.60002322
MYRF	myelin regul	-2.1213292	5.12E-11	5.79E-09	29.9958646	30.19712
MYRIP	myosin VIIA	2.60310222	0.01222708	0.05283447	0.60111953	0.38897985
MYT1	myelin trans	3.85164929	0.00248662	0.01602029	0.32059708	0.10236312
N4BP2L1	NEDD4 bindi	-1.8484098	0.04720713	0.13876831	1.72320932	1.49450153
N6AMT2	N-6 adenine-	1.50291048	0.00487687	0.02662847	5.16962797	5.3433548
NAALAD2	N-acetylated	-1.9245818	0.01441347	0.05955533	2.38444081	2.35435173
NABP1	nucleic acid	1.65148788	7.50E-05	0.00101222	16.2302273	18.4867792
NADSYN1	NAD synthet	1.54589469	2.84E-05	0.00046156	22.6020944	23.2978458
NAGA	N-acetylgala	-1.5276602	0.00032422	0.00326531	17.3322798	15.1906868
NALCN	sodium leak	-1.8318001	0.01808601	0.07009621	10.2591067	10.2158392
NANOS1	nanos homol	-1.7898747	1.28E-05	0.00024003	13.4250029	11.2189978
NAP1L2	nucleosome	2.50748108	7.99E-05	0.00107039	1.52283615	1.26930267
NAT1	N-acetyltrans	1.72422305	0.00827288	0.03976804	2.58481398	2.02678975
NAT8L	N-acetyltrans	-1.732509	5.33E-07	1.69E-05	39.2130307	35.4995296
NAV1	neuron navig	-1.6724285	3.55E-06	8.16E-05	55.4833327	56.9343666
NAV2	neuron navig	-1.7225575	0.00077667	0.00650692	7.51399414	7.73865178
NCAM2	neural cell a	-1.652788	0.00307421	0.01886763	9.99862153	11.0552168
NCAPD2	non-SMC cor	-1.8447153	5.61E-07	1.74E-05	337.488542	343.817243
NCAPG	non-SMC cor	-1.8201254	2.12E-07	7.59E-06	120.103682	130.901956
NCAPG2	non-SMC cor	-1.7709503	5.79E-09	3.54E-07	72.3347169	71.5927652
NCAPH	non-SMC cor	-1.5548268	6.35E-05	0.00088696	41.0364266	41.9074608
NCF2	neutrophil cy	2.93592556	0.00125588	0.00953814	0.58108221	0.38897985
NCOR2	nuclear rece	-1.6072613	0.00022586	0.00246456	237.983222	230.624106
NDC80	NDC80 kinet	-1.6591124	1.73E-05	0.00030779	34.5042611	32.8585611
NDRG1	N-myc down	-2.9929098	3.36E-19	1.65E-16	508.687384	511.733703
NDUFAF1	NADH dehyd	1.53100711	9.95E-05	0.00128075	26.9902669	24.7718747
NDUFS5	NADH dehyd	1.53566243	0.00017375	0.00200986	74.3384487	68.6856527
NDUFS6	NADH dehyd	1.62957548	0.0004096	0.00395474	48.8109059	44.4051209
NEAT1	nuclear para	1.65728328	0.00052482	0.00483157	185.084704	176.57638

NEBL	nebulette	-1.8717454	0.00052134	0.00481191	6.89283729	7.80006965
NEFL	neurofilamer	2.29407544	1.89E-25	2.36E-22	50.7545257	50.9358879
NEIL3	nei-like DNA	-1.9177235	1.36E-05	0.00025111	14.066197	15.2521047
NEK2	NIMA-relate	-1.9331434	4.92E-06	0.000107	47.0075473	48.6224814
NEMP1	nuclear enve	-1.5774464	3.73E-05	0.00058152	46.4264651	47.3122335
NES	nestin	-1.8832619	3.54E-11	4.20E-09	333.040257	337.9416
NET1	neuroepithel	-1.5330227	0.00017744	0.00203764	211.193329	213.48852
NETO1	neuropilin (N	-1.6215967	0.00091455	0.00743686	8.45574807	9.60166054
NEU1	sialidase 1 (l	1.55615512	0.00130996	0.00986174	23.9245573	23.1750101
NEURL1B	neuralized E3	-2.2363553	0.00407288	0.02321702	1.18220174	1.92442663
NEXN	nexilin (F act	1.90955349	1.54E-06	4.04E-05	8.09507635	7.88196014
NFASC	neurofascin	1.59489973	9.51E-05	0.00123332	10.5396291	9.66307841
NFATC1	nuclear facto	-2.4400453	1.25E-07	4.89E-06	8.63608393	8.33235786
NFATC2	nuclear facto	-1.8910779	6.60E-07	2.00E-05	12.4632116	14.2694187
NFATC4	nuclear facto	-2.1854769	1.24E-07	4.87E-06	13.6454134	15.4977762
NFE2	nuclear facto	-1.9977954	6.18E-05	0.00086952	8.3755988	8.04574113
NFE2L3	nuclear facto	-1.6659274	9.23E-10	7.35E-08	81.2112486	83.1802703
NFIX	nuclear facto	-1.9013268	1.95E-07	7.09E-06	168.934626	172.543273
NFKBIB	nuclear facto	1.59130205	0.0010864	0.00846729	20.7786985	21.004912
NGEF	neuronal gua	-3.1458868	2.13E-06	5.36E-05	7.09321047	6.59218485
NGFR	nerve growth	-1.8797276	4.57E-12	6.26E-10	85.1986749	84.4495729
NHLH1	nescient heli	2.97121964	0.00324189	0.0196513	0.30055977	0.20472624
NHLH2	nescient heli	4.20052829	9.98E-07	2.84E-05	0.58108221	0.65512396
NID1	nidogen 1	-2.5223467	2.11E-07	7.57E-06	259.904048	256.051105
NIPBL-AS1	NIPBL antise	-1.5033324	0.00136439	0.01018004	16.4506378	16.7466062
NKX3-1	NK3 homeob	1.63688683	0.0052275	0.02817592	7.1332851	6.5512396
NLRC4	NLR family; (	2.22457268	0.04311159	0.13011082	0.52097026	0.45039772
NLRP1	NLR family; p	1.80295764	1.05E-06	2.94E-05	11.7218309	13.2048423
NME1	NME/NM23	1.52803887	0.00069363	0.00596943	209.129485	200.324623
NME1-NME2	NME1-NME2	1.76659494	0.00164553	0.01166603	4.5284338	6.38745861
NMNAT2	nicotinamide	-1.8002349	1.06E-10	1.08E-08	48.4301969	52.6965335
NMT2	N-myristoylt	-1.5218758	1.02E-06	2.87E-05	31.9595217	36.3593798
NMU	neuromedin	-1.7335828	0.01711966	0.06741883	3.6267545	3.86932589
NOCT	nocturnin	1.57533137	0.0005414	0.00494898	11.0004874	10.6867096
NOG	noggin	-1.6449115	0.00195205	0.01329125	8.45574807	7.84101489
NOL4L	nucleolar pro	-1.8059851	1.32E-07	5.13E-06	36.7885153	38.9798756
NOL6	nucleolar pro	1.52817078	1.30E-07	5.08E-06	139.219283	144.680032
NOTUM	notum pectir	-4.6612156	0.004856	0.02656027	1.16216443	1.59686465
NOV	nephroblasto	1.68523189	1.59E-06	4.15E-05	8.73627052	8.86464608
NOXO1	NADPH oxida	1.77590524	0.00217383	0.01442906	2.44455276	2.51813272
NPC2	Niemann-Pic	-1.5406423	1.58E-05	0.00028599	118.761182	121.423131
NPHP3-ACAD	NPHP3-ACAD	-1.6240384	0.00298842	0.01843635	7.85462854	5.97800613
NPHP4	nephronopht	-1.5647794	1.30E-05	0.00024226	21.9007882	20.67735

NPIPB6	nuclear pore	2.47719111	0.0198255	0.0749226	0.48089562	0.51181559
NPIPB9	nuclear pore	2.17320663	0.00077073	0.00646482	1.38257492	1.49450153
NPL	N-acetylneur	1.50249515	0.00226417	0.01489002	5.32992651	4.66775821
NPTX2	neuronal per	-1.9787043	0.0063459	0.03263636	2.68500057	3.84885326
NR1H3	nuclear recep	-1.5034719	0.00018076	0.00206415	29.0340733	29.112071
NR2F1	nuclear recep	-3.1481516	9.97E-10	7.81E-08	85.2988614	83.5078323
NR2F1-AS1	NR2F1 antise	-1.7718768	1.88E-05	0.00033097	9.17709151	9.60166054
NR2F2-AS1	NR2F2 antise	-2.2199106	0.03173172	0.10455693	1.04194052	0.71654183
NR4A1	nuclear recep	3.21757758	4.11E-16	1.27E-13	29.3145958	29.2349067
NR4A3	nuclear recep	4.12456532	6.27E-06	0.00013143	1.00186589	0.96221332
NRBP2	nuclear recep	-1.5394601	0.00971395	0.0444552	11.7819428	11.7103408
NREP	neuronal reg	-1.5208285	9.47E-06	0.00018572	64.5802749	66.0037389
NRG1	neuregulin 1	1.78991455	4.57E-05	0.00067732	4.92918015	5.05673806
NRG4	neuregulin 4	2.72281249	0.0068021	0.03432605	0.54100758	1.02363119
NRM	nurim (nucle	-2.6052206	2.07E-14	4.40E-12	15.9897795	16.7261336
NRN1	neuritin 1	-2.7155354	7.55E-14	1.45E-11	43.9218004	42.1736049
NRP1	neuropilin 1	-1.5426995	0.0055459	0.02940276	239.486021	246.285664
NRXN2	neurexin 2	3.50777686	0.01129148	0.04978801	0.26048513	0.18425361
NRXN3	neurexin 3	1.95578574	4.04E-05	0.00062129	2.56477667	2.21104336
NT5DC3	5'-nucleotida	-1.5338408	2.21E-06	5.45E-05	31.9394844	33.6774661
NTN1	netrin 1	3.04017103	2.23E-07	7.89E-06	2.36440349	1.61733728
NTNG1	netrin G1	1.60466706	0.02143876	0.07941869	2.22414226	1.96537188
NUAK2	NUAK family	1.5337301	0.00488755	0.02666631	7.85462854	7.24730881
NUCKS1	nuclear casei	-1.5170626	2.40E-06	5.85E-05	567.65721	561.011308
NUDT14	nudix (nucleo	1.71954849	0.01507211	0.0615981	2.04380641	2.39529698
NUF2	NUF2; NDC80	-1.5698237	1.70E-05	0.00030486	60.5327368	60.9265283
NUP210	nucleoporin 2	-2.4443175	1.06E-05	0.00020455	69.6697536	73.9880622
NUSAP1	nucleolar and	-2.0651489	3.64E-11	4.28E-09	88.0840486	91.7173544
NYNRIN	NYN domain	-4.0798063	4.07E-07	1.34E-05	8.75630783	7.77959702
OAS3	2'-5'-oligoad	-1.5502988	0.00025191	0.00269117	80.3897186	80.1093767
OIP5	Opa interacti	-1.5446719	9.69E-05	0.00125104	17.3122425	15.9891191
OLFML2A	olfactomedir	-2.4818097	1.28E-11	1.62E-09	11.3210845	12.1402659
OLFML3	olfactomedir	-2.145814	0.03930038	0.12183375	1.50279883	1.5559194
OLMALINC	oligodendroc	-2.4226629	6.39E-08	2.73E-06	17.6128023	16.5623526
ONECUT2	one cut home	1.65655122	0.02039465	0.07656589	2.62488862	1.92442663
ORAOV1	oral cancer o	1.68434527	2.68E-05	0.00043934	13.3448536	11.7308134
OSBPL5	oxysterol bin	-1.5532973	5.12E-05	0.00074631	15.007951	15.9481739
OSGIN1	oxidative stre	2.29148384	0.00017927	0.00205042	3.80709036	3.02994831
OSR2	odd-skipped	1.63312904	0.01262867	0.05412025	2.14399299	2.47718747
OTUB2	OTU deubiqu	1.55209674	0.00039207	0.00382432	7.11324778	8.06621376
OVCA2	ovarian tumo	1.50601526	0.00349361	0.02069306	32.7610144	33.9026649
OVGP1	oviductal gly	2.33465817	0.01144687	0.05027099	0.52097026	0.75748708
P4HA1	prolyl 4-hydr	-1.604577	1.17E-07	4.63E-06	142.425254	141.650084

P4HA2-AS1	P4HA2 antise	-1.6976323	0.03592701	0.1143869	4.08761281	3.74649015
PABPC4L	poly(A) bindi	-1.8226784	0.0027521	0.0173081	3.14585888	3.48034604
PADI2	peptidyl argi	-3.5947944	4.38E-05	0.00065802	2.08388104	1.86300876
PADI3	peptidyl argi	-3.1885428	7.52E-06	0.00015315	8.47578539	9.78591415
PADI4	peptidyl argi	2.81319385	0.00614952	0.03182744	0.3406344	0.24567148
PAG1	phosphoprot	1.70091217	0.01016689	0.04607196	5.02936674	4.68823084
PAGE1	P antigen far	2.38147339	0.00100983	0.00801736	1.26235102	1.2897753
PAICS	phosphoribos	-1.5640767	3.29E-07	1.11E-05	406.797624	407.85561
PALM	paralemmin	-2.5922959	3.89E-09	2.52E-07	24.8462739	25.1199093
PAQR8	progestin an	-1.9966844	6.58E-09	3.92E-07	17.3923918	17.7497648
PARD6A	par-6 family	1.85845382	0.00011395	0.00142819	6.69246411	6.91974683
PARM1	prostate and	3.46742819	3.00E-06	7.07E-05	0.86160466	1.10552168
PAX6	paired box 6	-1.9474087	0.01378911	0.05772874	1.86347055	1.84253614
PBK	PDZ binding	-1.8017696	3.09E-07	1.06E-05	56.9260196	54.9689948
PCDH18	protocadheri	-2.4215063	5.84E-06	0.0001241	9.01679297	9.86780464
PCDH7	protocadheri	1.79388617	0.00305116	0.01876653	5.7707475	5.42524529
PCDHA6	protocadheri	-1.539988	0.04043689	0.1243561	10.2591067	15.3954131
PCDHGA7	protocadheri	-1.6010803	0.03059621	0.10182777	12.5433609	12.5906636
PCDHGA9	protocadheri	-2.4378804	0.01164847	0.05095071	1.82339591	2.78427683
PCDHGB2	protocadheri	-2.0171157	0.00213784	0.01423659	6.73253875	4.52444985
PCDHGB5	protocadheri	-2.1293685	0.00109348	0.00850608	5.47018773	4.93390232
PCDHGB7	protocadheri	-3.865819	0.00426995	0.02410191	1.2423137	1.33072054
PCDHGC3	protocadheri	-1.7535573	1.12E-07	4.47E-06	130.062229	126.254671
PCK1	phosphoenol	7.82879702	0.00375256	0.02189021	0.10018659	0.10236312
PCLO	piccolo presy	1.52511843	8.18E-05	0.00109072	27.8719089	26.552993
PCOLCE	procollagen (	-2.5931911	5.69E-14	1.17E-11	110.325471	103.693839
PCSK1	proprotein co	-2.2345792	5.05E-07	1.61E-05	10.2390693	11.4851419
PCSK1N	proprotein co	2.04335995	0.00311665	0.01907876	1.9235825	1.49450153
PDCD1LG2	programmec	1.97786281	0.02473459	0.08768473	0.88164198	0.8598502
PDE3A	phosphodies	1.61964708	0.01053359	0.04725028	2.54473935	3.1937293
PDE4B	phosphodies	-2.5294664	0.00151471	0.01097969	12.3830623	13.4709864
PDE4C	phosphodies	4.71733807	5.01E-05	0.00073523	0.64119417	0.30708936
PDE5A	phosphodies	-1.5534284	0.04582926	0.13575969	2.58481398	2.9275852
PDE9A	phosphodies	-1.5831607	0.00195704	0.0133076	6.35182971	6.83785633
PDGFB	platelet-deri	-3.0056638	1.20E-06	3.29E-05	11.100674	8.82370083
PDGFD	platelet deri	2.38105764	8.35E-06	0.00016579	1.42264956	1.94489926
PDGFRA	platelet-deri	-2.1201015	0.00366239	0.02143902	2.58481398	3.72601752
PDGFRB	platelet-deri	-2.1552198	1.41E-08	7.46E-07	22.1211987	23.2773732
PDK1	pyruvate deh	-1.8149104	8.88E-08	3.64E-06	23.3034005	24.8742378
PDK3	pyruvate deh	-1.5531287	0.00224615	0.01481256	32.9413503	36.4207976
PDLIM1	PDZ and LIM	-1.5915315	1.73E-08	9.00E-07	109.203381	109.057667
PDRG1	p53 and DNA	1.68124991	0.00026742	0.00281051	21.7805643	22.8679207
PDX1	pancreatic ar	2.19483817	0.0038168	0.02212903	0.58108221	0.79843233

PDZD2	PDZ domain	-1.610397	0.00101025	0.00801736	34.8048208	34.4554258
PDZRN3	PDZ domain	1.55940053	0.00203756	0.01370976	5.81082213	5.13862856
PEAR1	platelet endo	1.57768534	0.00199848	0.01349777	4.04753818	4.52444985
PEPD	peptidase D	1.52921442	0.00054387	0.00496203	36.2875824	37.3625383
PER3	period circad	-2.0779851	0.00569439	0.02993391	2.36440349	2.72285896
PFKFB4	6-phosphofru	-2.0991051	5.61E-11	6.29E-09	172.922052	178.21419
PFKL	phosphofruct	-1.905334	3.23E-09	2.14E-07	113.371144	110.40886
PFKP	phosphofruct	-1.5166932	3.30E-06	7.65E-05	290.200472	282.419845
PGM1	phosphogluco	-1.5251575	5.64E-08	2.45E-06	163.885221	163.473901
PGM2L1	phosphogluco	1.74519089	0.00024054	0.00260085	5.95108336	6.34651336
PGM5	phosphogluco	-2.4663871	0.00082212	0.00680768	2.04380641	1.96537188
PHF13	PHD finger pr	-1.6471167	7.92E-05	0.00106272	47.0876966	48.5815361
PHF19	PHD finger pr	-1.7030799	1.85E-07	6.85E-06	83.0146072	84.4086277
PHLDA3	pleckstrin ho	2.10693196	7.26E-10	5.88E-08	115.134428	112.230923
PHLDB2	pleckstrin ho	-1.6172088	0.00093774	0.00756533	218.72736	222.701201
PHLPP1	PH domain a	-1.7713545	5.01E-07	1.60E-05	29.8756407	30.1357022
PHPT1	phosphohisti	1.9336501	2.49E-07	8.71E-06	48.5904954	46.247657
PI15	peptidase inh	2.10357626	1.27E-05	0.0002389	3.64679182	3.95121638
PI3	peptidase inh	3.66762908	0.01289984	0.05500766	0.6812688	0.53228822
PIANP	PILR alpha as	-2.392967	9.48E-05	0.00123266	4.32806062	3.25514718
PICK1	protein intera	1.51258226	0.0001415	0.00170309	19.0354518	19.6332462
PID1	phosphotyros	2.21849354	0.00336789	0.02014935	1.44268687	1.47402891
PIDD1	p53-induced	1.61144286	3.64E-05	0.0005686	20.8187731	19.3875747
PIEZO2	piezo-type m	-2.9688958	3.59E-05	0.00056403	4.46832185	4.01263425
PIF1	PIF1 5'-to-3'	-1.7705465	1.24E-05	0.00023339	11.2008606	12.7749172
PIGN	phosphatidyl	-1.5447556	0.00061469	0.00545788	18.5144816	22.3970504
PIK3R3	phosphoinosi	1.74927378	1.95E-06	4.97E-05	9.75817372	9.58118791
PIM1	Pim-1 proto-	-2.0892167	3.91E-12	5.46E-10	20.9189597	23.2159553
PINLYP	phospholipas	3.74465003	0.00111068	0.00860232	0.30055977	0.26614411
PINX1	PIN2/TERF1	1.60708981	0.00156075	0.01121083	6.03123263	6.42840386
PITPNM2	phosphatidyl	-1.6695856	0.00027207	0.00284883	24.9063859	27.4537884
PKIB	protein kinas	-1.5202989	0.04228645	0.12836584	8.97671833	8.80322821
PKMYT1	protein kinas	-1.5893064	3.12E-05	0.00049909	24.2852291	25.2017998
PKN3	protein kinas	-1.7675657	7.91E-08	3.29E-06	29.6752675	26.3277941
PLA2G4C	phospholipas	2.89363356	0.01230664	0.05311383	0.66123148	0.20472624
PLA2R1	phospholipas	-1.9703382	0.00026724	0.00281051	5.97112067	5.36382742
PLAC8	placenta-spe	-2.3340499	6.66E-08	2.84E-06	6.57224021	6.71502059
PLAT	plasminogen	1.6133159	2.17E-08	1.09E-06	334.723392	333.376205
PLAU	plasminogen	-1.5116237	0.02387813	0.08571754	6.85276265	6.20320499
PLCD1	phospholipas	-1.9415614	5.07E-06	0.00010975	11.4012338	11.7308134
PLCE1	phospholipas	-1.9978932	5.66E-06	0.00012032	27.0503789	28.0884398
PLCG2	phospholipas	1.87163393	0.00058874	0.00527347	2.20410495	2.17009812
PLCL1	phospholipas	3.06121552	0.00140886	0.01042993	0.28052245	0.69606921

PLCL2	phospholipas	1.79200818	0.00023195	0.00251945	6.21156849	4.99532019
PLCXD2	phosphatidyl	2.45523669	0.00294645	0.01821688	2.34436617	2.17009812
PLCXD3	phosphatidyl	2.29222684	0.00365854	0.02143413	1.26235102	0.92126807
PLEK2	pleckstrin 2	-2.5778746	0.00613205	0.0317543	1.76328396	1.5559194
PLEKHA2	pleckstrin ho	-1.6805658	0.00345591	0.02050383	26.3891474	26.0821226
PLEKHA7	pleckstrin ho	2.07424422	2.45E-07	8.57E-06	4.64865771	4.4016141
PLGRKT	plasminogen	1.61959828	9.15E-05	0.00119651	9.65798713	9.99064039
PLIN2	perilipin 2	-1.6305473	9.23E-10	7.35E-08	617.550132	630.208777
PLIN4	perilipin 4	-2.7784502	3.63E-08	1.69E-06	11.7418682	10.5229286
PLK1	polo-like kina	-1.6660463	0.00027493	0.00287032	153.746339	145.846972
PLK2	polo-like kina	1.53488589	6.79E-08	2.88E-06	112.6498	112.660848
PLK3	polo-like kina	2.89084165	1.98E-09	1.41E-07	13.6454134	16.4804621
PLK4	polo-like kina	-1.7778593	3.00E-05	0.00048377	29.6552302	26.4915751
PLOD1	procollagen-l	-1.7875675	3.62E-08	1.69E-06	468.01163	463.930127
PLOD2	procollagen-l	-1.7260608	2.19E-07	7.77E-06	176.348433	169.349544
PLP1	proteolipid p	-2.0583822	9.56E-06	0.00018696	25.1067591	27.0033907
PLXDC2	plexin domai	-2.2923369	5.13E-06	0.00011076	11.5815696	14.8426522
PLXNA1	plexin A1	-1.5682891	0.00021671	0.002388	110.70618	112.558485
PLXND1	plexin D1	-1.8554241	1.72E-06	4.47E-05	85.0383763	81.3377341
PNP	purine nucle	1.6047551	7.69E-05	0.00103744	45.4045619	42.3578585
POLA1	polymerase (	-1.685777	4.24E-05	0.00064097	61.7349758	61.049364
POLB	polymerase (	1.54102336	0.00025589	0.00272339	18.9953772	18.6710329
POLE	polymerase (	-1.5325114	5.58E-05	0.00079975	67.6660219	70.4462983
POLE2	polymerase (	-2.204904	4.92E-06	0.000107	7.71436731	7.34967192
POLE4	polymerase (	1.50615978	0.00126008	0.00955692	20.498176	21.0663298
POLN	polymerase (	2.79611506	0.0188824	0.07234894	0.36067172	0.40945247
POLR2L	polymerase (	1.55515212	0.00096207	0.00772423	64.2596779	61.1107819
POP4	POP4 homolo	1.62118072	0.00017762	0.00203807	25.3071323	25.5703071
POP5	POP5 homolo	1.52547202	0.00091532	0.00743686	24.5056395	23.6049352
PPAPDC1A	phosphatidic	2.30703784	3.91E-05	0.00060377	1.9235825	2.14962549
PPEF1	protein phos	2.90772954	0.00366318	0.02143902	0.42078367	0.32756198
PPFIA4	protein tyros	-3.1835557	6.81E-11	7.41E-09	24.8863486	25.5703071
PPM1D	protein phos	2.14132412	6.87E-14	1.38E-11	27.8518716	25.8159785
PPM1H	protein phos	1.60312136	0.00048661	0.00455337	5.30988919	6.36698598
PPP1R15A	protein phos	2.31553382	0.00044332	0.00420623	59.1902365	57.9989431
PPP1R1B	protein phos	-5.8834968	1.09E-15	3.17E-13	6.43197898	6.75596584
PPP1R3B	protein phos	-1.5588547	0.00672427	0.03403833	11.541495	11.7717587
PPP1R3E	protein phos	-1.7419593	0.00021943	0.00241475	6.37186703	7.69770653
PPP1R3F	protein phos	1.5738883	5.04E-05	0.00073822	9.11697955	8.68039247
PPP2R3B	protein phos	-1.6825797	0.00011283	0.00141917	15.2283615	16.54188
PPP4R3A	protein phos	1.61872674	5.77E-08	2.50E-06	56.2848254	55.6241187
PRAMEF12	PRAME fami	17.816241	2.18E-06	5.39E-05	0.12022391	0.10236312
PRAMEF7	PRAME fami	97.1564453	4.95E-06	0.0001076	0.02003732	0

PRB1	proline-rich p	4.62121111	0.00018531	0.0021093	0.3406344	0.32756198
PRB2	proline-rich p	4.12567296	0.00179921	0.01255047	0.32059708	0.22519886
PRC1	protein regul	-1.8235054	2.24E-06	5.53E-05	171.559514	171.601532
PRDM1	PR domain co	1.60818631	1.14E-06	3.17E-05	21.2796314	20.513569
PRDM8	PR domain co	-3.2639929	0.0002246	0.00245453	2.10391836	2.53860534
PRICKLE1	prickle homo	-1.6758343	8.17E-06	0.00016401	17.1719813	17.2788944
PRKAB1	protein kinas	1.52953961	0.0006347	0.00558341	23.8243707	26.6348835
PRKAR2A-AS	PRKAR2A ant	1.68023843	0.01861554	0.07160327	1.32246297	1.59686465
PRR11	proline rich 1	-2.1241382	6.90E-09	4.05E-07	118.941518	119.478232
PRR20A	proline rich 2	3.05647553	0.01452235	0.05988369	0.38070904	0.24567148
PRR20B	proline rich 2	3.05649093	0.01461781	0.06019023	0.38070904	0.24567148
PRR20C	proline rich 2	3.05649516	0.01464417	0.06024655	0.38070904	0.24567148
PRR20D	proline rich 2	3.05649471	0.01464136	0.06024655	0.38070904	0.24567148
PRR20E	proline rich 2	3.05649869	0.01466619	0.06028495	0.38070904	0.24567148
PRR34-AS1	PRR34 antise	2.36497805	4.53E-05	0.00067481	2.84529911	2.21104336
PRR36	proline rich 3	-2.2806422	0.00886404	0.04166273	2.32432885	2.25198861
PRR7	proline rich 7	-1.6172201	2.98E-06	7.03E-05	29.6953048	30.3813736
PRR9	proline rich 9	111.727263	4.06E-09	2.60E-07	0	0
PRRG2	proline rich G	2.14421592	0.01023431	0.04627618	0.88164198	0.90079544
PRRT2	proline-rich t	-1.6268896	0.00187237	0.01293412	10.0587335	10.4205655
PRRX1	paired relate	-2.9597428	3.16E-06	7.39E-05	67.5257606	65.9423211
PRSS23	protease; ser	-1.5664383	3.00E-08	1.44E-06	96.4796847	99.2717525
PRSS33	protease; ser	-4.6529035	2.94E-16	9.32E-14	7.73440463	7.16541831
PRSS35	protease; ser	4.15042155	0.00032671	0.00327881	0.80149271	0.57323346
PRSS53	protease; ser	-1.5269989	0.02427399	0.08652766	4.28798599	3.84885326
PRTG	protogenin	-1.6205681	0.00186511	0.01289862	17.6729142	15.9686465
PSIP1	PC4 and SFRS	-1.58685	1.11E-07	4.45E-06	234.737177	237.973778
PSMC4	proteasome	1.50306464	0.00024353	0.00262325	165.227722	155.530523
PSPH	phosphoserin	1.58238758	2.77E-05	0.00045144	19.7968699	20.9639667
PSRC1	proline/serin	-1.7864727	1.06E-08	5.88E-07	31.9995964	31.0569702
PSTK	phosphoseryl	2.27817146	0.0001198	0.00148577	2.2642169	1.74017302
PTCH1	patched 1	-1.7239974	0.00158082	0.0113094	20.117467	21.9466527
PTCHD4	patched dom	2.34184517	7.86E-13	1.26E-10	7.65425536	6.32604074
PTGER4P2-C	PTGER4P2-C	2.03265397	0.00925627	0.04284327	0.94175393	0.81890495
PTGES	prostaglandi	-1.769168	1.69E-05	0.00030408	40.9562774	39.9216163
PTHLH	parathyroid h	1.54895416	0.00029376	0.00302705	8.45574807	8.86464608
PTK2B	protein tyros	1.81155855	0.0022323	0.01473482	2.30429154	2.45671485
PTK7	protein tyros	-1.5617044	3.71E-08	1.72E-06	63.3379613	61.4997617
PTN	pleiotrophin	-2.4735638	0.00101287	0.00803251	3.00559766	2.29293386
PTP4A1	protein tyros	2.21026482	1.30E-16	4.51E-14	137.195514	147.70998
PTPRB	protein tyros	-2.1452468	0.00020723	0.00231088	13.0843685	13.1434244
PTPRD	protein tyros	-1.6647078	0.00464404	0.02567194	6.59227752	6.69454796
PTPRH	protein tyros	-2.0560799	8.41E-10	6.77E-08	13.3448536	14.2080009

PTPRJ	protein tyros	-1.6547839	0.00025116	0.00268576	75.8612848	78.676293
PTPRN	protein tyros	2.21764087	1.18E-12	1.79E-10	7.31362096	8.107159
PTPRZ1	protein tyros	-2.1350904	0.00050966	0.00472459	6.91287461	6.96069207
PTRH2	peptidyl-tRN	1.50658205	0.00046012	0.00434538	21.4800046	21.004912
PVRL4	poliovirus re	7.57642445	9.97E-19	4.59E-16	2.24417958	1.67875515
PVT1	Pvt1 oncoger	1.9565188	0.00058231	0.00523233	2.90541107	3.43940079
PXDN	peroxidasin	-1.7555556	9.00E-09	5.07E-07	153.185294	149.859606
QPRT	quinolinate p	-1.8467357	0.02459443	0.08729643	2.24417958	2.21104336
QRFPR	pyroglutamyl	-1.7626289	0.00026615	0.00280584	6.51212825	6.18273237
QRICH2	glutamine ric	1.63091542	0.04424081	0.13211983	1.3625376	1.04410381
RAB27A	RAB27A; me	-1.6561741	0.00208056	0.01393708	7.95481513	8.08668638
RAB31	RAB31; mem	-1.8917293	3.64E-07	1.21E-05	25.4874681	26.9624455
RAB39B	RAB39B; me	2.41657154	5.48E-05	0.00078799	1.82339591	2.067735
RAB3A	RAB3A; mem	1.5202484	0.00233098	0.01521706	11.0205247	10.2158392
RAB4B	RAB4B; mem	1.85505358	0.04235936	0.12848612	10.8602262	3.35751029
RABGGTA	Rab geranylg	1.61736487	9.67E-05	0.00124968	19.3760862	21.1891656
RABIF	RAB interact	1.60778514	0.00019433	0.00219444	16.2302273	16.869442
RAC2	ras-related C	-1.9517271	0.00733734	0.03631316	1.88350786	2.08820762
RAC3	ras-related C	-2.7767624	4.32E-11	4.96E-09	9.81828567	8.02526851
RAD51AP1	RAD51 assoc	-1.5234568	0.00014597	0.00175107	22.6221317	20.3088428
RAD51C	RAD51 paral	1.51671752	2.44E-05	0.00040699	26.9902669	24.6695116
RAD54L	RAD54-like (!	-1.7269001	5.11E-07	1.62E-05	28.3127299	27.9860767
RAPGEF3	Rap guanine	-2.1299304	1.81E-09	1.33E-07	13.6454134	15.1497416
RAPGEFL1	Rap guanine	-1.5231685	0.01066634	0.04763587	12.4832489	10.9323811
RARRES3	retinoic acid	-1.9858215	0.03998968	0.1234871	1.2423137	1.37166579
RASA4	RAS p21 prot	-1.9940954	1.77E-07	6.60E-06	15.3085107	18.8143412
RASA4B	RAS p21 prot	-1.8827728	5.07E-05	0.00074023	43.2806062	38.4475874
RASEF	RAS and EF-l	1.73129487	0.02231353	0.08167584	1.703172	1.33072054
RASL11A	RAS-like; fan	2.52204296	0.00011908	0.00148054	1.12208979	1.47402891
RASSF4	Ras associati	-1.5670875	0.0055136	0.02934066	6.75257606	6.32604074
RASSF8	Ras associati	-1.7215295	4.18E-05	0.00063507	405.575348	413.096602
RBM14-RBM	RBM14-RBM	1.51264399	0.00474342	0.02609982	10.5596664	6.039424
RBM18	RNA binding	1.61192138	1.29E-06	3.46E-05	32.7810518	30.9546071
RBPJ	recombinatic	-1.6362652	5.81E-09	3.54E-07	219.308442	219.691725
RCAN2	regulator of	-3.5646229	0.0089444	0.04195744	0.64119417	1.04410381
RCL1	RNA termina	1.58418764	1.80E-05	0.00031886	27.310864	27.8632409
RCOR2	REST corepre	-2.0286443	0.043184	0.1301932	0.96179125	1.14646693
RECK	reversion-inc	-1.5308599	0.01248796	0.05371746	13.625376	14.474145
RFX2	regulatory fa	-1.9639373	4.55E-05	0.00067634	5.51026237	5.22051905
RGAG4	retrotranspo:	1.7499346	0.0014071	0.01042772	2.2642169	2.90711257
RGL1	ral guanine r	1.80046311	4.64E-07	1.50E-05	21.6403031	20.9230215
RGS10	regulator of	-1.5053485	0.00090709	0.0073972	15.9096303	16.4190442
RGS2	regulator of	1.71395181	2.88E-06	6.83E-05	9.97858421	8.9670092

RGS20	regulator of (	1.81019819	0.00185552	0.01286768	3.30615742	2.68191371
RHOB	ras homolog	1.68265234	5.72E-07	1.76E-05	41.9381059	43.3200718
RHOU	ras homolog	-1.5432897	0.00028626	0.00296871	15.3485854	16.8080241
RIMKLB	ribosomal m	1.50731704	1.79E-06	4.62E-05	56.4651613	55.4603377
RINL	Ras and Rab	1.52816811	0.00098914	0.00790149	8.63608393	9.08984494
RMI2	RecQ mediat	-2.3393507	1.33E-09	1.01E-07	23.4837363	22.4994135
RN7SK	RNA; 7SK sm	5.72123958	1.23E-06	3.35E-05	2.98556034	2.21104336
RNA18S5	RNA; 18S rib	1.51084006	0.00732491	0.03627078	6228.3798	5827.88038
RNA5-8S5	RNA; 5.8S rik	3.04660946	6.09E-08	2.63E-06	10.1388828	9.33551643
RNASE4	ribonuclease	-2.034233	0.02995846	0.10028619	1.84343323	1.84253614
RND1	Rho family G	3.59335148	1.29E-05	0.00024167	0.76141807	0.47087035
RND3	Rho family G	1.52531116	0.00185732	0.01286772	28.7735882	30.524682
RNF144A-AS	RNF144A an	-1.836702	0.00301549	0.0185792	6.1113819	4.44255935
RNF144B	ring finger pı	2.93586229	1.99E-05	0.00034523	1.00186589	0.8598502
RNF157	ring finger pı	-2.2492966	1.77E-09	1.31E-07	12.4431743	14.801707
RNF165	ring finger pı	-2.8411827	0.0010296	0.00813803	3.52656792	3.76696277
RNF208	ring finger pı	1.59278359	0.03207544	0.10538889	1.3625376	1.45355629
RNF25	ring finger pı	1.54352274	0.00178718	0.01248489	21.5000419	20.8001857
RNFT2	ring finger pı	1.90645995	0.00225424	0.01485901	4.46832185	3.93074376
ROBO4	roundabout g	-1.7391987	4.18E-09	2.65E-07	34.9050074	33.8207744
ROMO1	reactive oxyg	1.52555087	0.00093624	0.00755994	73.476844	65.9218485
ROR1	receptor tyro	-1.8170747	0.00018795	0.0021308	12.2027265	10.2363119
RORB	RAR-related	1.69678311	0.01647813	0.0655217	2.76514984	3.93074376
ROS1	ROS proto-oı	-2.0722227	0.00392214	0.02260188	3.90727695	3.64412703
RP9	retinitis pigm	1.57302812	0.00254941	0.01638039	13.4851148	15.1906868
RPL13AP20	ribosomal pr	3.43006707	2.64E-12	3.92E-10	4.96925479	4.27877836
RPL23AP7	ribosomal pr	1.91409554	0.00160054	0.01142181	2.66496325	2.94805782
RPL23AP82	ribosomal pr	1.71711228	0.00027035	0.00283496	7.39377023	6.42840386
RPL23AP87	ribosomal pr	1.94852339	0.01665167	0.06611951	0.78145539	0.90079544
RPL26L1	ribosomal pr	1.56916573	0.00015241	0.00181607	28.9338868	29.7876675
RPLP0P2	ribosomal pr	2.20526301	0.02416545	0.08624637	0.72134344	0.59370609
RPP40	ribonuclease	1.500718	0.0140618	0.05854358	7.07317315	7.3291993
RPPH1	ribonuclease	2.05670045	0.0221997	0.08134943	1.0219032	0.96221332
RPS27L	ribosomal pr	3.12416141	1.83E-18	8.16E-16	105.396291	101.114289
RPS6KL1	ribosomal pr	1.92289622	0.0003054	0.00311991	2.46459008	2.68191371
RPSAP52	ribosomal pr	2.8733533	5.33E-05	0.00077052	3.2861201	2.88663995
RRAD	Ras-related a	16.0441317	3.84E-09	2.50E-07	0.24044781	0.02047262
RRM1	ribonucleotic	-1.5188697	1.41E-05	0.00025954	112.509539	111.65769
RRM2B	ribonucleotic	2.56597687	9.82E-22	6.99E-19	62.6566924	65.5942865
RRN3P1	RRN3 homol	2.02775284	0.0089578	0.04200644	0.86160466	0.90079544
RRN3P3	RRN3 homol	1.50669271	0.03426864	0.11054752	2.14399299	2.68191371
RTL1	retrotranspo:	2.37641768	0.00270632	0.01709562	1.00186589	1.2078848
RTN4R	reticulon 4 re	-1.7457783	0.01788758	0.06963013	3.24604547	2.86616732

RTN4RL2	reticulon 4 re	-2.1199642	9.00E-05	0.00118095	7.63421804	7.43156242
RUNDC3A	RUN domain	3.47055363	8.34E-07	2.45E-05	0.94175393	1.00315856
RUNX1T1	runt-related	2.10798744	0.00327401	0.01975757	0.88164198	1.31024792
RUNX2	runt-related	-2.2368467	0.00029202	0.00301123	6.67242679	6.5512396
RYR3	ryanodine re	2.23128277	0.04806296	0.14087726	0.82153003	0.24567148
S100A1	S100 calcium	-2.0963736	0.00641994	0.03289795	2.98556034	3.1937293
S100A3	S100 calcium	-1.7957183	1.11E-05	0.00021243	29.7153421	26.4711025
S100A6	S100 calcium	1.53320163	0.00095321	0.0076617	1074.32083	1058.06614
S1PR1	sphingosine-	1.65717666	0.0005019	0.00466272	7.41380755	7.26778143
S1PR2	sphingosine-	-1.7451555	0.00049435	0.00461056	13.7055253	12.8363351
S1PR3	sphingosine-	2.43546991	0.01852377	0.07132728	0.94175393	0.38897985
SALL2	spalt-like tra	-1.7209642	0.00027351	0.00285977	15.6090705	16.1324275
SAMD12	sterile alpha	1.50734349	0.00272791	0.01719392	5.550337	5.62997153
SAMD14	sterile alpha	-1.513987	0.0016456	0.01166603	10.5396291	12.0993206
SAMD5	sterile alpha	2.239939	0.00090784	0.0073972	1.20223906	1.67875515
SAT1	spermidine/s	1.69995867	2.90E-06	6.86E-05	35.826724	34.3530626
SBDSP1	Shwachman-	1.52945071	0.00053376	0.00489801	20.7185865	18.8552865
SCARA3	scavenger re	-3.1897281	1.19E-06	3.29E-05	9.17709151	8.92606395
SCD	stearoyl-CoA	-2.5550383	2.79E-20	1.66E-17	471.197563	488.394912
SCG5	secretograni	1.77474182	4.29E-05	0.00064829	6.2316058	6.039424
SCMH1	sex comb on	-1.6200329	1.85E-06	4.76E-05	26.4492594	26.716774
SCN2A	sodium chan	1.82560189	2.80E-06	6.68E-05	7.17335974	6.18273237
SCN9A	sodium chan	2.91821695	0.01910052	0.07297628	0.38070904	0.24567148
SCNM1	sodium chan	1.75185979	0.00910999	0.04251031	23.0028407	25.9592869
SCNN1A	sodium chan	-2.5838567	4.14E-05	0.00063244	5.83085945	5.50713579
SCO2	SCO2 cytochr	2.84416696	2.94E-08	1.43E-06	3.14585888	2.86616732
SCUBE3	signal peptid	-1.7171435	0.00370543	0.02164854	12.0624653	13.4709864
SDC3	syndecan 3	-3.6143207	6.04E-14	1.23E-11	102.030022	101.544214
SDCBP2-AS1	SDCBP2 antis	1.57418184	0.01419602	0.05886163	3.00559766	3.56223653
SDSL	serine dehyd	1.97789593	2.17E-05	0.00037179	7.41380755	6.67407534
SEC11C	SEC11 homol	1.85617459	1.43E-05	0.00026237	14.8276151	13.491459
SELM	selenoprotei	1.63842807	0.000202	0.00226844	15.9897795	14.9245427
SEMA3A	sema domai	-1.9529197	4.80E-06	0.00010499	148.536636	141.424885
SEMA3C	sema domai	-1.6394568	0.00306705	0.01884575	245.637478	250.605387
SEMA3D	sema domai	-1.6596653	0.00029998	0.00308206	14.8075778	15.8048655
SEMA3F	sema domai	-1.9866346	4.70E-09	2.94E-07	25.9884011	27.3718979
SEMA4B	sema domai	-1.8181669	9.49E-10	7.49E-08	58.3286318	56.8524761
SEMA4C	sema domai	-1.5139773	2.06E-05	0.00035442	42.0984045	41.2932821
SEMA4G	sema domai	-1.6955768	0.00100262	0.00797786	14.9278017	18.0568541
SEMA5A	sema domai	1.83408746	0.00745491	0.03682493	2.32432885	2.29293386
SEMA6A	sema domai	2.36198521	6.22E-08	2.67E-06	5.69059823	4.56539509
SEMA6B	sema domai	-4.3175197	1.62E-14	3.60E-12	84.7177792	83.5692501
1-Sep	septin 1	-1.9703736	0.04254791	0.12887361	1.94361982	1.84253614

5-Sep	septin 5	-1.6046433	0.0042795	0.02413418	7.97485244	6.87880158
SEPT5-GP1B	NA	-2.3227313	0.00187583	0.01294552	8.51586002	9.43787955
SERPINA3	serpin peptic	-2.8933509	1.15E-07	4.58E-06	168.373581	166.114869
SERPINA5	serpin peptic	-2.025986	1.64E-07	6.19E-06	33.6025818	33.9845554
SERPINB2	serpin peptic	3.34408903	5.41E-06	0.00011606	3.78705305	3.93074376
SERPINB5	serpin peptic	2.80257295	1.54E-06	4.03E-05	1.2423137	1.2078848
SERPIND1	serpin peptic	1.81364414	5.61E-05	0.00080315	4.54847112	4.17641524
SERPINE1	serpin peptic	2.22130101	5.01E-07	1.60E-05	9.85836031	10.6457643
SERPINF1	serpin peptic	-3.5009573	1.08E-09	8.38E-08	11.7017935	12.2221564
SERPINI1	serpin peptic	2.23187427	6.63E-05	0.00091801	2.44455276	2.43624223
SERTAD1	SERTA doma	2.98205867	3.06E-08	1.46E-06	14.2665702	14.3513092
SERTAD4	SERTA doma	-1.6550281	2.02E-05	0.00034925	23.7842961	26.1230679
SESN1	sestrin 1	2.91527253	3.31E-30	6.74E-27	14.0060851	15.0678511
SESN2	sestrin 2	3.60278348	2.83E-18	1.22E-15	14.4869807	14.2694187
SESN3	sestrin 3	1.51753554	0.00225745	0.01486641	7.83459122	9.02842707
SETBP1	SET binding p	-1.6184661	0.01528523	0.06224044	7.49395682	6.63313009
SFRP1	secreted friz	-1.7636131	1.89E-07	6.93E-06	379.326461	377.5766
SFTA1P	surfactant as	2.60822185	0.04419529	0.13203921	0.28052245	0.24567148
SGCZ	sarcoglycan;	-1.6139547	0.03718989	0.11694702	1.98369445	2.4976601
SGK3	serum/gluco	1.80332241	0.00607624	0.03153407	2.14399299	3.84885326
SGSM2	small G prot	-1.6824043	6.80E-06	0.00014083	70.9321047	71.4289842
SH2B2	SH2B adapto	-1.6153939	0.00197725	0.01340521	9.31735273	7.02210994
SH2D1B	SH2 domain	-1.6817384	0.00244042	0.01576541	5.37000114	6.69454796
SH2D2A	SH2 domain	1.70384222	4.32E-07	1.41E-05	18.0936979	17.1765313
SH3BP1	SH3-domain	-1.7856439	5.26E-08	2.31E-06	43.7615019	41.7436798
SH3D21	SH3 domain	-2.0347311	1.31E-07	5.08E-06	9.19712882	9.49929742
SH3GL3	SH3-domain	-1.8194337	0.03619783	0.11494828	2.24417958	2.23151599
SHC2	SHC (Src hom	-1.8288816	0.0071182	0.03539478	3.12582156	2.66144109
SHF	Src homology	-1.7123205	0.00046809	0.00441184	11.7418682	11.1575799
SIK1	salt-inducible	1.67668017	0.00053625	0.00491455	5.39003846	6.4693491
SIPA1L2	signal-induce	1.58919599	0.0003064	0.00312558	13.9459731	14.6788712
SIRPA	signal-regula	-1.5192825	0.00017665	0.00203022	16.0098168	16.9103872
SIRT4	sirtuin 4	1.99085018	0.00311585	0.01907876	1.48276151	2.067735
SIRT7	sirtuin 7	1.65594972	0.00022366	0.00244621	19.7968699	20.349788
SLAMF7	SLAM family	3.22091835	1.04E-12	1.61E-10	3.74697841	4.23783311
SLAMF9	SLAM family	3.26276112	7.18E-06	0.00014738	1.20223906	0.94174069
SLC12A7	solute carrier	-1.6411269	1.21E-08	6.58E-07	77.0635239	77.0589558
SLC12A8	solute carrier	-1.911685	0.00132496	0.00994316	4.12768745	4.50397722
SLC13A3	solute carrier	1.67858652	0.01836746	0.07085942	1.80335859	2.21104336
SLC16A5	solute carrier	-1.5746572	1.75E-06	4.52E-05	28.252618	27.4333158
SLC22A18AS	solute carrier	-1.7281591	0.02371326	0.08530291	2.70503789	2.96853044
SLC25A16	solute carrier	1.56527939	0.00681091	0.03435835	3.88723963	3.11183881
SLC25A20	solute carrier	1.72511562	3.50E-06	8.05E-05	18.9352652	17.3403123

SLC25A27	solute carrier	-1.7354784	0.00215552	0.01431419	4.60858307	4.23783311
SLC25A37	solute carrier	-1.6535102	3.58E-08	1.68E-06	47.6487415	47.2508156
SLC25A4	solute carrier	1.63225119	1.27E-06	3.44E-05	64.2797152	59.0225743
SLC25A45	solute carrier	1.77333981	3.10E-05	0.00049642	5.47018773	5.71186202
SLC27A1	solute carrier	-1.8431516	5.06E-08	2.24E-06	29.4748943	30.0538117
SLC29A1	solute carrier	-1.619085	4.75E-06	0.00010446	28.6533643	25.734088
SLC29A3	solute carrier	1.51326263	0.02294244	0.08313061	2.72507521	2.53860534
SLC29A4	solute carrier	-3.6848328	1.06E-08	5.88E-07	7.45388218	6.65360272
SLC2A12	solute carrier	-2.465566	9.22E-07	2.64E-05	11.8821294	12.897753
SLC2A3	solute carrier	-1.5332917	1.24E-06	3.40E-05	61.3743041	56.9343666
SLC2A4RG	SLC2A4 regul	-1.5736754	2.17E-06	5.38E-05	65.5220289	63.3218252
SLC30A1	solute carrier	1.90100119	1.13E-06	3.13E-05	20.6584745	22.3151599
SLC31A2	solute carrier	1.6267456	0.00074135	0.0062812	4.68873234	5.73233465
SLC38A3	solute carrier	-1.911872	0.0238875	0.08571754	2.18406763	1.22835742
SLC3A2	solute carrier	1.89748565	5.26E-07	1.67E-05	187.268771	187.140254
SLC43A2	solute carrier	1.96405745	0.00145194	0.01064383	2.14399299	1.88348138
SLC44A2	solute carrier	-1.6202556	4.01E-07	1.32E-05	75.8612848	78.2463679
SLC44A5	solute carrier	1.50674782	0.03257032	0.10640982	3.42638133	2.45671485
SLC4A7	solute carrier	-1.5136332	0.00207276	0.01389413	77.6245688	86.0669102
SLC52A1	solute carrier	5.04231357	5.23E-06	0.00011262	0.44082099	0.18425361
SLC6A10P	solute carrier	-1.5563262	0.00076077	0.00640388	7.83459122	10.338675
SLC6A12	solute carrier	72.512723	4.31E-09	2.72E-07	0.02003732	0.02047262
SLC6A17	solute carrier	3.64546544	0.00010666	0.00135592	0.52097026	0.40945247
SLC6A6	solute carrier	-1.6216198	8.39E-07	2.45E-05	88.3846084	91.0008125
SLC7A5	solute carrier	1.57228672	7.54E-07	2.25E-05	578.657698	587.154849
SLC7A5P1	solute carrier	2.44297735	0.04212035	0.12804121	0.70130612	0.28661673
SLC8A1	solute carrier	-1.8011	5.92E-06	0.00012544	17.913362	17.626929
SLC9A7	solute carrier	-1.503291	3.62E-05	0.00056662	44.9837782	44.6303198
SLCO4A1	solute carrier	-1.6866319	8.53E-09	4.84E-07	153.105145	146.522568
SLCO4A1-AS	SLCO4A1 ant	-2.2902419	4.46E-06	9.90E-05	27.811797	28.0474945
SLITRK6	SLIT and NTF	-1.5205767	0.00032487	0.00326955	123.449914	122.712907
SMAD3	SMAD family	-1.5395344	3.16E-07	1.08E-05	161.661079	166.155814
SMAD6	SMAD family	-1.6499521	0.00065435	0.00570001	15.5689959	15.9686465
SMARCC2	SWI/SNF rel	-1.5682335	4.23E-06	9.50E-05	173.002201	169.410961
SMC2	structural ma	-1.5667151	1.36E-05	0.00025198	104.113903	109.200975
SMIM11	NA	1.55329962	0.00093234	0.00754927	9.67802445	8.6189746
SMIM4	small integra	1.57725016	0.00107471	0.00840599	5.73067286	4.93390232
SMKR1	small lysine-	1.6876319	0.0208579	0.07786226	1.72320932	1.67875515
SMN1	survival of m	1.74462104	0.00241665	0.01566154	64.3197898	44.3232304
SMO	smoothened,	-1.5498849	1.50E-05	0.00027447	31.4185142	28.7640364
SMPDL3B	sphingomyel	3.0558069	0.00126863	0.00961152	0.40074635	0.40945247
SNAI2	snail family z	2.67236426	0.01529534	0.06224044	0.48089562	0.40945247
SNAP25	synaptosoma	-2.2910012	7.86E-06	0.00015889	10.6398157	9.84733202

SNAPC1	small nuclea	1.62562392	0.00051062	0.00472828	44.0820989	42.5011669
SNCG	synuclein; ga	-2.1147863	0.00173026	0.01213478	4.90914284	5.36382742
SNED1	sushi; nidoge	-3.941014	3.46E-07	1.16E-05	13.8858612	13.4505138
SNHG12	small nucleo	1.58419832	0.01662916	0.06608537	10.7600396	11.751286
SNHG15	small nucleo	1.51374413	0.01427297	0.05904327	9.41753932	9.66307841
SNHG9	small nucleo	2.76159549	0.00188824	0.01300599	0.62115685	0.67559658
SNORA57	small nucleo	2.82560091	0.0116517	0.05095071	0.26048513	0.26614411
SNORD3A	small nucleo	2.67113198	0.02553188	0.08990729	1.08201516	0.73701445
SNTA1	syntrophin; a	-1.6723362	0.00061254	0.00544974	12.1826892	12.1402659
SNTB1	syntrophin; b	-2.2723537	6.02E-05	0.00085246	48.690682	49.8303662
SOBP	sine oculis bi	-2.1916197	2.86E-06	6.81E-05	11.3411218	12.078848
SOCS2	suppressor o	1.77925617	6.22E-07	1.90E-05	11.6416816	8.72133771
SOCS3	suppressor o	-1.8502589	1.60E-10	1.55E-08	23.2232512	22.8269755
SOGA3	SOGA family	2.27118084	0.00839627	0.04017973	1.00186589	0.49134297
SORBS3	sorbin and SI	-1.751343	2.23E-09	1.53E-07	68.6879251	67.1706785
SORCS1	sortilin-relat	-5.734705	2.42E-11	2.96E-09	20.2777655	18.6710329
SORCS2	sortilin-relat	-2.3674223	1.18E-05	0.00022487	6.85276265	6.8992742
SOX12	SRY (sex det	-1.5440472	3.68E-05	0.0005743	86.5411751	81.1944258
SOX15	SRY (sex det	2.11384588	0.0099661	0.04536149	0.98182857	0.7779597
SOX18	SRY (sex det	-1.5911469	0.0016133	0.01149912	7.57410609	6.63313009
SOX2	SRY (sex det	-2.2897774	7.94E-06	0.00015995	15.5088839	15.8458108
SOX4	SRY (sex det	-1.734626	8.52E-05	0.00112707	41.1967252	43.3610171
SOX6	SRY (sex det	1.59364306	0.01589152	0.06389922	2.18406763	3.31656505
SOX9	SRY (sex det	-1.9932394	4.19E-11	4.85E-09	31.4385515	29.9514485
SP1	Sp1 transcrip	-1.5411389	0.00020431	0.00228362	125.553833	125.251512
SPACA6P	sperm acros	-1.51314	0.0042886	0.02415685	6.81268802	5.7732799
SPAG1	sperm associ	1.56639363	0.00028249	0.00293854	18.1538098	19.858445
SPANXA1	sperm protei	-1.9557922	4.08E-05	0.00062552	10.5396291	9.76544153
SPANXA2	SPANX famil	-1.9557922	4.08E-05	0.00062552	10.5396291	9.76544153
SPATA6	spermatoger	-1.7918125	5.40E-08	2.37E-06	24.4655649	27.2900075
SPATS2L	spermatoger	-1.5005546	1.71E-05	0.00030669	84.3571075	78.6967657
SPC24	SPC24; NDC8	-2.2048832	0.00014076	0.00170045	8.25537489	9.58118791
SPC25	SPC25; NDC8	-1.7082723	0.00096206	0.00772423	7.99488976	8.31188524
SPEG	SPEG comple	-1.5368393	0.00011	0.00139097	29.3546704	29.0301805
SPHK1	sphingosine	1.89255313	1.31E-06	3.51E-05	12.1626518	15.722975
SPIN4	spindlin fami	-1.5527025	0.00326207	0.0197149	14.4669434	15.7639203
SPON2	spondin 2; ex	3.17207647	1.12E-06	3.12E-05	7.29358364	5.83469777
SPP1	secreted pho	-1.6503999	0.00055198	0.00501992	19.5363848	20.2064796
SPRR2D	small proline	3.04999694	0.00025559	0.0027223	0.72134344	0.7779597
SPRY1	sprouty RTK	-2.3601089	2.13E-07	7.59E-06	6.05126995	5.67091678
SPRY4	sprouty RTK	-1.8417975	3.74E-13	6.42E-11	72.595202	76.1376877
SPTBN5	spectrin; bet	1.93774297	6.85E-05	0.00094099	3.06570961	3.78743539
SQSTM1	sequestosom	1.85351964	7.27E-05	0.00098906	673.955181	644.846703

SRA1	steroid recep	1.9983513	5.51E-07	1.71E-05	29.6552302	28.7026185
SRCIN1	SRC kinase si	-1.7624431	0.00190394	0.0130762	9.59787518	11.3213609
SREBF1	sterol regula	-2.1655172	5.81E-10	4.78E-08	162.602833	156.779353
SREBF2	sterol regula	-1.6719717	4.34E-08	1.97E-06	147.695069	148.610776
SRGAP3	SLIT-ROBO R	2.15853256	3.02E-10	2.71E-08	7.41380755	7.3291993
SRGN	serglycin	-1.6214264	0.00163234	0.01160749	5.06944138	5.73233465
SRP19	signal recogr	1.52426092	5.97E-05	0.00084694	35.3057538	32.2443824
SRPX	sushi-repeat	-1.6889601	1.25E-10	1.24E-08	147.354434	143.8816
SRPX2	sushi-repeat	-2.1000396	1.94E-09	1.40E-07	18.4343323	19.3466294
SRRM3	serine/argini	-1.7082489	6.30E-05	0.00088165	8.83645711	8.04574113
SRSF8	serine/argini	1.5624967	1.39E-06	3.69E-05	38.9325083	42.112187
SSC4D	scavenger re	2.46477427	0.01895694	0.0725444	0.70130612	0.7779597
SSFA2	sperm specif	-1.880053	3.64E-06	8.33E-05	504.058764	506.922637
SSSCA1	Sjogren synd	1.54248768	0.0042876	0.02415685	22.9226914	20.1245891
ST3GAL5	ST3 beta-gal	-2.3369183	7.22E-14	1.43E-11	41.7577701	41.1499737
ST6GALNAC2	ST6 (alpha-N	-3.1834258	0.00063418	0.00558225	2.84529911	2.21104336
STAC2	SH3 and cyst	-2.1986494	0.01152976	0.05058825	1.56291078	1.12599431
STARD4	StAR-related	-1.6076185	1.90E-07	6.93E-06	29.074148	27.0238633
STARD4-AS1	STARD4 antis	-2.1180685	0.00121188	0.0092852	10.2591067	10.8709632
STARD8	StAR-related	-1.5906945	0.01575848	0.06352873	4.00746354	4.93390232
STC2	stanniocalcir	1.63214137	0.0003217	0.00324641	29.5550436	27.7404052
STMN1	stathmin 1	-1.7601078	1.10E-11	1.44E-09	267.077408	270.422887
STMN2	stathmin 2	-4.6332968	0.00267864	0.01695837	1.04194052	1.31024792
STON1	stonin 1	-1.7348546	0.0001756	0.00202466	13.304779	13.6347674
STRA6	stimulated b	-3.2336288	9.51E-10	7.49E-08	164.125669	160.89435
STX3	syntaxin 3	1.76256872	3.29E-05	0.00052063	27.2106774	26.0207048
SUGCT	succinyl-CoA	2.21494136	1.90E-06	4.86E-05	3.52656792	3.02994831
SULF1	sulfatase 1	-1.5501735	0.00047155	0.00442982	12.8238833	13.5733495
SUN2	Sad1 and UN	-1.7711848	1.89E-08	9.75E-07	93.8748334	91.8811354
SURF2	surfeit 2	1.65416926	0.00012962	0.00158147	24.0648186	22.253742
SUSD1	sushi domair	1.5967905	0.02763798	0.09504559	1.9235825	2.00631713
SUSD2	sushi domair	-2.625368	6.29E-11	6.90E-09	62.8370283	57.8965799
SUSD6	sushi domair	2.19857937	3.48E-10	3.07E-08	10.6398157	10.1134761
SYNE2	spectrin repe	-2.0309161	0.0020299	0.01366466	19.8569818	20.8820762
SYNGAP1	synaptic Ras	-1.5463468	7.49E-05	0.00101216	19.3961235	16.1529001
SYNGR1	synaptogyrin	-1.9005491	8.40E-07	2.45E-05	26.5895206	28.4774196
SYNGR3	synaptogyrin	-1.61706	0.00918044	0.04269924	4.8690682	5.17957381
SYNM	synemin; inte	-1.509407	0.00031816	0.00322028	77.5243822	81.2558436
SYP	synaptophysi	4.79082444	2.17E-10	2.00E-08	1.84343323	0.83937757
SYT3	synaptotagm	1.64084422	0.02241368	0.08184543	1.64306005	2.12915287
TAC1	tachykinin; pı	21.9414333	9.46E-06	0.00018572	0	0.10236312
TAF13	TAF13 RNA p	1.77383956	1.92E-06	4.89E-05	17.7931381	18.6915055
TAF3	TAF3 RNA po	1.55336713	4.57E-06	0.00010113	13.304779	13.6347674

TAGLN3	transgelin 3	1.87367183	1.98E-05	0.00034403	8.85649442	9.99064039
TANC2	tetratricopep	-1.8315271	1.40E-07	5.36E-06	56.765721	61.5611796
TANGO2	transport and	1.58950887	0.00027296	0.00285607	8.07503903	8.45519361
TAPBP	TAP binding	-1.5177919	1.62E-05	0.00029302	77.6646434	73.2305751
TARSL2	threonyl-tRN	1.6440903	0.0001029	0.00131685	7.25350901	6.32604074
TAX1BP3	Tax1 (humar	1.52523868	4.43E-05	0.00066299	62.436282	60.2918769
TBC1D3H	TBC1 domair	710.807983	0.00024218	0.0026122	0	0
TBX2-AS1	TBX2 antiser	-1.983804	0.02722316	0.09425589	1.62302273	1.76064564
TCEA2	transcription	-1.5770599	1.19E-05	0.00022556	38.4916873	40.1672878
TCEANC	transcription	1.77902352	0.02984792	0.10003827	1.50279883	1.16693955
TCEB2	transcription	1.51542033	0.0050065	0.02722152	74.0980009	70.8148055
TCEB3-AS1	TCEB3 antise	2.65879664	0.00558717	0.0295446	0.30055977	0.47087035
TCF7	transcription	-1.8489052	0.01624401	0.06491285	2.64492594	2.96853044
TCF7L1	transcription	-1.7119413	0.01963742	0.07439169	3.06570961	2.51813272
TCHH	trichohyalin	3.21901655	1.31E-07	5.09E-06	1.90354518	1.10552168
TCN2	transcobalan	-1.5647123	0.0061195	0.03170082	7.03309851	9.88827727
TCTA	T-cell leuken	1.51684402	3.47E-06	7.99E-05	25.1468337	26.0207048
TCTN2	tectonic fam	-1.7371381	2.30E-05	0.00039101	13.9860478	13.2867328
TEAD2	TEA domain	-1.6178279	4.78E-05	0.00070664	19.0154145	20.0631713
TENM1	teneurin trar	-1.6991675	0.00512318	0.02770792	4.80895625	4.74964871
TEX14	testis expres	10.5083368	5.91E-06	0.00012536	0.22041049	0.02047262
TEX9	testis expres	1.78128873	0.00193175	0.01319168	2.2642169	2.17009812
TF	transferrin	-2.5131469	0.00672837	0.03403833	1.52283615	1.35119317
TFAP4	transcription	-1.5488238	4.00E-06	9.04E-05	28.252618	26.8396097
TFEC	transcription	6.02978248	0.00320723	0.01950609	0.14026122	0.12283574
TFF2	trefoil factor	-1.6543721	0.01065298	0.04760618	3.98742622	3.78743539
TFPI	tissue factor	-1.8436924	0.00264882	0.01681443	7.61418073	8.82370083
TFPI2	tissue factor	2.340775	2.76E-11	3.34E-09	37.3295229	36.3798524
TGFBI	transforming	-2.7343932	3.95E-09	2.55E-07	548.140863	538.737094
TGFBR2	transforming	-1.5523467	0.00343851	0.02046538	124.091109	133.624815
TGM2	transglutami	-2.1651922	2.10E-13	3.78E-11	894.205377	888.204781
THBD	thrombomoc	2.40278894	0.01122428	0.04958377	0.36067172	0.53228822
THBS4	thrombospor	-1.8804526	0.04971392	0.14458079	1.46272419	1.51497416
THRB	thyroid horm	-2.7432075	2.36E-14	4.95E-12	27.5713492	27.7813504
THSD1	thrombospor	2.2986308	0.03234318	0.10586189	0.52097026	0.3480346
THSD4	thrombospor	-1.8853702	0.00015989	0.00188155	87.2024066	89.7519825
TIAF1	TGFB1-induc	3.50962253	0.03549018	0.11337508	2.54473935	1.6378099
TIAM1	T-cell lymph	-2.3205762	4.44E-07	1.44E-05	46.105868	47.5783776
TICRR	TOPBP1-inte	-1.684939	6.84E-05	0.00094099	25.5275428	24.8332926
TIE1	tyrosine kina	-2.3278506	3.16E-06	7.39E-05	5.59041164	5.81422514
TIGAR	TP53 inducec	2.95986303	2.85E-26	4.52E-23	33.6827311	30.5860999
TIGD3	tigger transp	1.76557677	0.04244045	0.12863438	0.74138075	1.00315856
TIMM22	translocase c	1.51161791	0.00054659	0.00498043	31.2782529	29.5624687

TIMP1	TIMP metallo	1.52033062	0.00038517	0.00376996	153.906637	156.451791
TIMP2	TIMP metallo	-1.7767024	8.83E-08	3.63E-06	274.691588	284.856087
TK1	thymidine kii	-1.8159897	9.97E-11	1.04E-08	101.909798	96.2622768
TLCD1	TLC domain c	1.85976849	3.29E-06	7.64E-05	16.9716081	14.8221796
TLL2	tolloid-like 2	2.34365939	2.79E-06	6.68E-05	1.66309737	1.6378099
TLR6	toll-like rece	-1.6948073	0.00144183	0.0105915	14.5871673	15.4773036
TLR8-AS1	TLR8 antisen	3.97907225	0.00273292	0.01721026	0.10018659	0.24567148
TM4SF18	transmembra	-1.8350664	6.35E-09	3.83E-07	19.6365713	18.9985948
TMCC2	transmembra	1.55870461	0.001273	0.00962415	6.81268802	6.85832895
TMED7-TICAI	TMED7-TICAI	1.99436549	0.00192823	0.01317948	10.3392559	9.60166054
TMEM107	transmembra	-1.7144972	0.00107062	0.00838317	8.51586002	8.06621376
TMEM121	transmembra	-1.9508321	2.24E-05	0.00038205	11.8019801	14.310364
TMEM123	transmembra	-1.5270064	1.53E-06	4.02E-05	250.546621	264.588189
TMEM130	transmembra	-1.5737771	0.0275003	0.09482379	3.54660523	3.11183881
TMEM154	transmembra	-1.7431663	0.00064264	0.00563242	6.2316058	7.53392554
TMEM158	transmembra	-1.6320828	0.00234744	0.01529027	105.937299	111.452964
TMEM161B-/	TMEM161B a	1.7214714	0.01457378	0.06002629	2.86533643	2.29293386
TMEM175	transmembra	1.50648685	0.00189282	0.01301707	10.9804501	12.3449921
TMEM182	transmembra	1.5132347	0.02709397	0.09403705	1.94361982	2.47718747
TMEM199	transmembra	1.58451164	7.79E-06	0.00015765	26.7498191	25.5907797
TMEM2	transmembra	1.75732666	1.04E-06	2.92E-05	274.23073	284.037182
TMEM200A	transmembra	-1.5004796	0.00709358	0.0352877	11.1407486	10.338675
TMEM200B	transmembra	-2.3527649	2.53E-06	6.11E-05	12.423137	10.993799
TMEM208	transmembra	1.61847532	0.00062193	0.00550308	28.2325806	26.3687394
TMEM217	transmembra	2.62876023	0.02847442	0.09711718	0.28052245	0.26614411
TMEM231	transmembra	-1.5262117	0.00026608	0.00280584	16.1100034	16.2962085
TMEM234	transmembra	1.66147166	0.01497729	0.06136901	3.66682914	3.97168901
TMEM254-AS	TMEM254 ar	2.22609016	0.04605329	0.13624777	0.50093294	0.45039772
TMEM255B	transmembra	1.64408553	0.0015425	0.01112458	4.90914284	4.70870346
TMEM27	transmembra	2.94574324	0.00050571	0.004695	0.6812688	0.67559658
TMEM45A	transmembra	-1.6995653	2.65E-05	0.0004371	14.066197	14.9245427
TMEM74B	transmembra	-3.2304315	0.00125567	0.00953814	2.2642169	1.9858445
TMEM92	transmembra	-2.0100812	0.00086202	0.00708052	3.76701573	3.1937293
TMPO	thymopoietir	-1.6971927	2.38E-06	5.81E-05	227.363444	227.225651
TMPO-AS1	TMPO antise	-2.7301458	8.21E-06	0.00016401	6.83272534	7.00163732
TMPRSS15	transmembra	-1.7392748	4.50E-08	2.00E-06	59.0099006	57.2619286
TMTC4	transmembra	-1.5903659	0.00022932	0.00249661	18.2740337	17.626929
TMX4	thioredoxin-r	-1.7920363	1.26E-06	3.43E-05	174.104253	174.426754
TNC	tenascin C	-1.6499771	9.06E-05	0.00118738	639.190435	641.202576
TNFAIP2	tumor necros	-2.6905159	1.03E-16	3.66E-14	137.536149	135.467351
TNFAIP8L1	tumor necros	-1.8516183	6.53E-07	1.98E-05	27.0103043	25.7750333
TNFRSF10B	tumor necros	1.55201828	1.30E-08	6.91E-07	136.313872	137.064216
TNFRSF11A	tumor necros	-2.1518418	1.52E-05	0.00027751	5.28985187	5.30240955

TNFRSF11B	tumor necros	-2.285137	7.44E-14	1.45E-11	20.8388104	21.92618
TNFRSF12A	tumor necros	1.83568514	2.55E-05	0.00042269	111.3073	102.60879
TNFRSF19	tumor necros	-1.8808677	1.81E-05	0.00032071	38.2111649	41.190919
TNFRSF1B	tumor necros	-2.2159223	8.14E-05	0.00108658	7.71436731	7.51345291
TNFRSF21	tumor necros	-1.8164248	8.28E-06	0.00016478	352.316157	353.459849
TNFSF15	tumor necros	2.56372336	0.00016094	0.00189084	0.94175393	0.69606921
TNFSF9	tumor necros	1.83498971	3.82E-08	1.76E-06	28.7936255	31.2412238
TNNT1	troponin T ty	-1.5992815	0.00028536	0.00296191	41.056464	44.3232304
TNRC6C-AS1	TNRC6C antis	-2.0976667	0.00456368	0.02533579	4.22787403	4.23783311
TNS1	tensin 1	-1.5251265	0.00915428	0.04264715	4.30802331	4.66775821
TNS2	tensin 2	-2.0853121	4.99E-08	2.21E-06	110.465732	114.708111
TNS3	tensin 3	-2.503514	5.47E-10	4.59E-08	150.119584	163.125866
TNXB	tenascin XB	-1.5559646	0.00144513	0.01059965	10.8001142	12.7749172
TOB2P1	transducer o	1.65985875	0.00125113	0.00951432	3.18593351	3.09136619
TONSL	tonsoku-like;	-1.5841945	2.79E-05	0.00045366	52.2973992	50.1988734
TOP2A	topoisomera	-2.5149158	2.43E-11	2.96E-09	499.30992	515.152631
TOX2	TOX high mo	-3.9609673	4.18E-23	3.50E-20	26.1286623	28.3136386
TP53I3	tumor protei	2.61788796	1.97E-10	1.83E-08	40.615643	40.3515414
TP53INP1	tumor protei	3.16342528	2.51E-19	1.33E-16	7.97485244	7.77959702
TP53TG1	TP53 target 1	2.19097293	9.19E-06	0.00018115	4.34809794	5.22051905
TPPP	tubulin polym	3.26762664	0.00118447	0.00909479	0.50093294	0.38897985
TPX2	TPX2; microt	-1.5459717	5.05E-05	0.00073826	239.886768	232.691841
TRABD2B	TraB domain	-2.2314108	0.0037734	0.02198449	2.9454857	2.8456947
TRAF1	TNF receptor	-1.5986006	0.0016789	0.01186218	11.0806367	11.2804157
TRAF4	TNF receptor	1.65153502	1.88E-05	0.00033127	75.2000533	76.178633
TRAF5	TNF receptor	-1.5413581	0.00034253	0.00341358	19.5163474	19.5104104
TRAM2-AS1	TRAM2 antis	-1.513017	0.03274507	0.10680934	3.88723963	4.36066886
TRAPPC2B	trafficking pr	1.56903736	0.00405715	0.02314589	4.62862039	5.54808103
TRAPPC2L	trafficking pr	1.68015254	0.00022006	0.00241987	9.71809908	9.15126281
TRERF1	transcription	-1.5142639	0.00141508	0.01045972	23.6239976	26.225431
TRIAP1	TP53 regulat	2.06211176	9.46E-14	1.77E-11	36.8285899	33.4113219
TRIB2	tribbles pseu	-2.6659378	1.03E-10	1.06E-08	80.1893454	77.8369155
TRIL	TLR4 interac	-2.0539726	0.04622107	0.13663067	1.14212711	1.49450153
TRIM22	tripartite mo	2.11123289	6.68E-06	0.0001386	2.34436617	2.86616732
TRIM3	tripartite mo	1.53840763	3.41E-05	0.00053953	10.8802635	9.78591415
TRIM35	tripartite mo	1.62757605	2.91E-07	1.00E-05	17.5526903	19.2647389
TRIM36	tripartite mo	1.58780405	0.00100129	0.00797173	5.51026237	5.7732799
TRIM43	tripartite mo	451.507038	4.95E-07	1.59E-05	0	0
TRIM48	tripartite mo	8.6252592	0.00287746	0.01789902	0.02003732	0.02047262
TRIM49	tripartite mo	41.0844135	0.00587784	0.03068316	0	0.02047262
TRIM49C	tripartite mo	17.7614213	0.00137365	0.01023843	0.24044781	0.02047262
TRIM49D2	tripartite mo	25.335567	5.51E-07	1.71E-05	0	0.16378099
TRIM53AP	tripartite mo	4.22099066	0.00010954	0.00138633	0.64119417	0.3480346

TRIM64B	tripartite mo	43.7996871	4.19E-05	0.00063507	0.06011195	0
TRIM68	tripartite mo	1.54601565	0.00042071	0.00404553	6.69246411	6.91974683
TRIML2	tripartite mo	2.61205164	7.15E-06	0.00014694	1.72320932	1.80159089
TRIOBP	TRIO and F-a	-1.6150429	4.19E-05	0.00063507	209.309821	210.253846
TRMT61A	tRNA methyl	1.61497212	0.00030693	0.00312885	23.1431019	18.6710329
TRO	trophinin	1.71931034	0.02162745	0.07999295	1.56291078	1.37166579
TRPM2	transient rec	1.84336452	1.10E-05	0.00021078	8.73627052	10.2158392
TRPM8	transient rec	-1.8348799	0.030402	0.10134711	2.02376909	1.92442663
TSGA10	testis specifi	4.65322778	5.01E-05	0.00073523	0.20037318	0.38897985
TSHZ1	teashirt zinc	-1.9450933	7.91E-05	0.00106267	62.9171776	58.654067
TSPAN1	tetraspanin 1	1.68199682	0.02086016	0.07786226	1.46272419	1.84253614
TSPAN10	tetraspanin 1	1.83736381	0.00047069	0.00442914	4.62862039	3.95121638
TSPAN11	tetraspanin 1	2.15580838	1.63E-11	2.04E-09	9.51772591	9.88827727
TSPAN14	tetraspanin 1	-1.5026037	0.00013148	0.00160152	222.494376	218.954711
TSPAN33	tetraspanin 3	1.53459717	0.00306801	0.01884575	5.04940406	5.32288217
TSPYL2	TSPY-like 2	1.57345472	8.41E-05	0.00111745	11.6617189	11.5465598
TSSC4	tumor suppre	1.56052847	0.00015703	0.00185093	27.3910133	27.4333158
TTC1	tetratricopep	1.54543997	6.94E-06	0.00014345	72.0141198	72.80065
TTC3P1	tetratricopep	1.73233286	0.02177437	0.08039171	1.40261224	1.53544678
TTPAL	tocopherol (a	1.51181071	4.83E-07	1.56E-05	51.215384	56.5658594
TUBB	tubulin; beta	-1.5082157	2.09E-05	0.00035919	1341.73887	1305.31402
TUBB2A	tubulin; beta	1.5853681	8.79E-05	0.00115701	18.8751533	17.6064564
TUBB2B	tubulin; beta	-1.726179	0.00208112	0.01393708	7.15332242	8.16857687
TUSC2	tumor suppre	1.5814301	0.00022158	0.00243084	30.9376185	31.8554025
TXNDC17	thioredoxin c	1.64641785	2.68E-05	0.00043934	76.4624043	72.8825405
TYRO3	TYRO3 prote	-1.8789925	6.80E-08	2.88E-06	41.2167625	37.8948265
TYW1B	tRNA-yW syr	1.63646706	0.00031751	0.00321593	5.37000114	5.32288217
UACA	uveal autoan	-1.7508128	1.58E-07	5.96E-06	119.382339	123.142832
UBA7	ubiquitin-like	-2.202612	5.14E-06	0.0001109	10.5195918	7.82054227
UBE2C	ubiquitin-cor	-2.1127014	1.06E-09	8.28E-08	107.299836	107.337966
UBE2QL1	ubiquitin-cor	2.23271984	0.0157032	0.06335967	0.66123148	0.47087035
UCA1	urothelial ca	4.69931248	1.42E-10	1.40E-08	115.094353	113.930151
UCKL1-AS1	UCKL1 antise	2.74378192	0.00051483	0.00476107	0.80149271	0.83937757
UCN2	urocortin 2	-1.7638276	4.24E-06	9.51E-05	30.1160885	32.3262729
UHRF1	ubiquitin-like	-1.5809618	4.38E-05	0.00065802	144.709508	141.895755
UNC5B-AS1	UNC5B antis	6.89514325	0.00014069	0.00170045	0.14026122	0.10236312
UNC80	unc-80 homo	-2.5079076	0.01173906	0.05119135	1.08201516	1.06457643
UPK1A-AS1	UPK1A antise	-4.2611316	3.07E-05	0.00049247	2.20410495	2.08820762
UPK3B	uroplakin 3B	-2.231009	7.29E-05	0.00099037	9.29731541	6.9811647
UQCRHL	ubiquinol-cyt	1.7481585	0.0029276	0.01813966	4.08761281	3.84885326
USP43	ubiquitin spe	-1.5921097	0.00070991	0.00607261	9.63794981	9.04889969
VANGL2	VANGL plana	-1.518477	0.00062554	0.00552331	14.066197	16.1529001
VCL	vinculin	-1.5912911	0.00015438	0.00183185	271.866327	282.174173

VCX	variable char	1.84107257	0.03451132	0.11104557	1.00186589	1.76064564
VDR	vitamin D (1;	-1.5737128	2.15E-07	7.68E-06	50.2335555	52.8603145
VGF	VGF nerve gı	2.333326	6.48E-20	3.55E-17	336.646975	335.648666
VIM	vimentin	-1.5538982	9.67E-06	0.00018831	3682.23784	3643.67663
VIT	vitrin	-7.5718917	0.00035788	0.00354176	1.42264956	1.35119317
VLDLR	very low den	-1.7860927	6.99E-06	0.00014421	18.795004	21.1891656
VPS33A	vacuolar prot	1.50343064	1.34E-06	3.59E-05	36.5681048	38.8775125
VPS37D	vacuolar prot	-1.6132075	0.00314374	0.01919626	5.73067286	5.36382742
VSNL1	visinin-like 1	-1.5691811	0.00327467	0.01975757	6.43197898	7.20636356
VWA1	von Willebra	-1.6387242	0.01034644	0.0466015	4.74884429	3.82838064
VWA5B2	von Willebra	2.7793537	0.0128269	0.05479502	0.3406344	0.20472624
VWCE	von Willebra	1.93786349	0.03170174	0.10453064	1.04194052	0.65512396
WDFY3-AS2	WDFY3 antis	-1.775307	0.03816968	0.11928863	1.38257492	1.59686465
WDHD1	WD repeat a	-1.8555439	0.00017265	0.00200005	20.2577282	23.5435173
WDR25	WD repeat d	1.56539844	0.00271183	0.01711167	5.61044896	5.85517039
WDR63	WD repeat d	2.51281597	0.00201886	0.01359679	0.64119417	0.79843233
WDR66	WD repeat d	2.42097908	0.03692942	0.11648438	0.66123148	0.55276084
WDR76	WD repeat d	-1.7113266	1.12E-06	3.12E-05	23.6239976	23.2773732
WHSC1	Wolf-Hirschh	-1.6310511	5.27E-05	0.00076217	225.580123	232.937513
WIPF1	WAS/WASL i	-1.6668512	7.77E-08	3.25E-06	62.8771029	62.5848108
WNT16	wingless-typ	-2.8361418	0.00675526	0.03416224	0.78145539	1.2078848
WNT5A	wingless-typ	-1.5046536	0.00343817	0.02046538	139.860478	139.541403
WNT9A	wingless-typ	2.82309277	2.14E-06	5.37E-05	2.70503789	2.60002322
WSCD1	WSC domain	1.59909299	8.99E-08	3.67E-06	18.3942576	18.2001625
WWC1	WW and C2 (	1.73564845	2.90E-07	1.00E-05	12.6836221	12.7749172
WWC3	WWC family	-1.5614973	4.19E-05	0.00063507	60.9735578	64.3454564
WWTR1	WW domain	-1.6623755	1.27E-05	0.0002389	605.748151	640.834068
XG	Xg blood grou	1.78240278	5.51E-05	0.00079153	4.70876966	4.52444985
XPC	xeroderma p	1.59403578	7.36E-09	4.30E-07	74.8594189	80.8054459
XRCC2	X-ray repair (	-1.588075	0.0154145	0.06256682	15.3485854	15.9686465
YJEFN3	YjeF N-termi	1.54927456	0.02252296	0.08209071	2.32432885	2.00631713
YRDC	yrdC N(6)-thı	1.55608225	1.03E-05	0.00019989	54.1408324	54.0886719
ZAK	sterile alpha	-1.5698781	1.90E-06	4.86E-05	34.5844104	36.0318178
ZBTB11-AS1	ZBTB11 antis	1.58224957	2.98E-05	0.00048104	7.97485244	8.31188524
ZBTB12	zinc finger aı	-1.6632136	1.30E-05	0.00024167	14.1062717	14.3922545
ZBTB37	zinc finger aı	1.86881302	0.02105461	0.07843092	2.76514984	1.76064564
ZBTB46	zinc finger aı	-1.8146806	0.00136063	0.01015732	10.8201516	10.0315856
ZC2HC1C	zinc finger; C	2.21328648	0.03643436	0.11535794	0.42078367	0.40945247
ZC3H10	zinc finger C(	1.5266799	0.00024187	0.0026113	8.87653174	8.76228296
ZCCHC24	zinc finger; C	-2.2597554	2.33E-09	1.59E-07	169.395484	172.666109
ZEB1-AS1	ZEB1 antiser	-1.5493289	0.02248724	0.08200247	4.04753818	3.86932589
ZFP36	ZFP36 ring fi	1.67793528	0.00033325	0.00333973	10.3993679	11.8331765
ZFP42	ZFP42 zinc fi	1.58614723	2.67E-05	0.00043927	14.2064582	13.1229518

ZFP69B	ZFP69 zinc fi	1.61454306	0.00819564	0.03957846	4.38817258	4.9134297
ZIC2	Zic family m	-1.7191594	0.00101533	0.00804302	11.5014204	11.1985252
ZMAT3	zinc finger; n	1.79121546	3.00E-10	2.71E-08	34.1636267	37.4034836
ZMIZ1	zinc finger; M	-1.6104383	0.00059997	0.00536395	142.044545	143.267421
ZMYM3	zinc finger; M	-1.5241224	0.00025457	0.00271552	73.3365828	75.4416185
ZMYND8	zinc finger; M	-1.6020704	1.67E-06	4.35E-05	203.999932	200.693131
ZNF10	zinc finger pr	1.97974036	2.24E-05	0.00038205	3.1658962	3.48034604
ZNF114	zinc finger pr	26.5627238	5.52E-09	3.39E-07	0.16029854	0.20472624
ZNF195	zinc finger pr	1.57086763	0.00034169	0.00340761	10.8401889	10.5434012
ZNF208	zinc finger pr	1.61523622	0.04921323	0.14342286	2.42451544	2.55907797
ZNF213-AS1	ZNF213 antis	1.9060057	0.03433916	0.11069155	1.20223906	1.35119317
ZNF219	zinc finger pr	1.53142937	0.00016621	0.00194308	20.0974297	19.2647389
ZNF222	zinc finger pr	1.68526141	0.00954109	0.04391222	2.72507521	3.13231143
ZNF235	zinc finger pr	1.65648063	0.0295674	0.09954288	2.72507521	3.33703767
ZNF28	zinc finger pr	1.59031005	0.00091503	0.00743686	7.0130612	7.04258257
ZNF280A	zinc finger pr	4.33521052	0.00863609	0.04092107	0.12022391	0.06141787
ZNF30	zinc finger pr	2.15472807	1.73E-05	0.00030757	3.56664255	3.74649015
ZNF35	zinc finger pr	1.5994921	0.00052213	0.0048161	6.97298656	7.39061717
ZNF362	zinc finger pr	-1.5979995	0.00020345	0.00228051	18.1137352	16.214318
ZNF395	zinc finger pr	-2.6079234	1.28E-14	3.15E-12	73.9577396	80.7645007
ZNF416	zinc finger pr	1.5525034	0.02102569	0.07835699	4.38817258	4.93390232
ZNF419	zinc finger pr	1.52166093	0.00864418	0.04092107	5.63048627	5.99847876
ZNF420	zinc finger pr	1.50387872	0.00669982	0.03397836	5.26981456	5.09768331
ZNF425	zinc finger pr	1.5294576	0.00924203	0.04280421	4.26794867	4.4835046
ZNF433	zinc finger pr	1.63999322	0.02238215	0.08179142	2.90541107	2.43624223
ZNF441	zinc finger pr	1.78927988	0.0015509	0.01115695	5.95108336	4.44255935
ZNF442	zinc finger pr	3.12694979	0.02172101	0.08025567	0.28052245	0.4299251
ZNF460	zinc finger pr	1.83092661	0.01828637	0.07056794	1.54287346	2.31340648
ZNF467	zinc finger pr	-2.4939013	7.20E-07	2.16E-05	12.0624653	13.65524
ZNF469	zinc finger pr	-2.173455	3.41E-05	0.00053953	13.625376	12.5292457
ZNF480	zinc finger pr	1.60156725	0.00019955	0.00224628	9.9585469	10.9323811
ZNF496	zinc finger pr	-1.5133442	0.00029049	0.00299829	35.9269106	38.0995528
ZNF503-AS2	ZNF503 antis	1.56536363	0.00576483	0.03023726	3.30615742	3.39845554
ZNF512	zinc finger pr	-1.5336644	2.29E-05	0.00038918	18.8350786	18.9576496
ZNF528	zinc finger pr	1.83138502	0.00229848	0.01506702	1.703172	1.92442663
ZNF540	zinc finger pr	3.61577499	0.00075902	0.00640049	0.26048513	0.40945247
ZNF556	zinc finger pr	2.47740888	0.00014855	0.00177715	0.9016793	0.90079544
ZNF559-ZNF	ZNF559-ZNF	4.20921743	0.01258792	0.05401698	0.3406344	0.26614411
ZNF561-AS1	ZNF561 antis	2.24447626	0.00015255	0.00181618	2.32432885	2.25198861
ZNF563	zinc finger pr	1.73598107	0.02851545	0.09713556	1.60298542	1.53544678
ZNF569	zinc finger pr	1.51227025	0.00816642	0.03945071	7.09321047	6.9811647
ZNF570	zinc finger pr	1.70138415	0.01580905	0.06364261	2.42451544	2.37482435
ZNF582	zinc finger pr	1.75137149	0.01639422	0.06529739	1.54287346	1.53544678

ZNF583	zinc finger pı	1.68146259	0.03816689	0.11928863	1.32246297	1.47402891
ZNF585B	zinc finger pı	1.60794601	0.00230784	0.01509364	6.81268802	6.91974683
ZNF593	zinc finger pı	1.62992599	0.00553115	0.02938177	14.1864209	13.3686233
ZNF608	zinc finger pı	-1.5124194	0.00262071	0.01668803	17.0717947	16.6647157
ZNF684	zinc finger pı	1.60500867	0.03769741	0.11803739	2.74511252	2.66144109
ZNF773	zinc finger pı	1.77001633	0.00099104	0.00790725	4.80895625	4.79059396
ZNF79	zinc finger pı	3.35860613	5.60E-08	2.44E-06	5.00932943	5.50713579
ZNF790-AS1	ZNF790 antis	2.3696122	0.00422004	0.02389343	0.98182857	0.83937757
ZNF792	zinc finger pı	-1.6048727	0.01803064	0.06992099	5.79078482	5.24099168
ZNF81	zinc finger pı	1.59334788	0.04782681	0.14035828	6.89283729	6.48982173
ZNF816	zinc finger pı	1.57917434	0.00265538	0.01684282	4.1877994	4.31972361
ZNF827	zinc finger pı	-1.6111169	0.0001058	0.00134747	43.4208675	43.4019623
ZNF844	zinc finger pı	1.96063069	0.00079898	0.00665472	3.18593351	3.76696277
ZNF845	zinc finger pı	1.50630736	0.03067297	0.10194041	5.67056091	5.95753351
ZNF850	zinc finger pı	1.77801645	0.00603264	0.03136177	2.30429154	2.27246124
ZNF853	zinc finger pı	-1.6402643	9.38E-05	0.00122232	9.85836031	11.1575799
ZP1	zona pellucic	-3.6661858	1.46E-05	0.00026698	2.58481398	2.90711257
ZP4	zona pellucic	-8.5283929	0.00017662	0.00203022	1.42264956	1.14646693
ZSCAN16	zinc finger aı	1.50324553	0.02373863	0.08534288	4.02750086	3.72601752
ZSCAN4	zinc finger aı	19.7431353	3.56E-07	1.19E-05	0.14026122	0.06141787

A3	B1	B2	B3	C1	C2	C3
5.03651929	6.4413187	8.76763847	6.90698171	7.59653322	9.31133955	7.47099229
0.58837842	1.38028258	1.89257394	2.19301793	0.83939594	3.96021703	1.18717138
3.50673539	4.33803096	4.01689164	4.69346828	6.06463564	6.93520933	7.53239771
74.6534541	61.1925277	57.974561	57.9817545	33.9115958	37.7283115	41.1416288
8.09608708	14.1095553	15.2564634	13.424549	11.8564676	13.9476912	14.4098043
10.4496008	15.7308396	17.3421572	16.8063056	16.9977677	14.6817802	19.6702016
6.30741668	10.5164387	10.3512208	9.87882844	11.3318451	17.6567725	18.2169401
31.8901104	50.3474503	49.8828418	49.8245476	77.0985167	57.587351	62.7563353
80.4430978	54.4663888	58.4380485	59.1909886	26.5039267	25.5578884	29.1471042
0.37656219	0.70109591	0.75316718	0.51238737	1.3640184	1.02386099	0.90061277
6.4486275	3.00156688	4.15207549	3.99662146	3.33659884	2.87840164	4.60540621
0.42363246	0.98591613	0.88835103	1.12725221	0.96530533	1.71931373	1.06436055
67.0986752	49.2958064	45.2286549	47.7135116	33.1561395	41.031712	31.7466001
3.05956779	2.25665247	1.46771041	2.07004496	0.81841104	0.63749835	1.28951374
107.908602	132.15658	129.139204	134.081526	154.364913	238.752791	235.981014
121.912009	94.4945836	88.2364321	88.1716181	46.1877614	84.3622817	91.2484483
3.64794621	0.87636989	1.71876613	1.06576572	1.04924492	0.52158956	0.28655861
1.9769515	7.2738701	6.52744891	6.82499973	5.162285	12.6533764	9.5178395
0.72958924	1.94992301	2.06638176	1.5371621	5.05736051	2.83976538	2.84511761
1.3415028	2.8262929	3.39890831	2.58243233	6.08562053	5.94998461	5.13758648
2.80068129	1.57746581	1.52564634	2.56193684	2.51818781	1.08181538	1.28951374
12.0970603	9.17997462	9.42424576	7.76779248	4.97342092	8.42270548	7.98270409
33.7964565	23.9029888	21.4749207	23.8977468	26.5458965	17.4635912	16.5794623
22.4289854	56.7449505	54.4018449	53.0423402	74.4334346	62.2237027	59.0924788
0.51777301	1.2488271	0.69523124	0.43040539	1.65780697	0.73408901	0.85967583
0.82372979	0.52582194	0.63729531	1.25022518	2.68606699	2.27953956	2.74277525
10.6378819	12.9483652	15.4495832	14.2648643	20.3553514	15.8601862	21.3895533
340.765247	299.061226	300.571641	295.893457	83.016258	126.630354	119.35166
192.164392	142.03765	147.910447	149.555625	78.1897314	113.43607	126.556563
2.37704882	0.61345892	0.88835103	0.7583333	0.46166776	0.44431703	0.22515319
2.235838	0.46009419	1.87326196	0.69684682	0.18886409	0.88863406	1.3918561
3.34198943	2.34428946	2.89679685	1.88558551	1.48992779	2.78181098	2.10825262
54.9310094	34.1565165	31.4978378	29.7594583	13.010637	17.1158648	17.9303815
3.36552457	1.86228602	2.3946854	1.76261254	1.69977677	2.00908571	0.92108124
1.88281095	3.52738881	2.0277578	2.7054053	2.20341433	3.80567197	3.62291955
18.8281095	11.0422606	11.2009478	12.3792788	5.91774135	6.9158912	7.08209132
33.5611052	21.0328774	21.0886811	20.0445938	21.0268682	14.8363253	17.561949
3.67148135	5.95931526	6.43088901	5.84121598	3.8612213	8.1329335	6.63178494
58.8378421	44.9577754	45.981822	43.3069803	25.5805911	23.4328939	23.0474995
263.405252	185.637052	182.363018	189.480848	117.263612	133.526927	124.857679

9.55526556	6.26604473	6.73988068	5.20585565	1.53189758	2.91703791	2.53809053
77.4541354	115.374096	122.824187	118.750896	178.161787	140.558727	142.337755
21.8641421	10.6259849	12.6686582	12.7277022	17.5853449	14.5465533	14.5530836
21.2286934	12.7511819	14.3488004	14.1213958	10.9331321	7.59202581	7.55286618
3.3184543	7.01095913	5.21423434	4.85743224	8.83464222	5.1579412	3.78666733
5.8131788	7.82160128	6.85575255	8.0752249	17.1866318	12.8658758	12.5267049
197.130306	130.601023	134.585182	134.757878	95.8800007	96.8224768	96.4269718
90.3278552	137.809165	145.940625	138.201121	196.334709	183.251799	175.251058
307.83959	152.072085	165.001549	161.279048	61.2759033	108.046311	107.131983
9.69647638	5.52113032	4.40313122	4.59099081	1.86765596	3.4579456	5.99726231
9.03749255	5.67449505	6.27639318	6.31261236	3.52546293	5.11930494	3.92994663
28.2892345	17.6588533	14.9667837	16.7243237	10.5134341	8.80906812	8.43301048
26.6888452	32.0970473	36.0554648	31.8499987	61.6116617	40.6646675	45.5014133
91.9047094	55.3208494	51.8140397	50.8288268	17.354511	27.7794736	31.1530144
25.1825964	38.6479122	33.5449076	36.7279265	66.3122789	37.5737664	43.0247282
19.8165852	35.3396159	35.9975289	31.9319807	48.2232965	47.3873774	42.3288002
35.3027053	51.6181866	52.3161512	54.026124	75.9863171	53.781679	58.1304606
70.4406646	48.0907978	51.6788559	50.1524755	29.2109786	33.0533236	29.617879
4.51874628	2.97965763	2.97404477	2.60292782	4.57470785	3.3999912	1.5351354
8.51971954	5.34585634	5.04042652	5.34932411	3.50447803	3.65112692	4.66681162
6.56630318	5.49922107	3.47615622	2.72590079	6.77812218	3.67044505	4.09369441
9.34344933	5.63067655	3.8623958	4.48851333	6.82009198	4.53976098	3.33636094
27.347829	15.5993841	19.6595946	17.5851344	18.382771	18.2942709	14.3279304
105.83751	65.2019199	62.6673719	67.450673	56.2605126	41.5146653	38.9719708
20.0284015	15.6212933	12.7072822	12.5842337	6.92501647	10.4704275	12.8746689
1.60038931	0.32863871	2.00844582	1.5576576	5.62395277	3.67044505	2.08778415
38.9506515	22.3693415	26.3029154	26.9105845	14.0179121	20.5931285	15.2285432
1.22382712	0.61345892	1.5642703	1.59864859	4.51175315	3.38067307	3.50010872
10.661417	32.0970473	24.4296535	23.9797288	36.0730403	29.88515	32.4220597
0.21181623	1.2488271	0.61798333	1.04527023	0.18886409	2.06704011	0.42983791
65.5453561	48.4413458	54.3632209	51.6281511	39.0948657	32.241962	26.711356
0.37656219	0.56964043	0.79179114	0.57387385	1.17515431	1.00454286	1.28951374
32.855051	20.9233312	20.1810181	26.1932422	12.6329088	15.3385967	15.5355703
263.805349	194.269296	198.430584	207.98828	97.3489436	159.818905	154.659775
5.46015175	9.09233763	7.51235984	7.00945918	17.6902693	9.85224724	13.6115339
2.14169745	1.11737161	0.88835103	0.96328825	1.65780697	0.56022582	1.35091915
60.5794423	24.8231772	30.9957263	21.376801	37.6678926	30.329467	32.3197173
1.29443253	1.68701204	3.64996403	3.64819805	1.61583718	3.24544615	1.78075707
7.06054106	4.60094193	3.68858799	6.10765741	2.11947474	4.61703351	4.72821704
2.49472451	1.75273978	1.00422291	1.72162155	0.56659226	1.62272307	1.73982012
185.174457	104.068925	112.086726	112.766212	51.5389104	67.1305081	63.8206958
15.5096552	23.1799836	20.6638176	22.9754495	29.1480239	30.484012	26.8751038
10.6849521	18.6885879	18.9836754	17.4211705	35.0657652	30.2715126	33.7115734

3.41259484	7.18623311	9.26974993	8.95653118	7.7853973	11.7840604	9.31315478
2.09462718	2.78247441	4.01689164	2.3159909	2.47621801	4.249989	4.05275746
6.04853017	4.55712344	4.17138747	4.75495477	1.67879187	3.53521813	5.40367662
6.58983832	4.16275699	4.17138747	4.57049531	3.16871966	3.20680989	5.23992884
245.683294	167.540014	172.243541	170.50202	67.2565993	105.052001	113.86611
53.8013229	33.1925097	32.2703169	35.9695932	12.6748786	19.1249505	18.2578771
119.770311	87.7684447	86.7880337	82.6378346	42.7672229	75.6884405	79.9907887
1.43564335	2.49765419	5.58116194	3.79166652	2.77000659	2.39544835	4.03228899
1.27089739	0.63536817	0.42486354	0.61486484	0.0209849	0.3670445	0.10234236
1.41210821	2.47574494	2.43330936	1.59864859	6.10660543	4.09544395	3.74573038
0.30595678	2.14710623	0.86903906	0.59436935	1.74174657	2.24090329	1.33045068
2.37704882	3.96557376	3.2251005	4.03761245	4.78455683	6.02725713	5.05571259
6.56630318	9.15806537	8.96075826	10.1657654	10.744268	12.1897412	12.3424886
21.6523259	29.3145729	29.8563196	26.9105845	49.1886018	38.2305829	41.2439712
10.9909089	19.6087763	21.0307452	20.6389631	23.125358	23.5294846	21.9217336
22.5466611	12.312997	13.1128338	13.4450445	6.44236381	9.63974779	9.68158727
29.6307373	16.6291187	16.9752296	14.9207201	12.5069994	12.4215588	9.16987547
46.5525007	32.250412	27.8285618	29.4315304	21.2996719	20.515856	17.7871022
206.426685	106.413214	111.603927	112.417788	59.3033229	49.1260093	46.3406207
1.0826163	1.97183226	2.43330936	3.0743242	1.63682207	1.6999956	2.61996442
6.16620586	4.27230322	4.90524267	5.28783763	1.90962575	3.18749175	3.25448705
10.143644	18.5352232	16.531054	18.4459452	25.0559687	24.3988005	21.5737695
1.29443253	6.76995741	7.78272754	7.37837808	35.4854632	26.1374324	30.7845819
19.8165852	35.7120731	33.5449076	37.1788273	95.9219705	71.2645884	67.9757956
92.3283419	65.6401049	72.690289	71.9801773	29.8615104	52.0430472	50.7822791
66.7927184	49.0548047	44.1278721	43.3889622	32.2537888	36.1055884	43.6387824
12.5912982	7.71205505	9.3856218	9.01801766	5.30917929	5.98862087	7.3891184
39.727311	32.2065935	29.3542081	28.6117106	17.6692844	21.1147181	23.1907788
3.83622731	0.87636989	2.89679685	2.09054046	1.17515431	1.12045165	1.9035679
2.70654074	1.40219183	1.27459062	2.00855848	0.98629022	1.25567857	1.43279304
2.77714615	1.6212843	1.04284687	1.06576572	1.80470126	1.10113351	0.8801443
15.5331903	10.9984421	12.3596666	11.8668914	6.42137891	8.17156977	9.76346116
15.0389525	11.8090843	11.2009478	12.1538283	7.51259362	6.45225603	7.02068591
54.3190959	33.8497871	37.6583591	31.1941429	19.8517139	19.9556302	22.679067
10.2142494	13.2989131	16.6855499	15.1051796	21.5095209	20.7476736	18.5239672
0.77665952	1.64319355	1.54495832	1.96756749	2.79099149	2.26022143	1.57607235
1.9769515	2.51956344	3.36028435	3.52522508	2.05652004	3.86362637	3.60245108
202.896415	146.353772	139.277993	144.759679	120.411347	117.724695	127.39577
21.7229313	14.4162847	13.0742098	13.8549544	10.7022982	11.5329247	11.3395335
9.48466015	14.26292	14.9088478	13.9164409	20.1874723	19.6465401	19.6906701
36.5265324	28.701114	25.2793805	30.4768006	11.2479055	18.9704055	20.9801838
53.9660688	34.1346073	33.8925232	39.8637372	28.0987789	34.4828653	37.7847994
2.8712867	5.58685806	4.59625101	4.09909894	6.75713728	10.4124731	7.59380313

1.15322171	0.85446064	1.91188592	2.02905397	2.07750494	2.53067527	2.23106345
23.7704882	18.1408568	18.9064275	17.7081074	8.89759692	17.4249549	16.3952461
4.44814087	1.53364731	1.5642703	1.63963957	0.60856205	0.50227143	0.47077486
3.15370834	4.11893849	5.61978589	4.54999982	8.07918588	7.3408901	6.09960467
7.08407619	3.33020559	3.5920281	3.93513498	2.89591598	1.85454066	1.96497332
72.6294323	121.442958	129.00402	125.350445	76.1541963	136.424647	147.35253
65.7571724	44.2785888	47.6619642	47.2216197	42.0957062	32.0487807	29.1880411
23.5351369	16.7386649	16.1641264	17.4621615	9.90487204	13.1749659	13.2635699
0.84726493	1.31455484	1.25527864	0.86081078	1.23810901	1.17840604	1.35091915
37.3502622	42.8544877	39.2805653	44.3112595	79.931478	76.1520757	72.7449496
270.583468	205.705923	197.368426	203.02837	109.730034	158.041637	155.703667
168.911677	103.937469	107.258731	108.277698	30.8897704	56.5248538	62.5925875
1.71806499	0.10954624	0.67591927	0.65585583	1.07022982	0.15454505	0.28655861
5.29540579	11.874812	9.44355774	10.2477473	16.2423114	14.4499626	13.6729393
94.6583204	82.7293178	69.3879406	75.0545015	35.4434934	56.8146257	52.3583515
46.434825	81.2175797	78.252139	70.9758981	124.545372	90.4668114	89.8361238
2.77714615	26.5101892	22.073592	26.1317557	117.72528	101.478147	108.728523
69.8522862	93.9249431	94.9183769	89.4013478	177.007618	117.801968	123.875193
8.44911413	15.6870211	16.8207337	16.4578822	28.0148394	23.0272132	19.9567602
11.8852441	2.38810796	3.03198071	2.47995486	2.43424821	1.13976978	0.98248666
9.72001152	7.88732903	9.1152541	9.01801766	3.67235722	5.31248626	5.21946037
91.0103742	139.518087	118.208624	135.249769	253.05689	135.36215	156.051631
30.3838617	48.5728013	40.3041002	48.9637368	99.342509	47.7351038	62.5925875
91.99885	128.93592	123.847721	121.066887	216.606121	143.610992	134.252708
32.3608132	46.4695135	45.8852621	45.6844576	71.0338811	51.6373664	50.3729097
1.92988122	3.26447785	2.62642915	2.2954954	5.72887726	4.3079434	3.58198261
3.10663806	1.38028258	0.94628697	1.98806298	0.98629022	1.04317912	0.69592805
78.25433	43.7089484	46.3487496	47.5700432	18.9073935	26.3885681	31.828474
4.40107059	1.77464903	1.9311979	2.25450441	3.12674986	2.70453846	3.09073928
24.1941207	18.3161307	16.7627978	16.7038282	7.13486545	14.4885989	15.1262008
1.90634609	2.30047097	2.25950155	2.58243233	3.44152334	4.19203461	6.12007314
57.0727069	38.450729	43.258833	36.543467	37.2901644	28.6101533	24.7054457
31.3252672	48.3537088	50.0759616	48.102926	113.234512	75.8043493	68.5079759
1.24736225	0.48200344	1.21665468	0.69684682	0.56659226	0.32840824	0.47077486
22.2642395	14.4820125	17.7283967	17.0932426	6.44236381	11.9386055	11.7489029
73.264881	28.9421157	32.7531164	27.5664403	14.7943534	12.0351961	15.4741649
2.47118937	1.22691785	1.17803072	1.08626122	0.65053185	1.12045165	1.0234236
50.3651929	27.7590163	29.412144	26.0702692	20.1035327	14.0829181	14.7577683
0.07060541	8.82942666	0	0.14346846	0.0419698	5.25453186	0.12281083
40.9982084	32.9734172	30.6094867	30.2308547	11.8354827	22.5249417	22.0445444
21.2757637	32.338049	29.0645284	29.6159898	46.9851875	35.7192258	31.2758253
8.40204386	6.50704645	5.83221766	4.6114863	4.25993437	4.7329423	4.44165843
13.5091686	5.78404129	10.6409004	7.80878347	9.1074459	5.40907691	4.03228899

101.954213	240.191078	225.660475	217.744135	350.762577	229.847133	232.132941
12.9678604	24.275446	31.0150383	24.0002243	51.2870917	41.4180747	38.5421328
63.4271938	38.2535458	37.774231	34.80135	29.6726463	24.6306181	23.027031
38.009246	20.7918757	25.5690602	27.4229719	25.5386213	18.0624533	19.1380214
1.03554602	0.74491441	0.59867135	1.08626122	0	0.30909011	0.81873888
0.47070274	4.16275699	2.14362967	3.40225212	6.4843336	3.4579456	5.46508203
2.02402177	0.81064215	0.86903906	1.43468463	0.50363756	0.25113571	0.73686499
0.47070274	1.11737161	1.39046249	0.88130627	1.61583718	1.56476868	2.06731568
88.2567632	17.5054886	18.0567004	19.2862605	11.8354827	12.460195	11.9331192
10.3319251	6.81377591	6.58538485	6.49707181	7.28175974	8.07497911	6.14054161
11.0850495	6.68232042	7.99515931	6.78400874	9.96782674	6.78066428	7.26630757
1.95341636	0.81064215	0.77247916	1.57815309	1.15416941	0.96590659	1.04389207
31.8901104	59.9217913	52.8568866	57.6128355	98.2093245	71.4384515	79.4586084
7.03700592	3.70266279	4.57693903	4.59099081	2.26636903	3.63180879	5.13758648
13.8621956	7.40532559	9.28906191	9.28445909	3.9031911	2.74317472	4.4621269
16.9688337	10.6478942	11.8961791	10.0222969	3.8612213	9.4658846	8.96519075
4.51874628	5.52113032	6.68194474	6.37409885	8.05820098	8.05566098	7.14349674
4.42460573	0.67918667	1.23596666	1.31171166	0.16787919	0.17386319	0.1432793
226.408017	155.511837	146.655169	150.149994	126.643862	102.695189	107.909785
117.510938	79.5524769	71.6667541	78.5592311	59.7649906	58.9396202	66.0722277
2.84775156	1.51173806	2.87748487	1.37319814	1.3010637	2.14431263	1.65794623
20.7109204	29.6870301	29.0065925	29.7799538	66.1234148	31.1601467	42.1241154
20.7109204	29.6870301	29.0065925	29.7799538	66.1234148	31.1601467	42.1241154
1.95341636	1.00782538	1.64151822	0.7788288	0.79742614	0.86931593	1.51466693
7.50770866	9.88107053	8.43933483	8.21869337	14.5215497	12.0931505	16.1086875
3.55380567	6.17840774	4.59625101	3.93513498	4.82652663	5.00339615	6.61131647
22.5701962	15.8403858	18.5201879	18.6509002	8.41494425	11.8613329	14.5121467
40.2921543	55.9123991	49.1876106	53.3702681	102.343349	61.547568	64.9873987
14.097547	29.5993931	24.873829	22.1351343	88.4933165	21.3851719	21.7989227
2.14169745	3.39593333	2.33674946	2.76689178	2.79099149	3.18749175	4.70774857
5.95438962	1.48982882	1.83463801	1.76261254	3.65137232	1.04317912	1.33045068
114.616116	74.1189836	72.1688656	73.291889	41.8648723	59.4032554	61.6715062
21.0874826	70.613504	71.0680828	74.8290511	101.27312	104.742911	97.2661791
0.35302705	1.31455484	0.77247916	0.81981979	1.74174657	0.8499978	1.20763985
63.8272912	48.3098903	45.4990226	51.3207187	30.8897704	36.2214972	35.0010872
85.9267847	68.7293088	62.7059959	61.9988714	27.9308998	47.0976054	47.7529453
8.66093036	0.98591613	0.61798333	0.92229726	0.71348655	0.54090769	0.96201819
1.90634609	2.8701114	3.99757966	2.60292782	5.85478665	4.75226043	5.07618106
1.41210821	2.38810796	2.06638176	2.25450441	6.31645442	4.44317032	3.72526191
6.96640051	12.9702744	8.53589473	7.84977446	22.6217205	11.436334	12.1378039
0.98847575	2.62910968	1.44839843	3.01283772	3.08478006	2.47272088	2.9269915
4.94237874	2.69483742	2.761613	2.50045035	1.57386738	2.00908571	2.70183831
11.3910062	4.42566795	4.59625101	5.28783763	8.26804997	3.76703571	3.37729789

1.7886704	1.35837333	0.75316718	1.29121616	0.33575837	0.88863406	0.45030638
15.3919795	22.9608912	24.3717175	23.5698189	21.8242943	34.3090021	28.3488338
30.6427482	46.7543337	47.7199002	45.0081063	79.51178	44.9919291	53.6478652
28.1480237	18.0532198	22.3632717	25.2709449	18.2148918	13.5613286	18.2578771
1.76513526	2.80438365	3.70789997	2.62342332	5.07834541	3.12953736	2.96792845
19.3458825	25.6557286	24.4103415	19.0813055	45.3483654	31.4692368	32.3401858
14.450574	22.2817045	19.0029874	19.7166659	43.3338152	19.2215412	23.067968
4.18925436	1.84037677	3.2251005	2.04954947	0.92333553	2.08635824	2.16965804
4.44814087	1.9937415	2.16294165	2.27499991	3.39955354	1.39090549	1.12576596
2.70654074	0.81064215	1.23596666	1.27072067	1.86765596	0.96590659	0.8801443
37.3973325	22.9170727	24.2944696	26.1727467	11.3947998	14.7010983	13.0588852
55.2369662	35.4053436	38.0639106	38.7569804	24.9300593	27.547656	24.2346709
1.64745958	2.71674666	3.43753227	3.83265751	1.97258045	4.59771538	4.58493774
21.5581854	13.0140929	12.1472348	10.5961707	16.0534473	13.2329203	8.84237992
93.1991419	132.309944	130.394482	125.32995	216.396272	141.87236	151.425756
10.0730386	6.4851372	6.75919266	5.69774752	4.86849643	6.14316592	4.81009093
1.74160013	3.15493161	3.05129268	2.58243233	4.38584376	3.36135494	2.64043289
3.20077861	1.2050086	1.73807811	1.35270265	0.48265266	0.3670445	0.67545958
197.506868	99.9280769	94.0879618	97.7020231	46.2297312	56.3896268	52.2764776
3.05956779	3.85602752	5.46529006	6.04617093	8.31001976	6.14316592	9.08800158
13.5562388	7.55869032	6.43088901	6.76351324	4.78455683	5.19657746	4.76915398
0.30595678	0.65727742	0.32830364	0.65585583	2.5601576	0.44431703	2.35387428
24.8766397	33.6526038	35.1671138	31.6245483	61.3808278	39.1578532	42.0217731
5.67196798	3.63693505	4.40313122	4.6319818	1.21712411	2.43408461	1.3918561
0.91787034	1.94992301	1.52564634	1.9265765	2.35030862	2.56931153	2.25153192
6.02499503	10.2097092	8.05309525	6.25112588	10.2406304	11.8806511	11.8103084
0.65898383	1.46791957	1.27459062	1.39369364	1.3640184	1.41022362	1.00295513
16.5216661	9.74961505	11.007828	12.2563058	8.81365732	8.51929614	7.90083021
83.0790331	34.3975183	30.8219185	32.1984221	9.88388714	8.80906812	11.7898399
4.58935169	7.49296257	6.93300047	7.74729699	5.56099807	9.71702032	7.92129868
2.80068129	1.73083054	1.33252655	2.52094585	2.16144453	1.00454286	1.0234236
2.49472451	0.10954624	0.13518385	0.3689189	0.16787919	0.40568077	0.1432793
0.56484328	1.79655828	1.27459062	1.02477473	1.09121472	1.77726813	1.57607235
6.02499503	3.92175527	4.51900309	4.40653136	2.74902169	3.09090109	2.2720004
7.6018492	26.5101892	23.9854779	28.837161	50.8254239	40.3555774	41.9194307
15.5096552	20.0688705	22.4018957	21.3153145	41.3822196	24.6885725	25.4627792
49.870955	31.1111312	31.7488935	29.9439177	28.8332504	19.5306313	21.4509587
2.11816232	0.59154968	0.3669276	0.63536034	0.29378858	0.25113571	0.3684325
52.0597227	36.2598043	36.7506961	38.0396381	24.2375576	30.0203769	32.6267444
62.250437	35.3615251	34.2015148	32.5468456	27.8259753	25.6158428	26.6090136
6.16620586	4.27230322	3.55340414	3.21779266	5.81281685	3.90226263	2.43574817
1.22382712	1.77464903	1.89257394	1.70112606	3.29462905	1.06249725	2.68136984
52.2244687	37.7058146	38.623958	41.7083317	11.8984374	28.4749263	33.4454833

2.98896238	3.30829634	2.91610883	3.05382871	4.28091927	5.77612142	7.06162285
108.285165	80.3412098	77.1899801	84.4209426	60.5414319	56.8918983	54.3847302
442.931276	313.346055	288.405095	302.042105	210.142772	208.326734	203.88645
5.08358956	2.51956344	2.54918123	2.52094585	3.9661458	2.41476648	1.7602886
15.8626822	9.28952085	10.5636525	9.69436898	14.4166252	12.8851939	9.10847006
6.87225996	11.8090843	9.28906191	15.0027021	18.6555747	14.1022362	14.614489
27.2536885	19.7621411	18.0953243	20.2700442	11.1849508	15.976095	14.8805792
176.866553	117.455475	119.34803	125.391436	81.2535266	103.622459	108.50337
14.8036011	13.0579114	11.4326916	11.723423	9.48517407	8.1329335	9.55877644
15.9568228	11.1956254	10.2353489	9.01801766	8.49888385	8.03634285	10.3979838
29.748413	19.4773208	18.1146363	20.8234226	10.0517663	14.9908703	14.6758944
0.37656219	0.70109591	1.02353489	1.37319814	0.90235063	1.31363297	1.61700929
1.36503794	2.76056516	1.50633436	2.50045035	2.03553514	2.14431263	2.80418067
6.51923291	10.363074	7.16474422	6.88648621	13.9549574	9.65906592	11.8717138
0.21181623	0.92018839	1.1007828	0.84031528	2.18242943	1.60340494	2.2720004
7.53124379	11.8309935	10.0229171	8.73108073	13.1785162	14.0829181	11.9535877
0.77665952	3.33020559	1.62220624	2.09054046	2.11947474	1.13976978	1.14623443
30.8310293	12.050086	9.21181399	9.44842305	12.7378333	7.93975219	5.11711801
13.9092659	8.32551397	7.26130411	7.68581051	8.35198956	5.91134834	5.07618106
0.02353514	0.65727742	0.59867135	1.04527023	5.07834541	3.05226483	3.86854121
8.33143845	14.3067385	15.642703	15.412612	19.2641367	17.4056368	14.9215161
73.8061892	51.4867311	45.8852621	51.874097	29.6516614	32.7055972	31.1530144
66.6515076	43.8184946	42.4863538	45.6639621	40.5008539	26.7362945	26.9979146
51.8949768	36.6541707	34.1242669	33.8380617	22.0341433	22.4669873	23.4773374
43.9401005	24.363083	26.2642915	26.2957197	17.8161787	17.2317736	17.6438229
2.44765423	0.59154968	0.98491093	1.49617111	0.46166776	1.29431483	1.04389207
2.30644341	5.14867311	3.45684424	4.30405388	5.2462246	7.39884449	6.61131647
1.71806499	4.27230322	3.1285406	3.34076563	4.49076826	3.30340054	3.21355011
1.71806499	6.94523139	5.75496975	5.69774752	8.75070263	11.3783797	8.06457798
4.30693004	6.70422967	5.34941819	6.78400874	9.42221938	10.4511093	10.6640739
1179.46338	820.019308	822.53581	870.115731	656.008909	710.92657	681.98902
153.237276	312.951688	302.039352	305.649312	275.531716	292.669697	282.587725
58.3906745	83.5399599	84.3740363	83.2936904	131.554328	97.8463378	87.4413125
0.14121082	1.73083054	0.32830364	0.51238737	1.88864086	0.75340714	0.92108124
239.422947	174.682429	175.449329	170.932426	133.296075	120.197416	129.094653
12.5677631	6.37559096	6.3536411	6.21013489	7.99524629	5.48634944	4.13463135
2.40058396	3.94366451	5.94808954	3.60720706	4.15500988	5.66021263	3.88900969
70.1817781	43.9718593	46.966733	48.020944	31.8760607	36.6464961	39.9544574
1.74160013	0.15336473	0.23174375	0.18445945	0.60856205	0.34772637	0.04093694
20.4284988	12.6854542	12.900402	13.9779274	15.7806436	10.4317912	9.59971338
26.2887479	14.8544697	17.7477087	16.8882876	9.23335529	13.2329203	15.740255
24.1470504	17.0453944	19.311979	17.0932426	14.7104138	8.34543295	10.3979838
71.4762106	38.5164567	42.7180976	37.5067553	35.5694028	33.4590043	30.5184918

0.84726493	1.27073634	2.10500571	2.21351343	3.48349313	3.12953736	3.15214469
13.9563362	8.56651569	8.61314264	6.47657632	2.95887067	5.38975878	6.40663175
16.7570174	13.6275518	13.5763213	11.9693689	7.42865403	8.30679669	7.16396521
90.4455309	54.7950275	53.1465663	49.0662143	36.0310705	35.313545	28.7377347
27.7949966	21.4272439	18.6360598	17.441666	13.9969272	15.7635956	12.833732
30.10144	20.4851462	20.8569373	21.5817559	12.3601052	19.2408593	17.9917869
0.68251897	1.55555656	0.98491093	1.37319814	2.14045964	1.85454066	1.16670291
153.378487	536.754649	547.398045	559.690969	913.95528	729.529931	703.071546
55.4252473	35.1205234	36.4223924	35.5391878	41.4241894	35.4680901	35.1034295
23.2997855	10.1658907	11.5099395	8.11621589	6.5263034	3.36135494	4.42118996
47.5645116	22.3912507	26.9788347	24.1641882	25.6435458	18.4488159	19.6702016
13.7209848	8.28169548	8.7483265	9.83783745	7.93229159	6.10452966	9.84533505
50.4593334	30.6948555	30.6094867	30.5997736	19.8936837	26.6010675	30.1500593
0.75312438	1.35837333	1.25527864	1.94707199	2.24538413	2.06704011	3.64338802
70.2759186	44.7167737	44.3016799	44.7621604	44.2151809	33.4976406	31.091609
11.8146387	4.97339914	7.85997546	6.23063038	7.44963893	3.84430824	2.76324372
24.5000775	19.9812335	18.5201879	19.4502245	7.23978994	15.7442774	15.0852639
74.8417352	56.788769	60.4464943	60.8921147	23.2722523	38.539673	36.3929433
8.75507091	14.5696495	16.1255025	16.7448192	24.6992254	17.0192742	16.3747776
20.4991042	16.0594783	11.4520036	13.588513	14.5005648	9.34997581	9.08800158
61.4267072	53.3271079	48.5696272	52.7349078	27.259383	33.4783225	40.4252323
383.387379	226.125341	216.004485	223.072964	81.2115568	130.70648	154.168531
41.1158841	24.2097183	26.708467	26.4801791	27.9308998	22.85335	24.2346709
6.61337346	4.20657548	3.30234841	2.64391881	2.51818781	3.0329467	2.70183831
18.8045743	16.0813875	13.4990733	12.0923419	9.46418917	11.9772417	9.84533505
55.0722202	38.0344533	38.5273981	39.1463948	38.4653188	35.603317	32.7290868
3.83622731	2.16901548	1.62220624	2.21351343	0.48265266	2.27953956	1.16670291
4.37753545	2.10328774	1.89257394	0.94279276	1.00727512	0.75340714	0.71639652
50.1769118	74.0313466	81.5737994	80.834231	93.2778733	88.3031806	80.91187
0.75312438	1.46791957	1.25527864	1.5371621	2.26636903	1.42954176	1.08482902
0.70605411	1.6212843	2.0277578	1.72162155	1.82568616	0.98522472	1.71935165
11.7204982	7.47105333	6.23776922	6.57905379	3.42053844	6.18180219	4.54400079
3.5773408	3.81220903	4.96317861	5.26734213	37.1852399	25.345389	23.9071753
13.5091686	11.1079884	10.9112681	9.87882844	7.07191076	6.52952856	6.63178494
21.2992989	29.6651208	27.5195701	29.8209448	47.9714777	40.7033038	39.7702412
11.8146387	9.11424687	9.1152541	9.71486448	7.57554832	8.34543295	6.09960467
6.42509236	13.2112761	11.7609952	11.7849094	14.668444	10.3352005	11.7898399
11.014444	4.99530838	4.6928109	4.18108091	3.10576496	3.76703571	3.35682941
1.27089739	0.39436645	0.44417552	0.43040539	0	0.61818022	0.5117118
0.4471676	1.02973462	1.98913384	1.43468463	2.6650821	2.02840384	2.37434276
33.8199917	21.6244271	22.8653832	22.1351343	13.3254105	13.4067835	13.6115339
116.616603	84.6573315	81.3034317	75.2184655	49.6082998	48.9521461	49.779324
2.91835697	1.64319355	1.25527864	1.51666661	1.40598819	1.44885989	1.00295513

5.88378421	10.1878	8.76763847	9.20247711	15.696704	14.6045077	15.8630658
4.84823819	3.11111312	2.68436508	2.80788277	3.35758374	4.48180659	3.4387033
16.5922715	39.1737342	40.2461643	39.2283768	26.839685	32.976051	35.1034295
1.76513526	2.30047097	4.48037913	4.03761245	3.81925151	3.67044505	4.66681162
78.7721031	50.3693595	52.3161512	52.7144123	24.3844519	42.5771626	39.9135205
25.7709749	37.2676296	36.4223924	37.8141877	64.444623	60.6202977	53.9548923
107.53204	64.3255501	61.3155334	50.3779259	40.7736576	27.991973	29.2085096
55.4017122	37.3333574	33.5255956	40.3761245	21.6354302	25.345389	26.1382388
0	1.13928086	1.87326196	1.78310804	2.03553514	2.16363077	2.0468472
36.5029973	23.5305316	24.893141	25.3324314	26.2940777	14.797689	16.4157146
13.250282	6.66041118	6.87506453	4.98040521	7.8063822	6.52952856	5.60836134
3.69501649	4.71048817	3.92033174	5.51328807	5.62395277	7.24429944	6.52944258
502.898804	354.51353	332.841958	349.632644	220.215524	301.478765	329.235373
89.3158444	57.9280498	56.2364829	57.8177905	49.2305716	46.8657878	40.7936648
19.6283041	7.86541978	8.51658275	6.84549522	4.34387397	5.62157637	5.15805495
25.2532018	32.6447785	36.6927601	36.5229715	49.0417075	39.408989	45.4400079
130.337588	73.9218004	66.4138958	74.1117088	61.7795409	46.7112428	47.220765
11.8852441	10.0563445	7.57029578	6.78400874	6.14857523	6.31702911	5.42414509
0.28242164	1.42410107	0.59867135	0.47139638	0.39871307	1.85454066	2.19012651
78.25433	59.9437006	56.0819871	55.2558537	22.915509	42.5192082	39.4018087
34.573116	23.5743501	20.0072103	19.265765	8.89759692	19.1249505	16.3747776
32.3843483	27.5180146	23.8116701	22.2171162	14.248746	18.8544967	14.0004349
6.80165455	6.87950365	4.48037913	4.14008993	1.69977677	4.3079434	3.295424
39.374284	23.6181686	23.3481826	20.2905397	6.40039401	11.5329247	15.0238585
12.732509	18.1189475	20.0265222	17.4621615	18.802469	19.5885857	18.9333366
9.34344933	7.20814236	7.49304786	9.20247711	4.32288907	6.81930054	7.02068591
85.9973901	60.2723393	57.7235053	53.3292772	28.4555222	26.4078862	25.7084009
10.2848548	4.68857892	3.53409216	4.07860344	3.73531191	4.17271648	3.8071358
485.318057	258.989212	266.428063	262.055395	79.1340518	130.764434	120.723048
944.535647	489.605949	520.264715	506.013268	212.870809	269.429985	255.58981
0.75312438	2.03756	1.68014217	2.35698189	0.10492449	1.56476868	1.49419846
71.0761133	49.6682636	50.2304574	52.4274754	27.2174132	38.8873994	39.9135205
346.154793	205.070555	227.707545	219.752694	105.784873	148.846206	163.03138
3.83622731	6.96714064	7.97584733	7.52184655	5.68690746	9.77497471	8.00317257
257.050765	153.320913	157.566437	150.867336	96.8872759	103.564505	106.395118
107.579111	44.2347703	44.9389752	39.9867101	27.5741565	29.1896972	28.2055545
60.5088369	12.7730912	16.0868785	13.2195941	4.95243602	12.9431483	10.0500198
11.4851468	3.81220903	3.24441248	4.85743224	1.3430335	2.10567637	2.39481123
1.45917848	0.32863871	0.44417552	0.59436935	0.81841104	0.65681648	0.34796402
584.801081	357.339823	399.371726	345.062148	254.294999	172.974552	188.985402
18.7810392	7.40532559	5.71634579	8.95653118	2.6650821	4.07612582	2.51762206
1.57685417	3.2425686	2.41399738	2.25450441	2.24538413	2.1249945	3.39776636
0.65898383	7.29577935	0	6.31261236	11.5416941	0	1.26904527

0.25888651	1.18309935	0.81110312	0.63536034	0.98629022	1.46817802	1.16670291
130.878896	183.533765	176.839792	175.830849	330.113437	197.315399	218.480471
51.1653875	71.6870571	65.6800406	70.7094566	123.768931	70.62709	82.4879423
7.01347078	11.2394439	12.3982905	12.0718464	8.79267243	19.3760862	21.2258055
39.044792	53.6119281	55.1743241	60.6256733	41.2772951	72.5589032	70.7185709
3.03603265	1.33646408	1.15871874	0.81981979	0.27280368	1.10113351	0.5117118
1.81220554	5.03912688	3.84308382	4.42702685	9.96782674	9.63974779	8.45347895
29.1600346	12.4444525	16.8400457	15.33063	12.9057125	11.571561	9.88627199
2.54179478	2.56338193	1.79601405	2.07004496	1.63682207	1.17840604	1.65794623
19.3223474	15.7308396	15.1985275	16.0479723	6.84107688	11.3011071	11.360002
100.848061	67.4366632	60.1761266	59.1704931	64.1508344	39.2158076	39.156187
1.36503794	1.27073634	1.08147083	1.31171166	3.12674986	4.40453406	3.09073928
3.38905971	2.14710623	1.33252655	2.11103595	1.93061065	1.29431483	1.35091915
9.72001152	12.7730912	15.5461431	15.4740985	17.0817073	18.9510873	17.4800751
26.6888452	43.2488542	38.4308382	38.2650886	64.5285626	44.1805675	45.1125124
21.1816232	13.4522778	12.2631067	13.1581076	19.9146686	13.9283731	12.792795
2.73007588	0.67918667	1.12009478	1.06576572	0.65053185	1.46817802	0.73686499
12.8266496	12.3568155	9.34699785	11.2520266	6.23251482	5.2158956	3.90947816
2.94189211	13.3427316	10.8919562	9.16148612	4.61667765	8.82838625	9.82486658
703.865338	447.0801	455.202657	433.459217	175.895418	267.015218	248.589593
30.9957752	49.8216283	42.331858	44.5981964	55.1063432	47.3873774	51.0279008
1.24736225	2.97965763	2.06638176	2.2954954	4.25993437	3.28408241	3.60245108
180.938132	118.222298	131.109026	126.764635	79.51178	97.6531564	104.982793
30.9251698	20.463237	19.5823467	20.7004496	15.4239003	13.3874654	14.3688674
35.255635	52.9327415	48.782059	52.038061	131.302509	128.600804	134.109429
7.43710325	9.00470064	8.69039056	9.18198162	12.9686672	12.8079214	12.6290472
49.4237874	32.7543247	33.7573393	34.2274761	32.2747737	22.2931241	24.4393556
102.095424	59.2206954	57.4145136	63.7819794	63.8360609	55.9453098	63.1657047
16.168639	22.8075264	20.238954	18.4869362	28.0987789	25.055617	24.4393556
327.915062	230.81392	217.781187	224.384676	226.07031	188.873375	171.218769
14.61532	11.4585363	8.7483265	7.02995468	5.33016419	7.59202581	6.95928049
256.933089	192.889013	186.321974	180.175894	181.351492	131.247388	141.887448
1.22382712	0.7887329	0.79179114	1.10675671	0.86038083	0.65681648	0.85967583
1.48271362	0.48200344	0.4634875	0.43040539	0.33575837	0.01931813	0
3.29491916	5.89358752	5.54253798	5.84121598	4.42781356	8.03634285	5.97679383
376.256233	216.748183	204.552482	212.681748	179.966489	175.389319	184.032032
23.3703909	16.5414817	15.5847671	14.4083328	6.65221279	12.2670137	10.6026685
10.1201088	15.2050176	14.5226082	13.9574319	27.7630206	20.1488115	20.5298775
0.61191356	1.68701204	1.79601405	1.96756749	1.84667106	1.60340494	3.09073928
118.475879	176.172257	153.549545	167.448192	237.045412	196.36881	192.874412
41.9160787	118.068934	107.355291	109.691888	106.813133	132.638293	119.842904
1.15322171	0.24100172	0.34761562	0.47139638	0.54560736	0.27045385	0.3684325
3.48320025	6.20031698	7.01024838	5.18536015	20.7120947	18.1204077	16.6613362

1.05908116	3.17684086	5.11767444	4.26306289	6.84107688	8.1329335	11.9126507
2.00048663	2.05946925	2.35606144	2.02905397	3.04281027	2.26022143	2.53809053
0.25888651	0.39436645	1.08147083	1.06576572	1.19613921	0.71477088	1.14623443
0	0.15336473	0.21243177	0.7788288	2.20341433	0.69545275	1.43279304
3.1301732	2.4100172	1.6608302	2.07004496	1.21712411	0.83067967	1.82169401
74.2298216	100.782538	90.1483181	92.475672	241.515196	218.700571	207.059063
1.45917848	1.90610451	3.30234841	3.4842341	4.44879846	2.66590219	2.02637873
2.25937314	1.13928086	1.1973427	0.55337836	0.79742614	1.56476868	1.35091915
13.5091686	20.7699664	26.3415394	22.2990982	29.0430994	29.2862879	25.2580945
53.3776904	84.9202425	85.3589473	78.8666635	120.72612	99.8167872	90.2659617
0.75312438	0.67918667	1.00422291	1.72162155	1.90962575	2.82044725	1.35091915
18.0043797	14.8325604	13.4990733	14.6952697	4.11304008	8.38406922	13.7752817
3.50673539	1.29264559	1.52564634	0.7788288	0.77644124	1.08181538	0.65499111
5.69550312	8.54460644	13.2866416	10.1452699	5.62395277	6.58748296	9.41549714
22.35838	16.1690245	13.0742098	15.1256751	11.2688904	10.6829269	12.833732
48.2705657	84.2629651	89.8779504	101.104275	127.168484	96.0883878	88.9969164
9.81415207	17.0015759	15.5268311	15.7405399	104.022141	60.4464345	66.3587863
2.07109204	4.4475772	4.38381924	4.69346828	4.07107029	5.48634944	5.1989919
40.0332678	26.3349152	26.2256675	30.66126	11.5836639	25.5772066	33.5068887
8.943352	5.60876731	6.21845724	6.9479727	3.52546293	4.09544395	4.50306385
27.7714615	17.3083054	14.0977447	14.3673418	10.7022982	9.25338515	8.96519075
0.30595678	1.42410107	1.33252655	1.41418913	0.50363756	1.56476868	2.7837122
76.7480813	67.2394799	61.9335167	63.7614839	25.4546817	36.1635428	44.3756474
11.9558495	23.0266189	24.1785977	31.1736474	51.9376235	43.0601159	41.0597549
8.14315735	16.6510279	13.1128338	16.0479723	17.1866318	17.5795	16.6203993
118.522949	78.5227423	71.0294588	79.5225194	66.9628108	56.5055356	57.8643704
1.15322171	2.08137849	2.20156561	1.98806298	2.5811425	2.43408461	2.49715359
21.5817205	11.3270809	11.4906275	13.4450445	12.6748786	11.2045165	9.23128089
109.038289	53.1956524	54.7880845	56.1166644	52.672095	39.408989	36.7204388
40.1509435	31.8341363	32.0578852	31.4605843	18.9493632	26.5044769	22.3720399
15.297839	12.050086	10.0036051	11.8463959	4.93145112	11.0306533	13.181696
15.815612	11.2613531	13.8660009	12.6457202	5.39311889	9.73633845	8.33066812
3.55380567	1.70892129	2.2981255	2.00855848	1.69977677	2.04772197	1.61700929
5.57782743	6.20031698	9.63667753	8.62860326	10.2406304	10.2192917	10.6436055
118.287598	250.9047	236.784175	230.533324	436.318007	363.799059	394.980105
17.6984229	8.61033419	8.90282233	8.8745492	5.05736051	6.47157417	4.70774857
64.1567831	42.5477582	46.7349892	44.7006739	36.6186477	36.9362681	31.3781676
58.131788	41.4084774	44.0313122	44.413737	24.4264217	29.7692412	29.1266357
0	0.08763699	0.63729531	0.63536034	1.17515431	1.00454286	1.43279304
2.63593533	4.84194365	5.46529006	4.75495477	10.3875247	4.44317032	4.74868551
0.35302705	0.92018839	0.7338552	0.63536034	0.86038083	1.3329511	1.08482902
3.38905971	6.9014129	9.15387805	7.29639611	4.82652663	6.20112032	7.69614549
7.46063838	15.9937505	13.9046249	15.3511255	23.7129352	12.30565	13.0179482

5.5542923	9.96870752	9.71392545	9.48941404	8.24706507	9.00224944	10.623137
16.9217634	23.0047097	21.6680405	23.4468459	32.6944717	35.9124071	33.8753212
4.40107059	1.90610451	3.36028435	2.99234222	0.52462246	2.70453846	1.96497332
100.871597	80.5603023	85.4555072	83.7855823	37.9616812	65.7589208	65.4991105
18.4515473	12.0281768	10.4284687	12.9326571	5.51902828	9.67838405	9.33362325
37.6091487	26.1596413	30.416367	26.6031521	16.4731452	21.9260796	24.2346709
21.158088	25.3489991	31.8647654	21.7047289	46.670414	36.1249065	38.3579166
1.15322171	2.36619871	0.57935937	1.57815309	2.6650821	2.14431263	2.21059498
101.295229	66.8451135	65.2938011	66.7333307	52.8399741	51.2316856	55.4900277
225.466611	133.098677	143.854932	141.767337	84.7999744	99.7974691	81.0551493
0.30595678	0.39436645	0.77247916	1.00427924	1.95159555	1.15908791	1.67841471
30.8074941	48.7480752	51.3312402	51.5461691	51.8117141	63.9816527	51.4986756
203.649539	289.881251	300.629577	266.707872	568.082184	349.638868	383.149328
12.5206928	20.0688705	20.6831295	23.6313054	13.430335	25.5772066	22.6176616
11.2497954	17.9217643	16.8593577	17.0317561	34.7090219	28.5521989	23.3340581
11.7675684	19.1925006	20.7217535	19.6756749	35.4434934	37.0908131	40.3638269
32.0548564	48.5947105	48.8979309	49.5171151	89.227788	77.0020735	70.6162285
4.98944901	1.79655828	2.54918123	2.25450441	2.35030862	2.74317472	1.84216248
0.11767568	1.05164387	0.71454322	1.12725221	2.81197638	2.31817582	1.22810832
5.62489771	4.38184946	3.1285406	3.05382871	1.86765596	3.11021923	2.23106345
116.216506	83.4085044	84.3933483	84.6668885	84.9678536	62.0691576	63.6364796
1.67099472	3.68075355	3.9589557	2.60292782	3.18970456	4.77157856	3.82760427
20.2166826	33.4992391	28.9872805	31.2761249	39.451609	51.3862307	46.8932694
38.5034839	25.9843673	30.2425591	28.1403142	27.9099149	19.8397214	19.8953548
7.13114647	6.30986322	6.17983329	4.91891872	1.97258045	3.41930934	2.61996442
5.62489771	4.20657548	3.4954682	3.52522508	3.54644783	3.30340054	4.50306385
2.00048663	2.23474322	2.62642915	2.58243233	4.55372295	3.53521813	4.74868551
17.2512553	9.94679827	12.2437947	11.2520266	8.98153651	5.50566757	7.04115438
136.150767	96.0720494	95.6715441	92.8035999	67.4034936	76.2873026	79.9498518
33.4904997	22.2597953	19.8527144	22.5860351	23.9437691	13.8317824	13.5910654
12.7560442	34.550883	33.8152753	34.6168905	34.1214448	37.4192214	40.281953
121.723728	63.7340004	71.4350104	69.8076549	74.3914648	56.6021263	54.9578474
4.35400032	2.69483742	3.20578852	3.0948197	1.86765596	1.60340494	3.41823483
14.1916875	8.85133591	8.13034317	8.03423391	9.02350631	6.68407362	8.39207353
30.1485103	18.2723122	17.1490374	15.9045039	10.7652529	9.29202141	10.3979838
176.160499	126.32872	125.354056	123.075446	121.439607	97.5565658	104.75764
15.2743038	8.25978623	9.46286972	7.11193665	6.5472883	6.25907472	4.99430718
10.5672764	6.24413548	7.80203952	7.35788259	4.84751153	4.75226043	5.99726231
16.3804553	8.8951544	8.03378327	8.52612579	6.4633487	6.12384779	4.62587468
2.75361101	8.8951544	8.47795879	9.59189151	9.52714387	14.6045077	11.6260921
5.88378421	29.5336654	28.9486565	25.6193684	122.593776	126.842854	120.70258
0.42363246	0.76682366	2.81954894	0.7583333	2.87493108	2.72385659	3.25448705
0.35302705	1.48982882	1.40977447	0.96328825	2.6021274	2.26022143	2.88605456

3.48320025	29.2269359	26.2642915	14.3673418	0.96530533	22.8147137	23.415932
4.07157868	3.39593333	1.44839843	2.35698189	2.81197638	1.50681428	2.59949595
6.98993565	1.53364731	1.42908645	1.66013507	1.67879187	0.4249989	0.59358569
3.1301732	3.76839053	4.53831507	3.32027014	6.16956013	4.3079434	4.76915398
3.88329758	7.84351053	5.11767444	9.07950414	7.47062383	7.66929834	8.28973117
56.3195825	34.8795217	37.0017518	36.5229715	19.2221669	33.8067307	36.249664
6.84872482	3.11111312	3.8623958	3.13581069	4.84751153	4.13408021	3.41823483
129.607999	71.1612352	81.1103119	73.8452673	55.4001318	48.9135098	52.6653785
301.602779	201.784168	203.026835	209.402469	179.001183	199.981301	171.628138
76.9834327	56.438221	54.6142767	53.7596825	54.0990681	39.7373972	33.7320419
11.9087792	16.1471153	18.1532603	15.535585	24.9510442	26.0022055	23.5387428
155.543719	108.932778	110.580392	111.208554	98.5660677	76.345257	80.2159419
13.1796766	21.5586993	19.9299623	17.4621615	15.6127644	22.4863055	25.0534098
0.61191356	2.60720043	1.06215885	1.22972968	1.59485228	1.23636044	1.65794623
15.9332877	8.87324515	9.07663014	6.49707181	6.35842421	7.39884449	8.28973117
43.3752572	33.1048727	35.2443617	30.3333321	7.15585035	13.8511005	15.2490117
15.2036984	6.46322795	7.76341557	7.80878347	5.93872624	8.07497911	8.2692627
0.49423787	0.92018839	0.67591927	0.79932429	1.09121472	0.9079522	1.37138763
1.69452985	2.51956344	2.16294165	2.60292782	2.81197638	2.37613022	2.72230678
0.21181623	0.98591613	0.48279948	1.00427924	0.46166776	1.13976978	1.67841471
102.142494	82.7074085	81.882791	76.0792763	37.4370587	60.9680241	64.7213086
213.557832	162.654252	151.811467	168.390984	105.616994	111.89062	106.865892
387.105931	243.083099	230.507782	235.903144	203.112832	238.385747	250.943467
101.718861	157.089303	154.746888	152.527471	212.954749	238.173247	257.698063
64.2509236	40.3349243	47.0439809	44.5162144	22.1600527	28.4556082	27.1411939
22.1465638	14.087646	12.3210426	11.7029275	8.41494425	7.10907252	5.32180273
2.4241191	1.59937505	1.54495832	1.88558551	0.69250165	0.8499978	0.75733347
1.01201088	0.67918667	0.4634875	0.61486484	0.18886409	0.3670445	0.85967583
8.26083304	6.28795397	5.07905048	5.16486466	5.85478665	5.23521373	3.99135205
43.9401005	29.2926636	33.3710997	31.3171159	20.3133816	23.8192566	25.6060585
151.542746	77.4491892	78.0590192	84.5849065	40.4169143	57.3941697	62.2036865
29.9602292	10.4945295	12.1279228	12.4407653	7.70145771	5.96930274	4.70774857
3.41259484	4.79812516	5.65840985	5.53378356	6.44236381	6.22043845	9.88627199
0	0.3724572	0.28967969	0.40990989	2.91690088	1.02386099	0.30702708
24.9472451	42.3067565	33.8345872	33.8175662	57.8973347	41.0896664	48.2032516
1.95341636	2.8701114	2.97404477	3.40225212	6.65221279	2.62726593	3.15214469
2.09462718	1.88419527	0.8304151	0.96328825	0.83939594	0.59886209	0.5117118
9.93182775	7.44914408	4.51900309	7.05045017	3.98713069	3.11021923	3.72526191
8.87274659	5.25821935	3.76583591	4.32454938	4.70061724	4.28862527	4.03228899
6.56630318	4.71048817	3.16716456	3.66869355	6.88304667	3.34203681	2.96792845
14.6859254	20.7480572	19.6402827	21.8481973	31.4563627	25.6544791	26.6294821
7.86073571	14.26292	10.2353489	11.8054049	10.1986606	15.0101884	14.6349575
15.5331903	14.525831	9.32768587	10.8831077	5.95971114	5.38975878	7.88036173

3.5773408	1.16119011	1.21665468	2.11103595	1.40598819	1.52613242	1.22810832
2.28290827	1.9937415	1.12009478	0.96328825	1.07022982	1.25567857	1.43279304
31.6547591	49.2081694	45.5183345	47.6520251	36.9334212	58.9782565	58.4988931
7.67245461	4.4475772	5.40735413	5.06238719	4.67963234	4.28862527	2.57902748
1.20029198	2.01565075	1.62220624	2.23400892	2.70705189	1.93181318	1.30998221
9.60233584	7.20814236	6.93300047	6.43558533	4.93145112	4.21135274	7.36864993
6.42509236	5.25821935	5.58116194	4.38603586	4.02910049	3.32271868	4.27791066
26.6653101	15.3802916	16.0096306	14.6747742	10.0307814	13.8124643	11.012038
8.56678982	3.70266279	6.21845724	3.89414399	3.06379517	4.34657966	4.9738387
26.5947046	18.6228602	20.1230821	18.5074317	10.5554039	16.2465489	19.6906701
1.10615143	1.70892129	1.29390259	1.5371621	3.46250823	2.80112912	2.76324372
0.65898383	1.00782538	0.75316718	0.96328825	1.53189758	1.79658626	2.10825262
4.49521114	13.3208224	13.1128338	15.494594	12.3181354	16.555639	16.2929037
0.32949192	0.87636989	0.96559895	1.12725221	1.17515431	1.35226923	1.92403637
11.1556549	16.8920297	17.1104134	16.3554048	19.0962575	16.845411	20.2637873
185.315668	72.4976993	83.775365	71.9596818	54.728615	42.9442071	43.1475391
14.9448119	37.7277238	32.7338044	36.3385121	37.2272097	44.6248845	43.0042597
0.70605411	1.05164387	1.58358228	0.88130627	2.01455025	0.71477088	2.39481123
6.87225996	8.12833075	8.07240723	9.38693656	11.8564676	13.773828	12.5676418
2.61240019	6.63850193	9.71392545	3.32027014	2.95887067	5.35112252	6.14054161
1.9769515	3.2425686	3.05129268	3.34076563	3.31561395	4.63635164	2.88605456
4.80116792	8.8951544	8.47795879	9.59189151	22.4118715	12.9238302	12.6495157
22.9938287	17.3740331	18.2111962	15.6175669	12.5489692	9.07952196	10.3775153
3.08310293	1.57746581	2.51055727	1.96756749	2.49720291	1.37158736	0.8801443
15.6273309	9.66197806	10.9112681	12.7072067	8.24706507	9.54315713	8.84237992
26.147537	18.7324064	20.1423941	18.0565308	14.5215497	12.2476956	13.3045068
20.7109204	15.3364731	12.861778	15.453603	10.0727512	12.4795132	11.6260921
17.6042824	10.6478942	11.4520036	11.5389635	3.54644783	11.2238346	11.8307768
64.0155722	41.6713884	42.9498413	42.9175659	37.9406963	38.6748999	35.9631054
6.42509236	13.7151888	12.9776499	13.3630625	9.94684184	13.3874654	14.614489
0.70605411	1.22691785	0.54073541	1.39369364	1.2800788	2.20226703	1.59654082
1.55331903	3.72457204	1.6608302	1.84459452	3.16871966	2.64658406	1.49419846
43.3517221	27.3427406	26.650531	30.866215	13.2624558	21.5783533	23.2726527
108.332235	71.9718774	73.8683197	73.2713935	59.9328698	58.7464389	58.2942084
22.4525206	15.7308396	13.0355858	12.7277022	6.5263034	12.1124687	11.5646867
78.1366544	45.2645049	39.5123091	42.466665	19.6628498	25.345389	26.2815181
17.6513526	43.2050357	40.2075403	47.0576558	59.9118849	59.3066647	61.6305693
17.6748878	9.81534279	14.1749926	14.1009003	10.6183586	10.0261104	9.35409172
3.08310293	3.70266279	5.92877756	5.20585565	6.79910708	4.01817142	5.30133426
4.33046518	1.97183226	1.40977447	2.04954947	0.86038083	0.92727033	1.43279304
45.5169547	86.8482563	73.2310244	77.4114834	81.6732245	78.8566142	86.9296007
12.1676658	12.9483652	17.5739009	14.1009003	28.287643	22.1772154	20.2433188
4.73056251	2.54147269	3.88170778	3.79166652	2.6650821	1.98976758	2.08778415

25.1590613	15.8184765	14.6577921	15.7815309	11.35283	12.6147401	12.4243625
13.9563362	21.9968843	18.423628	17.9950443	26.5878663	28.571517	24.889662
5.95438962	4.57903268	2.70367706	3.4637386	4.07107029	3.38067307	2.76324372
0.91787034	2.08137849	2.12431769	2.2954954	2.85394618	2.78181098	1.78075707
16.0980336	25.6557286	23.1550628	23.1804045	47.8245834	26.9294758	31.1734829
22.9702936	68.3568516	65.0041214	63.2695921	117.179673	75.3020779	90.0408085
84.6558873	49.0986232	55.8695553	49.3326557	31.8340909	37.4385395	40.8346017
11.1085846	7.95305677	8.76763847	5.63626104	3.04281027	5.54430384	5.15805495
158.673893	108.604139	118.247248	106.556077	67.3615238	60.1180263	53.4841174
31.4194077	18.0532198	21.5907925	19.5117109	16.6200395	15.7442774	18.9128682
33.1610078	60.1847023	65.7186646	61.0560787	114.409666	83.9372828	93.6023226
3.29491916	1.94992301	2.00844582	1.51666661	0.98629022	0.38636264	0.65499111
3.93036785	1.51173806	3.01266873	2.2954954	1.76273146	1.48749615	1.47372999
3.38905971	1.2488271	1.71876613	1.98806298	1.04924492	1.08181538	0.90061277
1.03554602	1.27073634	1.37115051	1.35270265	2.37129352	2.04772197	2.29246887
1.69452985	0.74491441	1.23596666	0.73783781	0.73447144	1.25567857	0.8801443
30.5486076	19.3458654	20.258266	19.142792	15.4239003	14.8170071	16.1086875
56.3195825	37.6619961	41.8297466	41.2984218	16.8508734	34.7919554	29.6383475
785.132165	370.332007	389.213625	387.446831	108.156166	156.863231	154.72118
4.30693004	2.60720043	3.24441248	3.05382871	2.24538413	3.24544615	2.66090136
0.4471676	1.33646408	0.92697499	0.7788288	1.86765596	1.10113351	1.16670291
3.81269217	5.60876731	5.07905048	4.30405388	16.0534473	15.8795044	13.6524708
3.01249752	8.43506021	9.50149368	8.8540537	14.3746554	18.023817	16.9478948
7.08407619	5.2801286	4.6928109	5.14436916	1.99356535	4.07612582	3.70479344
6.21327613	3.98748301	5.40735413	3.4637386	4.61667765	5.83407582	3.15214469
3.67148135	3.11111312	2.45262134	2.50045035	3.48349313	1.71931373	2.16965804
175.407375	110.334969	118.594863	120.226572	83.6038352	97.884974	87.1752224
27.7949966	21.0547866	19.8140905	19.6961704	17.4804204	16.6135934	14.9419846
2.37704882	0.52582194	1.15871874	0.69684682	0.46166776	1.10113351	0.24562166
36.7148135	16.8263019	22.8653832	21.7457199	7.74342751	12.30565	13.0384167
0.21181623	0.30672946	0.25105573	0.02049549	2.26636903	1.71931373	2.76324372
128.501847	81.7434017	83.0221978	82.3918886	56.2185428	67.0532356	65.9698854
499.956912	344.522914	369.592654	343.012599	328.28775	281.600408	269.52884
54.2955607	32.1408658	31.3433419	34.9653139	19.4739857	28.3010631	28.2055545
2510.89315	3816.91952	3673.17703	3762.89084	7443.76316	5153.65258	5018.767
0.58837842	1.86228602	1.13940676	1.31171166	4.23894948	1.50681428	0.94154971
38.4564136	54.6854812	53.1465663	45.1925658	91.3472627	58.2441675	60.5252718
23.7234179	16.6729372	15.5268311	20.3520262	10.0937361	13.6386011	17.0911741
10.496671	6.17840774	5.40735413	4.16058542	5.66592257	3.11021923	2.43574817
1.60038931	0.3724572	0.52142343	0.61486484	0.29378858	0.54090769	0.38890097
3.1301732	0.70109591	1.1007828	1.20923419	1.17515431	0.38636264	0.40936944
78.1131192	37.6400869	37.0596877	34.8833319	17.8791334	21.9260796	21.1644001
48.2470306	29.7527578	29.9721914	27.8123863	22.5377809	20.5931285	22.3925084

5.22480038	4.33803096	2.78092498	3.79166652	2.53917271	3.18749175	2.4152797
31.1605212	100.278625	103.666703	93.7668882	288.01773	220.36193	238.785195
8.91981687	12.5539987	13.0935218	10.6986482	36.555693	30.1942401	27.3049417
1.3415028	2.49765419	2.62642915	2.54144134	3.71432702	2.14431263	2.37434276
2.98896238	3.52738881	4.90524267	3.19729717	6.96698627	5.48634944	4.1755683
2.44765423	5.80595053	5.48460204	5.26734213	4.88948133	5.56362197	6.46803716
3.67148135	4.90767139	6.68194474	5.98468445	4.49076826	7.86247966	7.53239771
2.56532992	5.58685806	7.02956036	6.10765741	9.65305326	4.81021483	5.05571259
5.20126524	7.66823655	7.20336817	7.58333303	9.33827978	8.88634065	8.10551493
8.30790331	5.36776559	5.25285829	5.0213962	4.88948133	3.70908131	3.17261317
2.00048663	5.45540258	5.67772183	6.08716192	6.33743931	5.85339395	5.95632536
0.56484328	0.92018839	1.06215885	2.25450441	2.20341433	1.71931373	2.51762206
0.11767568	0.28482021	0.67591927	0.65585583	1.59485228	0.88863406	1.67841471
10.3789954	4.46948645	7.28061609	7.02995468	5.98069604	6.81930054	5.3422712
3.88329758	1.81846753	2.91610883	1.57815309	0.92333553	1.91249505	1.00295513
71.4997458	105.602572	94.9763128	95.9189151	109.45723	113.43607	110.734434
2.11816232	2.30047097	3.2251005	2.97184673	4.23894948	3.01362857	2.0468472
9.72001152	7.60250881	6.68194474	6.12815291	2.85394618	4.40453406	3.70479344
18.1926608	12.9264559	12.842466	14.4288283	6.44236381	7.88179779	7.59380313
44.5284789	55.8685806	60.8906698	59.1909886	74.5173742	81.5804708	81.648735
98.2591964	131.039208	129.988931	138.693013	131.344479	182.20862	171.136895
10.7555575	44.4538628	37.2141836	35.1907644	98.6500073	61.6055224	62.1013442
1.74160013	3.72457204	2.91610883	3.97612597	5.12031521	4.21135274	3.23401858
12.0735252	17.3083054	16.569678	18.0565308	25.0139989	28.4942445	30.4570864
131.420204	101.483633	97.9696696	101.555176	71.5585035	71.5350422	65.5605159
0.4471676	1.90610451	0.54073541	0.63536034	1.07022982	1.89317692	1.65794623
0.8943352	3.35211484	1.91188592	2.27499991	3.16871966	3.30340054	2.49715359
35.3733107	26.1158228	27.1140185	25.3324314	21.9292188	19.0669961	22.0445444
26.4299587	16.0594783	19.0802353	16.4373867	17.4594355	10.6442906	13.8571556
8.4961844	13.2550946	14.3294884	12.0513509	12.8637427	14.4499626	13.8162186
36.4323919	14.5915587	13.7308171	14.8182427	4.13402498	4.67498791	4.64634315
31.1605212	47.4992481	47.0246689	44.3932415	68.5996328	54.0328148	50.90509
21.675861	18.1408568	17.458029	17.3391885	9.38024958	6.95452746	7.79848785
1.27089739	0.46009419	0.63729531	0.57387385	0.10492449	0.69545275	0.26609014
47.0702737	35.9092563	42.6601617	37.5067553	13.9759423	22.9885769	22.8837517
41.7513328	31.0673127	26.1484196	30.2718456	23.9437691	22.9885769	19.3222376
91.7634986	49.4929896	52.489959	57.674322	23.2092976	37.8635384	35.9017
19.1576014	26.3568245	23.1550628	24.2461702	47.5517798	30.039695	31.9512848
0.11767568	0.50391269	1.48702238	0.22545044	1.88864086	1.35226923	1.12576596
36.8324892	48.8795307	48.9751788	52.7349078	66.2283393	60.5430252	55.8789287
0.37656219	0.56964043	0.90766301	0.32792791	0.23083388	1.19772417	1.37138763
4.2127895	2.71674666	4.17138747	2.19301793	1.57386738	3.28408241	3.31589247
14.6623903	10.7355312	9.79117336	10.0632879	8.98153651	8.21020603	8.88331686

1288.384	896.066305	921.915254	916.701991	863.927282	689.019808	704.852303
73.5943729	40.137741	41.9263064	42.1182416	28.9801447	19.9942665	24.0095177
2.89482183	1.97183226	2.04706978	1.76261254	1.78371636	1.66135934	2.02637873
0.28242164	0.13145548	0.13518385	0.51238737	0.16787919	0.46363516	0.16374778
2.4241191	3.41784258	3.07060466	2.82837827	6.5263034	3.8249901	6.20194703
101.883607	142.169106	146.828976	145.784454	220.425373	176.277953	168.967237
42.6692031	54.3568425	63.3046672	54.6819798	80.749889	71.0134526	70.8823187
0.30595678	1.18309935	0.81110312	1.37319814	0.62954695	1.35226923	1.00295513
2.89482183	2.03756	2.10500571	1.88558551	1.97258045	1.12045165	1.59654082
4.49521114	1.88419527	2.89679685	1.35270265	0.73447144	1.95113132	0.53218027
131.067177	82.0501311	80.5309525	76.3252222	43.774498	41.5726197	41.0597549
3.20077861	1.70892129	3.08991664	2.58243233	2.45523311	1.98976758	1.73982012
47.2114845	37.8810886	34.954682	36.8508994	21.7403547	26.6397038	27.8985274
0.77665952	1.16119011	1.64151822	0.86081078	1.23810901	1.60340494	1.69888318
1.22382712	0.43818495	0.0965599	0.40990989	0	0.3670445	0.5117118
9.46112502	5.49922107	6.70125672	5.96418895	4.21796458	5.35112252	4.8305594
2.11816232	4.77621591	4.15207549	4.18108091	6.31645442	4.61703351	3.8071358
0	0.85446064	0.54073541	0.63536034	2.24538413	1.91249505	1.59654082
386.305736	601.233564	624.626649	622.981056	617.753439	631.277912	630.67456
7.53124379	3.83411828	4.09413955	3.83265751	5.66592257	4.48180659	3.47964025
0.42363246	1.09546236	0.56004739	0.98378374	0.71348655	1.12045165	1.08482902
76.1126326	97.101784	102.392113	100.48941	163.724177	108.954264	105.740127
30.948705	20.6823294	19.6789066	19.224774	11.8144978	13.9476912	13.7343447
212.428145	300.419599	316.27228	294.438277	557.337916	332.04005	344.668601
11.9793847	15.5993841	15.8937587	18.6304047	27.0915038	17.9465445	18.8309943
3.22431375	3.00156688	2.22087759	1.59864859	2.01455025	2.47272088	2.45621664
2.47118937	6.13458924	3.72721195	4.48851333	8.12115568	8.53861427	9.23128089
0.6354487	2.12519699	2.04706978	1.61914408	2.45523311	3.09090109	4.23697371
4.14218409	1.77464903	1.52564634	2.39797288	1.11219961	1.37158736	2.14918956
0.30595678	1.00782538	1.02353489	0.81981979	1.88864086	1.3329511	1.08482902
42.8574842	26.1815505	28.1761774	26.5416656	29.0221145	26.1760686	26.1382388
10.6378819	9.81534279	9.88773326	8.54662128	5.39311889	5.37044065	5.69023523
1.48271362	0.76682366	1.52564634	2.3159909	6.21152992	3.72839944	3.07027081
37.9857109	27.2770129	29.1610883	30.3948186	16.2003416	17.6567725	16.2314983
5.24833552	6.4851372	7.76341557	6.02567544	11.4787394	10.1613373	7.34818146
10.9203035	8.50078795	8.24621504	9.59189151	2.97985557	6.007939	8.16692034
1.10615143	0.65727742	1.17803072	0.53288286	0.54560736	0.23181758	0.28655861
4.00097327	0.8325514	1.12009478	0.81981979	1.42697309	1.08181538	0.32749555
2.54179478	1.77464903	1.21665468	1.57815309	1.07022982	0.57954396	1.04389207
0.40009733	0.92018839	1.23596666	0.96328825	2.26636903	1.87385879	1.7602886
28.0538831	14.438194	14.715728	17.0112606	5.66592257	6.18180219	7.18443368
14.0269416	8.56651569	7.93722338	7.80878347	6.40039401	6.29771098	4.72821704
14.4741092	10.7793497	9.79117336	11.1905401	3.54644783	3.8249901	3.82760427

12.0735252	16.4538447	15.5654551	19.1222965	29.4627973	25.055617	29.658816
5.31894093	1.70892129	1.75739009	1.5371621	0.08393959	0.09659066	1.08482902
3.22431375	4.55712344	5.48460204	5.24684664	5.87577155	5.37044065	5.69023523
9.81415207	14.3724662	12.4369145	12.1538283	18.7604992	29.595378	29.8430322
0.58837842	2.34428946	1.89257394	1.68063056	4.28091927	2.26022143	2.57902748
131.020107	91.3615612	85.3010113	88.8069784	34.4362183	55.3464477	57.9871813
27.3948993	18.7104972	20.1423941	19.5731974	9.1913855	13.329511	11.5442182
3.78915703	5.30203785	5.02111454	4.36554037	8.72971773	5.19657746	5.48555051
5.15419497	3.68075355	3.53409216	2.17252244	2.6650821	1.97044945	3.47964025
1.10615143	0.50391269	0.32830364	0.55337836	0.18886409	0.34772637	0.63452263
10.143644	18.1408568	17.2842212	17.8720714	23.5030862	24.0703923	23.456869
30.9016347	15.3145639	17.9601405	16.5808552	19.8936837	21.4624445	20.3251927
61.1442855	39.9405578	41.8104346	44.4752235	41.717978	30.6965115	35.0010872
0.82372979	0.8325514	1.79601405	1.41418913	4.21796458	2.62726593	2.84511761
5.90731935	2.93583914	4.65418694	5.26734213	4.51175315	3.43862747	3.27495553
28.4304453	10.1878	10.8340202	11.1495491	12.6748786	9.33065768	9.23128089
83.5026656	67.5681187	66.3752719	67.0202676	33.3659884	45.1078378	45.1125124
14.6623903	43.7527668	38.3342784	38.7774759	120.453317	72.3464037	78.9468966
0.28242164	0.48200344	0.79179114	0.18445945	0.44068287	1.71931373	1.59654082
4.28339491	8.54460644	10.7760843	10.2682428	14.7313987	14.0056456	15.4332279
0.35302705	0.81064215	0.86903906	0.96328825	1.84667106	1.29431483	1.80122554
9.22577365	25.8090933	27.3457623	24.2666657	54.3928566	35.1783181	32.2378435
0.25888651	1.29264559	1.69945415	1.37319814	5.47705848	3.47726373	2.96792845
18.569223	57.3365002	52.5865189	49.968016	109.058517	68.4055048	55.7561178
0.32949192	1.75273978	1.73807811	1.70112606	3.44152334	2.85908351	3.97088357
0.72958924	1.44601032	1.44839843	1.68063056	3.75629681	1.52613242	1.80122554
0.18828109	1.27073634	0.79179114	0.84031528	1.65780697	3.57385439	2.16965804
0.18828109	1.64319355	1.42908645	1.9265765	0.54560736	2.24090329	1.92403637
8.96688714	26.9702834	28.0216816	27.1565305	61.7375711	39.8146697	41.4486559
8.96688714	26.9702834	28.0216816	27.1565305	61.7375711	39.8146697	41.4486559
4.00097327	6.28795397	9.19250201	7.27590061	7.59653322	10.4897456	8.33066812
6.2603464	17.1987591	19.4471629	19.388738	28.4765071	25.1715258	22.7814094
0.91787034	2.34428946	2.0277578	2.23400892	2.07750494	3.97953516	3.74573038
0.96494061	1.9937415	2.35606144	2.13153145	2.6650821	4.50112472	3.99135205
0.96494061	1.9937415	2.35606144	2.13153145	2.6650821	4.50112472	3.99135205
3.71855162	6.98904989	7.18405619	7.13243215	13.0735917	10.1420192	11.4214074
3.71855162	6.98904989	7.18405619	7.13243215	13.0735917	10.1420192	11.4214074
5.74257339	7.62441806	6.77850464	7.50135105	15.906553	10.0067923	7.96223562
77.0540381	40.8169277	40.1109804	43.7783766	16.5780697	24.0897104	24.8487251
49.1178306	32.5790507	33.7187154	31.6040528	27.5951414	24.5919818	22.3720399
4.73056251	2.21283398	1.08147083	1.63963957	0.14689429	1.58408681	0.8801443
5.08358956	9.94679827	11.4713155	13.0556301	11.0380566	13.1556478	11.4214074
1.88281095	4.84194365	6.19914527	6.37409885	3.44152334	5.38975878	3.21355011

1.29443253	1.55555656	2.60711717	1.63963957	3.54644783	1.19772417	2.29246887
3.27138402	6.24413548	6.46951297	6.31261236	6.23251482	4.7329423	5.75164064
2.11816232	3.30829634	3.34097237	4.46801784	7.34471444	3.28408241	3.39776636
72.1822647	122.428874	116.007058	117.439185	125.300828	95.5088438	90.5115833
12.6854388	16.4538447	19.7175306	19.5936929	28.2037034	18.0431351	20.9392469
6.23681127	10.8669867	9.50149368	9.44842305	13.7031386	8.01702471	8.53535284
4.25985977	6.92332214	6.70125672	6.49707181	9.84191735	6.16248406	5.52648745
28.4304453	7.6901458	12.1279228	9.32545008	5.66592257	11.5908791	11.6056236
126.548431	78.4351053	73.1151526	71.4882854	73.2163105	49.1260093	51.4372702
12.7795793	10.012526	10.7374603	8.77207172	6.90403157	5.81475768	6.52944258
68.7696699	50.3474503	49.2455465	50.7263493	20.5861853	29.1124247	29.8225638
7.55477893	19.9155058	24.9703889	21.5612604	98.2722792	76.8861647	66.1950386
52.3186092	68.6416718	71.2418906	69.520718	133.254105	82.93274	87.4003756
2.09462718	0.98591613	1.00422291	1.04527023	0.37772817	0.23181758	0.06140542
11.2027251	8.58842494	8.11103119	7.23490962	5.68690746	3.28408241	4.29837913
22.3819151	13.4741871	16.2993103	16.7448192	12.1082864	10.6249725	10.1114252
12.3559468	6.4413187	7.8213515	7.54234204	9.1913855	8.2488423	8.02364104
0.40009733	0.32863871	0.88835103	0.73783781	1.3220486	1.15908791	0.85967583
3.55380567	1.27073634	1.79601405	1.98806298	1.23810901	1.12045165	1.24857679
32.7138402	53.8310206	45.1514069	45.0490973	93.0890093	57.8578049	58.580767
6.77811941	6.52895569	5.50391402	5.86171148	3.65137232	3.99885329	3.82760427
33.8670619	23.3114391	25.1055727	23.1394135	13.1155615	17.2704099	15.9244712
39.9626624	30.3662167	27.0947066	28.714188	20.8799739	18.2169983	25.2171575
0.51777301	0.92018839	0.79179114	0.67635132	2.18242943	1.17840604	1.67841471
9.08456283	18.162766	21.7066644	19.0813055	15.1930664	17.3863187	21.6761119
10.6143467	19.0829544	18.0760124	18.1795038	33.3869733	25.9442511	27.2025993
7.24882215	9.99061677	7.85997546	9.42792755	11.562679	9.98747416	11.9945246
1.12968657	0.52582194	0.63729531	1.1682432	0.27280368	0.71477088	0.47077486
89.1275633	166.948464	145.88269	166.13648	297.670784	165.962071	175.701364
159.332877	78.3255591	86.9425295	86.593465	81.7571641	82.4304686	80.6662483
2.32997855	1.2488271	1.27459062	1.12725221	2.22439923	1.60340494	1.69888318
30.8074941	19.8278688	19.292667	17.5646389	14.2907158	9.33065768	11.6465606
3.10663806	1.77464903	1.21665468	1.74211705	1.61583718	2.24090329	1.80122554
1.29443253	7.25196085	5.11767444	5.16486466	16.1373869	10.103383	9.35409172
42.7162734	67.4147539	69.059637	66.1389613	109.352306	75.4179867	77.0637972
16.5452012	11.3051716	11.6451233	11.2110356	15.8855681	7.55338955	8.18738881
78.5132165	122.341237	123.075242	110.778149	166.200395	135.033742	150.770765
9.88475748	15.4460193	14.1556806	15.3511255	30.6799214	16.5749571	16.0063451
2.47118937	0.39436645	1.1973427	0.49189187	0.35674327	0.96590659	0.40936944
2.63593533	0.74491441	0.7338552	0.49189187	0.86038083	0.61818022	0.53218027
70.2288484	46.9734262	47.9130199	45.1105838	41.3822196	40.8964851	35.6151413
48.1293549	39.1956434	35.2250497	33.3666653	22.6636903	25.210162	27.9804013
42.998695	32.7981432	33.17798	32.608332	17.3964808	23.587439	24.6031034

275.102215	209.189493	204.108306	211.513505	99.951071	127.403079	119.024165
387.600169	185.220777	199.009944	186.549993	96.866291	80.2282015	69.9612374
358.463669	181.802934	192.154191	196.756749	20.4602759	35.3328631	34.8987448
62.2739721	36.8951724	41.9263064	38.53153	37.6049379	26.234023	21.8398597
5.50722202	10.3411647	8.76763847	8.42364831	7.15585035	12.5181494	13.4068492
33.2551484	51.7496421	53.7452376	51.6691421	95.1245444	72.4623125	69.6746788
1.83574067	2.21283398	2.37537342	2.21351343	2.97985557	3.72839944	4.4621269
2.09462718	1.6212843	1.27459062	1.18873869	1.46894289	1.41022362	1.24857679
0.49423787	0.98591613	0.48279948	0.49189187	1.15416941	0.94658846	1.33045068
57.3786637	17.899855	18.0180764	18.9378371	6.35842421	8.59656867	7.12302827
9.03749255	7.16432387	5.34941819	6.5585583	5.83380175	4.34657966	4.27791066
20.7815258	14.3286477	11.8189312	12.8916662	9.63206836	10.702245	9.47690255
34.7849323	47.2363372	49.4000423	48.020944	70.005621	72.38504	68.9787508
3.3184543	1.92801376	1.77670207	2.00855848	2.14045964	1.89317692	1.7602886
0.32949192	1.22691785	0.52142343	0.69684682	1.74174657	0.79204341	0.85967583
13.7680551	84.964061	86.6142259	87.5362578	371.432702	289.733341	279.476517
0.72958924	1.79655828	1.6608302	1.49617111	1.40598819	1.81590439	1.45326151
80.6784491	49.3615342	52.3161512	53.0833312	62.4300727	45.3976098	43.9662779
27.4184344	19.937415	21.4942326	19.3272515	20.1455025	15.0681428	13.140759
3.67148135	9.22379311	11.8189312	10.5346843	9.0654761	9.48520273	9.16987547
2.21230286	5.47731182	3.99757966	3.97612597	2.72803679	4.67498791	4.89196482
385.576147	279.846816	279.173969	281.710574	184.520212	234.869847	260.829739
3.74208676	4.75430666	5.83221766	4.11959443	6.23251482	7.14770878	6.28382092
8.16669249	2.51956344	2.81954894	3.01283772	1.38500329	2.49203901	1.73982012
101.271694	76.5070916	72.670977	69.438736	29.7565859	41.9976186	41.4486559
53.8954634	37.6839053	42.9691533	41.2984218	21.8033094	32.2612802	26.4247974
18.6868987	13.0360021	11.6258114	9.50990953	12.192226	10.5670181	11.5237498
114.639652	77.5368262	80.6275124	74.0297268	45.5372295	59.055529	57.1889109
2.35351369	2.54147269	3.10922862	3.36126113	2.91690088	2.82044725	4.01182052
50.7652902	81.0203965	82.5393983	74.1322043	115.144137	87.7429548	87.9939613
10.9438386	7.12050537	8.6517666	6.5380628	2.03553514	1.79658626	2.25153192
1.48271362	1.90610451	1.9311979	2.35698189	3.54644783	2.91703791	2.47668512
370.490124	299.302227	300.243338	302.738952	72.2300203	156.592777	168.291777
10.8967684	4.55712344	3.18647654	3.23828816	2.39227842	3.09090109	2.06731568
57.0727069	38.6479122	41.5786908	39.1668903	16.4521603	31.1794648	34.9806187
375.879671	241.045539	253.604908	240.842558	205.295261	211.456271	202.392251
557.570927	368.031536	362.543782	362.626787	125.615602	219.280115	241.466565
2.63593533	0.13145548	0.28967969	0.22545044	0.25181878	0.11590879	0.18421625
1.88281095	3.30829634	2.43330936	1.96756749	4.05008539	2.22158516	2.70183831
201.17835	82.0063126	81.3806796	86.2860326	29.94545	59.1328016	59.5018482
1074.87324	609.975354	623.197563	587.154932	167.816232	207.264237	199.055891
343.259971	236.838963	244.257911	227.131072	158.855681	154.660964	154.536964
6.2603464	16.30048	16.7241738	14.0599094	10.6393435	9.54315713	9.27221783

1.76513526	0.7887329	1.5642703	1.29121616	0	0.81136154	0.92108124
17.4160013	22.325523	20.4513858	23.6108099	35.0237954	25.9635692	29.7816268
9.15516824	4.33803096	6.00602547	6.39459434	3.44152334	7.43748076	7.08209132
2.63593533	0.7887329	1.5642703	1.72162155	2.14045964	1.37158736	1.37138763
25.4885532	34.5289737	35.3022976	31.2761249	66.5640977	33.3624137	35.6356098
36.2205756	43.9499501	45.4797106	42.6716199	94.7048464	97.730429	86.2336727
51.8479065	37.8372701	39.7440528	41.3804038	21.4045964	33.9226395	33.1589247
159.94479	114.081451	119.985326	120.697968	65.8506112	67.3623257	70.0021744
1.12968657	1.38028258	1.81532603	2.27499991	4.72160214	4.19203461	3.02933386
4.80116792	9.11424687	5.04042652	7.84977446	7.65948791	11.5522428	11.5032813
2.11816232	0.3724572	0.28967969	1.02477473	0	0.69545275	0.3684325
13.1326064	8.45696946	8.63245462	9.03851315	5.54001318	4.13408021	5.50601898
0.37656219	1.42410107	0.81110312	0.43040539	1.46894289	1.13976978	1.14623443
5.03651929	3.11111312	2.45262134	2.52094585	1.67879187	2.02840384	1.08482902
1.50624876	3.2425686	2.24018957	2.97184673	2.97985557	3.65112692	4.35978454
4.51874628	2.8701114	2.58780519	2.72590079	2.68606699	1.79658626	2.35387428
10.5908116	5.30203785	5.36873017	4.95990971	3.23167435	4.13408021	4.48259538
13.8621956	7.55869032	7.58960775	5.49279257	5.95971114	5.94998461	6.18147855
30.2191157	22.4569785	25.0283248	22.1966207	12.8847276	14.6817802	15.8425974
6.35448695	3.30829634	4.90524267	4.46801784	2.39227842	3.32271868	2.98839692
47.9175386	37.5524499	32.4827487	34.0430167	19.3480763	22.4476692	26.6090136
1.90634609	3.33020559	7.49304786	6.31261236	7.30274464	13.5999648	11.9331192
12.5442279	18.6885879	18.423628	18.9788281	27.1964283	26.6397038	25.3399684
7.43710325	11.3489901	11.2395718	10.7191437	13.5142746	12.0545143	12.5267049
78.7721031	51.5305496	47.9516439	45.6434667	44.5299544	34.135139	34.0800059
2.61240019	3.11111312	3.93964372	4.16058542	3.39955354	6.87725493	4.35978454
1.15322171	2.89202064	3.01266873	2.84887376	6.58925809	6.16248406	6.30428939
17.6984229	11.4147178	12.842466	14.1623868	8.89759692	10.0454286	9.19034394
71.0055079	47.6307036	46.4259976	44.8646379	26.0842287	37.5930846	31.2348883
29.4424562	7.6901458	4.26794736	5.51328807	1.90962575	3.36135494	3.58198261
150.954368	95.5681367	96.4633352	97.6815276	51.5179256	73.0998109	74.3824274
74.5828487	62.5289918	59.847823	55.4813041	16.8089036	38.385128	33.8548527
38.5505542	27.2112851	22.3246477	24.9635125	9.75797775	16.9226835	15.6993181
34.5025106	26.9045557	26.4767232	28.1608097	17.8161787	19.4147225	16.3338407
100.495034	74.0970743	76.3402531	74.8290511	39.1368355	40.7033038	36.8841866
48.1293549	24.5602662	26.2256675	28.8986475	12.7798031	20.6704011	24.6235719
78.7014976	55.1236662	53.9383574	53.2882862	59.9538547	39.119217	39.1152501
104.142981	70.4163208	69.1561969	72.6155377	55.0224036	47.7157856	45.6446926
76.3009137	50.1721763	55.9468032	56.4855833	32.6105321	40.3362593	42.7381696
47.7292575	37.8591793	34.066331	34.2069806	27.1754434	23.2783489	20.9597154
252.178991	153.737188	167.74385	162.570264	78.0848069	123.191727	122.196778
1.76513526	5.52113032	7.14543224	4.34504487	11.0380566	10.5670181	12.3015517
8.19022763	4.77621591	5.65840985	5.36981961	3.04281027	3.18749175	4.38025302

21.3699043	15.4460193	16.7821098	15.9454949	9.75797775	13.329511	14.3893358
27.865602	37.0266279	42.5249778	41.0114849	41.2143404	48.3919203	47.8143507
141.163751	200.447703	195.282732	182.819813	253.203784	256.332291	247.1568
2.37704882	1.66510279	1.50633436	1.5371621	1.00727512	0.8499978	2.57902748
4.44814087	2.62910968	2.78092498	3.27927915	2.03553514	1.89317692	1.43279304
2.44765423	4.51330494	4.6928109	6.68153127	7.72244261	6.54884669	7.36864993
4.54228141	1.27073634	0.88835103	1.04527023	0.10492449	0.25113571	0.38890097
2.84775156	4.66666967	2.70367706	2.25450441	4.40682866	4.52044285	4.60540621
58.7907719	36.4788967	32.6758685	38.408557	22.4958111	28.3010631	31.6647262
7.36649784	4.18466623	4.30657132	4.44752235	1.80470126	3.11021923	1.3918561
0.09414055	0.81064215	0.71454322	0.69684682	2.91690088	1.35226923	0.59358569
0.30595678	2.8262929	2.08569373	0.90180177	2.49720291	3.70908131	1.69888318
0.40009733	1.48982882	1.6608302	2.27499991	5.09933031	3.09090109	3.58198261
2.98896238	3.6588443	4.13276351	3.99662146	3.02182537	5.44771318	6.44756869
4.68349223	7.58059956	7.49304786	6.10765741	6.98797116	11.3204253	11.2985966
19.1105311	23.3552576	29.3928321	25.8448188	49.9440582	26.1760686	29.658816
825.071293	653.333754	700.677223	631.753128	202.567224	321.840076	325.65339
1121.8729	729.205478	758.362104	726.893214	343.082104	496.321443	506.062503
991.700062	741.321291	764.850929	757.923393	411.576812	619.146125	623.551532
0.4471676	0.61345892	1.02353489	0.96328825	1.09121472	1.39090549	1.61700929
36.455927	52.9984692	54.305285	54.1900879	96.9502306	56.8725801	57.6187488
0.72958924	1.94992301	1.60289426	2.04954947	1.3220486	1.10113351	2.64043289
1.67099472	2.73865591	2.83886092	2.56193684	1.95159555	2.62726593	3.23401858
3.10663806	5.03912688	4.09413955	3.99662146	7.61751812	4.249989	5.64929828
1.20029198	3.17684086	2.58780519	2.86936926	3.48349313	2.76249285	2.68136984
15.5096552	5.10485462	4.38381924	5.22635114	5.33016419	1.41022362	2.0468472
0.28242164	0.7887329	1.21665468	0.84031528	2.28735392	2.62726593	2.96792845
4.75409764	2.56338193	3.05129268	2.47995486	1.00727512	0.54090769	0.79827041
1.31796766	2.47574494	2.18225363	2.50045035	3.8822062	2.66590219	3.17261317
197.295052	117.959387	115.369763	109.097518	75.9863171	108.838355	98.2077288
21.9112124	13.7809165	12.8231541	14.5313057	11.6885884	9.23406702	10.6640739
17.1806499	12.312997	10.3319088	10.3297293	7.57554832	4.61703351	7.08209132
1.7886704	0.50391269	0.65660729	0.3689189	0.75545634	0.21249945	0.61405416
38.7388353	29.3583914	28.7748487	32.567341	7.32372954	4.19203461	5.853983
0.37656219	1.2488271	1.12009478	0.88130627	0.65053185	1.12045165	1.84216248
32.9256565	47.1487002	46.928109	48.9022503	33.8906109	55.674856	66.9319036
85.1030549	122.472692	121.530284	127.60495	119.299147	177.630222	159.019559
96.6588071	66.4945655	67.3408708	71.5292764	46.5235197	59.5578005	63.5955426
6.82518969	9.15806537	8.45864681	10.6371617	16.305266	10.2386099	12.9974797
0.80019465	1.38028258	1.50633436	1.29121616	1.63682207	0.94658846	1.5351354
10.1201088	3.81220903	4.30657132	4.42702685	5.28819439	2.27953956	3.31589247
7.08407619	3.87793677	4.82799475	5.28783763	2.53917271	3.11021923	3.37729789
5.17773011	3.17684086	3.43753227	2.41846837	2.81197638	2.29885769	2.10825262

6.80165455	7.03286838	4.92455465	5.75923401	2.5811425	3.65112692	5.17852343
5.76610853	9.24570236	8.96075826	9.98130591	14.2907158	10.5670181	11.4828128
1.95341636	3.17684086	3.61134008	2.50045035	3.06379517	4.67498791	3.84807274
1.50624876	3.85602752	3.18647654	2.15202694	3.27364415	5.08066867	3.27495553
6.66044373	4.38184946	5.02111454	4.07860344	1.42697309	4.07612582	2.9269915
1.67099472	3.52738881	2.95473279	3.32027014	2.81197638	3.59317252	2.66090136
1.48271362	0.4162757	0.67591927	1.31171166	0	0.25113571	0.59358569
1.45917848	2.84820215	2.24018957	2.13153145	3.02182537	2.99431044	2.2720004
0.80019465	0.89827914	0.50211145	0.84031528	3.46250823	1.98976758	1.20763985
17.1100445	28.4601122	30.0687513	31.7680168	45.2014711	43.8714774	37.3140245
0.40009733	0.32863871	0.84972708	0.65585583	1.95159555	1.13976978	0.28655861
9.60233584	9.66197806	11.6837473	12.5842337	17.4594355	15.9181406	13.6729393
1.57685417	0.81064215	0.71454322	0.61486484	1.02826002	1.04317912	0.40936944
0.18828109	1.68701204	1.1007828	1.84459452	0.25181878	0.98522472	3.09073928
0.77665952	3.81220903	2.27881352	3.83265751	4.11304008	2.66590219	3.39776636
1.62392444	2.36619871	2.64574113	2.2954954	1.69977677	2.24090329	3.27495553
4.80116792	5.21440086	3.84308382	2.82837827	2.32932372	2.26022143	3.72526191
1.15322171	0.85446064	0.50211145	1.04527023	0.56659226	0.59886209	0.98248666
0.4471676	0.89827914	1.08147083	1.02477473	0.33575837	2.08635824	1.5351354
4.75409764	2.76056516	3.88170778	2.95135123	0.71348655	3.4579456	1.59654082
0.54130815	1.11737161	1.15871874	1.31171166	1.3010637	1.85454066	1.47372999
21.5581854	9.33333935	9.57874159	9.55090052	0.65053185	3.90226263	3.70479344
12.4736225	25.7871841	25.6076842	24.9635125	30.5959819	36.3180879	40.8960071
0.82372979	1.29264559	1.75739009	1.41418913	1.86765596	0.94658846	2.12872109
2.56532992	4.8638529	3.90101976	4.48851333	7.55456342	5.87271208	6.54991105
34.7143269	53.6557466	50.7905048	46.4018	75.1888909	55.2112208	58.7445147
141.657989	89.9812786	98.8580206	98.1324285	55.148313	60.2146169	61.48729
217.041032	170.957857	171.297254	172.674543	108.890638	119.134919	126.413283
4.2127895	4.90767139	5.25285829	6.37409885	7.7644124	7.68861647	5.66976675
4.6599571	2.91392989	4.98249059	3.83265751	2.30833882	3.30340054	3.60245108
43.1163707	28.8982972	28.2341133	29.7389628	17.7322391	20.4772197	17.9303815
0.51777301	0.70109591	1.48702238	1.27072067	3.23167435	0.69545275	1.51466693
1.43564335	1.88419527	1.60289426	2.04954947	2.89591598	2.62726593	2.10825262
10.0495034	5.2801286	5.54253798	6.47657632	3.54644783	4.81021483	4.1755683
1.5297839	0.74491441	1.25527864	1.5371621	0.48265266	0.4829533	1.06436055
2.96542724	1.27073634	1.25527864	1.29121616	1.2590939	1.06249725	0.61405416
31.5135482	44.9358662	44.5913596	42.2822055	63.0386348	58.031668	54.9987844
1.29443253	1.59937505	1.79601405	2.72590079	4.42781356	2.1829489	3.00886539
7.24882215	9.11424687	9.19250201	10.3707203	17.2286016	11.6101972	13.7752817
0.82372979	0.8325514	0.48279948	0.24594594	0	0.19318132	0
1.05908116	0.35054796	0.27036771	0.63536034	0.58757715	0.28977198	0.49124333
0.42363246	0.72300516	1.58358228	1.33220715	2.97985557	3.61249065	4.99430718
0.16474596	0.39436645	0.23174375	0.40990989	1.42697309	0.54090769	1.26904527

12.0735252	21.0328774	22.6143274	19.306756	30.1762839	18.9704055	21.4100217
1.50624876	3.39593333	2.91610883	2.47995486	4.95243602	2.6079478	3.17261317
0.54130815	2.03756	2.45262134	1.27072067	1.19613921	2.14431263	2.76324372
7.06054106	3.39593333	2.74230102	4.77545026	4.36485887	2.66590219	2.12872109
0.30595678	0.50391269	0.75316718	0.7583333	1.97258045	0.57954396	1.69888318
3.88329758	1.75273978	1.1007828	2.23400892	1.2590939	2.51135714	2.61996442
0.25888651	0.13145548	0.54073541	0.7583333	1.02826002	1.41022362	1.49419846
2.07109204	2.4100172	2.78092498	2.02905397	2.97985557	3.05226483	3.13167622
1.67099472	0.52582194	0.94628697	0.92229726	0.41969797	0.83067967	0.38890097
6.11913558	4.31612172	5.48460204	3.87364849	1.69977677	3.51589999	5.32180273
20.9227367	11.7652658	13.9239369	12.9941436	10.3455549	13.0976934	10.2956414
1.67099472	0.52582194	0.94628697	0.92229726	0.41969797	0.83067967	0.38890097
15.297839	26.1815505	23.5799264	25.2504494	40.3329747	31.7396906	33.8139158
2.75361101	2.43192645	3.28303643	3.64819805	10.8282076	3.59317252	4.81009093
6.56630318	7.75587354	8.70970254	11.8668914	26.5878663	13.0976934	11.7284345
0.75312438	0.98591613	0.48279948	0.88130627	1.97258045	1.15908791	1.55560387
6.16620586	2.91392989	3.99757966	3.81216201	1.2800788	4.75226043	2.86558609
13.9563362	10.2097092	9.42424576	8.62860326	3.44152334	7.03179999	8.00317257
10.3554602	6.41940946	6.02533745	5.04189169	3.9031911	7.05111812	7.30724452
317.206575	198.322507	213.030441	194.850668	128.868261	142.857585	133.945681
151.354465	53.4366541	58.6504803	59.86734	43.0610115	34.1737752	36.3929433
548.533435	289.684068	308.798545	308.805618	184.793015	192.659729	182.292212
2.75361101	1.11737161	1.1007828	0.94279276	1.13318451	0.88863406	0.96201819
57.1668474	45.7245991	43.6836965	42.9790523	20.8170192	33.8260489	37.5187092
13.7915902	9.66197806	9.81048534	8.34166633	9.1074459	7.26361757	5.95632536
9.24930878	5.54303957	7.28061609	6.84549522	4.11304008	7.08975438	7.6142716
76.0655623	53.1518339	53.37831	53.6981961	26.021274	39.8726241	40.58898
5.20126524	3.13302236	3.97826768	2.86936926	1.23810901	2.6079478	4.62587468
117.769825	82.4225883	87.6763847	81.2031499	47.3839006	58.5339395	55.3262799
9.03749255	3.81220903	4.98249059	3.93513498	1.69977677	2.56931153	1.7602886
0.96494061	1.27073634	2.99335675	1.90608101	3.75629681	3.07158296	2.35387428
53.9189985	44.212861	35.920281	32.3418906	24.7411952	23.4522121	20.9392469
3.15370834	2.49765419	2.91610883	2.74639629	1.00727512	1.64204121	1.43279304
1.01201088	1.22691785	1.17803072	1.39369364	1.72076167	1.79658626	1.82169401
0.23535137	0.4162757	0.96559895	0.3894144	1.78371636	1.12045165	1.20763985
3.01249752	1.79655828	0.98491093	0.57387385	1.84667106	0.46363516	1.37138763
37.6326838	25.7652748	25.0476368	27.5869358	8.22608017	18.2749527	18.687715
651.899756	495.871994	532.740253	475.741423	356.638348	321.859395	329.951769
2.61240019	1.97183226	1.31321457	1.25022518	0.33575837	0.83067967	0.73686499
0.4471676	1.13928086	0.65660729	0.7788288	2.35030862	4.07612582	2.37434276
1.5297839	2.4100172	2.62642915	2.19301793	2.41326331	2.47272088	3.09073928
14.7800659	31.5274069	28.543105	27.1565305	33.6807619	29.2283335	26.1996442
3.36552457	1.48982882	1.85394999	1.1477477	1.40598819	2.14431263	1.57607235

18.3809419	7.42723483	7.68616765	7.21441413	6.25349972	4.82953296	3.84807274
4.89530847	12.4663617	10.7953963	12.2972968	14.7104138	16.3624577	12.5471734
13.1796766	20.550874	21.5714806	21.9711703	25.8953646	24.8624357	25.0329413
1.31796766	1.27073634	2.62642915	1.5371621	5.2462246	4.46248845	4.50306385
41.2335598	62.2003531	64.9268735	40.8475209	73.6360085	53.9748604	72.2741748
0.56484328	1.18309935	1.71876613	1.47567562	2.68606699	2.82044725	1.43279304
1.43564335	2.30047097	1.71876613	1.96756749	1.11219961	3.26476428	2.61996442
3.38905971	7.47105333	6.17983329	6.00517994	7.7644124	6.93520933	8.14645187
1.71806499	3.17684086	3.93964372	3.19729717	5.35114909	4.69430604	4.11416288
490.96649	712.203902	585.481268	652.53556	2882.35973	908.43515	981.545108
128.525382	106.807581	95.0728727	96.7182394	52.3153517	66.3191466	65.2534889
57.8022961	34.7699755	38.1025346	33.8585572	12.4230598	25.4419796	24.3370133
26.0533965	17.2644869	14.6577921	16.6218462	6.75713728	12.9431483	9.57924491
23.5822071	14.26292	18.423628	15.7815309	13.8080631	11.6681516	11.4828128
1.55331903	2.05946925	1.13940676	4.34504487	5.47705848	3.43862747	5.17852343
4.56581655	0.87636989	1.08147083	0.96328825	1.00727512	0.56022582	1.59654082
55.4252473	76.5070916	77.1899801	68.5779252	124.251583	100.029287	94.6666832
31.6782942	17.987492	19.1574832	20.3110352	7.47062383	15.8408681	16.3133722
15.7685417	12.6635449	11.4520036	9.55090052	7.7853973	7.30225384	5.21946037
3.10663806	6.24413548	7.53167182	7.99324293	5.54001318	5.52498571	7.22537063
58.131788	39.6776468	40.3041002	40.1096831	41.2353253	30.0590131	26.1996442
0.14121082	0.70109591	1.23596666	1.74211705	0.0629547	1.08181538	0.85967583
0.35302705	0.4162757	0.86903906	1.25022518	1.15416941	2.10567637	1.61700929
11.7675684	18.075129	16.569678	18.1385128	18.991333	21.4431263	19.8953548
108.991219	58.1690516	58.7277282	56.8135113	48.7269341	46.4214708	42.2059893
3.5773408	2.38810796	2.18225363	1.63963957	1.17515431	1.39090549	1.04389207
15.5802606	23.4428946	23.4061186	24.5945936	21.068838	29.749923	29.2494465
0.56484328	0.59154968	1.23596666	0.92229726	1.42697309	0.86931593	0.8801443
6.30741668	4.33803096	3.51478018	3.62770256	1.17515431	3.92158076	2.70183831
1.22382712	2.43192645	1.91188592	1.59864859	1.90962575	2.24090329	2.35387428
27.3948993	43.5774929	45.2093429	39.4538273	35.9471309	45.7453362	44.4984582
183.362251	120.763771	120.931613	115.881527	91.284308	82.3725142	89.7747183
73.3590216	48.200344	47.4688444	44.7826559	33.5548525	30.4067395	32.4015912
75.9949569	49.2081694	51.4084881	51.0952682	31.5822721	35.1783181	36.1473216
2.28290827	2.80438365	2.3946854	2.43896387	3.35758374	2.83976538	3.70479344
92.9167203	50.500815	57.08621	54.0466195	48.9997377	31.4306005	28.6558609
105.413878	341.893804	330.640393	341.59841	609.506374	685.407318	729.025569
21.1816232	27.1017389	30.4549909	26.93108	45.2644258	30.3874214	32.2378435
0.6354487	1.16119011	1.9311979	1.88558551	1.59485228	1.25567857	1.37138763
58.7672367	38.363092	33.7573393	37.2608093	28.6863561	27.7408373	25.8107432
66.1101994	31.7684086	31.0536623	29.0626115	19.2641367	19.2215412	15.5560387
12.1441306	5.95931526	7.06818432	5.28783763	6.67319769	3.12953736	4.64634315
6.75458428	3.41784258	3.8623958	3.36126113	3.10576496	2.26022143	2.25153192

41.7748679	21.8216103	23.2709347	24.4716206	16.6410244	14.3920082	18.5239672
84.6323521	55.6494881	59.5002074	49.1072053	54.7076301	33.7487763	30.6208342
0.77665952	1.07355312	1.21665468	1.10675671	1.11219961	1.08181538	2.21059498
2.07109204	0.56964043	1.04284687	0.79932429	0.67151675	1.62272307	1.10529749
0.82372979	1.48982882	1.12009478	1.68063056	1.65780697	2.35681208	1.41232457
18.6398284	12.1596322	13.1707697	13.9574319	9.1074459	11.1465621	11.6670291
50.9771064	32.5352322	35.862345	35.7236472	33.4918978	31.1215104	28.8400771
1.29443253	0.21909247	0.32830364	0.08198198	0.31477348	0.61818022	0.20468472
8.61386009	12.7292727	11.3361317	11.9488734	28.287643	19.4340406	17.0707057
132.338075	91.5368352	101.426514	91.0204919	53.7633097	81.464562	88.1781775
0	2.76056516	2.83886092	2.47995486	0	0	1.35091915
11.6263576	6.35368172	5.40735413	4.69346828	4.13402498	1.42954176	1.45326151
12.4030171	22.3693415	22.3246477	21.9506748	28.707341	25.5385703	34.9601502
0.28242164	0.46009419	1.46771041	1.84459452	1.82568616	1.95113132	2.76324372
5.90731935	3.15493161	3.64996403	3.15630618	5.56099807	1.83522252	2.00591026
40.2686192	28.5039307	32.0192612	31.5835573	15.5707946	21.6749439	20.8983099
26.5476344	41.7152068	42.5442898	39.5563047	47.7196589	33.1885505	37.7847994
343.660068	216.989185	218.128803	225.901342	77.1195016	136.308738	125.000959
0.82372979	1.94992301	1.60289426	1.1477477	2.24538413	2.06704011	1.78075707
24.076445	14.1971922	17.0717895	13.7934679	10.9331321	9.77497471	7.43005535
23.2291801	17.4397608	15.2757754	14.5313057	8.81365732	12.9238302	14.4507413
6.63690859	4.90767139	3.37959633	4.57049531	6.42137891	4.86816922	4.4621269
7.97841139	14.7887419	17.6704608	17.277702	38.2974396	34.3476384	40.4866377
0.37656219	1.05164387	1.46771041	2.27499991	2.45523311	2.89771978	2.21059498
3.55380567	3.08920387	2.89679685	3.05382871	0.92333553	2.04772197	0.8801443
763.244488	491.205324	504.004028	483.181287	288.122655	291.259474	263.163145
0.94140547	0.48200344	0.86903906	0.96328825	3.56743273	3.22612802	2.66090136
42.8104139	10.6259849	9.30837389	10.3502248	8.64577814	5.23521373	7.51192924
1.20029198	0.59154968	0.8304151	0.43040539	0.37772817	0.61818022	0.40936944
7.93134112	3.63693505	5.523226	4.77545026	5.74986216	3.74771758	4.19603677
7.29589242	12.1596322	10.5636525	11.3135131	14.8573081	12.0351961	13.0998221
10.0495034	15.4460193	15.1792155	16.2119363	25.7484703	24.9976626	22.3720399
12.379482	17.4178516	15.642703	18.7328821	31.7501513	17.6181362	22.2287606
130.925966	103.455466	98.529717	93.6849062	57.2258179	63.8077895	71.2098142
0.98847575	1.38028258	1.25527864	2.13153145	4.25993437	3.78635384	4.11416288
28.8540778	43.1393079	42.8532814	42.9585568	77.7910183	43.1180703	41.5714667
17.1806499	21.9311565	21.9384082	21.9711703	53.9731587	27.7601555	30.00678
5.22480038	11.3270809	9.4821817	9.20247711	11.5207092	9.07952196	7.22537063
3.88329758	7.07668688	4.11345153	6.1486484	8.83464222	6.22043845	8.39207353
0.56484328	2.23474322	2.25950155	1.35270265	0.83939594	1.21704231	1.63747776
0.8943352	2.08137849	1.12009478	1.29121616	1.99356535	1.35226923	1.61700929
2.02402177	1.22691785	0.90766301	0.65585583	0.33575837	0.56022582	0.3684325
14.4270389	9.81534279	11.2975077	9.6328825	4.74258704	6.87725493	4.89196482

10.0965737	18.162766	18.7326196	15.0436931	17.3754959	23.7806203	25.974491
11.3204008	6.63850193	6.89437651	5.51328807	7.55456342	5.23521373	4.8305594
42.8104139	25.8090933	28.0216816	27.7099088	24.2375576	27.1806115	22.6585985
0.75312438	0.46009419	0.50211145	0.55337836	0.37772817	0.56022582	0.77780194
108.120419	67.8967574	75.471214	68.5779252	59.1144588	49.4737356	54.7531627
1.01201088	0.15336473	0.38623958	0.53288286	0	0.09659066	0.1432793
17.2512553	24.7355402	24.2558456	24.3076567	43.8164678	26.6203857	23.1907788
234.362893	173.170691	182.923065	182.061479	78.6094294	143.282584	131.509933
12.4030171	22.062612	20.3934498	20.1470713	24.4264217	22.6988049	26.8136984
6.23681127	9.92488902	12.5720983	10.7806302	9.86290224	11.8226967	12.8132635
0.09414055	1.09546236	1.37115051	0.7788288	3.02182537	1.41022362	1.49419846
0.16474596	1.81846753	0.42486354	0.22545044	2.32932372	1.2749967	0.75733347
0.09414055	0.81064215	0.7338552	0.3689189	1.48992779	1.13976978	1.41232457
2.25937314	4.75430666	2.35606144	3.42274761	4.57470785	5.98862087	3.62291955
32.0548564	19.8716873	14.4839843	16.2324318	12.2971505	11.9386055	12.1582724
0.32949192	1.09546236	0.77247916	0.57387385	1.55288248	0.79204341	2.12872109
0.21181623	1.44601032	0.54073541	0.61486484	1.15416941	0.65681648	0.53218027
1.81220554	0.7887329	0.63729531	0.3894144	0.83939594	1.12045165	1.65794623
4.98944901	6.66041118	7.10680828	6.64054028	8.91858182	9.56247526	7.71661396
2.02402177	1.53364731	1.68014217	0.79932429	0.92333553	1.44885989	0.63452263
15.9097525	23.7715333	23.039191	20.6184676	31.9180305	35.8158164	32.0740957
22.0524232	30.9577664	34.3946346	33.2641879	46.4185952	31.3340098	33.7525104
17.0865094	13.3646408	9.21181399	12.4817563	8.72971773	10.6249725	10.5412631
9.36698447	7.55869032	8.42002285	7.50135105	1.44795799	3.55453626	4.09369441
13.932801	8.85133591	8.53589473	6.82499973	7.23978994	6.18180219	5.46508203
0.98847575	2.93583914	2.68436508	2.88986475	2.24538413	4.67498791	3.58198261
1.76513526	2.62910968	3.43753227	3.13581069	5.1832699	4.07612582	3.56151413
33.2316132	22.0407028	25.3566285	24.7790531	17.9001183	15.976095	18.5649041
54.3897013	39.5242821	34.7615622	38.0806291	21.4675511	35.0817274	30.5594287
7.22528701	3.72457204	4.05551559	4.18108091	2.95887067	5.35112252	5.81304606
10.0495034	6.30986322	6.95231245	7.80878347	2.97985557	6.45225603	7.12302827
349.590923	227.04553	228.557272	227.950892	121.964229	154.912099	157.238802
122.217966	84.964061	79.2563619	82.699321	46.0198822	55.1919027	61.9785333
74.0650757	46.86388	48.7048111	46.0533765	31.9390154	35.8351346	36.7818442
40.9982084	29.7527578	31.266094	32.9772509	22.9364939	20.8829005	21.6147065
0.37656219	1.16119011	1.46771041	1.1682432	2.18242943	0.83067967	1.14623443
232.997855	180.335014	181.532603	168.493462	83.5198956	134.318971	124.837211
34.4083701	27.4741961	22.6915753	22.4425667	15.0042023	16.9613198	18.1145978
499.156718	209.540041	213.184936	206.061704	131.701222	125.355358	129.606365
25.4885532	35.7778008	35.4374815	30.6817555	51.1821672	39.6408065	43.8434671
62.085691	94.2535819	89.9551983	94.6481944	155.099384	94.214529	101.851117
42.7633437	66.7355673	58.2642407	62.3063038	122.530822	65.1021043	68.2828227
173.477494	228.228629	207.352719	217.94909	309.69513	388.661494	421.875677

7.39003297	3.81220903	5.0597385	5.04189169	2.11947474	3.14885549	4.40072149
53.7071823	110.181605	117.996192	115.82004	117.998084	125.026949	125.92204
13.5327037	8.65415268	9.77186138	9.20247711	6.14857523	5.85339395	5.05571259
45.9876574	33.8936056	30.9184784	29.3905394	18.5296653	16.2272307	17.561949
45.7758412	31.1111312	31.3433419	33.7765752	18.4457257	30.6965115	31.5009785
311.769958	201.083072	204.745602	188.210128	137.241235	148.401889	163.993398
212.734102	163.57444	168.748073	168.431975	97.8525812	111.388348	121.582724
9.08456283	6.09077075	6.39226506	6.25112588	4.02910049	4.44317032	6.26335244
24.594218	28.6353862	30.1846232	27.6074313	52.9029288	37.3226307	46.4839
1.81220554	1.00782538	0.7338552	0.69684682	0.56659226	0.81136154	0.57311722
7.81366544	11.8090843	12.8231541	13.7114859	20.7120947	15.1840516	16.6408678
10.0495034	16.4538447	12.9969619	14.8592336	13.9969272	18.7385879	19.4655169
7.90780598	3.17684086	4.57693903	3.13581069	1.97258045	3.86362637	3.64338802
13.3679577	6.76995741	7.74410359	7.54234204	4.88948133	7.72725274	7.71661396
14.097547	8.63224343	7.68616765	7.21441413	5.03637561	4.23067087	6.77506424
8.73153577	3.81220903	4.38381924	6.23063038	4.74258704	2.58862967	3.41823483
86.9858658	58.7825105	54.6335886	50.13198	44.6978336	46.8464697	46.6885847
168.817537	106.917127	111.410807	109.978824	55.4001318	78.3736609	74.6894545
21.2051583	25.1956344	25.7042441	26.5006746	51.6438349	34.734001	36.6999704
8.00194653	3.92175527	3.5920281	2.00855848	1.93061065	1.10113351	1.22810832
85.809109	33.6964223	45.8466382	44.7826559	52.5042158	49.3578268	45.5832872
0.68251897	1.77464903	0.98491093	1.06576572	1.13318451	0.75340714	1.33045068
0.77665952	1.40219183	1.91188592	2.21351343	3.84023641	3.67044505	3.88900969
261.946073	140.219183	140.687767	134.798869	43.5226793	83.1259213	74.4438328
15.6744011	11.0860791	13.0548978	11.1495491	6.82009198	11.5329247	11.360002
6.56630318	6.98904989	8.22690306	8.81306271	16.5570848	12.8079214	12.9156059
0.51777301	1.11737161	0.34761562	1.06576572	2.39227842	0.92727033	0.79827041
13.250282	20.1784168	19.7368426	19.0403146	22.5797507	24.9203901	31.1939514
196.471322	267.380454	261.445572	253.508774	475.05613	288.226527	306.167405
6.42509236	12.2472692	7.26130411	7.00945918	8.37297446	15.1067791	11.2576596
52.0832579	30.0594873	29.8370076	33.0797284	22.8315694	26.3499318	28.0418067
36.5029973	22.8513449	24.0820378	22.1351343	24.6152858	22.6794868	21.3690848
4.2127895	2.91392989	3.34097237	1.90608101	2.53917271	1.04317912	1.7602886
9.41405474	12.8607282	14.9088478	12.8096842	16.7669338	19.9942665	20.6731568
9.79061693	7.44914408	5.00180257	6.33310786	3.8612213	4.77157856	4.29837913
36.5500675	21.5806086	23.5026785	26.0702692	14.1857913	19.9363121	19.0970844
148.647924	205.223919	216.448661	211.861929	213.899069	243.698233	230.843428
0.98847575	0.04381849	0.23174375	0.34842341	0.81841104	0.15454505	0
8.84921146	13.5180056	13.4604494	14.6952697	15.7596587	15.8988225	15.8221289
2.30644341	3.2425686	3.88170778	4.18108091	5.93872624	4.90680549	3.68432497
114.639652	80.2973913	80.3378327	81.8999967	97.3069738	63.440745	57.3526586
4.77763278	2.71674666	4.24863538	4.03761245	3.8822062	3.92158076	4.11416288
20.3108231	12.137723	14.7929759	14.1828823	10.7232831	14.952234	13.570597

0.49423787	1.00782538	0.44417552	0.92229726	2.70705189	1.39090549	0.92108124
1.5297839	1.81846753	3.51478018	2.50045035	3.98713069	3.99885329	3.33636094
5.22480038	7.3176886	7.14543224	7.56283754	7.70145771	7.7079346	8.31019965
3.1301732	1.18309935	0.81110312	1.1682432	2.07750494	2.49203901	2.02637873
28.7599372	20.0688705	20.9728092	17.7286029	24.573316	18.0817714	14.1846511
84.8677035	38.9765509	40.9993315	33.5101338	15.0251872	16.9806379	15.6583811
9.62587097	5.6964043	5.81290568	5.92319796	4.63766254	5.66021263	4.3188476
1.10615143	0.30672946	0.57935937	0.47139638	0.16787919	0.3670445	0.67545958
31.1369861	71.8185126	72.4778573	70.8529251	128.532503	120.912187	112.556128
0.94140547	2.71674666	2.33674946	1.94707199	3.18970456	7.43748076	6.38616328
11.9087792	9.50861333	8.82557441	9.46891854	8.70873283	4.3079434	5.17852343
66.1572697	50.4789058	47.1598528	46.7092324	44.3201054	35.603317	34.4689069
5.50722202	8.71988042	8.30415098	7.48085556	10.4714643	11.6101972	8.86284839
0.54130815	0.7887329	0.94628697	1.43468463	4.02910049	2.22158516	2.08778415
18.8045743	7.79969204	7.26130411	6.57905379	7.38668423	5.52498571	4.99430718
45.2109979	21.2957884	19.1188592	20.3520262	12.4020749	11.6874698	11.8512453
244.318256	195.737215	209.322541	210.591208	81.5683	123.771271	125.512671
0.21181623	0.15336473	0.44417552	0.49189187	1.21712411	0.92727033	1.41232457
2.80068129	4.68857892	5.04042652	5.14436916	4.36485887	5.04203241	5.3422712
36.3617864	21.7777918	22.7301993	25.7218458	20.7540645	19.8397214	22.1264183
1.74160013	4.16275699	5.46529006	3.853153	6.10660543	9.13747636	6.12007314
2.07109204	2.69483742	3.90101976	4.11959443	2.30833882	3.99885329	3.07027081
8.82567632	9.46479483	8.51658275	11.6619365	13.1365464	13.3874654	17.213985
537.071823	398.551118	390.314408	416.468452	340.75278	319.000311	330.934256
2.09462718	3.1987501	2.6650531	3.50472959	6.5682732	2.99431044	3.56151413
59.1908692	44.82632	43.9347523	45.2540523	35.7792518	31.5271912	28.8400771
70.6289457	40.6416537	41.0379554	41.0934668	10.6603284	22.0999428	19.7930125
91.9753148	52.3192825	50.1532095	55.1123852	35.6113726	34.289684	35.7174837
7.6018492	2.05946925	2.37537342	3.73018003	0.48265266	1.00454286	2.16965804
78.2072598	57.9718683	60.6589261	63.4950425	30.9946949	47.5998769	47.220765
14.3093632	9.44288558	12.1279228	10.7396392	10.8701774	8.78974999	9.68158727
11.17919	4.42566795	4.96317861	5.0009007	3.42053844	5.2158956	4.87149634
1.64745958	1.53364731	0.75316718	0.24594594	0.27280368	1.06249725	0.5117118
16.4510607	8.1064215	8.69039056	6.78400874	8.93956671	5.87271208	3.39776636
2.35351369	3.06729462	2.16294165	3.68918904	3.75629681	5.37044065	4.8305594
12.0499901	19.0610451	17.1490374	15.7200444	26.0002891	23.3556214	23.804833
17.1806499	10.0782538	11.9154911	11.395495	10.0727512	9.42724834	9.06753311
3.03603265	5.76213204	5.19492236	4.54999982	13.2624558	8.6158868	7.90083021
2.49472451	4.64476043	2.45262134	3.29977464	3.81925151	4.32726153	4.70774857
8.42557899	10.2535277	11.046452	11.8054049	13.3044256	14.7590527	12.0763985
30.5015374	40.9702924	39.0681336	39.8432417	80.7918588	46.7885153	45.2353232
0.98847575	0.8325514	1.50633436	1.08626122	2.6231123	1.56476868	2.96792845
138.998518	97.9781539	98.0662295	101.30923	71.2227451	77.5816175	81.1779601

3.08310293	2.91392989	2.24018957	2.07004496	3.33659884	1.46817802	0.83920735
3.3184543	1.90610451	2.37537342	1.84459452	1.53189758	1.93181318	1.30998221
1.90634609	0.65727742	0.75316718	0.69684682	0.18886409	0.59886209	0.34796402
9.22577365	5.01721763	4.55762705	2.84887376	0.88136573	1.73863187	2.19012651
0.58837842	1.22691785	0.67591927	0.79932429	1.09121472	0.8499978	1.96497332
5.60136257	5.93740602	5.79359371	6.21013489	8.05820098	11.2045165	14.9010476
0.84726493	1.97183226	2.761613	1.90608101	4.80554173	2.26022143	2.53809053
410.429252	289.004881	275.852308	301.099312	235.576469	226.273278	238.723789
25.0178505	9.42097634	15.0633436	12.9531526	6.5682732	6.68407362	7.14349674
16.9217634	10.4068925	8.88351035	10.0632879	6.5472883	7.53407142	8.71956909
6.75458428	13.2989131	11.4906275	10.2067564	20.1664874	9.65906592	10.9096956
1.20029198	3.46166107	3.14785258	3.64819805	1.65780697	5.77612142	4.27791066
2.37704882	1.42410107	1.40977447	0.90180177	0.96530533	1.02386099	0.5117118
53.307085	33.5868761	39.608869	36.4819805	26.021274	22.5828961	25.0943467
8.09608708	5.47731182	3.61134008	5.4108106	3.14773476	2.45340274	2.19012651
5.38954634	13.7809165	13.3445775	12.0513509	5.2672095	8.34543295	6.7341273
14.9918822	9.83725203	8.63245462	12.0103599	2.91690088	7.63066208	11.7489029
12.1912009	10.7355312	9.92635722	9.44842305	2.26636903	6.56816483	7.67567701
1.55331903	1.40219183	0.52142343	0.55337836	0.52462246	1.73863187	0.30702708
5.22480038	1.70892129	3.53409216	2.07004496	1.3640184	3.96021703	3.68432497
8.09608708	4.25039398	4.28725934	2.56193684	1.15416941	2.6079478	2.49715359
1.10615143	0.28482021	0.17380781	0.34842341	0	0.73408901	0.34796402
131.514345	83.3865952	86.1700504	93.4594557	61.758556	58.9782565	58.580767
0.07060541	0	0.32830364	0.30743242	1.00727512	1.25567857	1.49419846
25.9121857	39.3490081	34.7229383	41.8313046	32.3377284	44.5089758	52.3174145
109.556062	52.4069195	53.2238142	49.1481962	36.8494816	30.2135582	27.7143111
11.2027251	5.45540258	6.75919266	5.67725203	3.42053844	3.43862747	4.70774857
1.74160013	1.81846753	4.46106715	2.84887376	5.33016419	2.9749923	3.66385649
0.75312438	2.10328774	0.67591927	1.63963957	2.30833882	1.75795	1.41232457
3.67148135	5.49922107	5.8901536	6.21013489	3.21068945	3.92158076	5.73117217
10.9438386	8.34742322	7.16474422	7.66531501	1.53189758	2.37613022	2.00591026
0.21181623	1.00782538	1.15871874	1.1682432	3.10576496	2.87840164	1.73982012
3.36552457	2.08137849	1.89257394	2.6849098	1.2590939	1.31363297	2.19012651
7.22528701	4.8638529	4.92455465	4.6319818	4.21796458	3.78635384	3.35682941
9.81415207	3.85602752	4.59625101	4.71396378	3.42053844	2.04772197	1.14623443
2.07109204	4.40375871	3.8623958	3.36126113	3.84023641	5.19657746	5.21946037
3.5773408	1.92801376	1.89257394	2.54144134	0.94432043	1.2749967	0.73686499
22.5466611	11.787175	11.992739	13.301576	6.00168094	9.96815603	9.98861435
26.3358181	15.3583824	15.7778869	16.1504498	11.562679	10.6249725	12.6290472
37.5620784	32.5352322	24.0820378	27.5254494	12.5489692	20.6704011	20.3251927
105.013781	74.9296257	68.9051411	65.4011235	77.2244261	57.587351	62.224155
21.0404123	29.4898469	27.8864977	26.0702692	53.1757325	43.1567065	41.1211603
1.0826163	1.77464903	1.40977447	1.49617111	2.30833882	1.71931373	2.08778415

34.0788782	23.5962593	23.4061186	24.0002243	9.98881163	22.0999428	25.237626
5.78964367	6.87950365	8.94144629	7.84977446	8.49888385	11.3204253	8.69910062
4.70702737	7.64632731	7.99515931	6.59954929	5.58198297	6.79998241	7.26630757
34.2671593	47.4554296	44.5720476	42.6921154	79.6167045	55.2691752	60.4843349
1.90634609	1.9937415	0.77247916	1.06576572	0.79742614	1.06249725	1.04389207
182.938619	97.014147	110.232776	100.325446	72.3559297	64.0782433	64.8441194
114.686722	70.2848653	72.2847375	69.1722945	53.3645966	44.103295	46.0745306
284.704551	216.682456	212.605577	204.48355	173.733974	158.31209	164.709794
159.662368	113.774721	111.603927	116.803824	102.700093	94.7747548	99.0059992
5.50722202	7.25196085	9.30837389	8.62860326	11.4787394	12.5181494	12.9770113
1.36503794	0.61345892	0.86903906	0.67635132	0.56659226	0.94658846	0.67545958
48.6471279	37.9906348	34.8581221	35.3342328	21.2996719	21.1533544	24.6031034
86.8211199	60.951526	57.8973131	55.0508987	40.0391861	45.2044285	39.4222771
113.227543	214.732533	219.133026	186.693461	357.309865	230.928948	226.442706
225.23126	176.325622	171.606246	166.853822	69.0822855	118.651966	121.930688
29.4895265	19.5211393	20.9148733	18.8558551	12.5279843	15.531778	13.6934078
43.2105113	78.6541978	77.2672281	76.3457177	139.570559	86.9702295	75.1192924
3.85976244	7.38341634	5.58116194	6.43558533	8.93956671	10.992017	8.88331686
0.28242164	1.00782538	0.61798333	0.28693693	5.45607358	0.88863406	2.7837122
4.14218409	0.7887329	1.69945415	2.07004496	2.20341433	1.46817802	1.55560387
20.0284015	23.9687165	30.1073753	25.5373864	38.2554698	28.4362901	31.2553568
1.60038931	1.55555656	2.761613	2.84887376	6.21152992	2.66590219	4.01182052
19.5341636	31.8779548	33.9697711	26.3572062	44.194196	28.861289	27.3049417
3.48320025	2.01565075	1.1007828	1.51666661	0.41969797	1.12045165	1.88309943
11.2027251	6.98904989	7.74410359	7.54234204	6.69418259	5.48634944	5.28086579
21.4169745	13.912372	14.3488004	15.289639	8.05820098	13.6772373	15.392291
9.60233584	14.087646	15.7006389	14.5313057	17.0817073	20.6510829	19.1994268
21.7229313	12.9264559	9.98429315	10.1452699	10.6603284	10.7601994	8.57628978
0.18828109	0.59154968	0.86903906	0.51238737	1.42697309	0.98522472	1.33045068
6.37802209	9.26761161	8.55520671	8.79256722	15.1091268	8.80906812	10.0295513
28.6422616	21.1205144	19.9106504	17.9745488	9.1284308	15.3772329	13.5091915
8.54325468	5.67449505	6.27639318	8.32117084	7.42865403	2.16363077	4.76915398
25.8886505	14.7449234	18.9450514	17.9335578	17.2915563	14.6624621	11.2576596
28.5010507	18.4694955	18.8291795	17.5031525	14.3746554	13.1556478	13.2840384
0.37656219	0.3724572	1.06215885	0.59436935	1.78371636	1.44885989	1.96497332
7.50770866	3.11111312	4.21001143	4.32454938	1.97258045	2.74317472	2.74277525
7.22528701	3.15493161	3.18647654	2.41846837	3.65137232	2.37613022	2.7837122
346.743171	563.922116	570.47586	554.567095	557.170037	538.222471	490.1585
6.21327613	5.08294537	4.48037913	5.26734213	5.162285	2.31817582	3.19308164
12.1441306	4.62285118	7.95653536	6.96846819	6.96698627	4.81021483	4.99430718
27.3007588	15.2707454	16.4731181	18.0770263	6.4633487	12.9045121	13.3249753
3.22431375	4.88576215	4.11345153	4.57049531	3.79826661	5.08066867	5.95632536
0.54130815	0.70109591	1.58358228	1.37319814	1.40598819	1.56476868	2.68136984

5.48368689	8.80751741	8.24621504	7.09144116	10.114721	12.2283775	13.3454438
1.95341636	4.8200344	4.09413955	4.32454938	1.40598819	6.31702911	10.8278217
0.91787034	2.54147269	1.37115051	1.39369364	2.07750494	2.87840164	3.99135205
1.76513526	0.92018839	1.13940676	0.90180177	0.39871307	0.30909011	0.26609014
28.1715588	22.1940675	21.5135446	21.5817559	8.72971773	11.2624709	10.6845424
4.16571922	8.19405849	8.57451868	9.14099063	7.8063822	10.7601994	10.3775153
10.0730386	14.3286477	14.3294884	12.8711707	21.1737625	17.0579104	16.5180569
639.967441	393.5339	391.878678	396.09593	403.707475	361.635428	368.596244
11.17919	5.17058236	6.3536411	4.07860344	2.43424821	2.9749923	3.04980233
146.97693	116.995381	115.756002	112.725221	65.263034	65.35324	59.9930915
114.192484	164.582266	158.165108	170.543011	182.568616	189.916554	176.41776
13.5327037	26.9921927	33.100732	26.5416656	59.471202	53.2407713	53.1156849
28.6893318	20.9671497	19.6982186	18.3229722	9.92585694	14.2567813	12.2606147
470.961624	312.951688	305.8245	304.419582	226.15425	216.63353	203.640828
173.359818	118.375663	113.226133	115.963509	67.3405389	90.4474933	96.0790077
24.3588666	15.7965673	16.6469259	14.9207201	6.4843336	12.1317868	8.31019965
14.0269416	8.25978623	7.51235984	7.3373871	3.14773476	4.07612582	4.95337023
114.098343	83.5180507	83.0608218	85.4252218	40.5218388	69.7577741	67.9348587
87.8331307	63.7559096	55.4833157	51.9765745	33.0302301	36.1635428	33.6092311
45.2580682	59.6150619	59.1912157	57.756304	103.182745	76.9441191	70.247796
59.0967286	41.7590253	40.8834596	41.8518001	19.5789102	33.2851412	38.419322
18.6162933	23.5086223	25.1828206	27.2385124	40.8575972	30.0010587	26.6908875
69.2639078	52.0782808	53.7259256	51.4846826	31.6452268	41.1862571	40.5071062
7.86073571	4.40375871	4.53831507	3.77117102	3.31561395	2.5499934	2.21059498
18.2868013	26.4663707	22.5563915	26.7466206	43.0819964	27.837428	33.650168
0.21181623	0.39436645	1.21665468	0.79932429	0.31477348	1.37158736	1.43279304
56.9785663	79.9030249	77.2092921	75.5258979	152.854	86.1009136	95.6082329
25.1590613	34.9890679	36.6927601	32.1574312	63.3324233	41.1283027	38.2555742
21.4169745	28.2191105	32.9655482	29.7389628	53.4275513	32.6669609	35.1443665
2.04755691	4.22848473	4.96317861	3.29977464	6.96698627	5.17725933	3.62291955
0.28242164	1.31455484	1.12009478	0.92229726	0.48265266	1.25567857	0.94154971
23.040899	8.4788787	10.6409004	11.5799545	4.49076826	4.42385219	6.54991105
28.3363048	51.4210034	52.1616553	49.8245476	59.3662775	67.4395983	70.9641925
6.82518969	10.275437	8.36208692	9.30495458	8.64577814	11.9192873	10.7868848
55.8253446	68.9045827	71.0680828	68.6599072	220.782116	178.402948	193.447529
5.76610853	1.40219183	1.33252655	1.33220715	1.17515431	0.54090769	0.65499111
11.9558495	9.33333935	10.2353489	7.39887358	3.924176	7.61134394	6.7341273
7.88427085	4.84194365	3.90101976	3.89414399	3.65137232	3.65112692	5.26039731
8.56678982	13.0798206	14.0204968	12.0923419	14.0179121	13.8704187	15.9244712
13.2738172	11.1956254	9.07663014	8.25968436	9.63206836	9.65906592	5.73117217
56.9550312	87.176895	81.4579275	82.1459427	85.8911891	107.23495	102.772198
0	0.30672946	0.63729531	0.53288286	2.43424821	2.08635824	2.35387428
0	0	0.40555156	0.3689189	1.65780697	2.22158516	0.90061277

0.21181623	0.81064215	0.48279948	1.20923419	2.43424821	1.25567857	2.02637873
0.25888651	0.67918667	0.52142343	0.63536034	2.77000659	1.37158736	0.71639652
168.841072	116.688651	119.714958	114.938734	66.7949316	66.3577829	77.0637972
19.2752771	31.2425866	27.3071383	32.1164402	31.4983325	37.9601291	36.3315379
2.49472451	0.54773118	1.35183853	0.57387385	0.67151675	1.00454286	0.20468472
15.7920768	10.9984421	11.007828	11.4569815	7.8273671	8.44202361	10.234236
24.5000775	28.6572955	32.251005	32.8747735	53.0078533	43.4078422	39.1152501
1.76513526	2.8262929	2.6650531	1.84459452	2.09848984	3.18749175	3.11120775
124.477339	72.6072455	70.7011552	72.0211683	46.4395801	39.3510346	40.5685116
0.18828109	0.30672946	0.57935937	0.3689189	1.3640184	1.15908791	1.24857679
0.18828109	0.30672946	0.57935937	0.3689189	1.3640184	1.15908791	1.24857679
0.18828109	0.30672946	0.57935937	0.3689189	1.3640184	1.15908791	1.24857679
0.18828109	0.30672946	0.57935937	0.3689189	1.3640184	1.15908791	1.24857679
0.18828109	0.30672946	0.57935937	0.3689189	1.3640184	1.15908791	1.24857679
2.30644341	4.68857892	4.63487496	5.12387367	10.114721	4.26930714	6.03819925
1.90634609	1.75273978	0.92697499	0.7583333	0.33575837	1.3329511	0.57311722
32.9256565	21.2519699	22.4791436	17.7286029	21.0478531	14.8170071	17.6847598
0.04707027	0.28482021	0.75316718	0.3689189	3.35758374	2.93635604	3.78666733
1.01201088	2.34428946	1.60289426	0.98378374	3.48349313	1.64204121	1.94450484
10.4496008	7.71205505	7.62823171	7.89076545	4.09205519	5.71816702	4.9738387
70.6995511	34.9671587	30.1073753	37.5682417	10.1986606	9.23406702	15.8835343
101.154018	60.7981612	70.3728515	66.0364839	68.9144063	58.4373488	54.507541
8.02548167	2.01565075	2.14362967	1.94707199	0.94432043	1.25567857	1.5351354
0.47070274	0.81064215	0.86903906	1.74211705	4.84751153	3.43862747	3.68432497
3.90683272	2.4100172	2.62642915	3.19729717	3.44152334	1.95113132	2.14918956
18.1455905	10.9984421	10.3319088	13.7524769	5.2672095	10.4704275	13.0793536
225.584287	161.142514	159.613507	170.461029	126.832726	134.724651	127.334365
151.401535	201.170709	205.885008	194.789181	330.449195	241.978919	245.130421
20.805061	30.738674	28.0409935	24.6150891	38.0666057	36.3760423	37.0069974
29.8425535	18.8200434	20.2003301	18.4664407	18.2778465	14.1022362	14.1437142
1.95341636	3.54929806	3.32166039	3.77117102	7.28175974	5.19657746	4.05275746
19.9107258	11.962449	16.1834384	14.2238733	5.09933031	14.6238258	9.78392963
6.40155722	15.862295	15.5268311	16.5808552	13.5982142	18.757906	15.1876063
0.68251897	1.31455484	1.23596666	1.5576576	1.65780697	2.06704011	2.12872109
41.3512355	24.3849922	26.0518597	25.8038278	30.9527251	15.1260972	15.8630658
8.19022763	11.6338103	12.6879702	12.6867112	16.8298885	11.6874698	13.52966
2.21230286	5.49922107	4.30657132	3.83265751	2.6440972	4.86816922	4.13463135
68.1342212	43.3364911	43.0657132	42.9585568	40.6477482	42.055573	35.0420241
2.35351369	0.67918667	1.31321457	1.61914408	0.48265266	1.19772417	0.8801443
148.577319	289.114427	273.129319	300.935348	315.130219	370.792223	367.102046
12.8031144	6.98904989	8.90282233	8.03423391	2.20341433	4.59771538	5.64929828
5.50722202	3.57120731	4.17138747	5.24684664	2.07750494	3.49658186	4.01182052
15.2036984	6.59468344	8.51658275	6.70202676	6.5053185	6.8579368	6.38616328

78.7956382	58.4100533	58.3608006	56.9774752	25.202863	41.4180747	41.6328721
8.00194653	17.9217643	19.0222993	19.2042785	14.6894289	15.9954132	17.0502372
6.51923291	3.70266279	3.82377185	4.79594576	1.2590939	3.36135494	2.14918956
20.0519366	27.6932886	23.1550628	30.1078817	42.0747213	30.9283291	34.5098439
1.55331903	8.36933247	10.9885161	8.54662128	21.1737625	16.8647291	17.2344535
3.43612998	3.41784258	5.90946558	5.65675653	8.75070263	6.83861867	7.69614549
148.200757	98.5039758	96.9847586	96.2673385	60.3945376	81.5418345	80.3796897
1.45917848	1.2488271	1.25527864	0.69684682	1.17515431	0.59886209	1.43279304
6.04853017	4.05321075	4.01689164	3.93513498	2.91690088	2.74317472	3.60245108
1.74160013	2.10328774	1.5642703	2.45945936	1.76273146	3.14885549	2.47668512
7.76659516	5.95931526	5.19492236	5.69774752	5.2042548	3.67044505	3.02933386
28.6893318	17.8560365	15.9903186	17.236711	9.90487204	10.9340626	13.8571556
2.35351369	3.57120731	3.84308382	3.34076563	5.68690746	6.22043845	7.51192924
10.0495034	16.2347522	12.0892989	12.5432427	22.9784637	16.2272307	15.0852639
5.46015175	4.16275699	6.29570516	12.5022518	24.3214972	10.1806555	15.5765072
18.5221527	28.5039307	24.9897009	28.6527016	46.3766254	32.3965071	30.2524017
15.4390498	24.5821755	23.5606144	18.9993236	37.5419832	26.6397038	24.7873196
2.63593533	1.11737161	1.39046249	1.25022518	1.3220486	0.65681648	1.0234236
8.80214118	3.17684086	3.76583591	3.17680168	2.47621801	4.03748955	2.53809053
18.8516446	17.0892129	13.9432489	14.5722967	10.8491925	12.4988313	12.1787409
23.911699	37.2676296	35.2829857	35.6006743	47.9295079	38.9839901	34.2232852
28.3363048	21.0109682	16.8014217	17.9745488	12.6119239	13.8704187	15.7607235
14.4270389	5.71831354	7.64754369	6.80450423	8.47789895	5.60225823	6.32475786
11.2027251	11.3928086	8.40071087	8.95653118	5.05736051	5.6408945	6.01773078
1.3415028	1.13928086	0.75316718	0.81981979	0.25181878	0.4829533	0.53218027
14.4741092	9.28952085	10.138789	8.95653118	7.47062383	7.01248186	5.87445147
43.681214	28.3943845	32.5213727	25.9268008	17.9420881	14.8556434	13.570597
0.91787034	2.62910968	2.2981255	1.5371621	2.45523311	2.80112912	2.00591026
1.74160013	2.12519699	3.03198071	2.78738728	4.82652663	4.7329423	4.35978454
6.84872482	4.75430666	3.28303643	5.18536015	4.44879846	2.91703791	4.85102787
395.955142	285.455583	274.152854	271.278368	122.845595	222.467606	234.896185
9.76708179	13.6494611	13.2673296	13.506531	13.4513199	16.4590483	9.41549714
31.3252672	46.2942395	42.6987856	44.9466198	53.9941436	59.345301	59.1948211
222.148157	155.402291	141.170567	149.965535	114.703455	124.60195	122.278652
1.43564335	0.15336473	0.32830364	0.53288286	0.33575837	0	0.38890097
25.276737	35.3396159	35.9975289	36.66644	52.3363366	48.0441939	46.5453054
1.45917848	0.28482021	0.52142343	0.73783781	0.62954695	0.30909011	1.0234236
14.0504767	11.7652658	9.98429315	10.3092338	4.17599478	7.57270768	11.2576596
5.22480038	2.12519699	2.58780519	3.4637386	2.81197638	2.56931153	2.68136984
3.10663806	4.88576215	3.84308382	4.48851333	5.09933031	4.42385219	6.2224155
21.2051583	33.7621501	33.4290357	33.1412149	32.7784113	45.9385175	50.5775944
14.6859254	10.3849832	10.4091567	9.65337799	14.3326856	8.34543295	9.35409172
10.0259683	16.6510279	13.769441	15.9045039	20.3133816	17.6567725	15.0238585

2.6830056	4.09702924	4.26794736	4.91891872	8.10017078	5.71816702	4.3188476
46.5525007	61.3020739	66.8966953	59.826349	87.5489961	83.666829	84.3096363
15.5567255	13.1893669	10.7567723	11.2725221	8.18411037	9.33065768	9.10847006
51.58902	86.2128881	76.1278213	76.1202672	72.9435068	87.2406834	94.3391876
9.55526556	11.8529028	11.3554437	12.6867112	18.6975445	14.353372	14.4302728
24.4765423	9.77152429	15.1019676	10.9240987	9.90487204	7.84316153	6.67272188
2.35351369	8.4788787	5.36873017	4.2425674	32.7364415	16.4783665	19.1789583
7321.68693	13466.1464	6177.63172	8062.02581	9723.81434	9249.94652	11874.3952
9.46112502	33.8716963	14.2136166	20.0855848	34.6040974	23.9738016	49.6155762
1.85927581	0.26291097	0.44417552	1.22972968	1.3430335	1.15908791	1.00295513
0.58837842	1.73083054	1.6608302	1.37319814	3.08478006	2.70453846	2.57902748
28.9246832	34.0688795	34.7808742	33.0592329	50.1119374	57.4907604	59.624659
5.86024908	3.83411828	3.84308382	3.29977464	1.69977677	1.85454066	3.33636094
1.0826163	2.47574494	2.00844582	2.21351343	2.47621801	4.13408021	3.99135205
14.3328983	7.40532559	6.91368849	7.29639611	5.37213399	5.37044065	4.60540621
4.33046518	2.97965763	0.94628697	1.66013507	0.27280368	1.25567857	1.06436055
2.00048663	3.00156688	1.9311979	2.80788277	2.68606699	2.16363077	2.74277525
19.8165852	27.9561995	25.3566285	26.7876115	51.6018651	32.1260532	27.9599328
3.24784889	7.53678107	4.30657132	5.7797295	12.6538937	5.83407582	8.35113659
36.3382513	21.6901548	24.1013498	20.3110352	18.7395143	18.6226791	17.3367958
61.3796369	94.0783079	79.0053062	87.7822037	159.904926	90.6020383	101.278
12.0264549	8.04069376	7.31924005	7.72680149	3.65137232	5.96930274	5.21946037
2.58886505	4.31612172	5.07905048	5.34932411	2.5811425	8.38406922	5.81304606
3.43612998	1.13928086	2.45262134	3.19729717	1.07022982	1.56476868	1.16670291
15.4155146	17.7683996	16.608302	19.3272515	36.5766779	22.9113044	25.5241846
5.46015175	13.6494611	12.842466	13.2400896	28.2456732	17.3670005	15.5560387
3.64794621	3.54929806	4.88593069	5.0009007	9.56911367	6.4329379	5.99726231
5.62489771	10.4288017	10.2160369	7.87026996	14.8573081	11.1851983	12.2810832
0.82372979	1.18309935	1.71876613	1.29121616	1.59485228	2.51135714	1.47372999
29.2777102	40.4444705	39.3191893	39.6177912	68.1379651	43.6203417	45.051107
0.65898383	2.12519699	0.8304151	0.7583333	2.72803679	1.02386099	1.26904527
8.09608708	9.11424687	7.70547963	8.71058524	16.0954171	14.4113264	11.4828128
1.03554602	1.68701204	1.31321457	1.5371621	4.63766254	1.87385879	1.3918561
94.9642772	289.136337	255.497482	276.197286	481.204705	303.352624	278.412157
2.49472451	4.22848473	4.01689164	3.83265751	6.08562053	5.08066867	6.16101008
3.17724348	4.00939225	5.83221766	5.24684664	15.7176889	11.9579236	10.9915695
0.14121082	1.97183226	0.79179114	1.06576572	2.6231123	3.32271868	3.37729789
115.510452	79.9687526	86.7107858	89.3808523	56.7431652	65.9521021	68.5079759
63.450729	159.061135	149.609901	152.076571	135.583429	189.047238	198.441836
0.91787034	1.44601032	1.54495832	1.18873869	2.24538413	2.16363077	2.29246887
2.6830056	3.74648129	4.38381924	3.58671157	2.39227842	4.63635164	3.86854121
0.91787034	2.91392989	3.03198071	3.21779266	0.92333553	1.71931373	3.09073928
3.20077861	1.64319355	1.9311979	1.90608101	2.85394618	1.42954176	0.90061277

7.90780598	5.12676387	4.19069945	3.58671157	1.65780697	3.0329467	4.07322594
0.87080006	3.22065935	1.89257394	3.42274761	5.2252397	2.93635604	2.90652303
0.94140547	2.8701114	1.6608302	2.60292782	1.46894289	2.22158516	2.4152797
6.58983832	4.29421247	4.15207549	3.68918904	1.2800788	2.14431263	2.14918956
0.35302705	1.53364731	0.52142343	0.55337836	1.82568616	1.04317912	0.90061277
3.10663806	1.88419527	2.56849321	1.20923419	1.74174657	0.77272527	0.67545958
23.4409963	17.0892129	18.4815639	18.3024767	13.6191991	11.3783797	9.82486658
991.700062	1479.8382	1323.83616	1531.27989	2419.70568	1380.58961	1444.50101
7.95487626	11.0422606	9.94566919	9.85833294	12.0453317	17.4635912	14.655426
17.0865094	9.85916128	7.62823171	11.0265761	9.82093245	7.03179999	4.62587468
0.70605411	0.35054796	1.48702238	1.35270265	2.39227842	2.10567637	2.25153192
16.4510607	10.7355312	10.8340202	12.3177923	5.45607358	9.4658846	7.18443368
5.03651929	6.96714064	9.07663014	8.19819787	7.00895606	8.84770438	8.80144298
11.1556549	7.60250881	9.26974993	8.0957204	7.68047281	6.29771098	5.69023523
1.24736225	3.6588443	3.05129268	2.88986475	1.76273146	4.61703351	2.53809053
32.2431375	50.6322705	51.9878475	48.9432413	83.016258	54.7475856	58.9491995
19.0163906	32.6447785	22.6722634	24.4716206	41.4032045	29.1703791	28.9014825
9.39051961	3.96557376	4.71212288	3.93513498	1.48992779	1.46817802	1.65794623
485.106241	219.968843	213.127	218.604946	136.905477	177.823404	164.423236
6.18974099	9.6838873	8.84488639	9.85833294	14.9412477	10.8761082	11.3395335
31.466478	19.5430486	18.7712436	18.3844587	18.6765596	13.9283731	15.1671378
6.11913558	12.9921836	11.5099395	11.8873869	9.35926468	11.7067879	13.6934078
0.18828109	1.27073634	0.67591927	0.28693693	0.41969797	0.67613461	1.47372999
7.06054106	35.2957974	18.44294	29.1855844	33.8906109	35.5646807	44.0072149
6.02499503	3.37402408	1.79601405	3.70968454	1.17515431	1.98976758	1.3918561
4.9188436	9.48670408	9.98429315	8.71058524	16.6620093	8.32611482	8.94472228
13.8151253	11.3928086	9.21181399	9.96081041	4.93145112	4.15339835	6.18147855
106.214073	35.3177066	42.5636018	37.7936922	22.3279319	19.1829049	14.2255881
2.84775156	4.92958064	4.28725934	4.32454938	7.49160873	3.68976318	4.95337023
6.707514	10.0782538	14.1943046	11.7029275	21.8452792	11.1851983	13.2840384
15.4155146	21.5367901	22.131528	23.1394135	43.0400266	25.210162	27.3049417
15.6273309	22.5665247	22.1894639	20.6389631	39.1578204	25.499934	22.4743823
147.8948	92.4132051	95.3818644	102.088059	43.0190417	56.7180351	58.7854517
246.012786	198.081505	202.833716	193.456974	68.9563761	123.867861	118.2873
14.8271362	11.1518069	10.3319088	9.12049513	5.2462246	10.2192917	8.67863214
30.3838617	15.6212933	16.2027504	17.2162155	10.5554039	10.7795176	13.918561
60.1558098	34.9452494	38.6046461	36.789413	27.1754434	27.1806115	28.1646175
42.0337544	31.3740421	31.7295815	30.4153141	25.5386213	22.1772154	24.4598241
16.1451039	10.9984421	12.3789786	13.342567	5.77084706	6.87725493	8.57628978
2.35351369	5.98122451	3.76583591	4.11959443	1.84667106	5.08066867	4.78962246
5.8131788	11.7652658	9.1152541	11.7029275	10.4714643	17.6374544	15.2080747
85.1030549	28.701114	28.1761774	25.3324314	14.7313987	10.9340626	9.49737102
1.69452985	0.72300516	2.20156561	0.84031528	0.79742614	0.59886209	0.38890097

6.0014599	3.39593333	5.1562984	3.0948197	3.8822062	5.23521373	5.21946037
11.1321197	5.93740602	5.25285829	6.12815291	3.9451609	2.9749923	0.79827041
168.417439	83.5180507	85.3010113	80.5472941	40.0811559	30.8510565	27.3254102
31.4194077	18.425677	21.2624889	21.3153145	13.1995011	11.4556522	12.0763985
3.5773408	6.74804817	7.10680828	6.29211687	14.5005648	19.2794956	21.6556434
1.05908116	2.4538357	3.82377185	3.40225212	3.71432702	2.83976538	3.47964025
3.7656219	6.46322795	7.68616765	7.99324293	9.25434019	7.03179999	6.91834355
10.7084873	16.2347522	16.4151822	17.4621615	31.3094684	27.8760642	29.3722574
12.3559468	5.58685806	3.99757966	4.34504487	2.74902169	2.53067527	1.51466693
1.92988122	4.4475772	3.78514789	4.95990971	8.20509527	4.61703351	4.44165843
15.4861201	29.2707544	29.6052638	29.6569808	81.7151943	45.571473	47.2412335
25.0178505	16.125206	16.6662379	14.4083328	9.52714387	16.7874566	17.0093003
13.6268442	40.838837	37.0983117	42.3641875	44.8447279	43.6010236	40.3024214
15.6037957	37.3114481	43.1622731	40.9295029	74.3075252	64.0782433	59.8498122
8.28436817	12.3568155	9.63667753	12.9941436	11.3738149	12.1704231	17.8075707
7.08407619	2.93583914	4.26794736	6.51756731	2.22439923	4.88748736	5.36273967
373.149595	260.785771	252.697245	245.351567	156.484387	181.146122	185.055456
0.4471676	0.43818495	0.23174375	0.43040539	1.84667106	1.15908791	0.98248666
2.65947046	1.9937415	1.85394999	1.31171166	1.15416941	1.21704231	1.30998221
2.54179478	6.81377591	7.04887234	5.14436916	3.08478006	4.98407801	3.72526191
68.8873456	50.5446335	48.9944908	51.4231962	32.9462905	37.805584	29.4131943
8.91981687	5.41158408	6.43088901	4.46801784	3.79826661	6.39430164	4.74868551
6.16620586	3.39593333	4.46106715	4.48851333	2.47621801	4.05680769	2.7837122
18.404477	28.1314735	33.8345872	27.1770259	38.3603943	26.5624313	28.8400771
44.6696898	28.1314735	28.9293446	26.3367107	24.5523311	18.4488159	19.4041115
9.72001152	4.4475772	5.61978589	4.81644125	4.15500988	4.63635164	4.23697371
1.83574067	1.75273978	1.60289426	0.71734231	1.46894289	0.59886209	0.79827041
3.41259484	0.92018839	2.20156561	1.74211705	1.67879187	2.14431263	1.35091915
10.3789954	7.18623311	6.9909364	7.00945918	8.56183854	4.05680769	5.07618106
5.24833552	7.88732903	7.87928744	8.58761227	9.65305326	13.0204209	10.3570469
15.2036984	20.6823294	20.5672577	18.0155398	19.7677743	30.3874214	29.8635007
17.1100445	11.8090843	11.5485635	12.8506752	9.1704006	11.3397434	9.12893853
1.50624876	4.35994021	2.83886092	2.09054046	2.87493108	3.11021923	4.89196482
20.6167799	25.1299066	27.3843862	26.6441431	50.1748921	34.5601379	37.3549615
4.471676	10.6478942	9.3856218	11.1290536	17.1026922	16.4783665	15.4946333
1.48271362	2.91392989	2.52986925	3.23828816	6.71516749	3.01362857	5.26039731
82.0905573	50.0626301	56.6613464	52.0585565	41.1933555	44.4317032	43.4340977
4.30693004	2.65101892	3.18647654	2.60292782	1.57386738	1.85454066	1.65794623
1.92988122	2.36619871	2.68436508	2.62342332	4.15500988	3.22612802	4.91243329
28.3127696	19.1486821	20.0265222	18.9378371	17.8581485	14.8749615	15.8425974
3.36552457	2.76056516	1.77670207	1.72162155	0.86038083	2.10567637	1.22810832
3.67148135	5.49922107	6.00602547	5.32882862	7.38668423	4.75226043	4.44165843
17.0629742	25.5680916	24.6807092	25.7628368	35.527433	35.0817274	37.4368354

4.73056251	2.84820215	2.3946854	3.54572058	1.97258045	2.26022143	2.61996442
47.9410738	34.9890679	28.1568654	30.784233	27.6580961	27.3931109	23.804833
59.2144043	93.4867582	86.3052342	86.79842	134.953882	95.6054345	98.7194406
5.22480038	10.8231682	7.58960775	8.36216183	10.2406304	10.9533808	10.2547045
31.1605212	18.1408568	20.0458342	18.5894137	16.2842812	13.4261016	11.9126507
29.23064	17.7026718	15.5268311	18.9583326	21.7823245	14.1408725	15.1876063
2.80068129	4.62285118	4.01689164	3.66869355	4.97342092	4.32726153	2.80418067
6.21327613	2.47574494	3.2251005	1.68063056	1.3640184	1.37158736	0.90061277
13.9092659	6.39750021	7.66685567	6.49707181	2.70705189	3.86362637	4.23697371
58.0376475	31.6588623	35.6499133	37.3222958	40.9205519	44.7214752	39.7293042
64.2509236	43.9499501	50.7132569	42.1387371	41.508129	34.0771846	33.0156454
19.9107258	36.4788967	35.3409216	32.5058546	29.9874198	49.2612362	55.5309646
4.84823819	7.05477763	6.75919266	8.28017985	8.68774793	8.92497691	9.98861435
1.92988122	0.61345892	1.08147083	0.7788288	0.75545634	0.79204341	1.55560387
194.612047	276.538519	294.140752	284.64143	509.618257	395.32625	399.155673
2.65947046	4.18466623	3.18647654	3.52522508	7.05092586	4.77157856	3.54104566
84.608817	57.8404129	54.7494605	55.8297275	41.7389629	41.8623917	42.6358273
2.65947046	3.85602752	4.38381924	4.38603586	4.49076826	5.83407582	2.80418067
80.8431951	66.4726563	54.0928532	56.5675653	26.9446095	59.4418917	59.5837221
0.51777301	1.55555656	1.62220624	0.86081078	2.95887067	2.37613022	2.16965804
8.47264927	6.00313376	5.9867135	5.34932411	6.08562053	5.31248626	5.50601898
0	0.52582194	0.3669276	0.40990989	2.70705189	1.71931373	1.47372999
0.30595678	1.94992301	1.69945415	1.78310804	0.73447144	1.13976978	1.78075707
92.3754122	55.1017569	65.2551771	62.8391867	39.1997902	59.7509818	52.9928741
604.005752	858.645311	913.051056	867.000416	906.274807	1041.36321	978.966081
0.28242164	1.11737161	1.12009478	0.92229726	0.16787919	1.19772417	1.71935165
18.8045743	10.0782538	11.2009478	12.0923419	6.10660543	10.1227011	10.7254793
47.1408791	33.2144189	35.9782169	34.3914401	24.1746029	27.7408373	26.4247974
159.615298	107.793497	100.016739	98.8292753	84.5271707	81.3100169	72.0899585
26.1710722	16.5414817	17.6125249	14.8182427	8.58282344	6.95452746	7.12302827
125.771771	101.549361	92.02158	100.03851	53.1547476	72.9452658	69.4904626
165.687363	113.884267	119.850142	117.439185	86.9194491	104.607684	98.3919451
16.4275255	12.9702744	11.6451233	8.91554019	11.2479055	6.47157417	6.89787508
167.123007	124.028249	127.362502	125.698869	87.9686941	94.948618	89.8156553
102.683802	75.7402679	74.4476791	77.2885104	44.5719242	64.0782433	67.2593991
7.97841139	13.6275518	12.0699869	11.3135131	19.1382273	12.6533764	12.8746689
4.98944901	9.07042838	7.35786401	7.50135105	9.96782674	7.47611702	8.04410951
2.00048663	3.74648129	1.5642703	3.13581069	3.02182537	3.11021923	3.66385649
52.45982	39.3928266	92.1181399	72.6155377	124.209614	119.888326	113.907047
34.3848349	19.6745041	24.2751576	22.6680171	20.8170192	18.2749527	16.2929037
0.40009733	1.64319355	1.48702238	1.06576572	0.71348655	1.71931373	0.79827041
0.40009733	0.43818495	0.69523124	0.61486484	2.18242943	1.60340494	1.3918561
10.3083899	6.98904989	5.02111454	5.61576554	2.93788577	3.12953736	3.19308164

44.8109006	50.588452	54.0156053	52.4274754	90.86461	91.6065812	87.6869342
5.50722202	3.11111312	3.32166039	2.64391881	3.25265925	0.81136154	1.78075707
13.7680551	6.83568516	4.09413955	4.81644125	1.82568616	2.24090329	1.04389207
10.8732332	12.1596322	13.1900817	10.0427924	30.7428761	18.1010895	21.5533011
8.61386009	10.2973462	11.8961791	8.62860326	20.9429286	14.353372	17.7461653
0.61191356	1.6212843	0.84972708	1.47567562	3.37856864	1.39090549	1.86263096
0.32949192	0.92018839	0.48279948	0.34842341	1.02826002	1.17840604	0.90061277
0.80019465	2.25665247	1.06215885	0.18445945	4.19697968	2.37613022	3.9504151
11.7675684	8.25978623	10.5250286	7.23490962	5.35114909	6.35566538	5.42414509
48.8824792	30.4976722	32.9076122	30.0668907	10.534419	12.8079214	12.9156059
11.9558495	6.28795397	6.83644057	7.29639611	4.72160214	3.57385439	3.56151413
9.08456283	15.9280228	15.4882072	16.2939183	18.6345898	20.7669917	17.7256968
21.8876773	11.5023548	11.7996192	11.5799545	13.1575313	13.9863275	11.4009389
0.77665952	2.25665247	1.33252655	1.41418913	1.3220486	2.85908351	1.14623443
72.6294323	46.8200615	44.3596158	43.5324307	36.6396326	34.9465005	31.7875371
20.0048663	4.8638529	5.58116194	5.45180158	1.78371636	1.31363297	1.55560387
6.04853017	3.02347613	3.84308382	3.81216201	1.57386738	1.98976758	2.47668512
84.7264927	63.0109952	67.224999	62.5932408	46.8592781	43.9101137	43.4136292
1.48271362	2.60720043	2.47193331	2.74639629	0.90235063	1.83522252	3.13167622
7.95487626	5.17058236	5.17561038	4.26306289	5.162285	4.63635164	3.4387033
14.8271362	9.26761161	8.26552702	9.65337799	4.91046622	4.61703351	3.62291955
41.4924463	29.9499411	31.2081581	31.3581069	16.9977677	16.4976846	19.3222376
2.6830056	4.27230322	4.01689164	4.32454938	2.85394618	4.86816922	5.75164064
29.4424562	18.6447694	16.1061905	16.3349093	11.4157847	13.9283731	14.7168314
128.996085	96.9046008	92.987179	95.4270232	48.6220096	82.6043317	76.3269322
6.73104914	4.25039398	5.00180257	4.14008993	4.21796458	4.34657966	3.56151413
19.6518393	28.8325694	27.9251217	28.0378367	20.6911098	36.9942225	38.1532319
10.4731359	6.26604473	5.38804215	6.37409885	6.4633487	3.67044505	3.31589247
10.4731359	6.26604473	5.38804215	6.37409885	6.4633487	3.67044505	3.31589247
26.1004668	17.0453944	16.4344941	15.0231976	10.744268	14.5851895	13.0588852
84.3263954	61.2144369	61.2769094	60.5846823	51.8746688	54.4771318	40.281953
8.11962221	6.22222623	5.02111454	4.57049531	1.84667106	2.95567417	2.9269915
7.62538434	6.02504301	4.73143486	4.77545026	2.68606699	4.88748736	4.91243329
26.0769316	20.3756	21.5135446	22.2376117	16.1373869	15.0488247	14.614489
13.7209848	22.2597953	24.6613972	19.0813055	36.8284967	26.8908395	27.7757166
14.1681524	10.8669867	11.4906275	12.3997743	5.45607358	8.1908879	8.7809745
7.3429627	15.5336563	11.2781957	11.4569815	39.3256996	25.345389	26.9569777
19.8871906	11.0641699	8.76763847	7.76779248	17.0607224	13.5999648	14.0004349
0.68251897	1.66510279	1.98913384	2.56193684	3.84023641	1.02386099	2.2720004
6.04853017	2.16901548	2.54918123	3.29977464	2.41326331	2.6079478	2.00591026
75.2653677	36.3036228	40.7868997	41.4008993	34.7509917	43.5430692	46.4224946
3.17724348	6.63850193	5.36873017	5.0213962	6.21152992	7.24429944	8.41254201
636.390101	893.700106	883.81272	859.417083	1823.25191	1381.01461	1406.79808

26.6417749	46.688606	44.7265434	43.5119352	88.9339994	57.9157593	57.9871813
9.53173043	7.49296257	7.5509838	6.04617093	2.68606699	6.10452966	4.66681162
163.757482	93.4867582	94.9956248	90.364636	55.1692979	53.5305433	58.6626409
156.41452	103.937469	100.982338	99.977023	66.6480373	83.9952372	85.988051
7.97841139	14.4162847	15.642703	15.412612	13.9129876	20.3999472	18.2783455
6.54276805	3.87793677	2.99335675	4.11959443	3.67235722	3.30340054	3.41823483
34.0318079	48.1565256	45.074159	44.3112595	69.9006965	49.6089626	52.6449101
153.001925	97.8028799	96.4440232	92.639636	81.0436776	83.7054653	74.4028959
19.1811365	10.7574404	10.3319088	10.3707203	6.27448462	8.09429724	8.41254201
9.67294125	5.60876731	4.90524267	4.87792773	5.68690746	4.52044285	5.48555051
39.7037759	53.1737432	58.7084162	58.2686914	73.3632048	62.3589296	71.4759043
0.54130815	0.46009419	1.73807811	1.12725221	3.81925151	1.31363297	1.5351354
492.966977	339.374241	326.160014	316.983321	145.362391	234.908483	237.10678
22.0994935	29.7527578	25.9166758	23.2009	55.7988448	33.2465049	33.0770508
39.9155921	21.9530658	19.6402827	20.7414406	15.3819305	14.1022362	13.3045068
2.235838	1.00782538	0.92697499	1.20923419	0.12590939	0.8499978	0.45030638
1.29443253	0.81064215	0.4634875	0.61486484	0.86038083	0.4829533	0.38890097
27.3948993	16.6729372	17.5352769	19.6141884	14.9832175	17.2704099	17.7871022
8.6844655	6.87950365	5.38804215	6.29211687	1.40598819	4.88748736	3.295424
4.44814087	2.93583914	3.39890831	2.91036024	1.51091268	3.34203681	2.72230678
26.830056	33.3896929	35.3216096	34.760359	57.1208934	55.9646279	58.0690552
264.370192	167.868653	150.401693	158.922066	170.103586	129.798528	134.08896
1.03554602	0.43818495	0.4634875	0.3689189	0.08393959	0.09659066	0
13.3914929	9.37715784	8.6517666	9.36644107	4.49076826	7.41816263	7.18443368
168.158553	70.0876821	73.0765286	74.3986457	29.9034802	29.9044681	27.6529057
29.8425535	40.0501041	35.4181695	35.8056292	58.5688514	63.4021087	59.5837221
3.45966512	7.53678107	7.57029578	7.97274743	9.71600796	5.31248626	6.28382092
12.379482	7.38341634	9.03800618	8.50563029	6.5472883	10.4897456	8.04410951
88.1626227	60.4695225	61.8369568	58.8425652	41.9488119	43.3305697	42.8814489
21.0168772	32.0532288	29.1417763	32.6288275	56.9739991	33.8840032	38.1327634
2.02402177	4.60094193	3.1285406	2.88986475	2.5811425	3.57385439	2.25153192
60.367626	29.4241191	31.8068294	27.6074313	18.7604992	17.1351829	13.2226329
8.84921146	21.5148808	18.404316	20.3110352	16.8718583	27.6635648	25.4627792
20.8756664	12.1596322	12.0892989	12.0513509	2.16144453	10.0454286	12.1582724
15.815612	11.3928086	11.7223713	11.6209455	8.66676304	11.5908791	11.4418759
28.1244885	17.2425776	15.681327	16.6628372	17.6692844	11.1658802	9.12893853
5.57782743	3.50547957	4.21001143	3.56621607	3.50447803	1.87385879	2.66090136
75.2889028	55.4742141	60.5044303	60.4822048	31.4353778	53.2987257	48.9401166
1.41210821	5.08294537	4.59625101	3.97612597	8.35198956	9.40793021	7.88036173
1.60038931	3.87793677	2.60711717	1.90608101	3.23167435	3.14885549	2.88605456
0.02353514	0.24100172	0.40555156	0.28693693	1.84667106	1.50681428	1.59654082
16.5687363	31.9217733	29.6825118	33.3666653	22.0341433	29.595378	41.6533406
13.5327037	20.0031428	19.3892269	20.0036028	23.3142221	21.4817626	21.5533011

9.24930878	15.949932	14.0398087	13.1171166	27.049534	17.3476824	17.8075707
62.0150856	40.8607462	36.5382643	37.1993228	21.0898229	29.6533324	31.5828524
9.24930878	11.4366271	13.5956332	11.2520266	17.2495865	12.8658758	15.5355703
79.5016923	57.05168	59.867135	57.3668896	43.9004074	41.1089846	44.2937735
5.5542923	10.3192555	10.1774129	8.81306271	10.5973737	11.436334	11.6260921
62.5799289	85.3803367	84.8954598	80.875222	128.847276	85.3281883	99.9680174
0	0	0.42486354	0	0	8.1329335	2.43574817
1.36503794	0.96400688	0.69523124	0.51238737	1.48992779	0.69545275	0.42983791
36.6206729	29.1392989	26.5732831	27.5254494	24.1326331	18.9510873	19.8953548
0.91787034	1.42410107	1.98913384	1.49617111	1.90962575	3.18749175	2.7837122
68.0165455	89.214455	93.9527779	80.7727445	187.122339	88.7668158	105.55591
0.35302705	0.65727742	1.29390259	1.10675671	0.94432043	0.75340714	1.24857679
2.54179478	2.14710623	1.96982186	1.57815309	0.75545634	1.52613242	0.83920735
2.47118937	1.57746581	1.64151822	2.25450441	1.74174657	0.92727033	1.26904527
1.50624876	4.22848473	3.26372445	3.70968454	5.79183196	5.44771318	6.67272188
6.49569777	5.19249161	6.2570812	6.21013489	4.19697968	4.249989	3.82760427
25.7239046	38.6479122	39.8213007	39.2488723	44.2571507	36.2794516	35.0215557
13.3679577	9.3114301	8.80626243	8.50563029	5.70789236	6.37498351	8.08504645
18.5456878	13.0798206	13.9625608	13.4040535	11.1849508	11.4556522	8.14645187
4.98944901	3.13302236	3.18647654	3.11531519	1.51091268	2.62726593	3.54104566
0.11767568	0.72300516	0.67591927	0.7788288	0.79742614	2.16363077	2.558559
2.4241191	5.19249161	4.13276351	2.93085574	3.77728171	3.84430824	4.56446926
1.31796766	0.92018839	0.27036771	0.30743242	0.73447144	0.38636264	0.71639652
30.3603265	17.987492	19.350603	20.5159902	19.1801971	18.468134	14.8191738
0.07060541	0.26291097	0.56004739	0.16396396	0.23083388	1.83522252	1.06436055
3.34198943	2.4100172	2.43330936	2.64391881	2.6231123	1.41022362	1.92403637
7.22528701	5.49922107	5.92877756	5.14436916	1.88864086	4.19203461	3.00886539
35.7498729	66.4726563	66.2207761	66.3849073	109.310336	100.241786	103.836559
545.07377	274.259958	268.687564	272.323638	136.40184	125.915583	116.056236
136.574399	116.447649	99.5339399	106.617563	43.2498756	74.4907164	67.6483001
916.128737	473.831291	496.259925	485.538269	348.370298	357.037713	331.609715
0.42363246	1.05164387	1.54495832	1.18873869	0.58757715	0.88863406	1.08482902
1.22382712	0.76682366	0.65660729	1.1682432	0.29378858	1.04317912	0.53218027
26.5947046	9.92488902	11.3361317	11.6619365	5.41410378	10.6829269	10.7050109
0.68251897	0.89827914	0.84972708	0.53288286	2.6440972	1.31363297	0.90061277
89.5276606	60.0751561	65.1199933	63.392565	22.9574788	36.3953604	34.734997
2.04755691	1.18309935	2.35606144	1.27072067	33.7856864	2.04772197	3.13167622
46.1759385	24.5821755	26.5925951	25.2094585	8.37297446	16.9806379	18.7900573
24.1235153	18.0532198	17.5932129	16.2939183	8.62479324	14.7204165	13.1202906
5.48368689	3.46166107	2.43330936	2.74639629	1.80470126	1.83522252	2.23106345
29.9837644	88.1189926	79.5074176	75.9153123	109.352306	104.704275	100.438792
0.87080006	1.6212843	1.27459062	1.1682432	1.59485228	1.23636044	2.35387428
27.0183371	38.9765509	39.4157492	36.1745481	64.8013662	41.901028	44.3961158

153.825654	203.624544	203.393763	205.180397	354.497889	213.581266	231.170923
280.209339	187.389792	188.079364	187.636254	123.517112	133.488291	125.185175
97.6002125	60.6228873	55.1357001	55.0099077	65.3259887	46.8464697	42.8200435
15.3684444	23.9029888	27.1912665	24.5945936	44.4250299	30.1169675	25.2171575
1.85927581	4.0313015	4.05551559	4.20157641	3.37856864	4.40453406	4.11416288
13.7209848	8.65415268	10.8147083	11.9078824	4.25993437	7.61134394	8.41254201
0.30595678	0.61345892	0.57935937	0.40990989	0.73447144	1.75795	1.10529749
20.9227367	12.5978172	12.0506749	10.4936933	10.5554039	9.85224724	9.35409172
8.0490168	10.6040757	9.36630982	9.32545008	10.9121472	14.7204165	12.7518581
10.4025305	25.962458	23.000567	17.318693	5.87577155	26.7749307	22.1059498
7.46063838	5.74022279	5.00180257	6.1691439	4.21796458	2.95567417	3.97088357
12.4265523	6.17840774	7.57029578	7.39887358	9.21237039	4.09544395	5.05571259
253.049791	185.001684	181.300859	174.847065	127.96591	160.552994	176.458697
4.77763278	3.28638709	2.33674946	2.50045035	2.89591598	1.58408681	1.92403637
6.49569777	4.66666967	4.55762705	4.50900883	2.85394618	3.74771758	2.90652303
112.61563	89.389729	93.1223628	81.5515733	60.7302959	44.3157944	35.2876458
1.74160013	3.00156688	2.54918123	3.29977464	6.00168094	4.05680769	4.89196482
11.861709	13.6275518	15.0826556	15.9659904	25.6435458	18.1397258	17.561949
2.73007588	2.80438365	3.5920281	3.83265751	3.42053844	4.3658978	3.60245108
26.3358181	38.5602752	35.1671138	37.0968454	54.3508868	41.8430736	42.3288002
271.713155	457.311719	459.837532	474.491197	339.262852	585.629167	600.565438
10.8967684	7.71205505	8.63245462	9.87882844	5.2462246	5.6408945	6.03819925
11.1556549	6.04695225	5.85152964	5.90270247	5.93872624	2.70453846	2.94745997
27.3713642	37.8372701	36.5962002	34.9448184	73.1323709	40.2783049	42.5744218
0.30595678	0.21909247	0.63729531	1.12725221	0.50363756	1.2749967	0.73686499
17.204185	9.39906709	11.1043879	12.3792788	13.3254105	8.84770438	9.94767741
2.58886505	4.33803096	4.6928109	4.32454938	10.0727512	5.52498571	5.07618106
0.42363246	0.43818495	0.7338552	0.86081078	1.97258045	1.58408681	0.55264874
3.69501649	7.3176886	6.14120933	5.73873851	9.1494157	7.41816263	8.06457798
0.77665952	2.25665247	1.98913384	0.98378374	2.6650821	3.34203681	1.35091915
12.6619036	8.15023999	8.7483265	9.89932393	8.43592915	5.89203021	7.90083021
2.98896238	1.02973462	0.44417552	1.57815309	0.90235063	0.17386319	0.34796402
2.82421642	1.53364731	1.25527864	1.82409903	1.65780697	1.23636044	2.23106345
222.054016	159.937505	157.334693	156.913507	89.3956671	112.837208	120.948201
8.37850872	3.6588443	3.82377185	3.42274761	0.96530533	2.27953956	2.10825262
57.5434096	36.237895	36.6348242	41.2779263	25.5176364	31.6624181	28.53305
19.2517419	12.400634	14.3294884	14.5108102	8.83464222	9.48520273	9.78392963
171.782964	117.521202	118.961791	122.132653	70.9079717	75.0702603	76.101779
647.122123	468.6388	465.882182	459.078585	224.244624	359.626342	358.935126
133.208875	58.2566886	65.6607287	62.2448173	38.7381224	37.3226307	39.7293042
26.2887479	17.1549406	16.7627978	15.9249994	15.1301117	10.0067923	10.4389207
138.386605	206.275563	211.65929	210.960127	204.665714	222.255107	222.308075
4.35400032	2.67292817	2.43330936	2.80788277	1.74174657	2.08635824	2.14918956

20.4520339	9.99061677	10.100165	10.9650897	8.77168753	7.78520713	7.71661396
103.13097	150.341255	156.407718	154.105624	322.558873	206.259694	174.350445
39.6331705	27.4084684	27.654754	26.5416656	13.1365464	16.845411	14.9829215
7.31942756	3.48357032	5.17561038	3.97612597	3.8612213	2.10567637	1.73982012
350.979496	242.535368	238.947116	253.201341	125.678556	152.42006	150.770765
1.03554602	3.17684086	2.31743748	2.17252244	2.24538413	2.08635824	1.71935165
29.8425535	45.3521419	47.9323319	47.7749981	74.5803289	58.32144	55.8993971
41.6571922	27.3646499	27.6740659	26.7671161	38.9060016	18.313589	19.8544179
6.02499503	3.33020559	2.70367706	3.68918904	0.90235063	1.95113132	1.22810832
4.89530847	2.84820215	3.24441248	2.64391881	2.77000659	3.16817362	3.50010872
114.427835	66.450747	71.5701942	68.8853576	37.7308473	44.5669302	36.4952856
165.193126	79.8372971	84.70234	82.9247715	35.4434934	47.7351038	51.5600811
12.1676658	8.50078795	10.0422291	8.4646393	5.62395277	6.64543735	6.65225341
3.5773408	5.32394709	5.33010621	4.65247729	5.2462246	6.74202801	5.40367662
52.6010309	36.4788967	42.1773622	35.6006743	25.2238479	30.9476472	25.3604369
498.309453	263.787337	252.05995	251.541206	126.874696	150.430293	158.3441
28.4304453	8.8951544	8.98007024	7.89076545	4.65864744	4.96475988	6.44756869
39.4448894	78.0188296	78.8508103	79.2355824	168.907447	115.619019	109.854289
8.09608708	21.3396069	22.6336394	24.2666657	19.7887592	32.4351434	30.4366179
3.97743813	9.28952085	8.53589473	8.15720688	16.2423114	9.40793021	7.77801937
0.30595678	0.94209763	0.84972708	0.96328825	1.2590939	1.21704231	2.64043289
232.079985	179.480554	176.067313	185.01283	112.688904	127.770124	130.588852
2.8712867	1.86228602	1.25527864	1.96756749	1.3010637	0.96590659	0.40936944
11.2968657	5.21440086	6.73988068	5.51328807	6.35842421	8.1329335	10.1318937
81.0785465	103.696467	106.215885	106.105176	183.093238	132.406476	136.299555
20.7344556	16.388117	13.9046249	15.0027021	9.44320428	12.0351961	10.7664163
4.14218409	3.17684086	2.761613	3.62770256	1.86765596	3.01362857	1.92403637
4.11864895	6.17840774	6.68194474	6.47657632	10.0097965	7.32157197	8.22832576
9.27284392	13.1674576	12.7459062	13.424549	23.6289956	15.8601862	15.7607235
22.0288881	19.4115931	18.6167478	17.1137381	10.0517663	17.1931373	12.5471734
34.573116	67.4585724	64.3088901	62.4907633	89.8153651	70.3566362	77.8825361
84.6794224	34.9014309	41.6752507	41.7903137	17.144662	23.9544835	22.5971931
1.29443253	1.16119011	0.38623958	0.3894144	0.35674327	0.38636264	1.14623443
2.65947046	6.17840774	6.66263276	4.85743224	5.09933031	5.83407582	4.60540621
11.8852441	14.7887419	16.531054	17.236711	17.6273146	16.9613198	16.9683633
18.92225	28.8325694	30.358431	28.1813052	27.1124887	34.6953648	32.2378435
6.04853017	9.26761161	7.78272754	8.01373842	8.35198956	12.0545143	9.55877644
0	0.65727742	0.34761562	0.84031528	2.85394618	2.27953956	0
0.16474596	0.19718323	0.27036771	0.06148648	1.23810901	1.21704231	0.59358569
0.04707027	0	0.38623958	0	0	1.83522252	3.84807274
0	0.7887329	0.38623958	1.35270265	5.09933031	2.00908571	0
0	1.29264559	0.21243177	1.74211705	1.53189758	2.85908351	1.18717138
0.40009733	1.05164387	1.69945415	0.7788288	2.07750494	3.74771758	2.43574817

0	0	0.48279948	1.1682432	0.37772817	2.1829489	1.88309943
7.29589242	9.6838873	10.3898447	9.46891854	11.7515431	11.2045165	12.1378039
2.00048663	3.39593333	3.90101976	3.97612597	6.4843336	6.99316373	4.11416288
214.381562	160.244235	165.928524	152.302021	110.149732	100.550876	95.874323
19.5812339	28.3067475	25.1055727	26.5006746	47.8665532	33.5942313	36.9660605
1.60038931	1.31455484	2.64574113	2.78738728	3.04281027	2.56931153	3.23401858
9.50819529	13.0798206	16.0675665	14.9822066	25.1818781	19.5499494	16.0677505
2.28290827	1.18309935	1.40977447	1.82409903	0.54560736	1.10113351	0.71639652
0.28242164	1.66510279	0.54073541	1.00427924	1.55288248	1.89317692	1.51466693
60.532372	44.2347703	40.0337325	41.4828812	16.5990546	21.2113088	23.6820221
1.9769515	2.49765419	3.53409216	2.52094585	4.40682866	2.1249945	2.68136984
4.75409764	7.33959784	7.41579994	7.82927897	12.6538937	8.07497911	5.69023523
9.08456283	23.7277148	21.4749207	20.4135127	16.0324624	22.563578	18.6672465
219.818178	173.959424	173.981619	172.613056	108.785713	121.994003	128.828563
5.27187066	6.28795397	8.34277494	6.96846819	8.18411037	9.91020163	8.31019965
11.2733306	16.9358482	15.5847671	14.3878373	19.2851216	20.9408549	21.3895533
28.7834724	36.3474413	36.9824398	38.1011246	62.8078009	44.6442027	42.06271
68.7696699	98.0877001	99.7077477	96.287834	143.368826	104.665638	118.082615
1.27089739	2.23474322	1.35183853	2.39797288	3.00084047	3.26476428	2.35387428
52.0597227	79.1581105	76.8230525	81.8590058	72.4188843	82.93274	90.1226824
1347.24537	895.452846	931.281564	943.428116	1004.46315	819.533107	702.559834
19.3694176	24.9546327	25.7042441	24.943017	40.7736576	29.1510609	31.5623839
6.56630318	3.30829634	5.21423434	4.93941422	5.14130011	2.37613022	4.38025302
31.2546617	40.9702924	45.2672788	41.2779263	75.5036644	46.4987433	47.95763
63.6625452	116.42574	94.6286972	92.3731945	169.767828	109.108809	119.167444
40.7393219	24.7574494	26.0904837	24.0822062	15.9485228	21.3658538	15.3104171
6.21327613	9.70579655	7.87928744	8.42364831	8.18411037	11.0886077	10.0295513
120.146874	83.2332305	84.7988999	79.2355824	54.3928566	56.8725801	55.7561178
9.29637906	5.12676387	6.08327339	3.32027014	4.34387397	2.87840164	3.33636094
111.721295	59.4178787	62.1652605	59.2114841	58.442942	34.3862747	35.3285827
0.56484328	1.86228602	1.1973427	0.96328825	1.59485228	1.39090549	0.59358569
113.910062	280.00018	297.501037	308.02679	867.872443	790.111592	679.655614
0.94140547	0.92018839	2.25950155	2.23400892	3.46250823	3.05226483	2.25153192
31.3723374	22.7417987	22.131528	18.8968461	12.3601052	14.5851895	15.6583811
139.798713	107.859224	112.202598	104.854951	67.1097051	70.0282279	77.3708243
0.28242164	0.76682366	0.54073541	0.57387385	2.77000659	2.00908571	0.61405416
0.87080006	0.54773118	0.27036771	0.69684682	0.20984898	0.32840824	0.34796402
2.61240019	0.72300516	0.23174375	0.67635132	0.60856205	0.11590879	0.8801443
8.61386009	3.92175527	4.44175517	6.37409885	2.43424821	2.45340274	2.68136984
3.50673539	6.09077075	3.99757966	6.72252226	9.04449121	5.38975878	8.80144298
9.03749255	5.34585634	7.47373588	6.1486484	4.74258704	5.73748516	5.36273967
16.0274282	11.6776288	11.4906275	11.559459	8.22608017	10.0647467	7.88036173
284.186778	216.682456	207.893454	209.013055	106.435405	160.668903	152.817612

0.94140547	2.76056516	1.89257394	1.37319814	4.02910049	2.16363077	1.45326151
52.5539606	34.6823385	36.3644565	33.9405392	31.0366647	34.8305917	26.9365092
342.036144	770.635864	815.50625	716.829926	896.831603	773.459363	760.280925
3783.13204	2514.56813	2545.41539	2456.24157	2609.99674	2129.74676	2042.32367
0.84726493	0.08763699	0.30899166	0.3894144	0	0.15454505	0
21.8876773	12.4444525	14.3681124	13.342567	7.19782015	10.9147445	10.9915695
33.3257538	51.2238202	51.4084881	52.2225204	54.3508868	58.5918939	59.317632
6.40155722	3.17684086	4.13276351	4.42702685	4.11304008	2.83976538	2.98839692
6.63690859	3.6588443	4.22932341	4.40653136	5.77084706	3.47726373	4.29837913
4.82470306	3.76839053	2.16294165	2.39797288	2.53917271	1.95113132	3.54104566
0.54130815	1.51173806	0.7338552	0.43040539	1.72076167	0.81136154	0.83920735
0.82372979	1.18309935	1.85394999	0.92229726	2.85394618	1.62272307	1.35091915
1.85927581	1.18309935	0.90766301	1.1682432	0.54560736	0.65681648	0.98248666
22.2171692	12.4444525	15.2950874	14.7362607	5.05736051	11.9192873	11.6874975
5.03651929	10.2535277	6.41157703	7.56283754	10.1986606	9.56247526	7.71661396
1.15322171	1.48982882	2.18225363	1.25022518	1.76273146	2.66590219	3.66385649
0.32949192	0.30672946	1.39046249	0.43040539	1.97258045	1.89317692	1.51466693
22.4525206	13.8028258	15.4302712	15.3101345	14.248746	9.65906592	12.6085788
229.53819	164.976632	172.262853	169.087831	85.5134609	127.151944	124.693932
62.085691	40.4663798	44.6492955	41.2164398	29.2949182	36.7430868	32.6472129
1.15322171	0.30672946	0.34761562	0.47139638	0.08393959	0.52158956	0.47077486
144.999978	119.90931	116.277426	120.800446	54.4348264	80.150929	72.5402649
3.15370834	7.40532559	5.21423434	5.16486466	7.21880505	8.57725054	14.1846511
18.2632662	28.6353862	29.431456	28.1813052	28.7912806	29.749923	30.6617711
13.9092659	23.0485282	20.3548259	22.2581072	18.8654237	24.1090285	28.0008697
65.9219183	48.2660718	48.3765074	45.0900883	27.4062773	38.8873994	36.9046551
637.684533	452.119227	452.421732	452.069126	263.486384	320.198035	326.656345
4.61288682	6.55086494	7.4544239	8.23918886	9.79994755	8.82838625	8.49441589
78.0895841	119.449216	125.431304	124.120716	110.653369	127.461034	138.100781
15.4625849	14.3286477	11.2781957	11.6209455	3.65137232	8.22952416	9.80439811
2.44765423	3.98748301	3.10922862	2.56193684	3.63038742	4.17271648	3.54104566
57.260988	78.5446515	79.9515931	69.2132855	112.311176	90.0804488	84.9236905
34.6201863	23.3990761	24.9317649	25.7218458	18.4457257	20.7476736	20.8164361
8.63739523	12.5320895	12.5914103	14.1009003	13.8920027	12.3636044	13.3863807
14.8742065	10.100163	10.0229171	8.54662128	8.54085365	7.20566318	7.73708243
1.36503794	2.69483742	3.24441248	2.93085574	1.80470126	6.25907472	5.11711801
9.69647638	7.60250881	7.33855203	7.3373871	4.19697968	3.97953516	3.21355011
0.56484328	1.73083054	0.65660729	0.88130627	1.63682207	0.75340714	0.53218027
9.08456283	12.5101802	11.2781957	13.1376121	15.2140513	14.7010983	14.7577683
167.570174	95.4585905	97.3130623	96.8412124	52.3573215	54.6316768	54.4461356
4.70702737	3.59311656	2.10500571	2.72590079	1.97258045	2.37613022	3.52057719
13.3444226	14.3286477	16.0482546	15.8225219	26.5458965	24.3022099	22.3106345
15.7685417	21.8216103	20.6831295	18.5894137	28.5814316	23.7033478	23.3135896

5.03651929	7.16432387	5.83221766	7.66531501	4.84751153	9.83292911	10.9506325
12.3088766	8.45696946	7.70547963	7.78828798	3.8822062	8.15225164	4.72821704
36.5029973	65.7934696	62.6480599	65.667565	51.014288	72.7714026	69.2448409
151.519211	109.896784	107.509787	106.08468	44.1732111	89.7134043	85.1079067
75.665465	56.4163118	62.7832438	57.3668896	34.8769011	44.25784	38.808223
204.237918	141.16128	146.577921	148.633327	92.9211301	115.271293	115.60593
3.36552457	5.2801286	6.29570516	5.3903151	7.05092586	7.74657087	7.88036173
0.30595678	1.11737161	1.98913384	1.18873869	11.4577545	8.90565878	11.2371911
11.4616116	12.9921836	14.9860957	15.0027021	18.5296653	19.781767	21.8807966
1.9769515	4.42566795	3.8623958	3.75067553	1.3010637	4.53976098	4.62587468
1.29443253	1.97183226	0.98491093	1.43468463	4.67963234	2.64658406	2.96792845
19.9107258	29.4022099	32.5020607	25.9268008	40.9205519	24.7272088	28.0827436
3.34198943	4.71048817	3.64996403	3.0948197	6.4843336	6.79998241	6.26335244
1.76513526	3.74648129	3.76583591	2.7054053	3.63038742	7.59202581	4.52353232
7.17821674	10.0782538	9.77186138	8.71058524	10.9121472	14.1215544	13.9390295
0.28242164	0.46009419	0.0965599	0.47139638	0.77644124	1.00454286	1.22810832
3.1301732	5.41158408	6.04464943	5.90270247	9.63206836	9.34997581	8.69910062
7.41356811	9.17997462	10.9112681	9.50990953	13.3463954	14.5272351	12.1787409
19.3458825	13.5180056	12.2631067	12.5432427	11.8774525	7.765889	9.21081242
74.1827514	35.9530748	36.9631278	35.7236472	20.1455025	23.2783489	23.4773374
5.3660112	3.98748301	6.39226506	6.96846819	8.98153651	11.0692895	8.18738881
5.41308148	7.53678107	7.68616765	5.94369346	12.1082864	9.25338515	9.35409172
5.03651929	6.17840774	6.81712859	6.90698171	7.36569934	9.4658846	9.59971338
4.49521114	5.54303957	6.68194474	6.88648621	5.47705848	9.71702032	6.20194703
2.16523259	2.76056516	3.39890831	3.21779266	5.37213399	3.53521813	6.3656948
5.74257339	6.63850193	7.84066348	7.09144116	9.31729489	12.0931505	14.7577683
0.30595678	0.67918667	0.28967969	0.26644143	2.68606699	1.08181538	1.3918561
2.02402177	1.94992301	2.10500571	2.99234222	4.07107029	4.82953296	5.58789287
12.5206928	6.39750021	6.39226506	6.82499973	3.9031911	2.35681208	4.78962246
13.1796766	6.35368172	7.12612026	8.60810777	2.37129352	5.94998461	5.77210911
11.7675684	14.1533738	14.6384801	15.0846841	17.7112542	20.1294934	22.8632833
37.5385433	25.4366361	28.562417	28.3042782	15.4029154	24.7851631	24.9305989
2.70654074	5.58685806	4.34519528	4.30405388	5.70789236	4.71362417	4.85102787
17.6984229	12.5759079	11.5292515	13.1581076	10.1776757	12.0738324	12.8542004
1.74160013	3.46166107	3.57271612	2.43896387	3.65137232	2.93635604	3.62291955
0.32949192	0.63536817	1.08147083	0.69684682	2.09848984	1.19772417	1.55560387
1.05908116	2.84820215	2.08569373	1.78310804	2.05652004	2.56931153	2.84511761
0	0.3724572	0.34761562	0.57387385	1.86765596	1.46817802	0.57311722
1.83574067	3.43975183	3.76583591	3.99662146	7.91130669	5.29316812	4.42118996
1.43564335	2.65101892	1.52564634	2.04954947	4.51175315	2.22158516	2.94745997
6.51923291	7.07668688	10.1774129	9.03851315	8.87661202	12.749967	14.3688674
2.09462718	2.47574494	3.36028435	2.95135123	3.52546293	5.09998681	6.05866772
1.01201088	1.97183226	2.3946854	1.61914408	2.6650821	2.72385659	2.98839692

1.41210821	1.84037677	2.0277578	1.33220715	3.54644783	2.91703791	2.49715359
6.8957951	8.4788787	9.05731816	8.50563029	15.906553	12.0351961	12.3629571
13.2267469	14.4162847	16.7048619	17.6876119	40.3539596	22.0613066	21.7375173
15.0389525	10.1439815	12.0313629	10.4527023	6.37940911	13.5033742	11.9740561
2.65947046	2.97965763	2.22087759	3.75067553	5.81281685	4.71362417	6.42710022
5.64843285	6.39750021	6.6240088	7.3373871	10.1986606	10.837472	12.5676418
4.68349223	9.44288558	9.98429315	11.7029275	21.6354302	21.7328983	27.6529057
1.24736225	1.59937505	1.62220624	1.27072067	4.42781356	3.16817362	2.45621664
8.4961844	4.79812516	5.29148225	5.10337818	2.30833882	4.23067087	2.57902748
6.35448695	12.312997	10.8340202	10.7396392	2.70705189	12.9045121	13.4068492
4.84823819	6.83568516	5.73565777	6.02567544	6.96698627	8.51929614	8.08504645
45.1874628	31.3740421	33.9504591	34.7398635	22.097098	20.3999472	21.3076794
3.78915703	3.89984602	5.50391402	6.10765741	7.26077484	9.98747416	9.35409172
5.27187066	8.85133591	6.19914527	8.01373842	4.88948133	9.44656647	13.52966
2.75361101	4.4475772	2.99335675	5.10337818	2.68606699	4.7329423	6.09960467
10.7555575	6.72613892	7.33855203	6.84549522	5.68690746	6.70339175	5.42414509
2.70654074	1.09546236	1.1973427	0.45090088	0.44068287	0.94658846	0.32749555
1.27089739	0	0.05793594	0.16396396	0	0.21249945	0.45030638
4.30693004	5.12676387	3.76583591	5.75923401	8.20509527	5.83407582	7.57333465
0.09414055	0.94209763	1.02353489	0.22545044	4.80554173	2.72385659	2.2720004

Table S4: List of Reagents, data and software used in this study with source and identifier.

REAGENT or RESOURCE	SOURCE	IDENTIFIER
Chemicals		
DMEM	GIBCO	Cat# 11965-092
RPMI	GIBCO	Cat# 11875-093
Trypsin-EDTA	GIBCO	Cat# 25200-056
Fetal Bovine Serum	GIBCO	Cat# 10438-026
Penicillin-Streptomycin	GIBCO	Cat# 15140-122
Agarose, Low gelling	Sigma-Aldrich	Cat# A9045
Real Time-Glo Annexin V apoptosis reagent	Promega Corp	Cat# JA1011
Matrigel Invasion Chamber with 8.0 µm PET Membrane	Corning	Cat# 354483
Antibodies		
Cathepsin B (D1C7Y) XP(R) Rabbit mAb	Cell Signaling Technology	31718T
p21 (CDKN1A) Rabbit mAb	Cell Signaling Technology	2947T
MDM2 (D1V2Z) Rabbit mAb	Cell Signaling Technology	86934S
Bax Antibody Rabbit mAb	Cell Signaling Technology	2772T
Cathepsin S Antibody	Cell Signaling Technology	25084S
β-Actin (D6A8) Rabbit mAb	Cell Signaling Technology	8457L
COL6A1 (B-4) Mouse mAb	Santa Cruz	sc-377143
Deposited Data		
RNA-Seq performed with TP-472-treated A375 cells	This paper	GEO: GSE179079
Experimental Models: Cell Lines		
A375-MA2	ATCC	ATCC CRL-3223
A375	ATCC	ATCC CRL-1619
M14	NCI-60	
SKMEL-28	MSKCC	
A2058	ATCC	ATCC CRL-11147
Experimental Models: Organisms/Strains		
Mouse: NSG	Jackson Laboratory	Stock No. 005557
Drugs		
TP-472	Tocris	Cat.No.# 6000
Structural Genome Consortium's epigenetic chemical probe inhibitor library	Cayman chemicals	Cat.No.17525
Software and Algorithms		
Prism 8.0	GraphPad	www.graphpad.com/scientific software/prism
ImageJ	https://imagej.nih.gov/ij	N/A
Other		
Reactome pathway analysis from RNA sequencing data	Reactome Pathway Database	https://reactome.org/
mRNA expression in normal and Melanoma cancer samples were analyzed and represented as box plot.	Oncomine Research Premium Edition	https://www.oncomine.org/resource/login.html
The Human Protein Atlas	The Human Tissue Atlas Dataset	https://www.proteinatlas.org/
